# Integrated Multiomics Reveals Alterations in Paucimannose and Complex Type *N*-Glycans in Cardiac Tissue of Patients with COVID-19

**DOI:** 10.1016/j.mcpro.2025.100929

**Published:** 2025-02-22

**Authors:** Sabarinath Peruvemba Subramanian, Melinda Wojtkiewicz, Fang Yu, Chase Castro, Erin N. Schuette, Jocelyn Rodriguez-Paar, Jared Churko, Pranav Renavikar, Daniel Anderson, Claudius Mahr, Rebekah L. Gundry

**Affiliations:** 1 CardiOmics Program, Center for Heart and Vascular Research, and Department of Cellular and Integrative Physiology, University of Nebraska Medical Center, Omaha, Nebraska, USA; 2 Department of Biostatistics, University of Nebraska Medical Center, Omaha, Nebraska, USA; 3 Department of Cellular and Molecular Medicine, The University of Arizona, Tucson, Arizona, USA; 4 Department of Pathology, Microbiology, and Immunology, University of Nebraska Medical Center, Omaha, Nebraska, USA; 5 Division of Cardiovascular Medicine, Department of Internal Medicine, University of Nebraska Medical Center, Omaha, Nebraska, USA; 6 Institute for Advanced Cardiac Care, Medical City Healthcare, Dallas, Texas, USA

**Keywords:** COVID-19, heart, multiomics, paucimannose, high mannose

## Abstract

Coronavirus infectious disease of 2019 (COVID-19) can lead to cardiac complications, yet the molecular mechanisms driving these effects remain unclear. Protein glycosylation is crucial for viral replication, immune response, and organ function and has been found to change in the lungs and liver of patients with COVID-19. However, how COVID-19 impacts cardiac protein glycosylation has not been defined. Our study combined single nuclei transcriptomics, mass spectrometry (MS)-based glycomics, and lectin-based tissue imaging to investigate alterations in *N-*glycosylation in the human heart post-COVID-19. We identified significant expression differences in glycogenes involved in *N*-glycan biosynthesis and MS analysis revealed a reduction in high mannose and isomers of paucimannose structures post-infection, with changes in paucimannose directly correlating with COVID-19 independent of comorbidities. Our observations suggest that COVID-19 primes cardiac tissues to alter the glycome at all levels, namely, metabolism, nucleotide sugar transport, and glycosyltransferase activity. Given the role of *N-*glycosylation in cardiac function, this study provides a basis for understanding the molecular events leading to cardiac damage post-COVID-19 and informing future therapeutic strategies to treat cardiac complications resulting from coronavirus infections.

## Highlights

• Mass spectrometry and RNAseq reveal alterations in the glycome of COVID-19(+) hearts.
• COVID-19(+) hearts have altered expression of glycogenes for *N*-glycan biosynthesis.
• High mannose and paucimannose structures are decreased in COVID-19(+) hearts.

## Graphical Abstract

**Figure:**
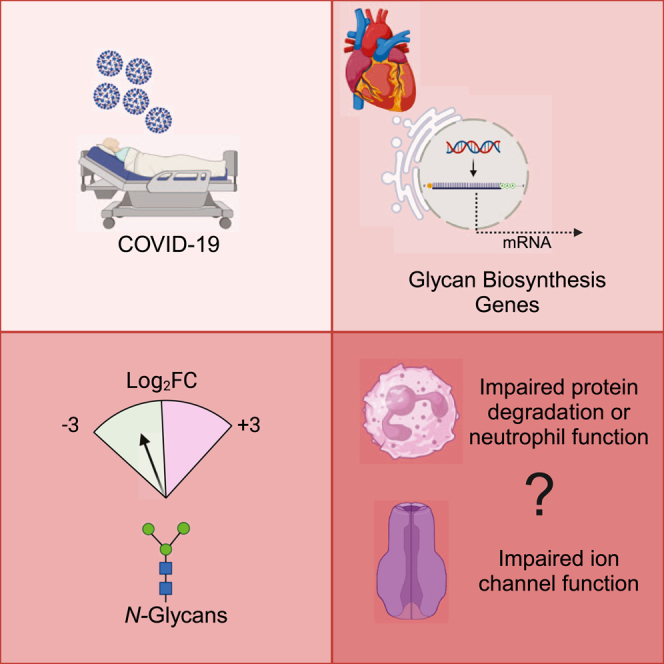

## In Brief

This study identified significant expression differences in glycogenes involved in *N*-glycan biosynthesis and a reduction in high mannose and isomers of paucimannose structures in COVID-19(+) hearts. These observations suggest that SARS-CoV-2 infection primes cardiac cells to alter the glycome with an overall trend to favor synthesis and reduce degradation. As *N-*glycosylation is critical for cardiac function, this work provides a basis for understanding the molecular events leading to cardiac damage post-COVID-19, and in the future may be extended to other viral insults and organs.

Coronavirus infectious disease of 2019 (COVID-19) is a respiratory disease caused by the severe acute respiratory syndrome coronavirus 2 (SARS-CoV-2) (1). In addition to respiratory failure, 10 to 20% of patients develop cardiac complications post-infection including myocardial injury, myocarditis, pericarditis, arrhythmia, ischemic and non-ischemic heart failure, cardiomyopathy, and cardiogenic shock (2, 3). While viral infiltration, cytokine storm, immune cell injury (4, 5), coagulopathies (6), activation of the complement cascade (7, 8), and respiratory insufficiency (9) are ascribed as factors leading to cardiac damage, the specific molecular drivers of these cardiac manifestations of COVID-19 remain elusive.

Glycosylation plays important roles in the viral life cycle, host immune response, and organ function. Glycans mediate the entry, maturation, and exit of viruses from host cells (10, 11). Additionally, viral infections influence the host glycosylation process, which can alter antibody and host cell membrane protein glycosylation and function. Core-fucosylation on antibodies (12) and α2-6 sialylation of complement proteins (13) is crucial for innate immunity and inflammatory responses during infection. In the heart, *N-*glycans on cardiomyocyte cell surface proteins (*e.g.*, corin/furin, α2δ1 subunit of calcium transporter, and G protein-coupled receptors) regulate essential cardiac functions such as cardiac output, electrical conductivity, and contractility (14, 15, 16). Consequently, changes in glycosylation on cell surface proteins are associated with reduced cardiac output, dilated cardiomyopathies, and arrhythmias (17, 18). Thus, if COVID-19 affects the molecular composition of glycoproteins in the heart, this could influence cell and organ function and contribute to adverse cardiac outcomes. While alterations in protein glycosylation have been reported in the lungs and liver of COVID-19 patients (13), glycosylation changes in the heart due to COVID-19 and their impact on heart function have not been reported.

We combined single nuclei transcriptomics, lectin-based tissue imaging, and high-resolution mass spectrometry (MS)-based glycomic analysis of cardiac tissue and isolated cardiomyocytes to discover previously undescribed differences in glycogene expression and *N-*glycan structures correlating with COVID-19. This study provides insights into alterations of protein glycosylation in the human heart due to COVID-19 and provides a framework for understanding glycosylation changes related to cellular alteration that will inform the future development of therapeutic strategies to treat adverse cardiovascular events resulting from coronavirus infections.

## Experimental Procedures

### Extracting Glycogene Data From Published snRNA-seq Data

**Fig. 1:**
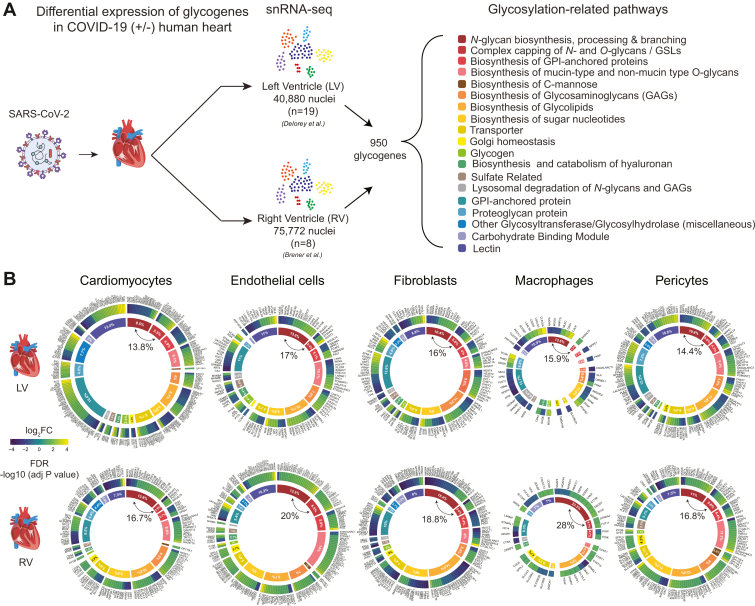
**Summary of snRNA-seq analysis of glycogenes in heart cells from COVID-19(+/) patients.***A*, schematic summary of the bioinformatic analysis of 950 glycosylation-related genes from 19 pathways interrogated from previously published snRNA-seq data (19, 20). *B*, relative distribution of glycogenes within individual cells of the *left and right* ventricular regions of the heart is presented as a radial heat map. The *outer circle* represents the gene expression profile of glycogenes having log2FC > ±1 and FDR <0.05. The inner circle represents the percentage contribution of each glycosylation-related pathway normalized to the total abundance. Glycogenes of *N*-glycan biosynthesis having 15 to 20% variation are indicated within the *inner circle*. The color gradients for the *inner* and *outer circles* in *B* correspond to the legend for glycosylation-related pathways in *A* and gene expression fold changes, respectively. Parts of this figure were generated with Biorender.com

Transcriptional changes in glycogenes were determined by interrogating published data from two studies that performed snRNA-seq analysis of cardiac tissue of COVID-19(+/−) patients collected at autopsy (19, 20). First, a reference list of 950 glycosylation-related genes (glycogenes) was downloaded from GlycoMaple (21). Data for these glycogenes were then extracted from the published snRNA-seq data (supplemental Tables S1 and S2). Transcript level changes in glycogenes with a false discovery rate with adjusted *p-value* < 0.05 and log2 fold change (log2FC) greater than +1 or less than −1 were considered significantly different. Differentially expressed glycogenes were then grouped into 19 categories (Fig. 1*A*) based on their involvement in glycosylation pathways to ease visualization and interpretation.

### Patient Cohort for Glycomics and Imaging Studies

Samples were obtained in compliance with institutional review board approvals and collected through the Nebraska Cardiovascular Biobank and Registry at the University of Nebraska Medical Center (PRO643–17-EP) or the Medical College of Wisconsin (IRB PRO00022202). Cardiac tissue from deceased COVID-19(+) donors was collected during autopsy in 2020 and early 2021 before the COVID-19 vaccine was available. The cohort comprised age and sex-matched samples from COVID-19(−) (n = 16, 10 male, six female) and COVID-19(+) (n = 18, 13 male, five female) patients (Table 1). Caucasians were the predominant ethnicity in both groups. Hypertension, obesity, coronary artery disease, cancer, and hyperlipidemia were the most prevalent comorbidities (Table 1). The cause of death for 83% of COVID-19(+) donors was attributed to COVID-19 or COVID-19 complications (supplemental Table S3).

**Table 1:** Patient characteristics for heart samples used for glycomics and imaging

Metadata	Tissue	Tissue	Isolated cardiomyocytes	Isolated cardiomyocytes
COVID-19 status	(−)	(+)	(−)	(+)
Sample Size (N)	16	18	6	3
Age in years (Median)	63	66	58	69
Gender (N)				
Female	5	6	3	1
Male	11	12	3	2
Ethnicity (N)				
Caucasian	13	9	4	2
African American	3	4	1	-
Asian	-	1	1	-
Hispanic	-	3	-	-
Unknown	-	1	-	1

Time from COVID diagnosis until death (days; mean ± SD)	Not applicable	11 (±7)	Not applicable	7 (±5)
Time of death to autopsy (hours; mean ± SD)	14 ± 9.5	26 ± 18.5	11 ± 4	44 ± 45
Anatomical region				
Left Ventricle	4	12	3	3
Ventricular Septum	9	5	-	-
Right ventricle	1	-	-	-
Ventricle, unspecified	2	1	-	-

BMI kg/m^2^ (Mean ± SD)	29.6 ± 4.6	33.3 ± 7.3	29.7 ± 3.4	25.5 ± 8
Cardiac comorbidities				
Hypertension	9	12	5	3
Cardiovascular disease^a^	9	4	-	1
Type 2 diabetes	3	6	1	2
Chronic obstructive pulmonary disease	4	3	2	2

a Cardiovascular disease includes congestive heart failure, ischemic dilated cardiomyopathy, coronary artery disease, non-ischemic cardiomyopathy, systolic congestive heart failure, hypertension, non-ST-elevation myocardial infarction, paroxysmal atrial fibrillation, stroke, mitral valve stenosis, mitral valve regurgitation, ischemic cerebrovascular stroke, myocardial infarction, arteritis, pulmonary embolism, large saddle pulmonary embolism, non-obstructive coronary artery disease, peripheral artery disease, supraventricular tachycardia, unspecified arrhythmia, cardiac allograft vasculopathy, pulmonary atresia, atrial fibrillation. N = number, SD = standard deviation.

### Tissue Collection

For tissue-level glycomics and imaging studies, tissue sections (10 μm) were prepared from formalin-fixed and paraffin-embedded (FFPE) tissue by the Tissue Sciences Facility at UNMC. To enable cardiomyocyte isolation, ∼500 mg of fresh tissue was cryopreserved in 10% dimethyl sulfoxide, 10% fetal bovine serum, 80% Dulbecco’s Modified Eagle Medium and stored at −80 °C until use (22, 23).

### Hematoxylin and Eosin Staining and Imaging of FFPE Tissue Sections

FFPE tissue sections were used for glycomics, as described below, or stained with hematoxylin and eosin (H and E) as described previously (24) and imaged using Biotek Lionheart FX microscope using a bright field 20× objective.

### Preparation of Tissue Sections and Isolated Cardiomyocytes for Glycomics Analysis

FFPE tissue sections were deparaffinized and hydrated according to details in Supplemental Methods. Tissue was scraped from the slide in 50 μl glyPAQ lysis buffer (beta-test version, ProtiFi) with a sterile scalpel and transferred into a 2 ml Precellys Lysing Kit (CK28-R, Bertin, Tech) tube. Lysates from five slides were pooled and protein was extracted by homogenization and sonication using Precellys evolution Homogenizer (Bertin Technologies, France) and UP 200 St Ultrasonic processor (Hielscher). Homogenization was performed at room temperature in hard mode for 30 × 20 s cycles @ 6800 rpm pulse and intermittent 30 s pause cycles. Sonication was performed on an ice bath at 5 × 30 s pulses, 20 s pauses, and 100% power.

Cardiomyocytes were isolated from human heart tissue as described previously (23). Briefly, tissue was thawed and cut into 1 mm^3^ pieces in an ice-cold wash solution (115 mM potassium gluconate, 2.5 mM potassium chloride, 5 mM potassium phosphate monobasic, 2 mM magnesium sulfate, 2 mM calcium chloride, 30 mM sucrose, 10 mM 4-(2-hydroxyethyl)-1-piperazineethanesulfonic acid, pH 7.4). Tissue slices were collected on a 70 μm cell strainer, washed with ice-cold wash solution, and dissociated using collagenase solution (300 U/ml collagenase type 4, 5 U/ml DNase I, and 1 mg/ml trypsin inhibitor in wash solution) in a 50 ml spinner flask at 37 °C. After three cycles of digestions (1 h digestion followed by two 30 min digestions), each fraction containing dissociated cells was filtered through a 200 μm cell strainer. Crude cardiomyocytes in each filtrate were collected by centrifugation (80*g* for 5 min at 4 °C), pooled, and washed with an ice-cold wash solution. Cellular debris and contaminating non-cardiomyocytes cells in the crude cardiomyocyte preparation were removed using Percoll gradient separation followed by negative selection using Dynabeads CD45 (Invitrogen) and Dynabeads CD31 (Invitrogen) to remove immune cells and endothelial cells. The supernatant containing the isolated cardiomyocytes was concentrated by centrifugation and protein was extracted from isolated cardiomyocytes by sonication using UP 200 St Ultrasonic processor in SDS lysis buffer (5% Sodium Dodecyl Sulphate, 50 mM Triethylammonium bicarbonate, pH adjusted to 7.5 with phosphoric acid) for 5 × 30 s pulses, 20 s pauses, and 100% power.

Protein concentration for the FFPE tissue homogenate and cardiomyocytes was determined using the Pierce 660 protein assay reagent per the manufacturer’s instructions. *N-*glycan release and preparation for MS were carried out using a glyPAQ kit per the manufacturer’s instructions. Briefly, 65 μg protein from FFPE samples and cardiomyocytes was reduced, alkylated, neutralized, resuspended in the binding buffer, and loaded onto a protein capture plate. *N-*glycans were released using PNGase F, reduced, and cleaned per the manufacturer’s instructions (25).

### High-Resolution Mass Spectrometry-Based Glycomics

Reduced *N-*glycans were reconstituted in 40 μl MS grade water containing retention time standards and analyzed using porous graphitized column (PGC) liquid chromatography-MS. Glycans were separated using a self-packed PGC column (10 cm × 180 μm) on an UltiMate3000 high-performance liquid chromatography system (Dionex), in line with an M3 MnESI source (Newomics) equipped with five nozzle emitters of 20 μm ID, coupled to an Orbitrap Eclipse Tribrid mass spectrometer (Thermo Fisher Scientific). The sample (5 μl) was directly injected into the column and data were collected as described (25) according to the details described in Supplemental Methods. All samples were run once using a robust LC-MS method incorporating a mixture of dextran polymer (malto-oligosaccharides, DP3-DP10) as an internal control for column stability that delivers root mean square deviation (%CV) between glycan peak areas of <10% (25).

### Compositional and Structural Analysis of N-Glycan MS Data

Raw MS data were imported into Skyline-daily (64 bit) 21.1.1.223 (26) and searched against an in-house curated *N-*glycan transition list generated from FFPE cardiac tissues and cardiomyocytes. Manual peak picking was based on retention time and calculated relative retention time. Three isotopic envelopes with a centroid mass accuracy value of 15 ppm were used for peak integration. Structural assignments were made based on the presence of C^12^ monoisotopic precursor mass, characteristic order of elution on the PGC column, and diagnostic fragment ions observed from collision-induced dissociation-based MS/MS analysis. Structural assignment of precursor mass was performed manually using GlycoWorkBench v2.1 (27). Structural annotation was made based on the characteristic A/X, B/Y, and C/Z product ions in MS/MS scans and elution order described previously (28, 29). Additionally, diagnostic D ions, D ion-18, D ion-221, and E ions were used to identify glycan moieties linked to the 3-Man and 6-Man arm, bisecting type structures and tri-antennary structures, respectively (30). Peak areas for each glycan structure were exported and the relative abundance (in percentage) of each glycan structure to total glycan abundance was calculated [Peak area of individual glycan/Total peak area of all glycan) × 100]. Comparative composition-based analysis was performed using GlyConnect Compozitor (1.0.0) and Glynsight as described (31, 32, 33), and GlyGen Sandbox was used for interrogating enzymes involved in structure formation (34). A summary of glycan class, observed and theoretical *m/z* and mass error, charge state, composition, structure, relative retention time, diagnostic ions, and GlyToucanID is in supplemental Table S4. Annotated MS/MS spectra of all annotated *N-*glycans are in supplemental File S1.

### Statistical Analysis

For glycomics data, relative abundance values were median normalized and log transformed, and LIMMA univariate analysis was used to compare the log2 fold change (log2FC) abundance between COVID(+) and COVID(−) samples *p* value of <0.05 was considered significant. Multivariate analysis was used to compare the log2FC abundance between COVID(+) and COVID(−) samples after adjusting for confounding effects of age, gender, and comorbidities. The Benjamini-Hochberg method was employed for false discovery rate (FDR) calculation and values < 0.05 were considered significant.

## Results

### Differential Expression of Glycogenes in Heart Cells From COVID-19(+) and COVID-19(−) Donors

**Fig. 2:**
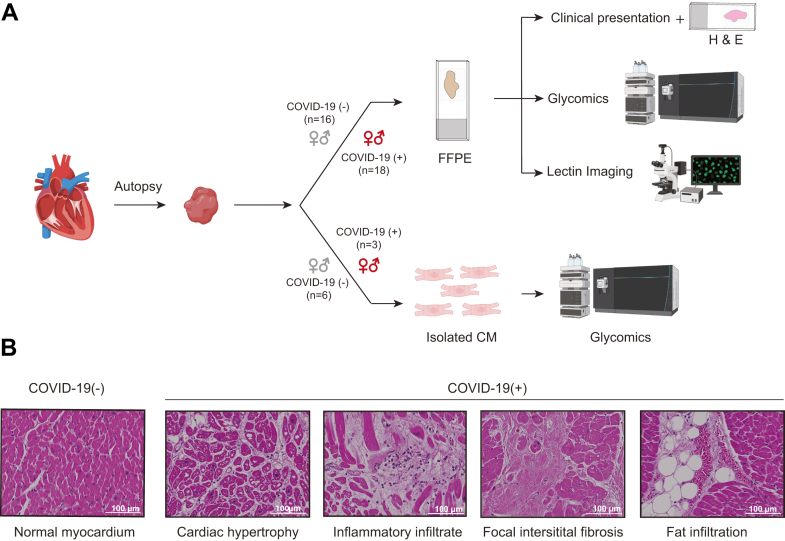
**Overview of experimental design for glycomics and imaging and results of histopathological analyses.***A*, FFPE tissue sections were analyzed by histopathology, MS-based glycomics, and lectin-based imaging. Cardiomyocytes were analyzed by MS-based glycomics. *B*, histopathological findings in COVID-19(+/−) cardiac tissues showing normal cardiac tissue for COVID-19(−) with intact myocyte array and COVID-19(+) tissue with cardiac hypertrophy, inflammatory infiltrate, focal interstitial fibrosis, and fat infiltration. Scale bar = 100 μm. Parts of this figure were generated with Biorender.com.

To gain insights into glycosylation changes associated with COVID-19, we first investigated transcriptional changes in glycogenes. Expression levels of 950 glycogenes from 19 glycosylation-related pathways were extracted from snRNA-seq data from the left and right ventricles from COVID-19(+/−) groups (19, 20) (Fig. 1*A*). Despite differences in sample size and nuclei number, transcriptional changes observed in the left ventricle by Delorey *et al.* (20) are similar to those in the right ventricle reported by Brener *et al.* (19). The COVID(+) group showed significant differences in the expression of glycogenes among different cardiac cell types and between glycosylation-related pathways (Fig. 1*B*). The greatest changes in glycogene expression were observed in cardiomyocytes, endothelial cells, fibroblast, pericytes, immune cells, and vascular smooth muscle cells. In contrast, changes in glycogene expression in macrophages were minimal. The percentage contribution of individual pathways was determined by summing individual glycogenes within a pathway to the total. The percentage distribution of individual pathways was found to vary 1 to 20% of the total (Fig. 1*B*), with genes belonging to the biosynthesis of *N*-glycans, mucin, and non-mucin type *O-*glycans, GPI anchored proteins, glycosaminoglycans (GAGs), glycolipids, nucleotide sugars, and transporters, and lectins being the most remarkable. In contrast, fewer changes were observed in glycogenes of Golgi homeostasis, sulfur-related pathways, and lysosomal degradation of *N-*glycans and GAGs. Among all cell types examined, alteration in glycogene expression was the highest in left ventricle cardiomyocytes, with 247 glycogenes and more than 50% of all genes presenting an increased expression pattern. As glycogenes involved in *N*-glycan biosynthesis were the top variants for both left and right ventricle in the transcriptomic data, the *N-*glycome of cardiac tissue and cardiomyocytes were further examined using lectin-based imaging and MS-based structural glycomics in conjunction with histopathology (Fig. 2*A*).

### Histopathological Changes in Cardiac Tissues

Major histopathological differences between COVID-19(+) and COVID-19(−) heart tissues used for glycomics analysis were revealed by H&E imaging. Myocyte hypertrophy, myocardial fat infiltration, inflammatory infiltrate, and fibrosis were more frequently associated with the COVID-19(+) group when compared to the COVID-19(−) group (Fig. 2*B*). These observations are consistent with pathological changes reported previously (35, 36).

### Lectin Imaging to Decipher Glycosylation Differences Between Patient Groups

To obtain a view of protein glycosylation within the tissue architecture, we applied lectin-based imaging to cardiac tissues. Lectins recognize localization of glycan types, motifs, class or patterns, and overall changes in glycosylation. We tested seven lectins with varying specificities to determine the localization and differences in glycan classes. Lectins Aleuria Aurantia Lectin (AAL), Erythrina cristagalli lectin (ECL), Maackia Amurensis Lectin (MAL), Peanut Agglutinin (PNA), Ricinus Communis Agglutinin (RCA), Sambucus Nigra Agglutinin (SNA) showed cardiomyocyte membranous staining in both COVID-19(−) and COVID-19(+) (supplementary Fig. S1). Vicia Villosa Lectin (VVA) staining was not specific to any cell type in both groups (supplemental Fig. S1). In addition to membranous staining, ECL and SNA also showed some nuclear staining, which was more frequent in the COVID-19(+) group. Lectin staining confirms the presence of Fuc, LacNAc, α2-3 and α2-6 Sia, and Gal moieties. However, staining patterns for all lectins were similar between patients and controls. Lectin-based approaches have been previously applied to study glycan changes in COVID-19 patients, while significant changes in α2-6 Sia and high mannose structures were detected in the lung and liver, no changes were reported for cardiac tissues (13). Our lectin imaging data yielded similar results. The lack of differences observed could be due to the promiscuous nature of lectins in recognizing sugars and the inability to distinguish subtle differences among glycan structures quantitatively. Thus, a large-scale analysis of *N-*glycan isomers using an advanced MS method was employed to examine quantitative differences in glycans.

### MS-Based Glycomics Defines the N-Glycan Profile of Cardiac Tissue and Isolated Cardiomyocytes

**Fig. 3:**
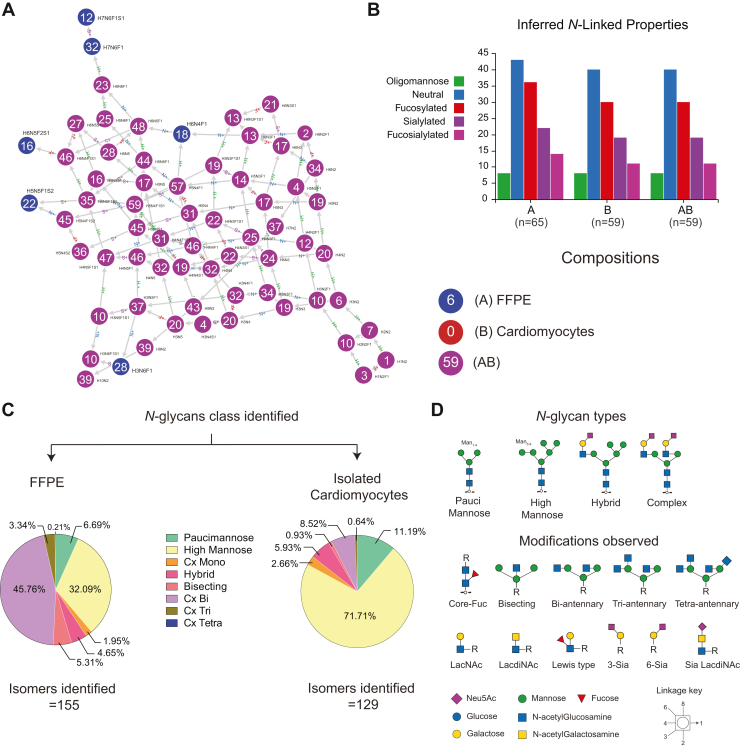
**Qualitative *N-*glycan profile of cardiac tissue and isolated cardiomyocytes.***A*, graph of relatedness of observed *N-*glycan compositions identified in FFPE cardiac tissue slices and isolated cardiomyocytes. Each node represents a unique glycan composition, and two adjacent nodes differ by one monosaccharide residue. *Blue* nodes are observed only in FFPE tissue, and *purple* is observed in both FFPE tissue and isolated cardiomyocytes. *B*, distribution of inferred *N*-glycan composition properties. Graphs in *A* and *B* were generated using Glycompozitor (31). *C*, Pie charts summarizing the percentage distribution of each *N-*glycan class calculated from the relative abundance values. *D*, graphical representation of glycan types identified, and modifications observed at the core and terminal branches.

Glycans are synthesized through a non-template-driven mechanism and exhibit tissue- and cell-type-specific baseline patterns. Therefore, generating a library containing well-established glycan compositions and structures for a given tissue and cell type is critical for comparative analysis. As a first step, we established the repertoire of *N-*glycans in heart tissue and cardiomyocytes from COVID-19(−) samples. We identified 155 and 129 *N-*glycans from FFPE tissue and cardiomyocytes, respectively, including *N-*glycans from all classes and multiple structural isomers (supplemental File S1 and supplemental Table S4). Glyconnect Compozitor was used to examine and fine-tune proposed compositions, ascertain potential gaps in observed glycan compositions, and identify similarities and differences in glycan compositions in FFPE sections and cardiomyocytes. Graphical representation of the relatedness of identified glycans reveals completeness in structures annotated consistently between sample types (Fig. 3*A*). A total of 65 nodes (unique compositions present in the query where two adjacent nodes differ by one monosaccharide residue) were identified with the majority of inferred *N-*glycans composed of neutral, fuco, sialylated, fuco-sialylated, and high-mannoses in descending order of abundance (Fig. 3*B*). No significant differences in overall composition were observed between cardiomyocytes and FFPE tissues, but 26 *N-*glycan structures were observed only in FFPE tissue slices and not cardiomyocytes, including H7N6F1S1, H7N6F1, H6N4F1, H6N5F2S1, H5N5F1S2, and H3N6F1 (H: hex, N: HexNAc, F: Fuc, S: Sialic acid). Altogether, these results represent an updated repertoire of *N-*glycans identified in cardiac tissue with composition level analysis showing complete coverage of the biosynthetic pathway of *N-*glycosylation. This library surpasses our previous report which identified 98 *N-*glycans in human heart tissue (37). We attribute this expanded coverage to technical improvements in sample preparation using the glyPAQ kit and improved ionization and sensitivity using the M3 multinozzle emitter during mass spectrometry analysis (25).

Glycan compositions can be further summarized into individual classes that are known to modulate protein structure and function. Paucimannose, high mannose, complex, and hybrid are major classes of *N-*glycans. As each glycan class is composed of multiple individual glycan structures, the structural details provided by our high-resolution glycomics method are essential for assessing changes in isomers which may be underrepresented by summaries of class or composition. For this reason, we analyzed the qualitative and quantitative glycomics data at both the composition and structure levels. Percentage distribution was determined by summation of the relative abundance of individual glycans to the total. The cardiac tissue *N*-glycome was composed of 46% complex-bi antennary type structures and 32% high mannose structures, followed by 6.7% paucimannose, 2% complex-mono, 4.6% hybrid, 5.3% bisecting, 3.3% complex-tri, and 0.2% tetra-antennary structures (Fig. 3*C*). In contrast, cardiomyocytes were comprised of 72% high mannose and 11% paucimannose structures, followed by 2.5% complex-mono, 6% hybrid, 1% bisecting, 8.2% complex-bi, and 0.6% complex-tri antennary structures (Fig. 3*C*). Complex tetra-antennary structures were not detected in cardiomyocytes. Further structural analysis also revealed subtle modifications occurring at the core and termini of the glycan structure (Fig. 3*D*). *N-*glycans from cardiomyocytes and FFPE tissue slices contained mammalian-type core-chitibiose modification (α1-6Fuc), N-acetyllactosamine (LacNAc), terminal sialylation (α2-6/3 NeuAc), bisecting (β1-4GlcNAc), and LacdiNAc (GalNAcβ1-4GlcNAc) with and without terminal neuraminic acid were the major structural components. α2-3 and α2-6 linked NeuAc was found in relatively equal abundance. Lewis-type motif (GlcNAcα1-3/4Fuc) was present in very low levels. Core-fucosylation was more abundant than terminal fucose. Interestingly, core-fucosylated high mannose (Man_6_Fuc (M-H)^2-^ m/z 771), a structure not very commonly reported in the human glycome, was also detected. This diversity in glycan structures observed by MS can be correlated to the activity of enzymes found in transcriptomic data including FUT8 (core-fucosylation), FUT2/3 (terminal fucosylation), B4GALNT (LacdiNAc), MGAT3 (bisecting GlcNAc), ST6GAL (α2,6 sialylation), and ST3GAL (α2,3 sialylation) (38).

From composition and structural analysis, it is evident that the *N-*glycome of whole tissue and cardiomyocytes differ qualitatively and quantitatively. Cardiomyocytes are the major contributors of high mannose-type structures in cardiac tissue, whereas the major contribution of complex-type *N-*glycans originates from other cell types such as immune cells, fibroblast, and vascular cells. Low abundance of complex and hybrid-type *N-*glycans relative to high mannose glycans in cardiomyocytes suggests there may be specific and subtle roles for these glycans or reduced expression of enzymes required for their biosynthesis.

### MS-Based Glycomics Reveals Differential Abundance of N-Glycans in COVID-19(−) and COVID-19(+) Heart Tissue and Isolated Cardiomyocytes

**Fig. 4:**
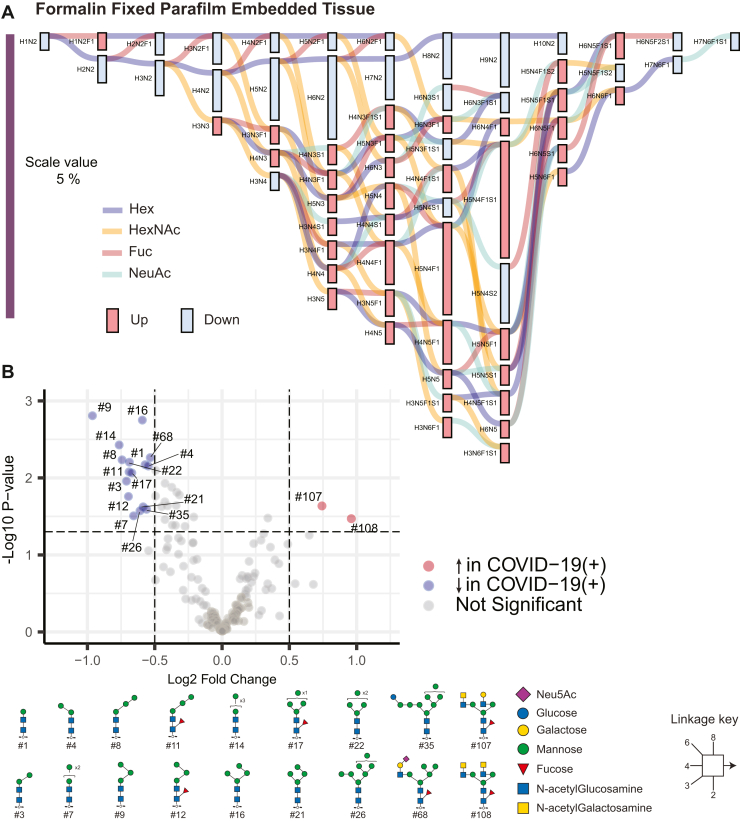
**Quantitative differences in the *N-*glycome of cardiac tissues (FFPE) from COVID-19(+) and COVID-19(−) patients.***A*, compositional analysis of *N-*glycans in cardiac tissue. Graphical results display output from differential expression analysis using Glynsight. Hex (H), HexNAc (N), Fuc (F), and Sia (S) are monosaccharide moieties that form glycan structures. *Red* and *blue* bars depict differences in relative abundance. *Colored lines* indicate the addition of monosaccharide moieties to a given composition. A downward trend (*blue bar*) in H_n_N_2_, corresponding to paucimannose, and oligo mannose indicates reduced levels, and an upward trend (*red bars*) corresponding to H_n_N_n_FS for complex and hybrid structures, indicates an overall increase in abundance. *B*, Volcano plot displaying *N-*glycan structures that are most significantly different between cardiac tissues from each patient group.

Utilizing the in-house generated *N-*glycan library described above, composition and structural differences in the *N-*glycome between COVID-19(−) and COVID-19(+) heart tissue and cardiomyocytes were determined (supplemental Tables S5–S8). Glyinsight (32) was used in differential mode to compare *N-*glycan compositions between COVID-19(−) and COVID-19(+) groups. Differential analysis of *N-*glycans from FFPE sections revealed a reduction in compositions with Hex (H) and HexNAc (N) (where Hex = 2–10 and HexNAc = 2, corresponding to paucimannose and oligomannose structures) and increase in compositions with Hex (H), HexNAc (N), Fuc (F), and Sia (S) (where Hex = >3, HexNAc = >3, Fuc = 1, and Sia = 2, corresponding to complex type structures) in COVID-19 (+) patients. However, several glycans with compositions with Hex (H), HexNAc (N), Fuc (F), and Sia (S) corresponding to complex type structures were reduced in COVID-19 (+) patients (Fig. 4*A*). Following compositional analysis, quantitative differences in individual glycans were determined by univariate analysis. Of 155 *N-*glycans, 18 (∼12%) *N-*glycans were significantly different between COVID-19(−) and COVID-19(+) groups (Fig. 4*B*). Isomers of paucimannose, high mannose, and hybrid-type glycans were significantly reduced and LacDiNAc-type structures in the complex glycan class were elevated in the COVID-19(+) group.

**Fig. 5:**
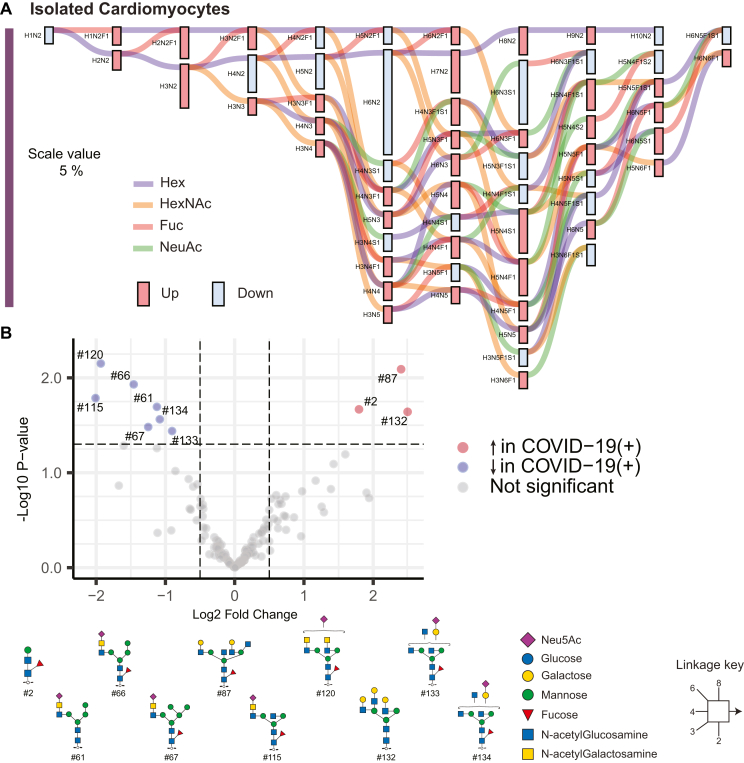
**Quantitative differences in the *N-*glycome of isolated cardiomyocytes from COVID-19(+) and COVID-19(−) patients.***A*, compositional analysis of *N-*glycans from cardiomyocytes. Graphical results display output from differential expression analysis using Glynsight. Hex (H), HexNAc (N), Fuc (F), and Sia (S) are monosaccharide moieties that form glycan structures. *Red* and *blue bars* depict differences in relative abundance. *Colored lines* indicate the addition of monosaccharide moieties to a given composition. A mixed trend in abundance was observed. Overall changes were indicative of increased complex type structures. *B*, Volcano plot displaying *N-*glycan structures that are most significantly different between isolated cardiomyocytes from each patient group.

Unlike FFPE cardiac tissue, *N-*glycan compositions in isolated cardiomyocytes had mixed patterns with few paucimannose, high mannose, and complex structures differing in abundance (supplemental Tables S7 and S8). Specifically, *N-*glycans with compositions corresponding to paucimannoses (H1N2, H4N2, H4N2F1), high mannoses (H5N2, H5N2F1, H10N2), and complex type structures (H4N3S1, H3N4S1, H4N4S1, H3N5F1, H6N3S1, H5N3F1S1, H4N4F1S1, H3N5F1S1, H6N3F1S1, H5N5S1, H4N5F1S1, H3N6F1S1, H5N4F1S2, H6N5F1S1) were decreased in COVID-19 (+) cardiomyocytes (blue bars, Fig. 5*A*), while the majority of paucimannose, high mannose, and complex structures were increased in COVID-19(+) cardiomyocytes (red bars, Fig. 5*A*). Of 129 *N-*glycans, 10 (∼8%) *N-*glycans were significantly different in abundance between COVID-19(−) and COVID-19(+) groups (Fig. 5*B*). Individual glycan types belonging to paucimannose, hybrid, bisecting, complex bi-antennary with LacDiNAc were reduced in cardiomyocytes of COVID-19(+) patients, and complex tri-antennary glycans were significantly reduced and LacDiNAc-type structures in the complex glycan class were elevated in the COVID-19(+) group. These findings reveal distinct variations in *N-*glycan levels within cardiomyocytes compared to whole tissue homogenate. Such differences indicate a potential pathological change in the glycosylation processes caused by the coronavirus, specifically affecting cardiomyocytes in contrast to other cell types.

### Differences in N-Glycans Independent of Age, Sex, or Comorbidity

**Fig. 6:**
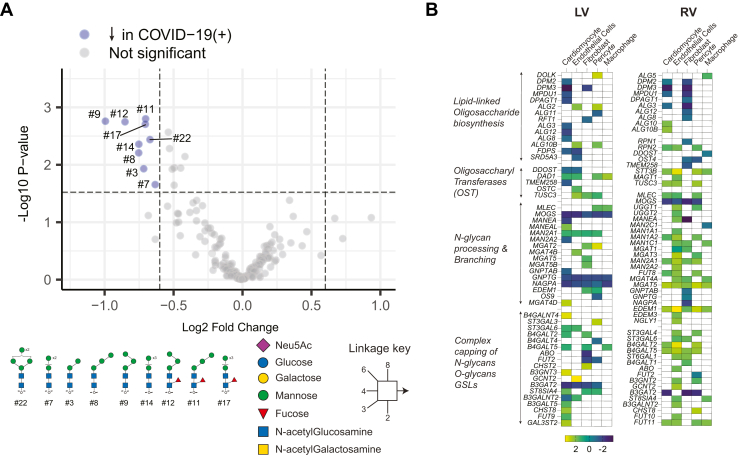
**Differences in FFPE tissue *N-*glycans that are independent of confounding variables**. *A*, median normalized data were subjected to multivariate analysis to determine glycosylation changes independent of confounding factors such as age, sex, and comorbidities. Volcano plot represents differential abundance of glycans between COVID-19(−) and COVID-19(+) groups. Paucimannoses were reduced exclusively due to COVID-19. *B*, heat map representing log2FC changes in expression of various enzymes in *N-*glycan biosynthesis obtained from snRNA-seq analysis (Fig. 1). LV-left ventricle, RV-right ventricle.

Following univariate analysis, multivariate analysis was performed on glycomics data from FFPE samples to identify glycosylation changes specific to COVID-19, independent of confounding variables. Due to the small sample size, multivariate analysis could not be performed on results from isolated cardiomyocytes. Log-transformed median normalized relative abundance values adjusted for age, sex, and comorbidities were used for multivariate analysis. Lower levels of paucimannose glycans, main isomers of Man_2_, Man_3_ (with or without α(1–6) fucose), and Man_4_ in the COVID-19(+) group, were found to be independent of confounding factors (Fig. 6*A*). The decrease in paucimannose structures in cardiac tissue from COVID-19(+) patients, regardless of comorbidities, implies that alterations in paucimannose glycans are the result of COVID-19, and may relate to pathological and cellular changes. While glycan abundance changes are an aggregate of changes in glycogene expression and activity, length of time in the ER/Golgi, and nucleotide precursor availability, in this case, the observed paucimannose abundance changes may be due to a shift in glycan types from high mannose to complex and hybrid structures without the formation of paucimannose, which could be driven by the activity of glycosyltransferases including increased expression of hydrolases, *N-*glycan processing and branching enzymes, and capping in individual cell types observed in snRNA-seq data (Fig. 6*B*).

## Discussion

In this study, we integrated single nuclei transcriptomics, glycomics, and imaging to generate molecular insights into how COVID-19 alters cardiac protein *N-*glycosylation and provide clues into how infection may lead to cardiac complications. Changes observed in glycogene expression across all major cardiac cell types suggest COVID-19 considerably affects host glycosylation machinery, including genes involved in nucleotide sugar biosynthesis and transport, and glycosyltransferases. These results inspired more detailed analyses using a high-resolution MS-based structural glycomics approach. To decipher the composition and structure of glycans, we employed a state-of-the-art sample preparation approach and high-resolution mass spectrometry technique for structural glycomics. These technical advancements led to the identification of 155 and 129 *N-*glycans in FFPE tissue and cardiomyocytes, respectively, from a 65 μg sample. This expands our previous work that reported 98 *N-*glycans identified in human heart tissues and isolated cardiomyocytes (37). Additional glycans identified in the current study included isomers of paucimannose, bisecting, and complex multi-antennary structures. The percentage distribution of glycan class identified here is comparable to our earlier study, with high mannose (71%) and paucimannose (11%) classes being predominant structures in isolated cardiomyocytes (37). As cardiomyocytes make up 50 to 60% of cardiac tissue by cell type (19) a large proportion of high mannose and paucimannose-type glycans observed in the *N*-glycan pool of cardiac tissues can be attributed to cardiomyocytes, whereas complex-type structures arise predominantly from fibroblasts, smooth muscle cells, and others.

Using this expanded *N-*glycan structure library, comparative glycomic analysis of glycans between COVID-19(+) and COVID-19(−) groups revealed a reduction in high mannose and isomers of paucimannose structures, with paucimannose directly correlating with COVID-19, independent of other confounding factors. The function of paucimannose, specifically its role in cardiac tissue, is yet unknown, but may be linked to lysosome function. Manipulation of the autophagy-lysosome system has been identified as a key mechanism in the multiplication of viruses in COVID-19. β-coronaviruses utilize lysosomal trafficking (non-lytic) for cell egress, leading to deacidification, impaired protease activity, and disrupted antigen presentation in infected cells (39). Our analysis of snRNA data from COVID-19(+/−) hearts corroborate aberrant lysosomal activity in cardiac tissues. Lysosomal-associated membrane proteins (LAMP) and lysosomal hydrolases, mannosidases (MANBA), glucosylceramidase beta 1 (GBA), and Asp-glucosaminidases (AGA) were upregulated in COVID-19(+) cardiac tissues. Conversely, GNPTAP and GNPTG, which are key enzymes involved in mannose-6-phosphate formation and lysosomal targeting, were down-regulated in the COVID-19(+) group (supplemental Tables S1 and S2). Glycosylation plays important roles in lysosomal function, including protein-targeting, maintaining structural integrity, protecting from hydrolases, and turnover of glycoproteins in the lysosome (40). Thus, alterations in paucimannoses and high mannose glycans could be attributed to excessive hydrolytic activity in lysosomes, leading to impaired lysosomal function. Targeting the autophagy-lysosomal pathway has been proposed as a strategy to treat COVID-19 (44, 45, 46). Among the various candidates, inhibitors of glycosyl hydrolases or targeting glycan-related genes that aid in lysosomal trafficking present potential therapeutic strategies. Additionally, paucimannose structures are elevated in cancer and inflammation (47), have been linked to modulation of neutrophil-mediated immune function (42, 48) and cell proliferation (49), and may act as a signal to target proteins to extracellular exosomes or to sort proteins to lysosomes (50). Thus, reduced paucimannose observed in COVID-19(+) hearts may be associated with impaired protein degradation or neutrophil function.

Direct premature transfer of paucimannose is another possibility, albeit with low probability based on our current understanding. Paucimannose-type glycans are formed by the action of hexosaminidases (HEXA and HEXB) on hybrid structures, which eventually get pruned by mannosidases, giving rise to Man5-Man3 structures with and without fucosylation (41, 42). However, the exact subcellular localization of this process remains unclear. In neutrophils, secretory granules of neutrophils from pathogen-infected lungs have been found to contain paucimannosylated proteins, suggesting secretory vesicles as possible subcellular sites (42). While one study has shown STT3A (a component of oligosaccharyltransferase complex) can directly transfer Man5 *N-*glycans to protein in *T. brueci* (43), direct evidence for this activity in mammalian cells is lacking.

Together with our observations of differences in baseline *N*-glycan distributions among cardiac cell types and reduced paucimannose in COVID-19(+) cardiac tissue, we posit that perturbations in *N-*glycosylation may play a role in lysosome dysregulation with viral infection. However, it is unclear whether this is due to alterations in glycosylation required for proper function of glycans on the vacuolar H^+^-ATPase needed to maintain lysosome pH (51), host response to infection, viral hijacking of host machinery, or other mechanisms. Furthermore, complex and hybrid-type glycans regulate Ca^2+^ handling and function of voltage-gated Na+ and K+ channels and play pivotal roles in the initiation, shaping, and conduction of cardiomyocyte action potentials (18). Reduction in hybrid structures in isolated cardiomyocytes of COVID(+) patients could dysregulate the electrical conductivity of cardiomyocytes and affect cardiac output and contractility. If so, alterations in hybrid-type glycans could be an additional factor causing arrhythmia and dilated cardiomyopathies in COVID-19 (3, 9). Overall, COVID-19(+) cardiac cells and tissues display altered expression of glycogenes and distribution of *N-*glycans, which provides insights into the molecular underpinnings of adverse cardiac outcomes due to COVID-19.

This study reports differences in *N-*glycosylation in the hearts of COVID-19 patients compared to non-COVID-19. However, there are several limitations. First, snRNA-seq data from published reports were used to obtain an overview of transcriptional changes in glycogenes at the cellular level and are a different patient cohort than used for glycomics; therefore, a direct correlation between glycan structures found and glycosyltransferase/hydrolase expression and their distribution in individual cell types could not be established. Second, despite differences in sample size and nuclei number, some of the differences in glycogene expression observed in the left ventricle by Delorey *et al.* (20), when compared to the right ventricle reported by Brener *et al.* (19), suggest a region-specific glycogene expression as one study used the left ventricle and the other used the right ventricle tissue. However, differences could also be due to different donor pools, inherent differences in cellular distribution, and/or the extent of infection across anatomical regions. As data comparing multiple regions within donors are not available, future analysis of age, sex, and anatomical site-matched samples of the human heart are necessary to determine if cardiac glycogene expression differences are regional. Finally, underlying comorbidities within the cohort used for our study add to the sample heterogeneity and could only be mitigated *via* statistical analysis. Given the practical challenges to biobanking during the pandemic, inclusion and exclusion criteria were not established before collection and the cohort sizes were modest, although consistent with many previously published studies of COVID-19 (19, 20).

In summary, we expanded on our prior *N-*glycome dataset for the human heart, which we expect will inform future studies designed to understand cellular and molecular changes occurring in cardiac disease. Using this library, we observed changes in *N-*glycan structure abundance that occur in the human heart post-COVID-19. Major changes in paucimannose and complex structures were observed in COVID(+) cardiac tissues. Our observations suggest that SARS-CoV-2 infection primes cardiac cells to alter the glycome at all levels, namely metabolism, transport of nucleotide sugar, and glycosyltransferases with an overall trend to favor synthesis and reduce degradation. These changes can be attributed to the viral life cycle (*e.g.*, hijacking host machinery) or cellular transformations occurring during infection. As *N-*glycosylation is critical for maintaining electrical conductivity, cell adhesion, and protein quality control in the heart, this work provides a basis for understanding the molecular events leading to cardiac damage post-COVID-19, and in the future may be extended to other viral insults and organs.

## Data Availability

Raw files from MS has been deposited in GlycoPOST (52). (https://glycopost.glycosmos.org/preview/1409704126670147788a822. Pin=7380).

## Supplemental data

This article contains supplemental data (53).

## Conflict of interest

R. L. G. is on the advisory board of ProtiFi, LLC, and receives no compensation of any kind.
