# Supplementary material for: Integrated Multiomics Reveals Alterations in Paucimannose and Complex Type N-Glycans in Cardiac Tissue of Patients with COVID-19
Source: Mol Cell Proteomics. 2025 Feb 22;24(4):100929. doi: 10.1016/j.mcpro.2025.100929 (PMC12131855; doi:10.1016/j.mcpro.2025.100929)
Supplement: Supplemental_Data_File_1 [file mmc1.pptx]

## Slide 1
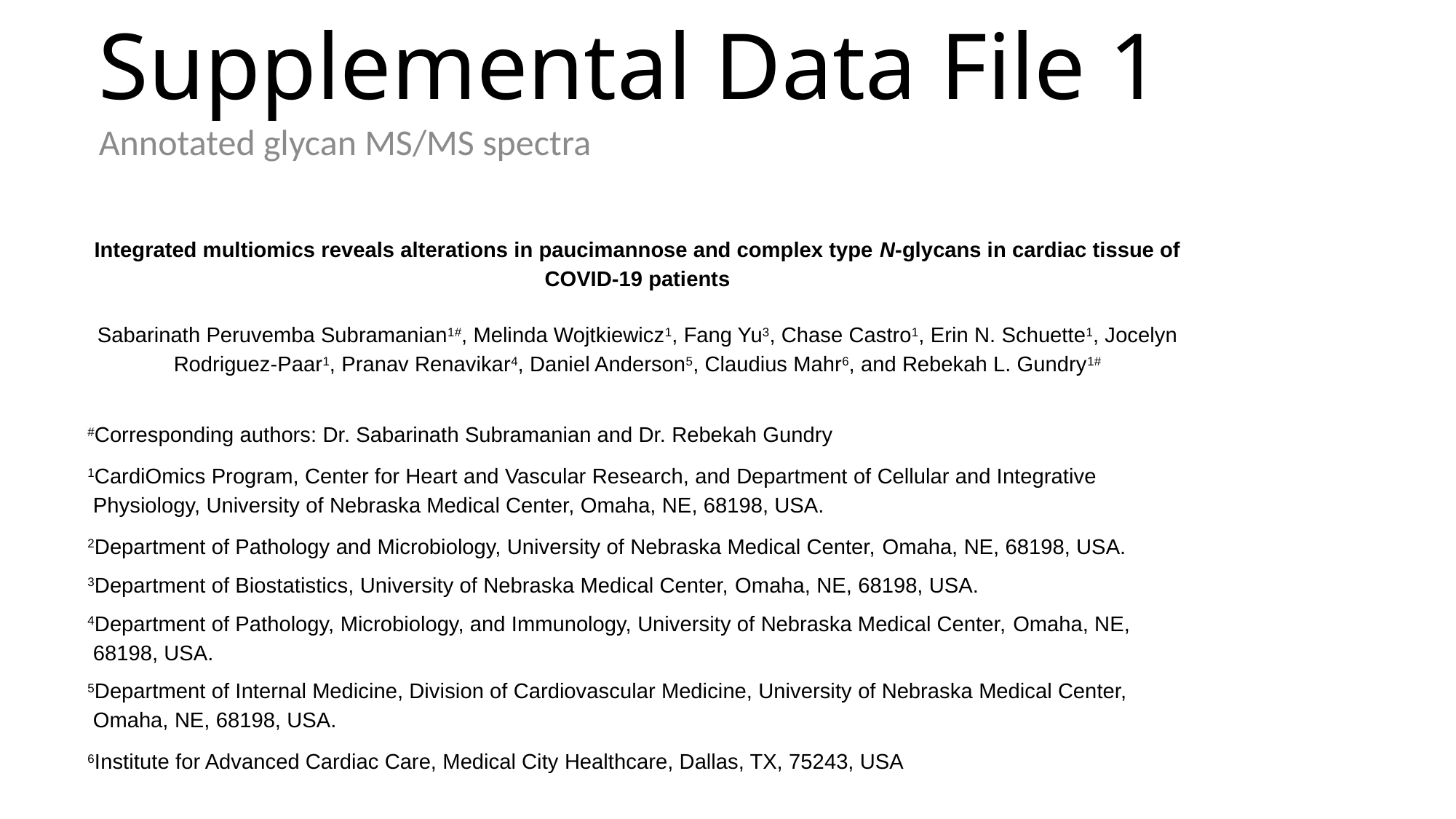

# Supplemental Data File 1
Annotated glycan MS/MS spectra
Integrated multiomics reveals alterations in paucimannose and complex type N-glycans in cardiac tissue of COVID-19 patients
Sabarinath Peruvemba Subramanian1#, Melinda Wojtkiewicz1, Fang Yu3, Chase Castro1, Erin N. Schuette1, Jocelyn Rodriguez-Paar1, Pranav Renavikar4, Daniel Anderson5, Claudius Mahr6, and Rebekah L. Gundry1#
#Corresponding authors: Dr. Sabarinath Subramanian and Dr. Rebekah Gundry
1CardiOmics Program, Center for Heart and Vascular Research, and Department of Cellular and Integrative Physiology, University of Nebraska Medical Center, Omaha, NE, 68198, USA.
2Department of Pathology and Microbiology, University of Nebraska Medical Center, Omaha, NE, 68198, USA.
3Department of Biostatistics, University of Nebraska Medical Center, Omaha, NE, 68198, USA.
4Department of Pathology, Microbiology, and Immunology, University of Nebraska Medical Center, Omaha, NE, 68198, USA.
5Department of Internal Medicine, Division of Cardiovascular Medicine, University of Nebraska Medical Center, Omaha, NE, 68198, USA.
6Institute for Advanced Cardiac Care, Medical City Healthcare, Dallas, TX, 75243, USA

## Slide 2
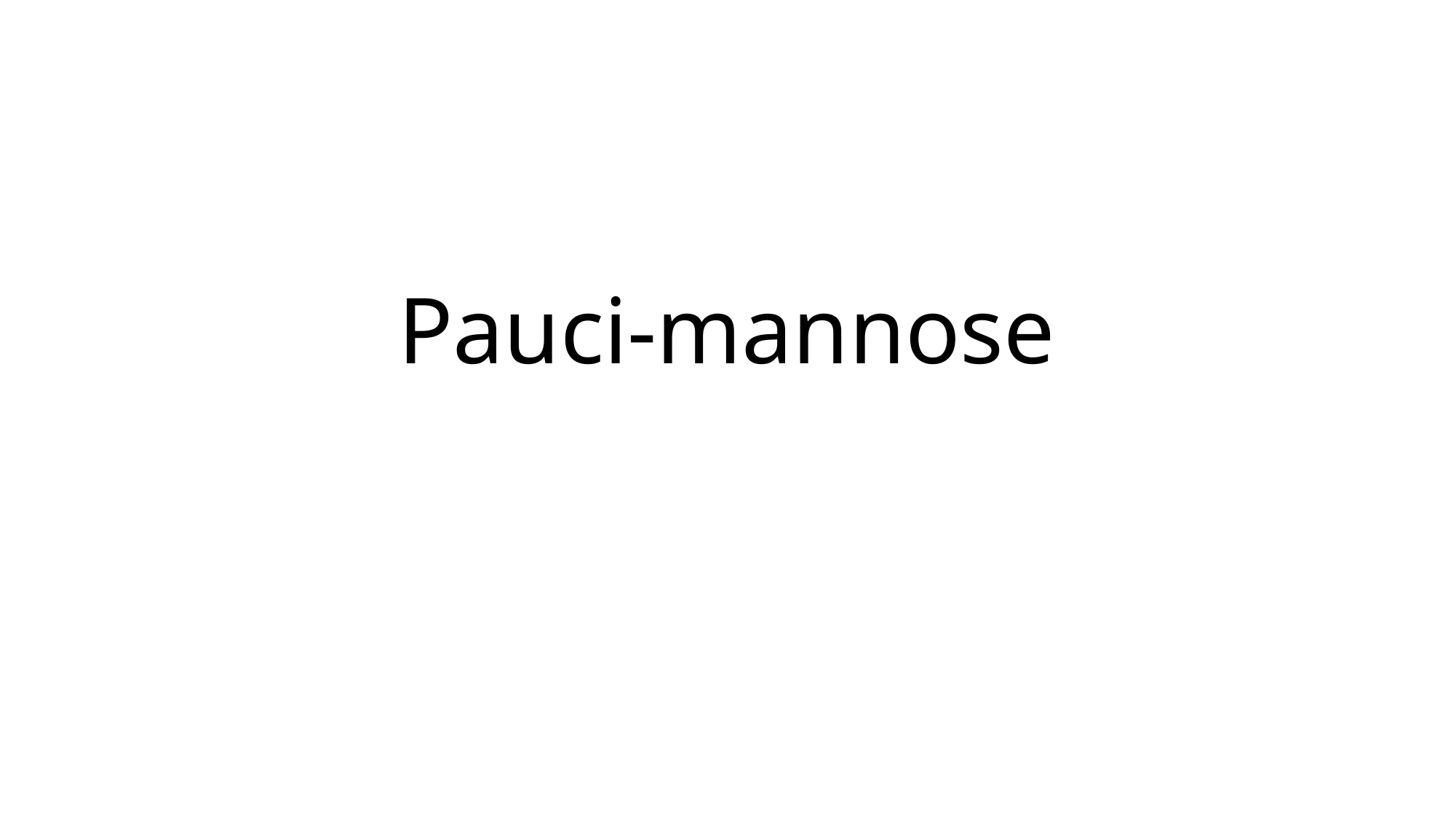

# Pauci-mannose

## Slide 3
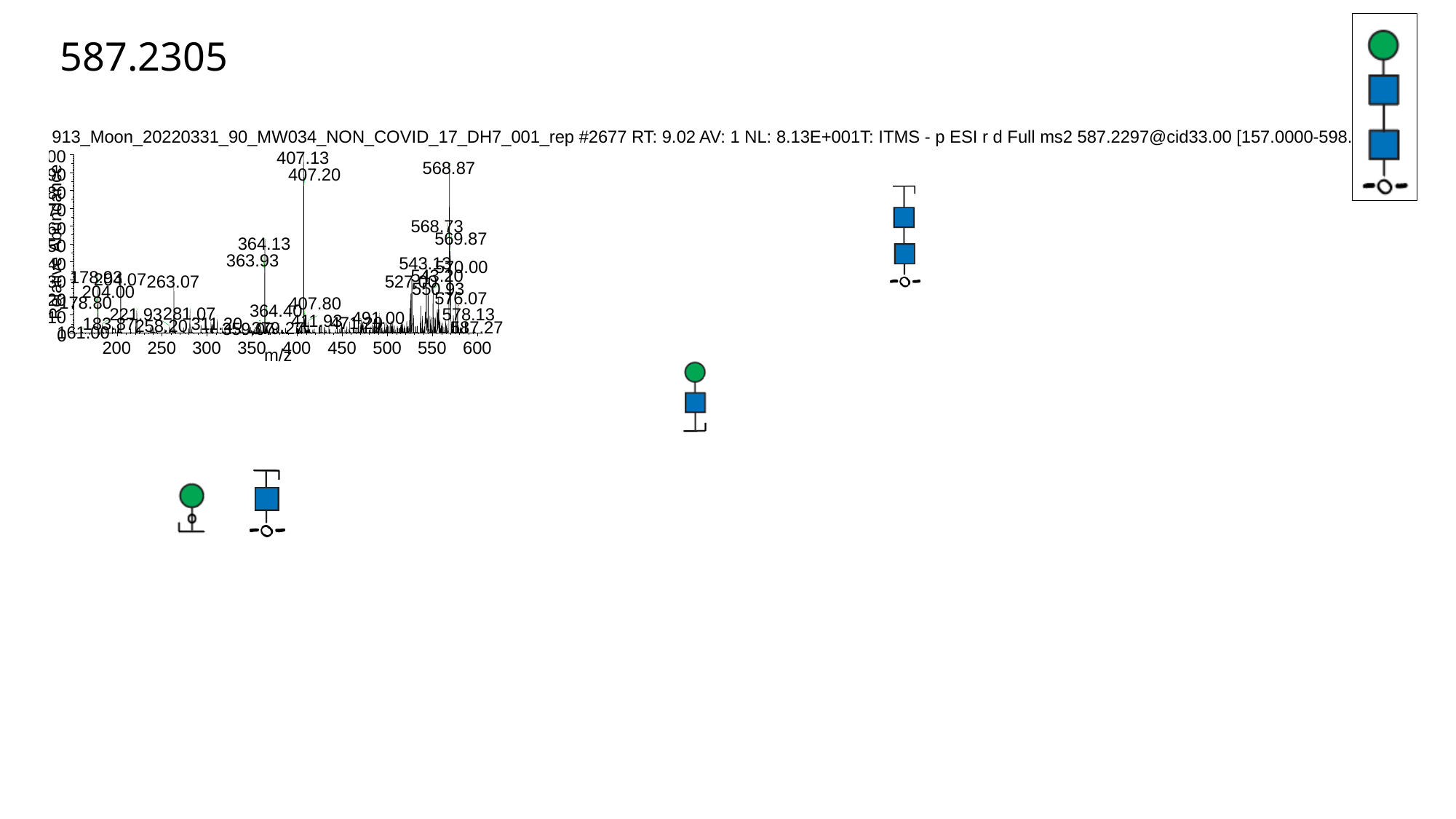

# 587.2305

## Slide 4
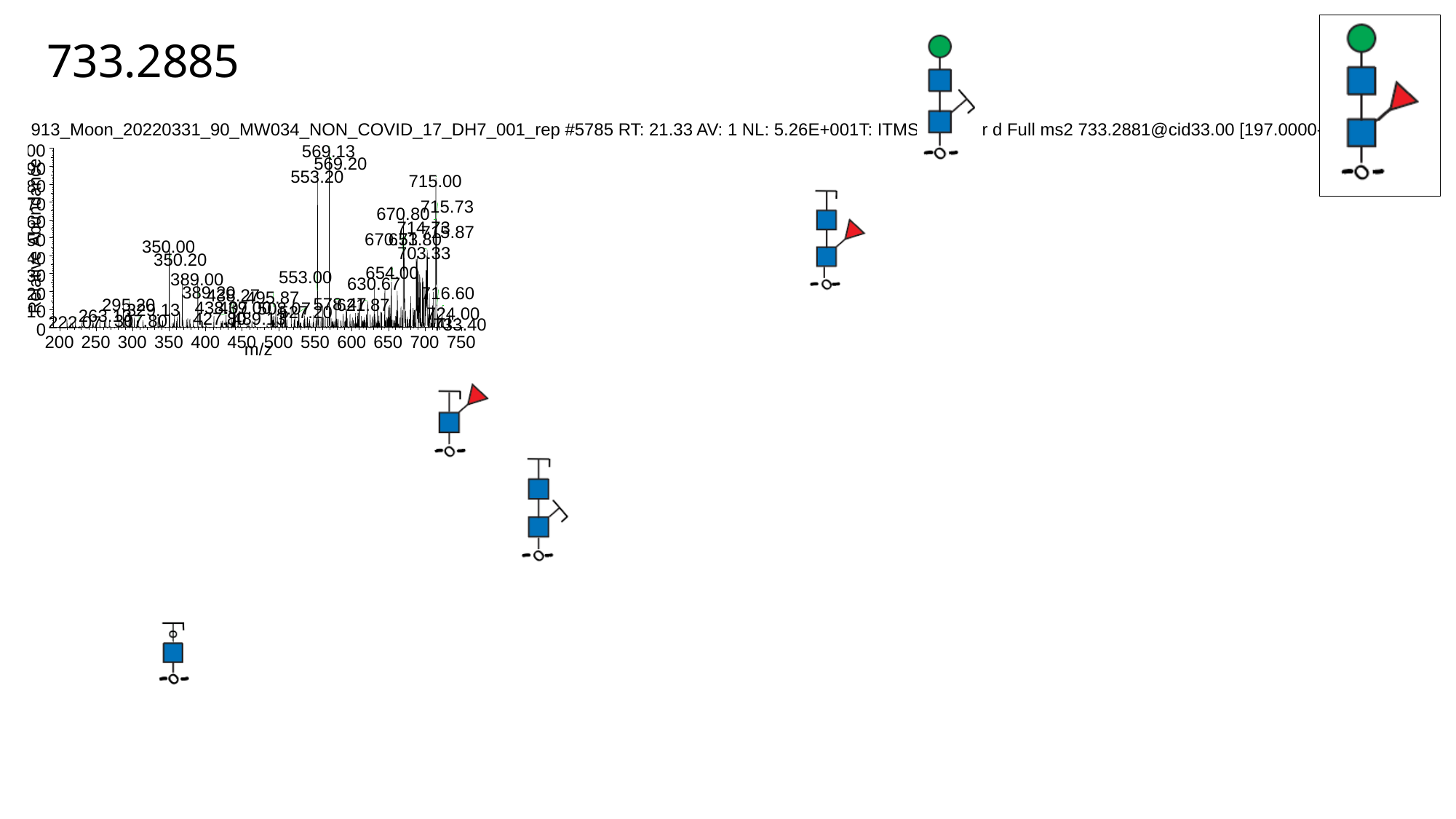

# 733.2885

## Slide 5
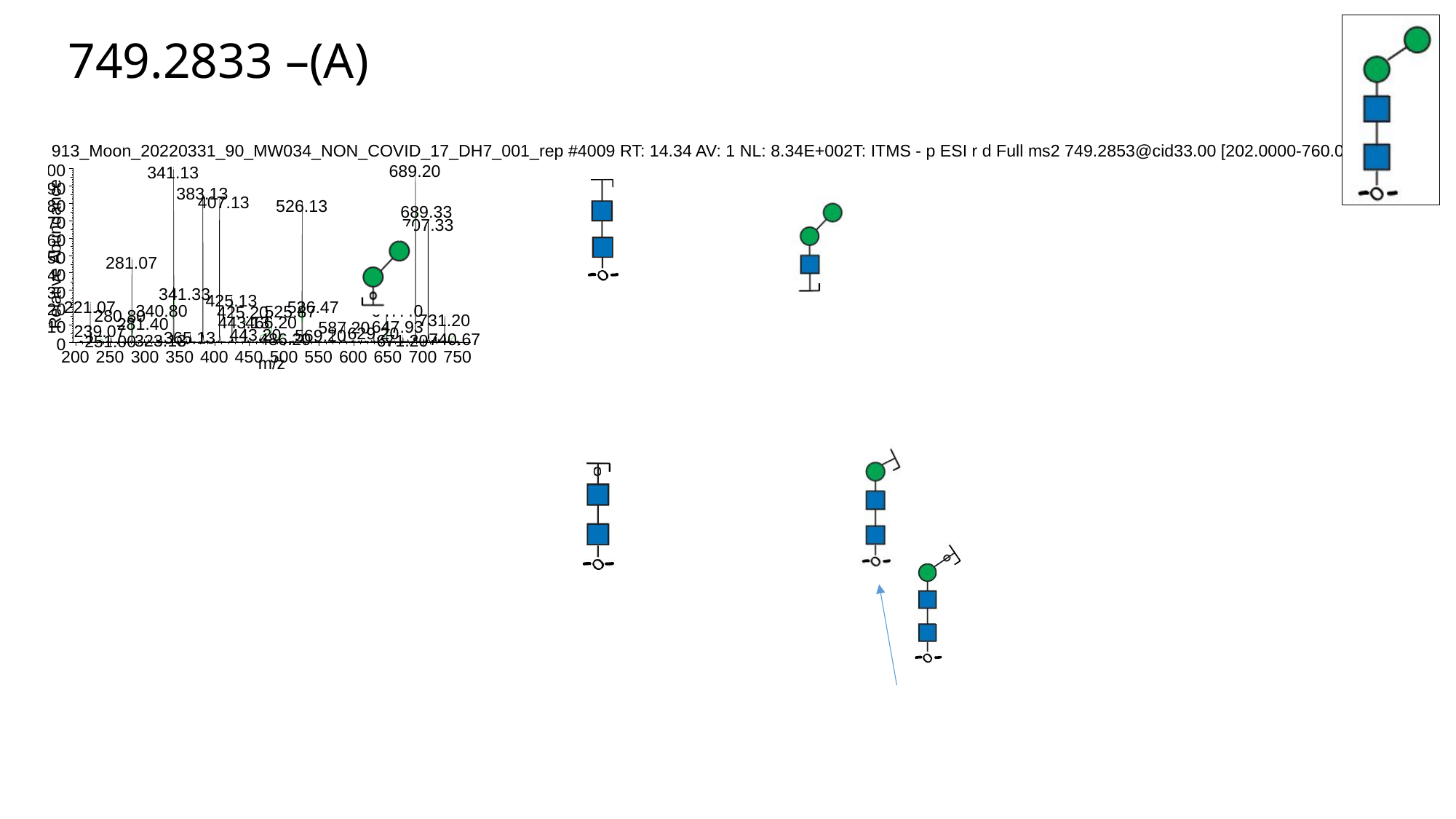

# 749.2833 –(A)

## Slide 6
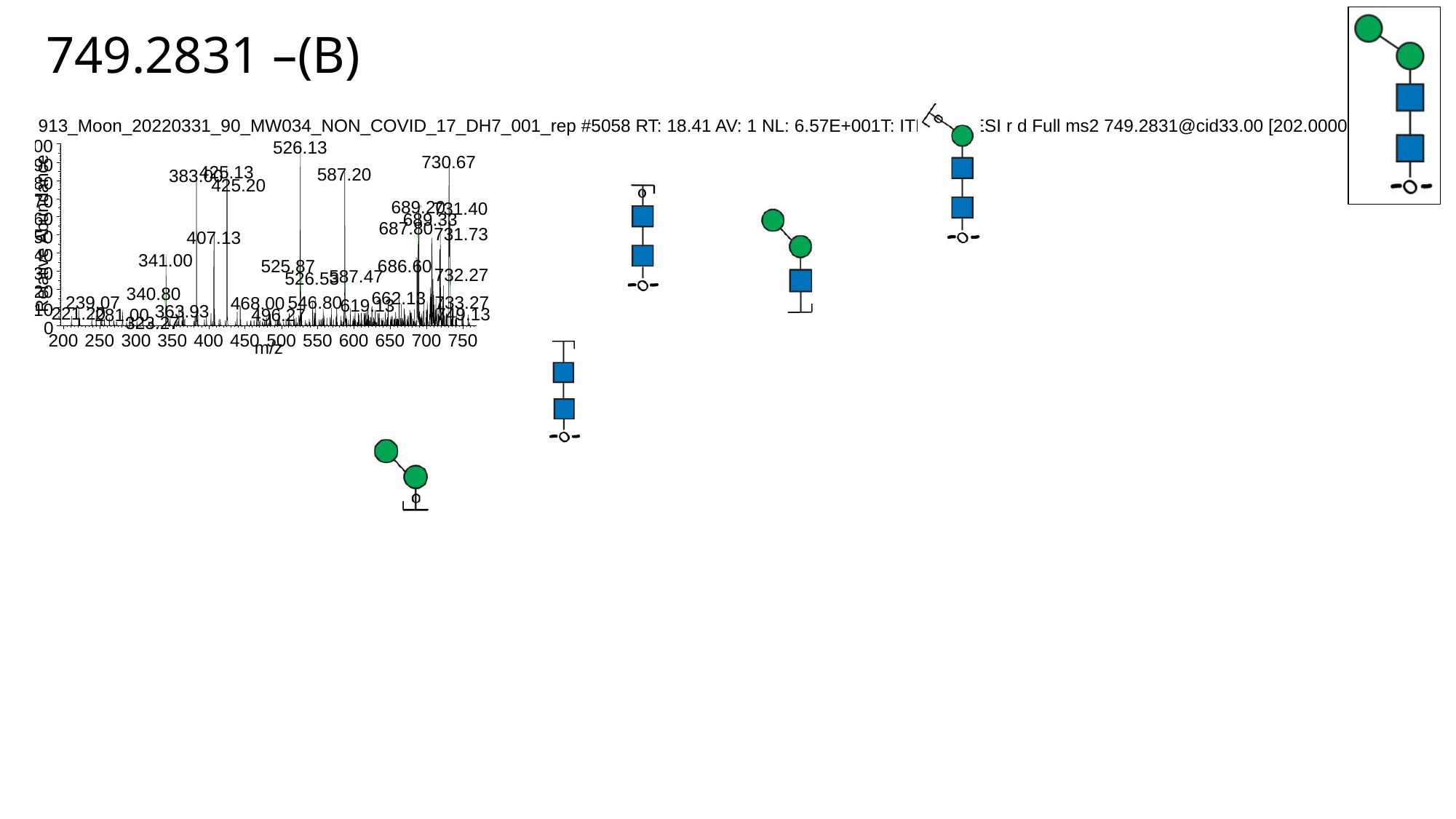

# 749.2831 –(B)

## Slide 7
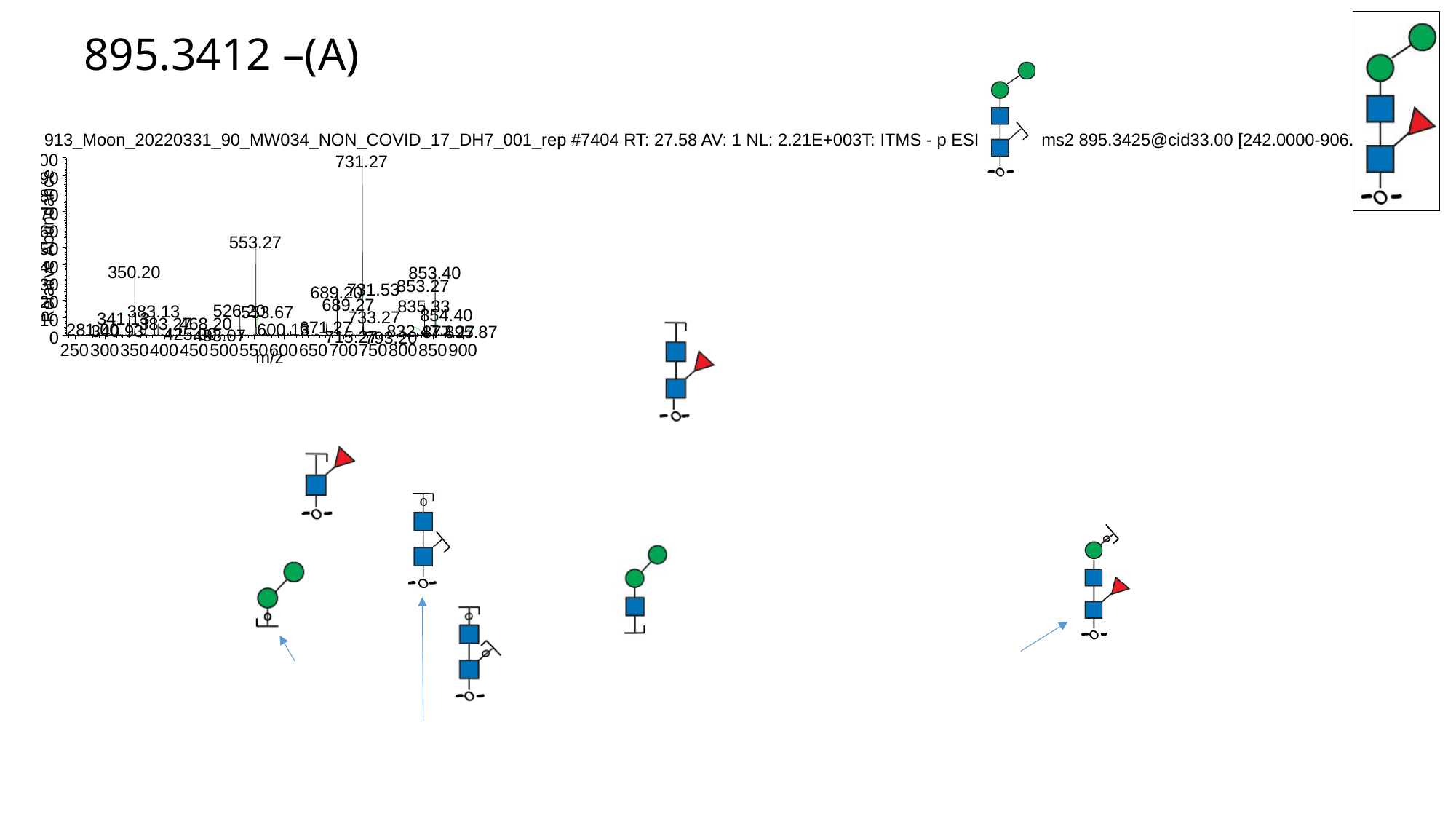

# 895.3412 –(A)

## Slide 8
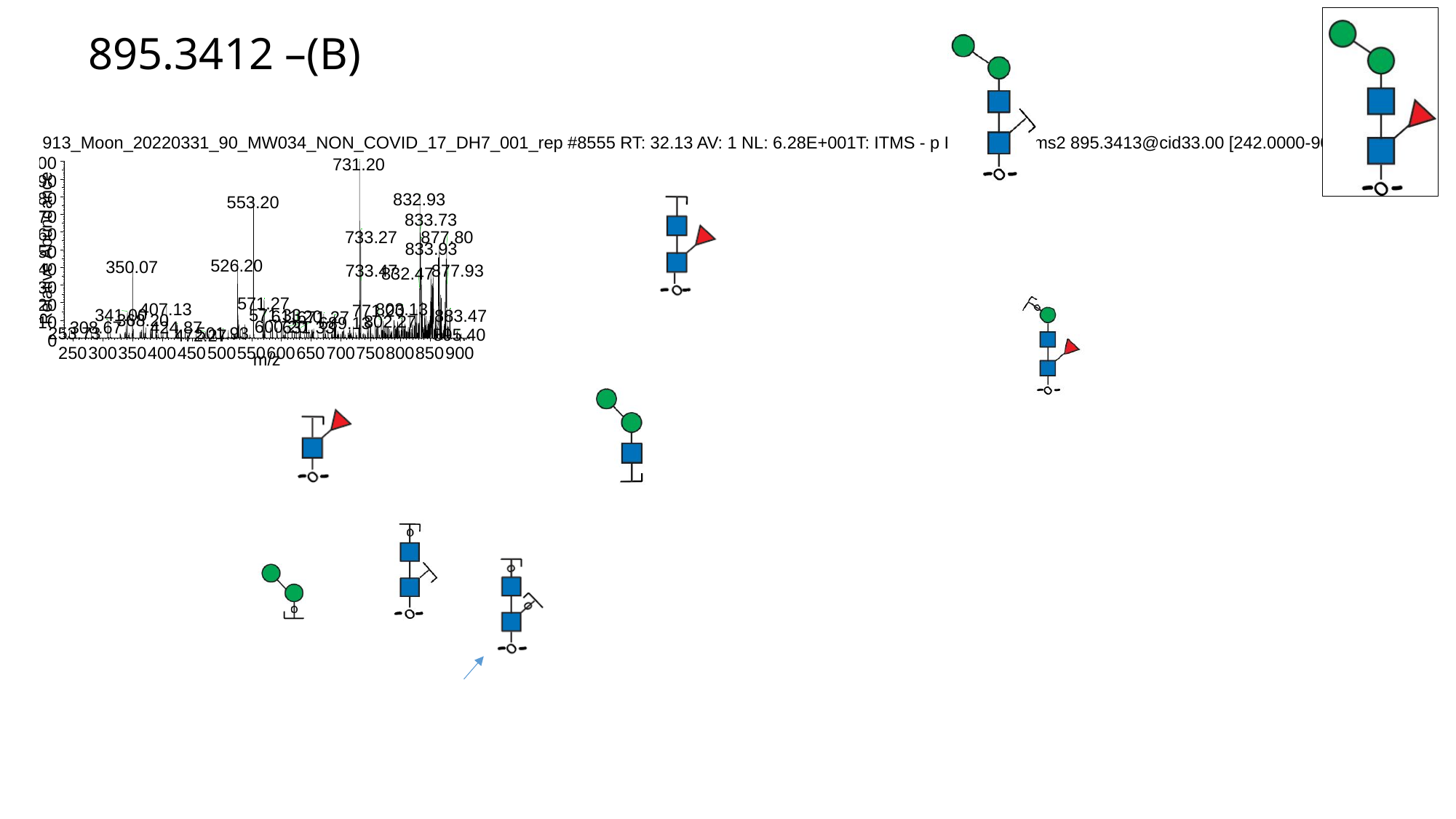

# 895.3412 –(B)

## Slide 9
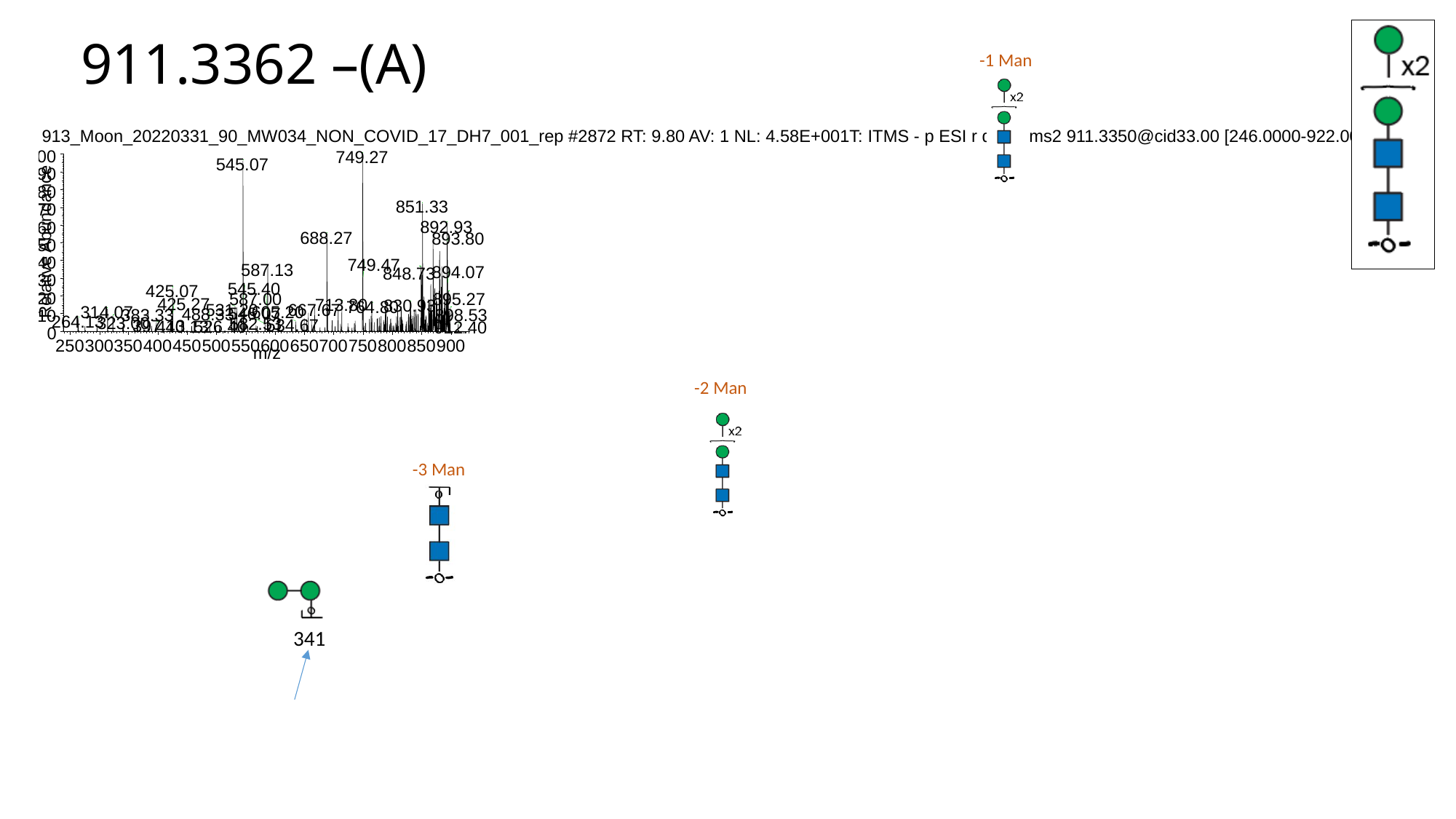

# 911.3362 –(A)
-1 Man
-2 Man
-3 Man
341

## Slide 10
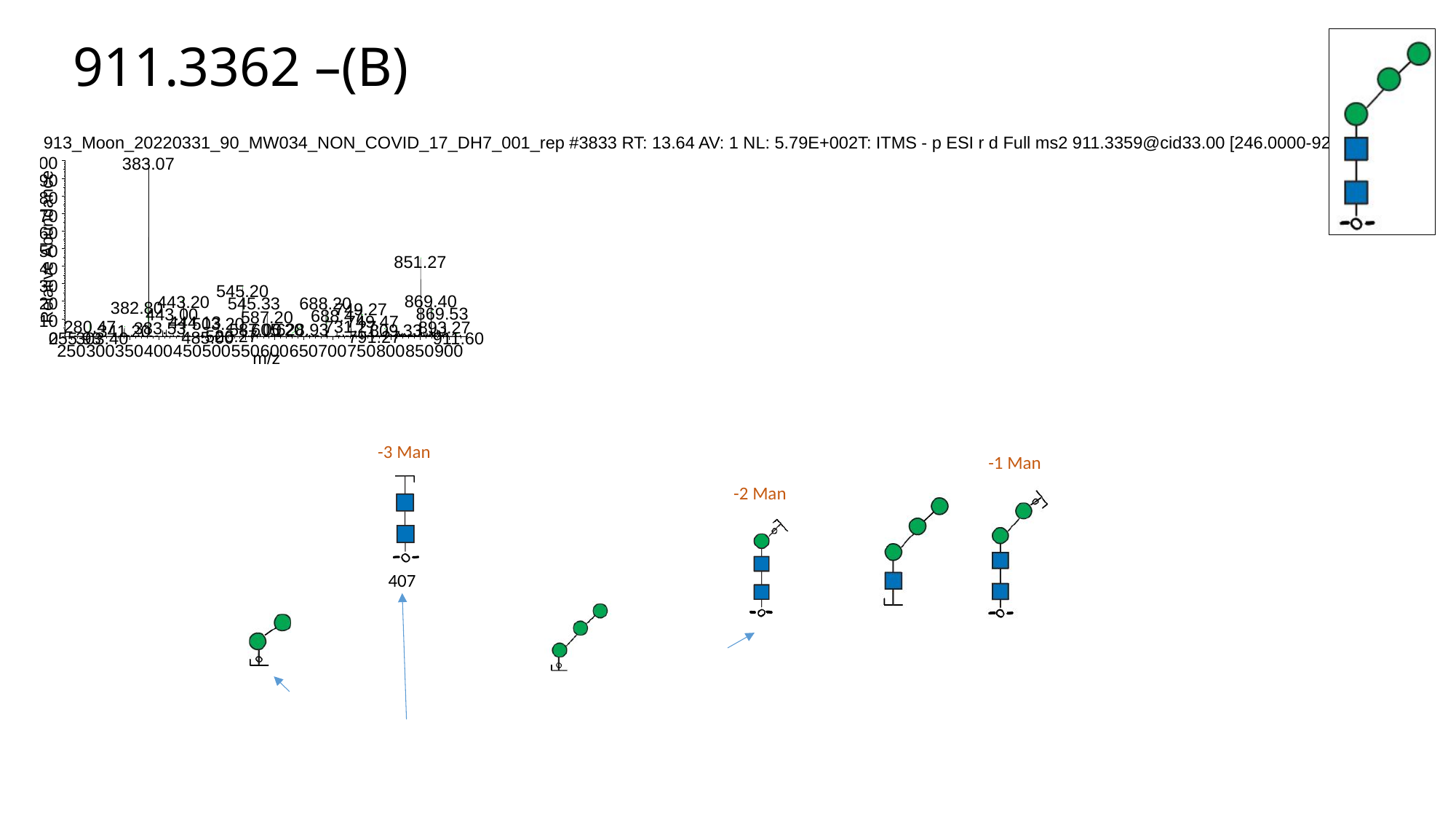

# 911.3362 –(B)
-3 Man
-1 Man
-2 Man
407

## Slide 11
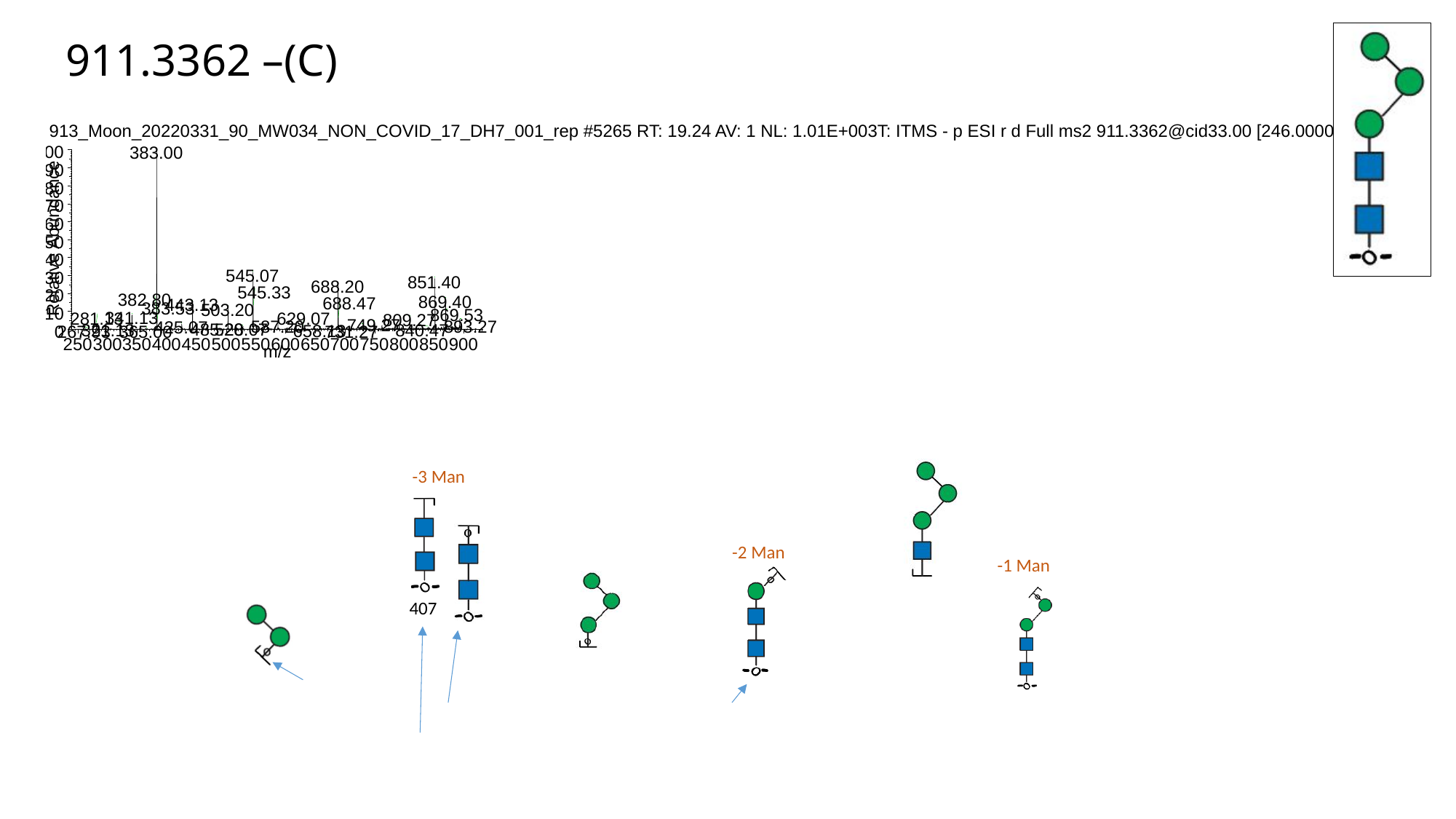

# 911.3362 –(C)
-3 Man
-2 Man
-1 Man
407

## Slide 12
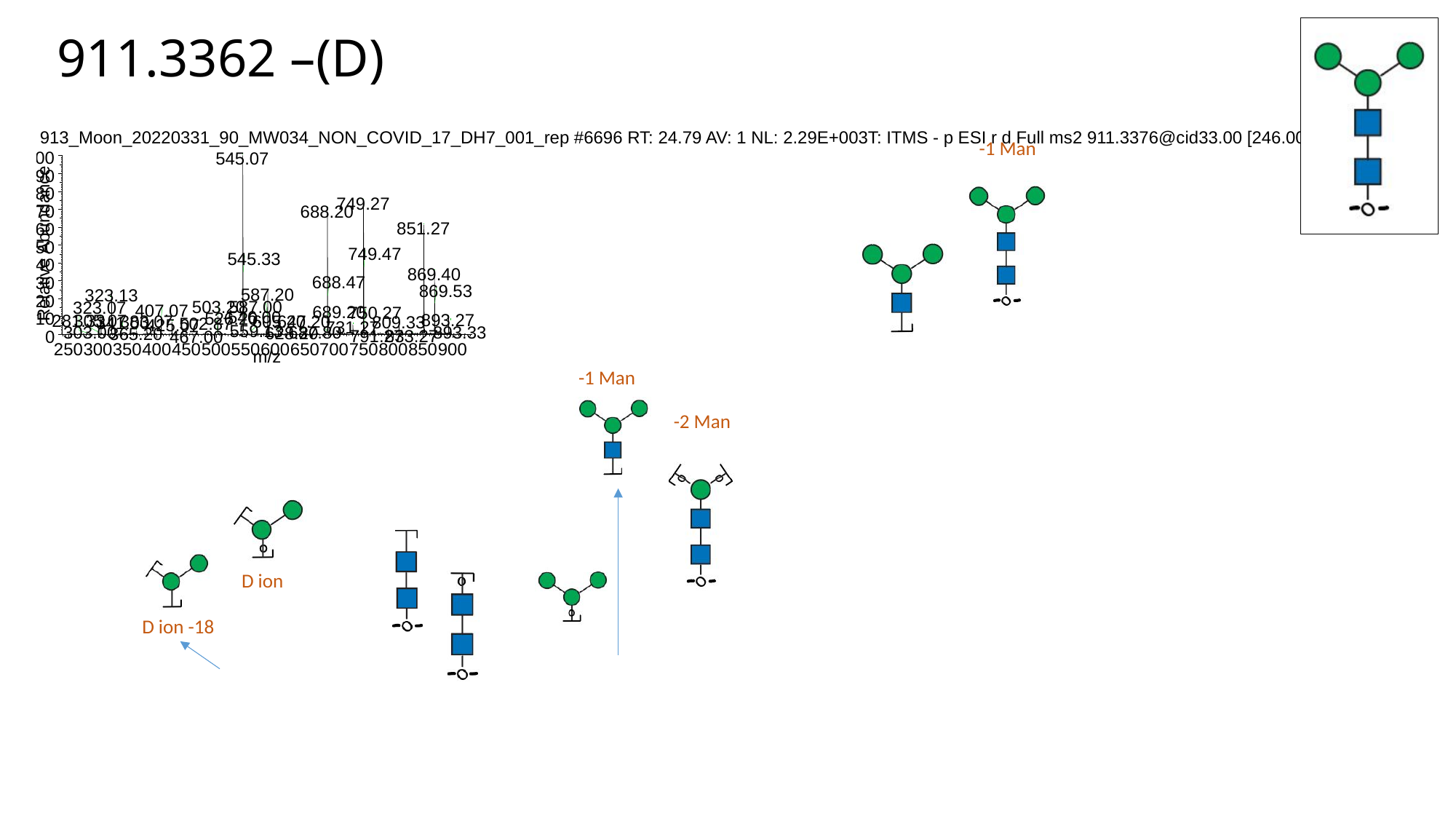

-1 Man
-1 Man
-2 Man
D ion
D ion -18
# 911.3362 –(D)

## Slide 13
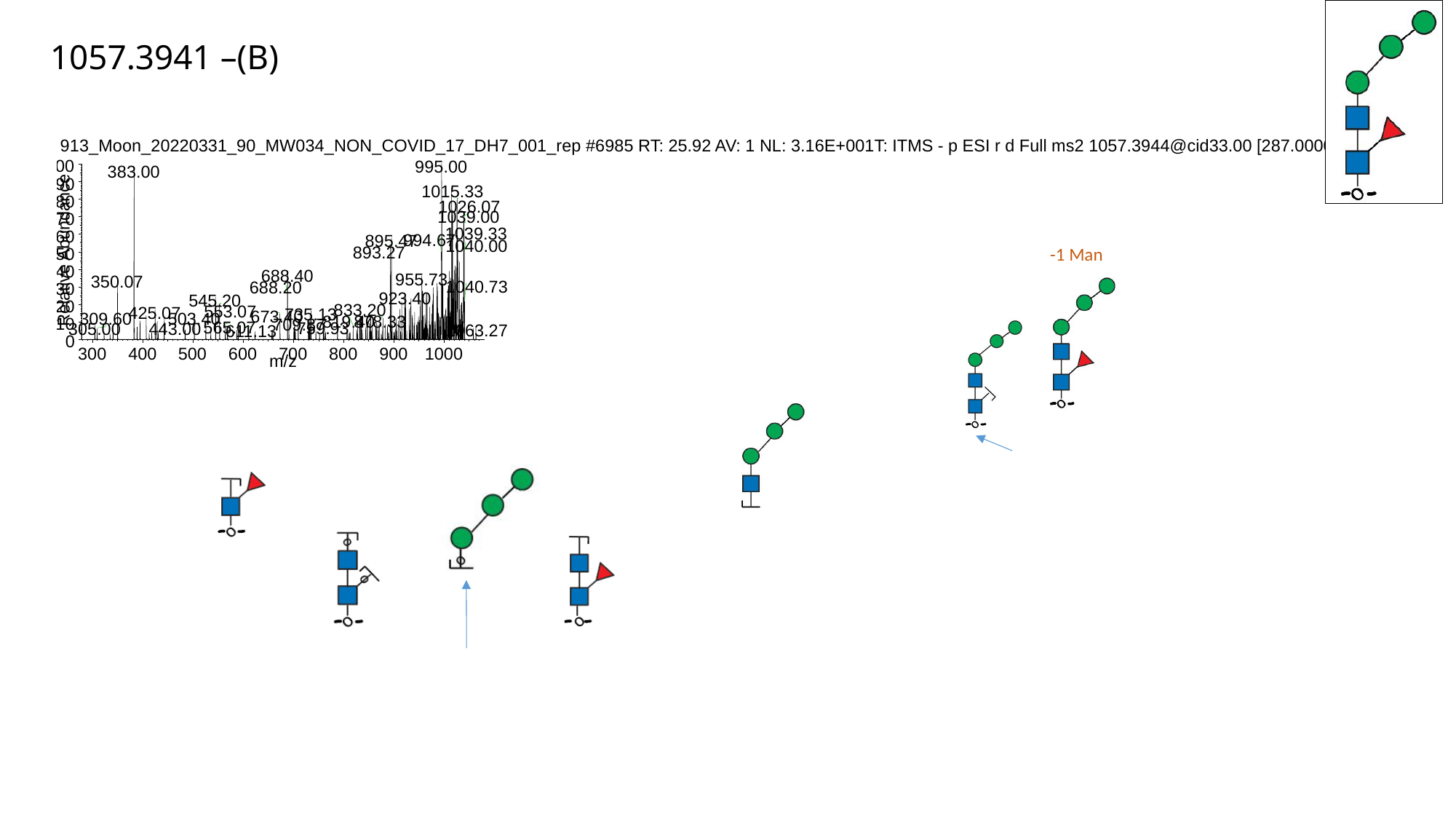

# 1057.3941 –(B)
-1 Man

## Slide 14
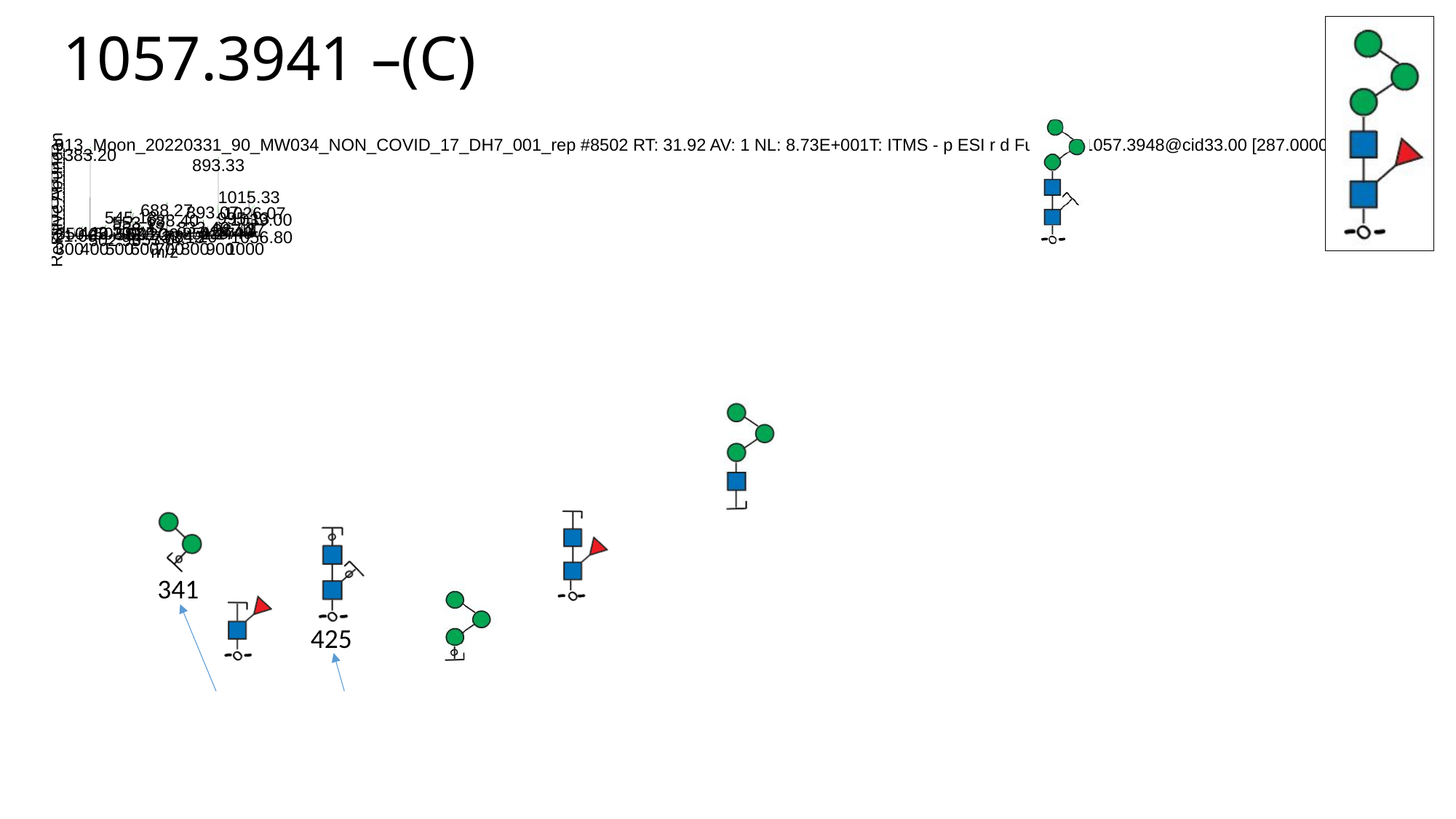

1057.3941 –(C)
341
425

## Slide 15
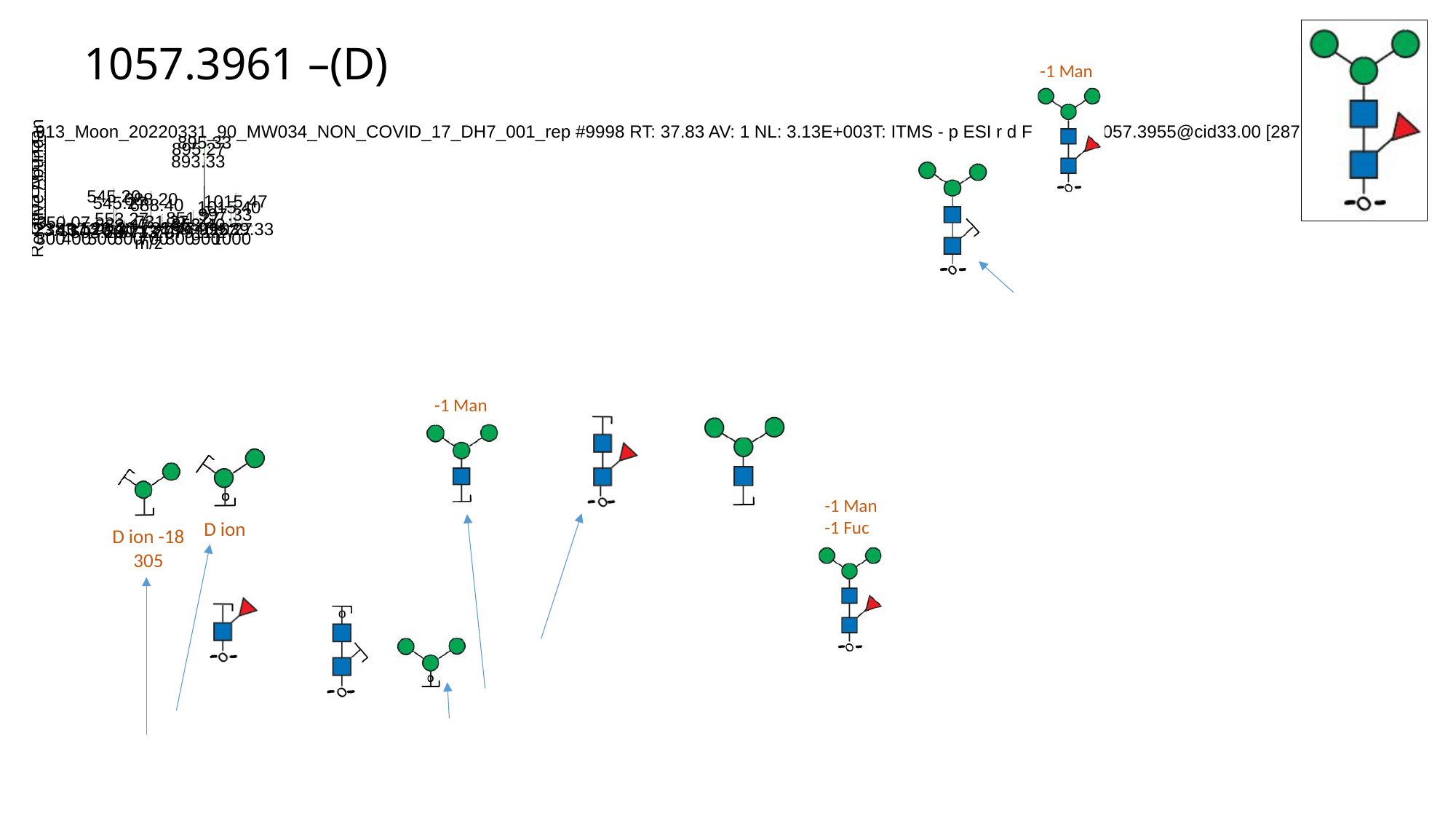

# 1057.3961 –(D)
-1 Man
-1 Man
D ion
-1 Man
-1 Fuc
D ion -18
305

## Slide 16
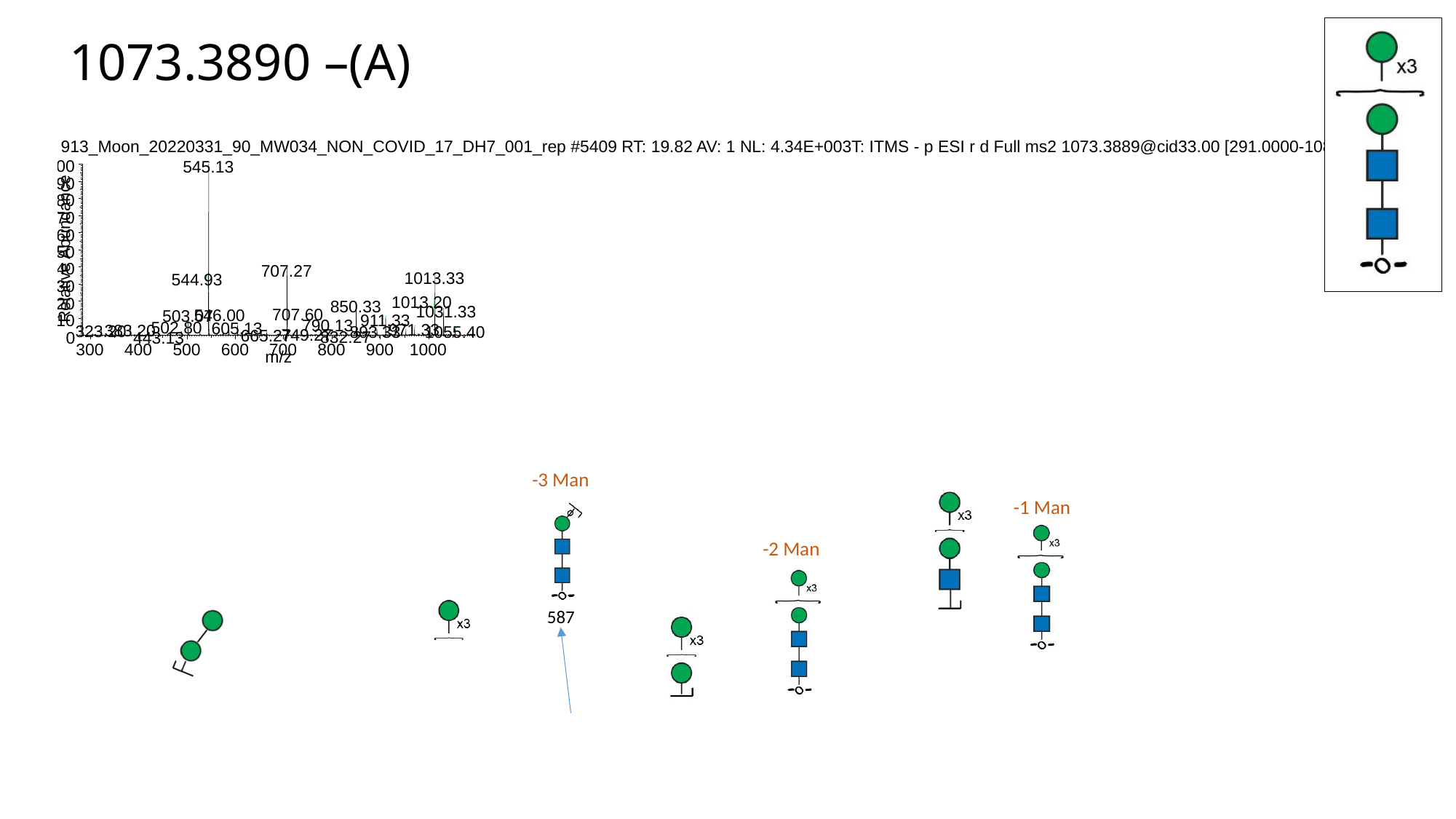

# 1073.3890 –(A)
-3 Man
-1 Man
-2 Man
587

## Slide 17
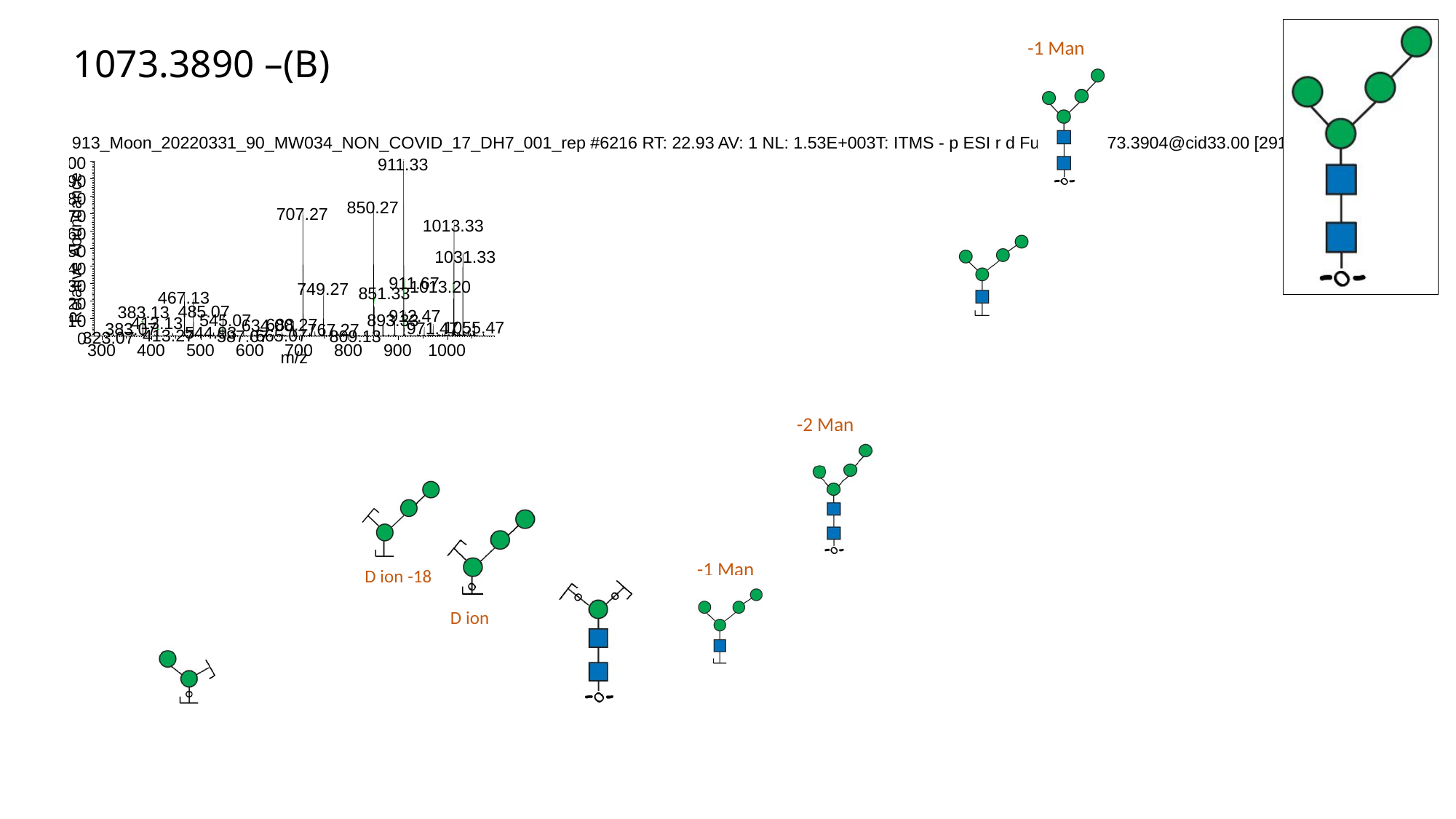

-1 Man
# 1073.3890 –(B)
-2 Man
-1 Man
D ion -18
D ion

## Slide 18
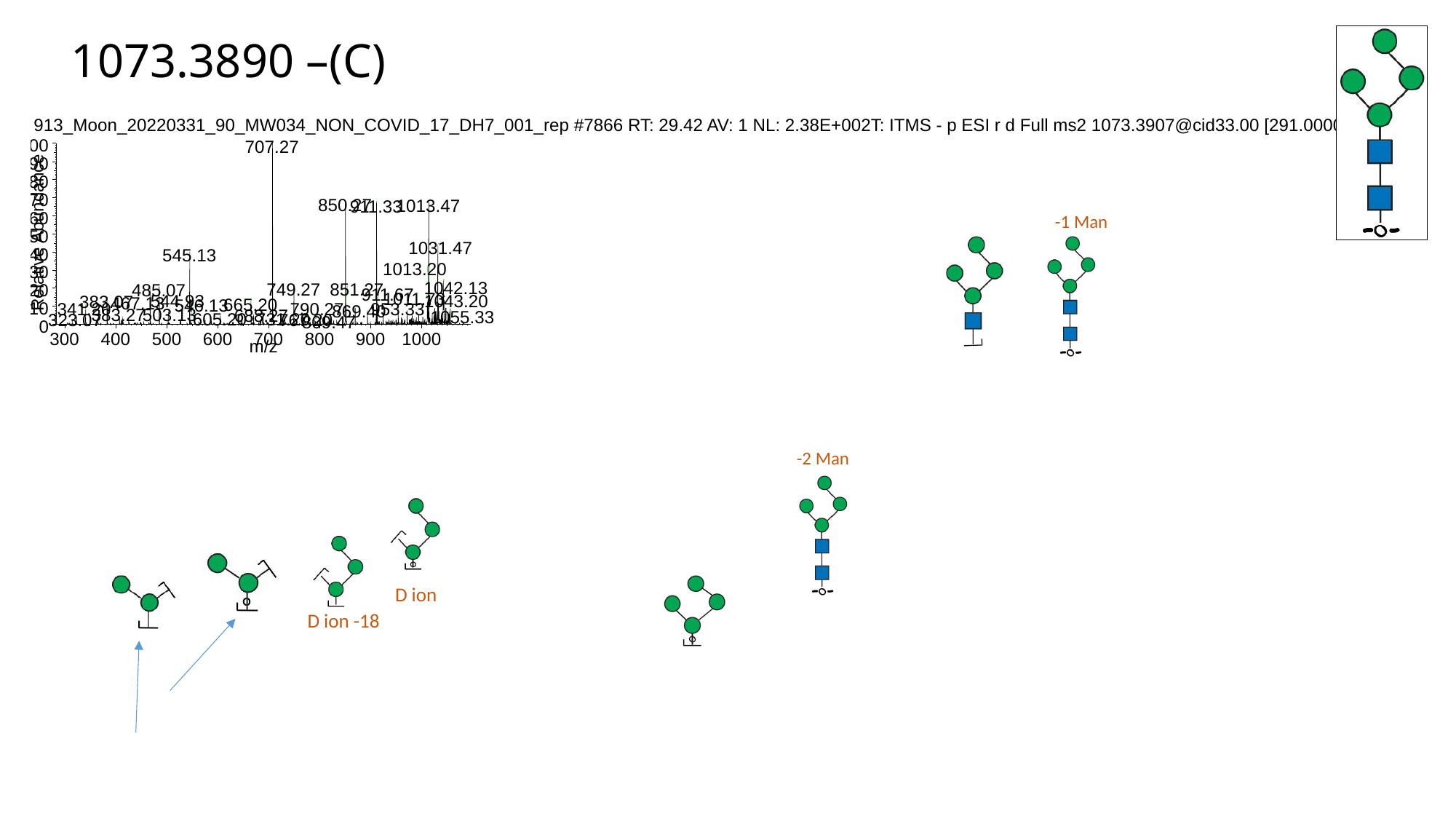

# 1073.3890 –(C)
-1 Man
-2 Man
D ion
D ion -18

## Slide 19
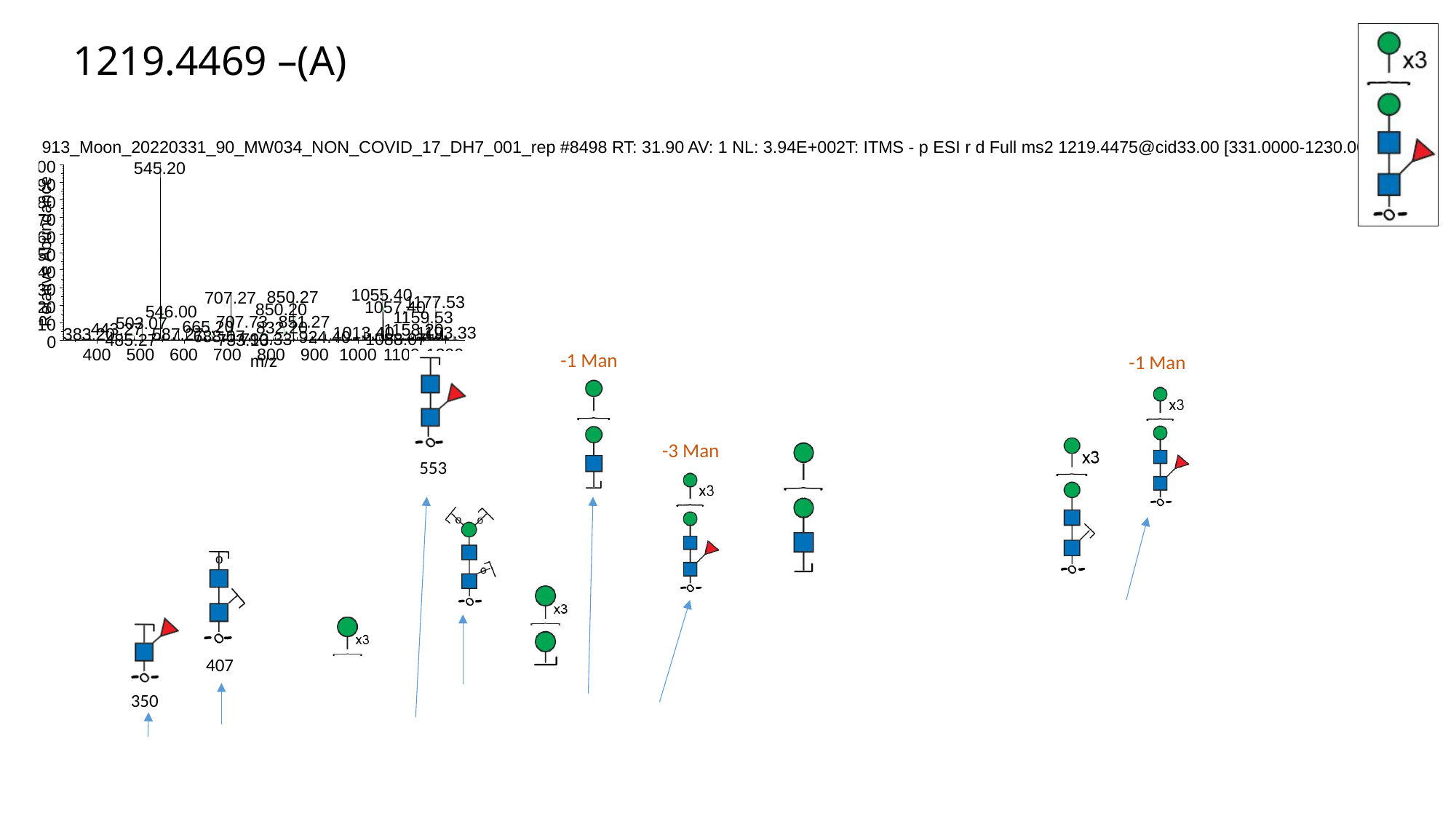

# 1219.4469 –(A)
-1 Man
-1 Man
-3 Man
553
407
350

## Slide 20
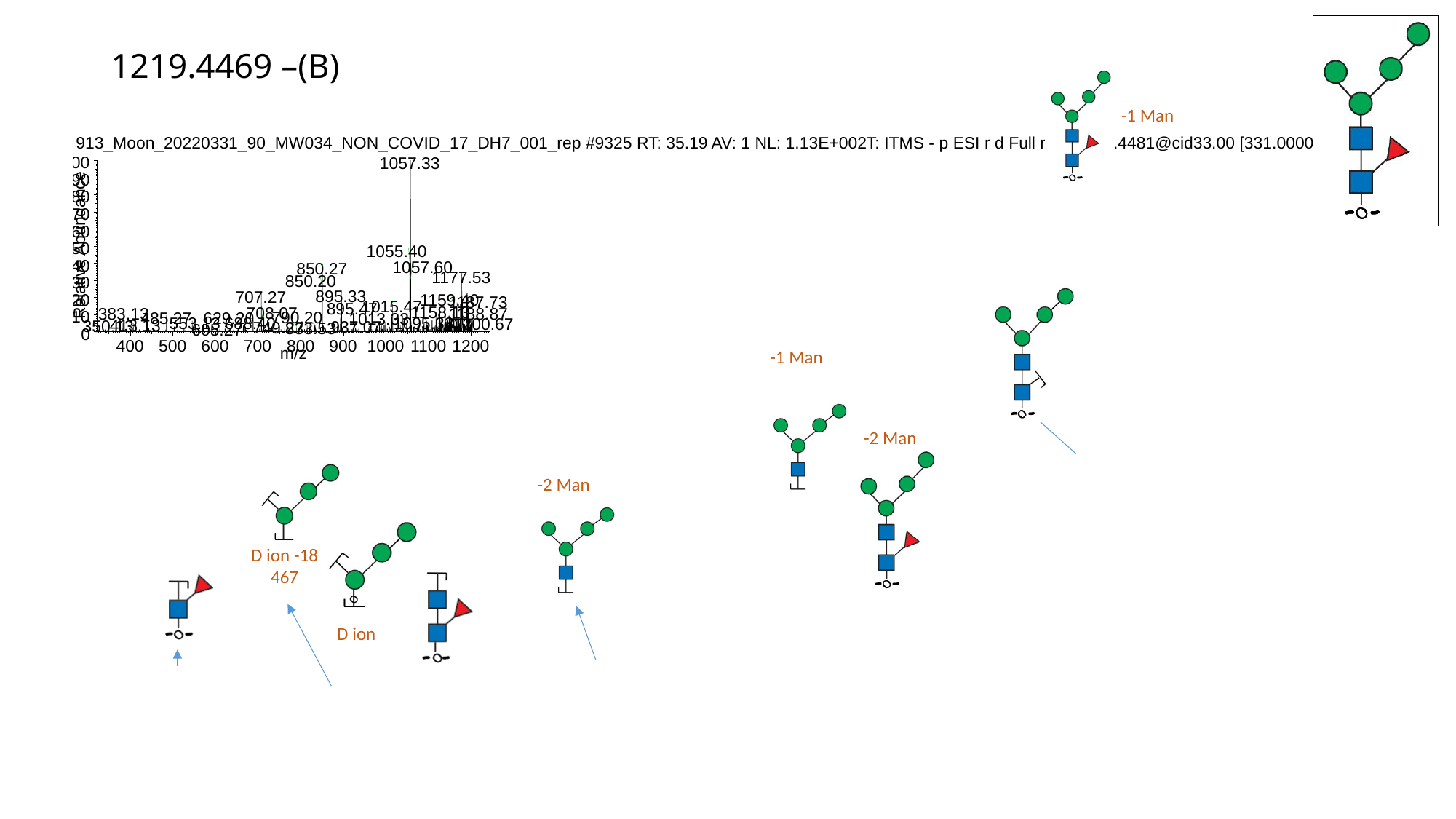

# 1219.4469 –(B)
-1 Man
-1 Man
-2 Man
-2 Man
D ion -18
467
D ion

## Slide 21
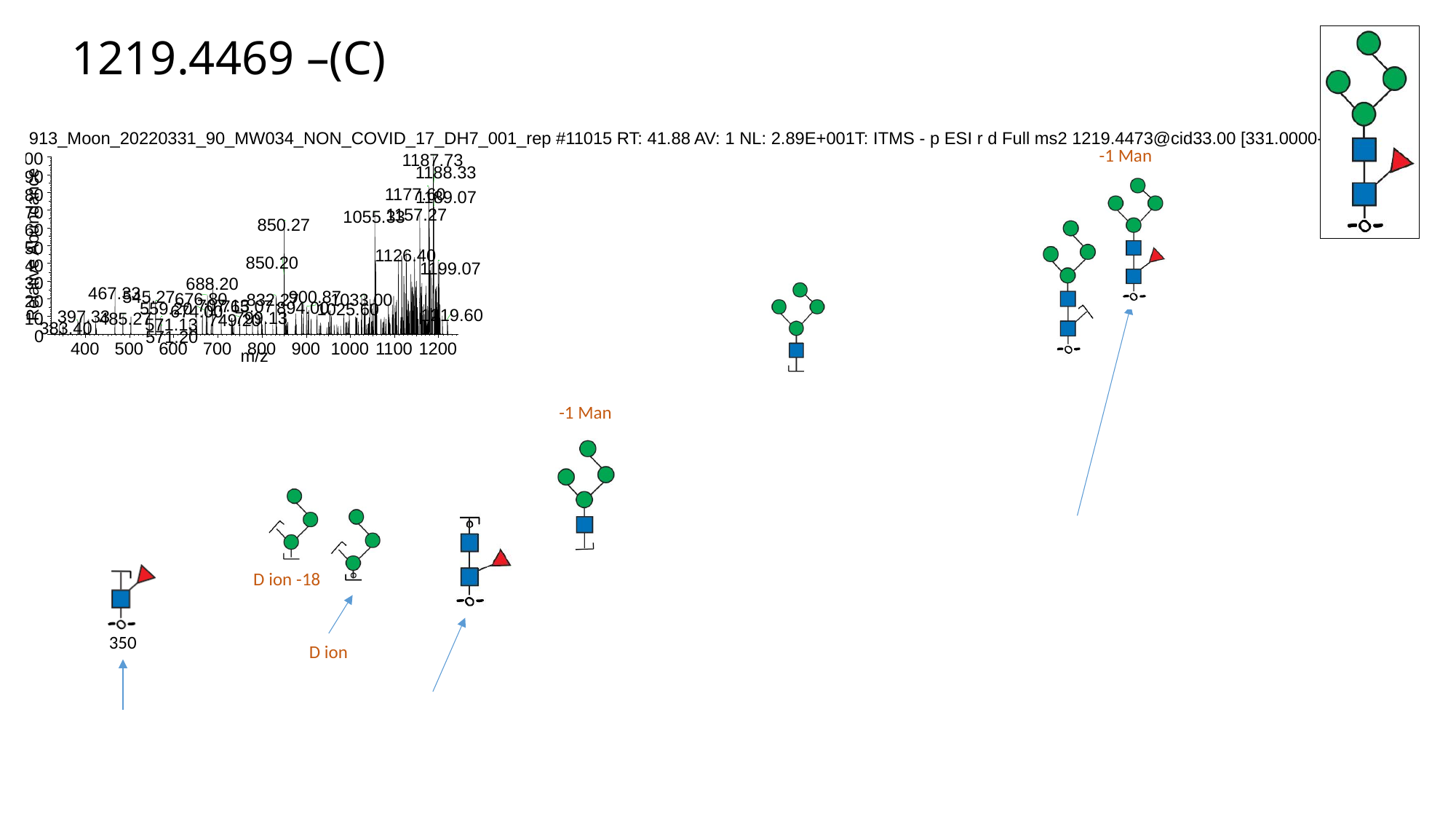

# 1219.4469 –(C)
-1 Man
-1 Man
D ion -18
350
D ion

## Slide 22
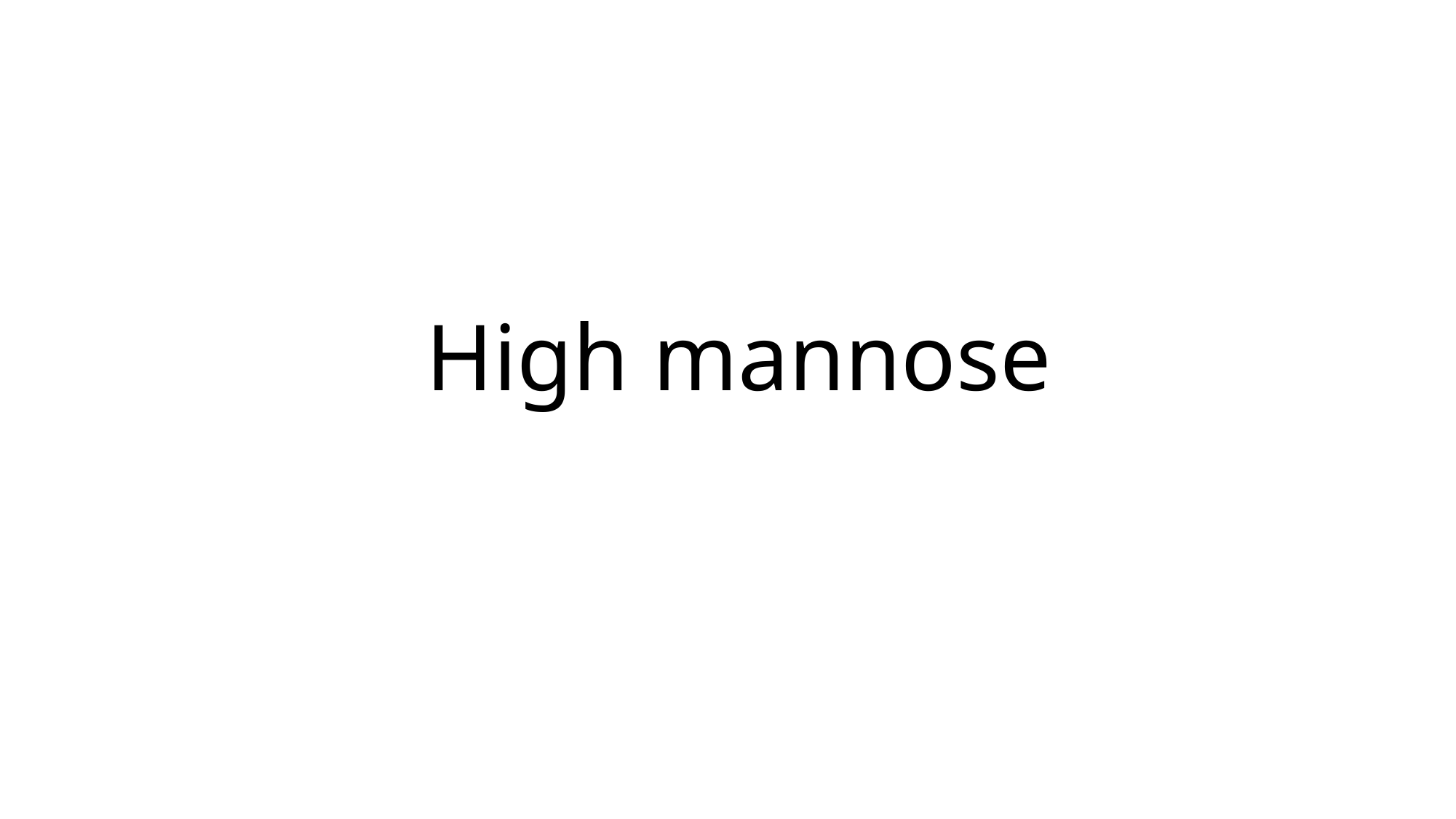

# High mannose

## Slide 23
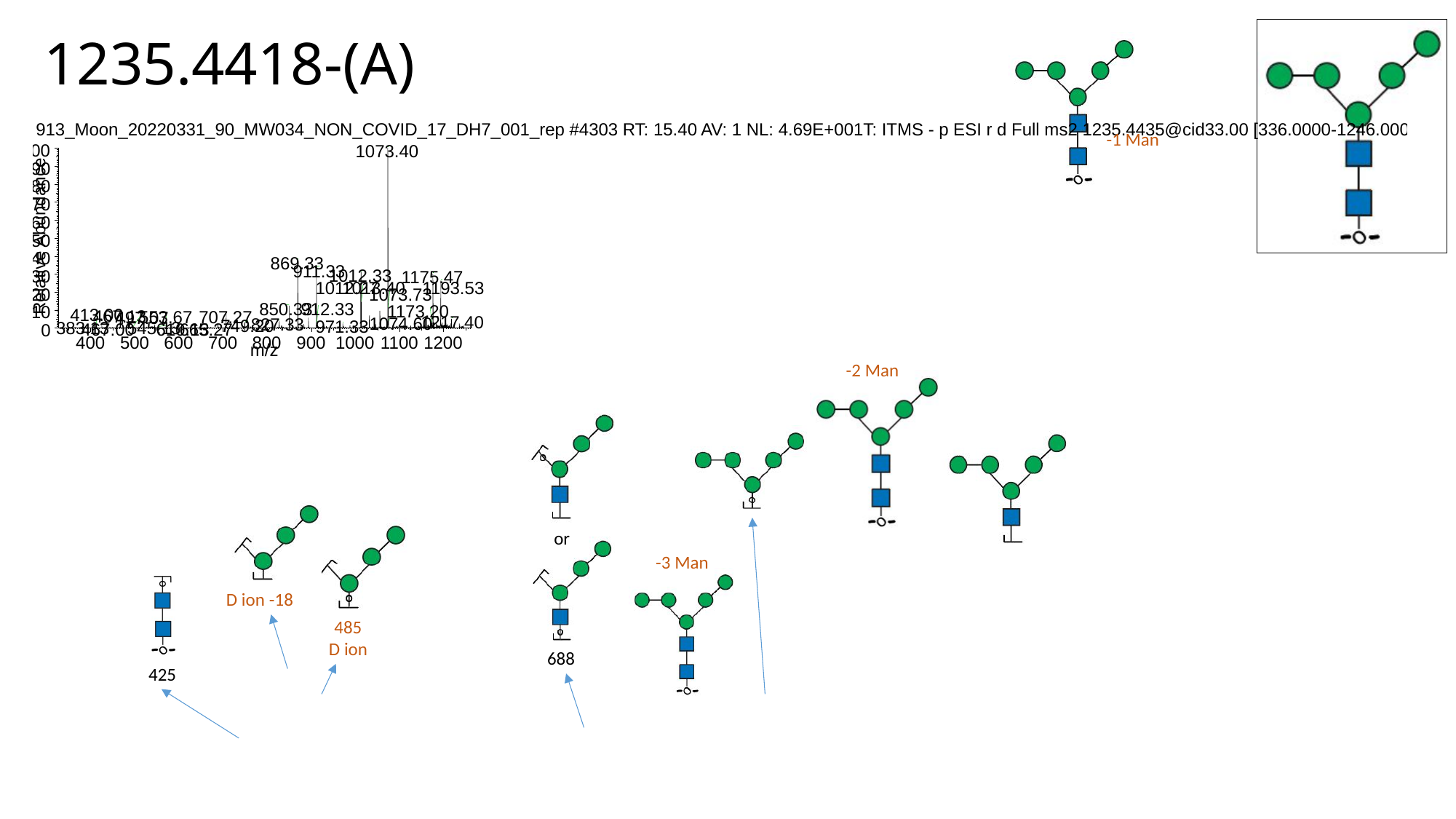

-1 Man
-2 Man
or
D ion -18
485
D ion
-3 Man
688
# 1235.4418-(A)
425

## Slide 24
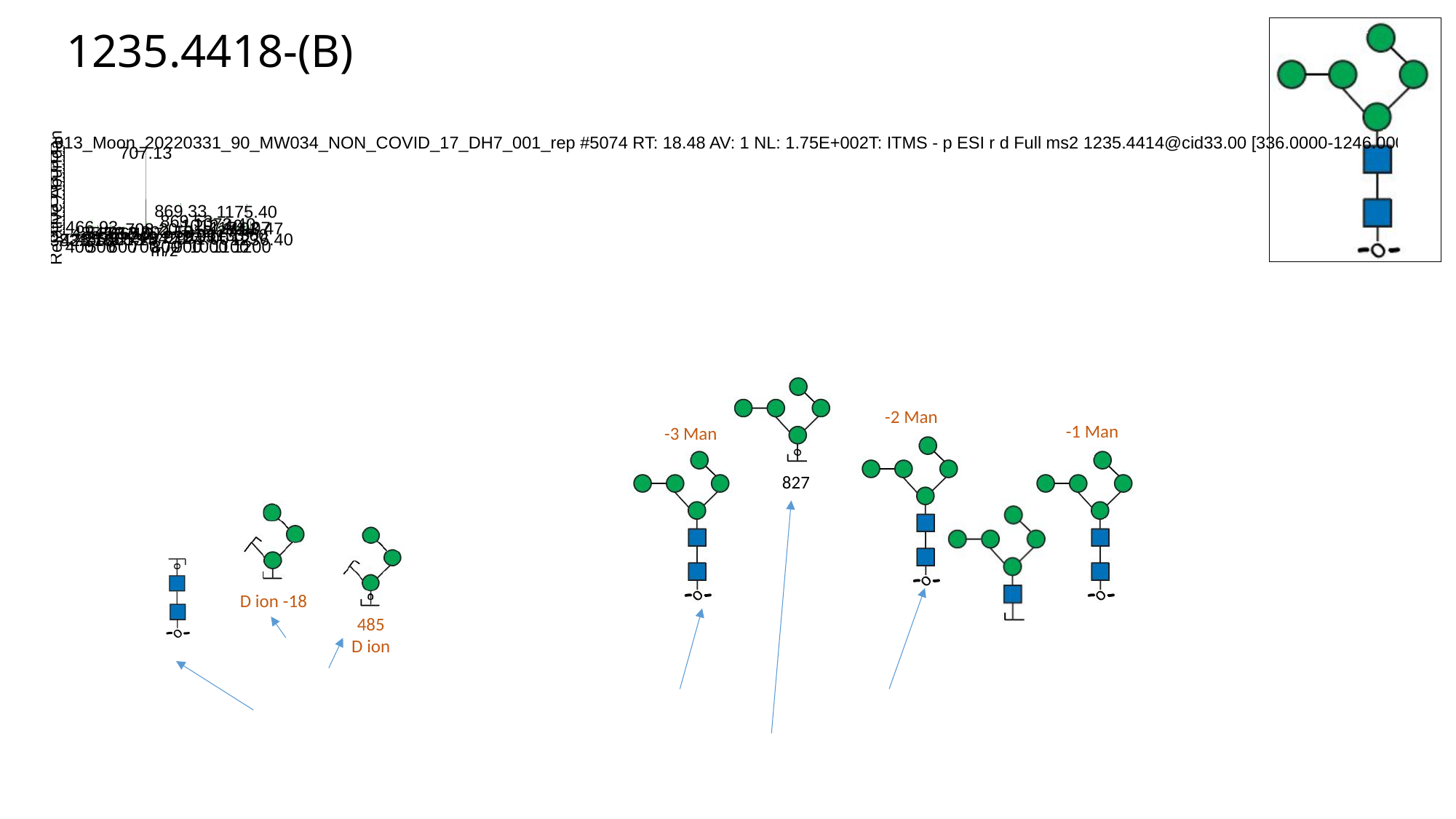

-2 Man
-1 Man
-3 Man
D ion -18
485
D ion
# 1235.4418-(B)
827

## Slide 25
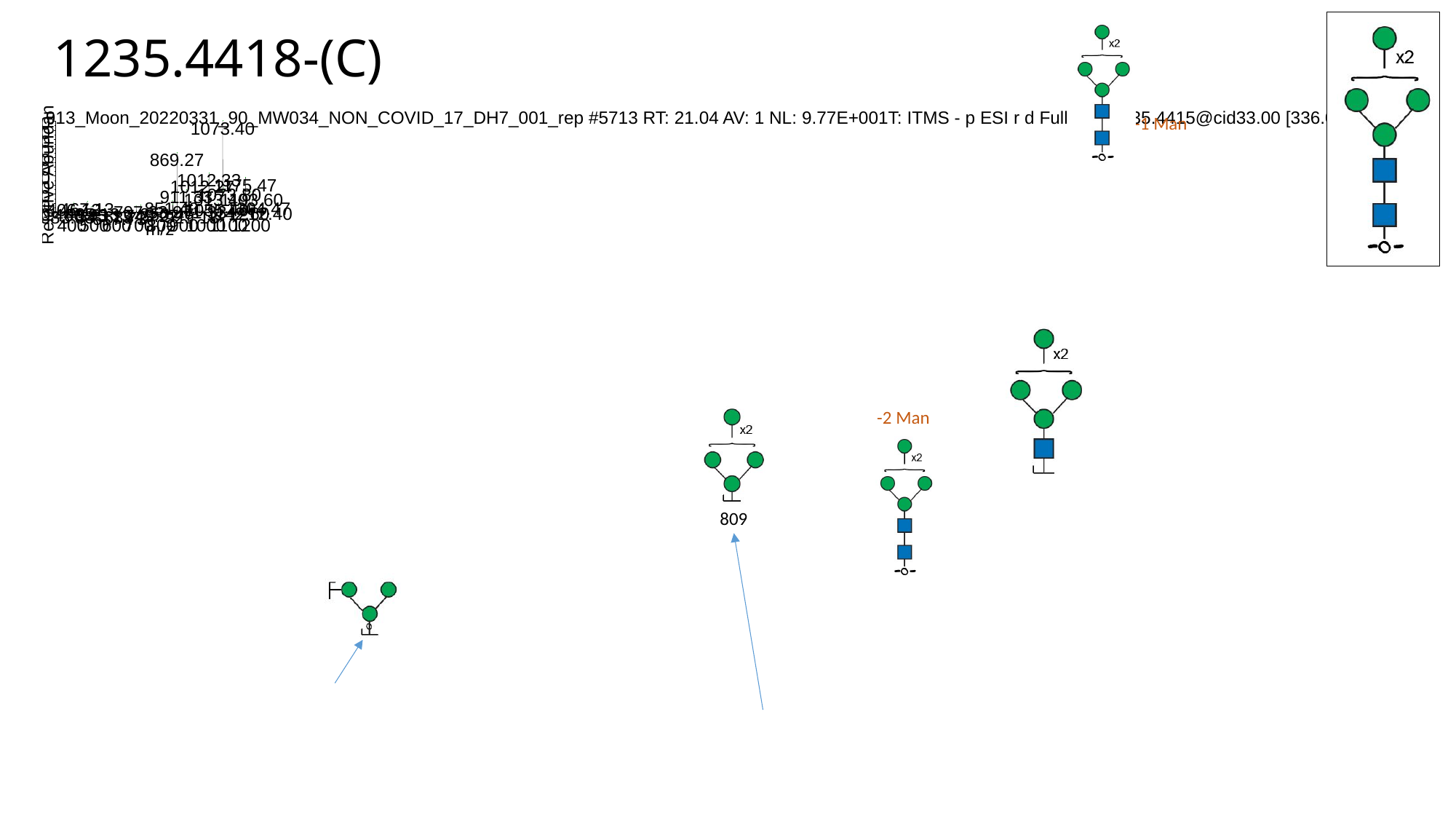

# 1235.4418-(C)
-1 Man
-2 Man
809

## Slide 26
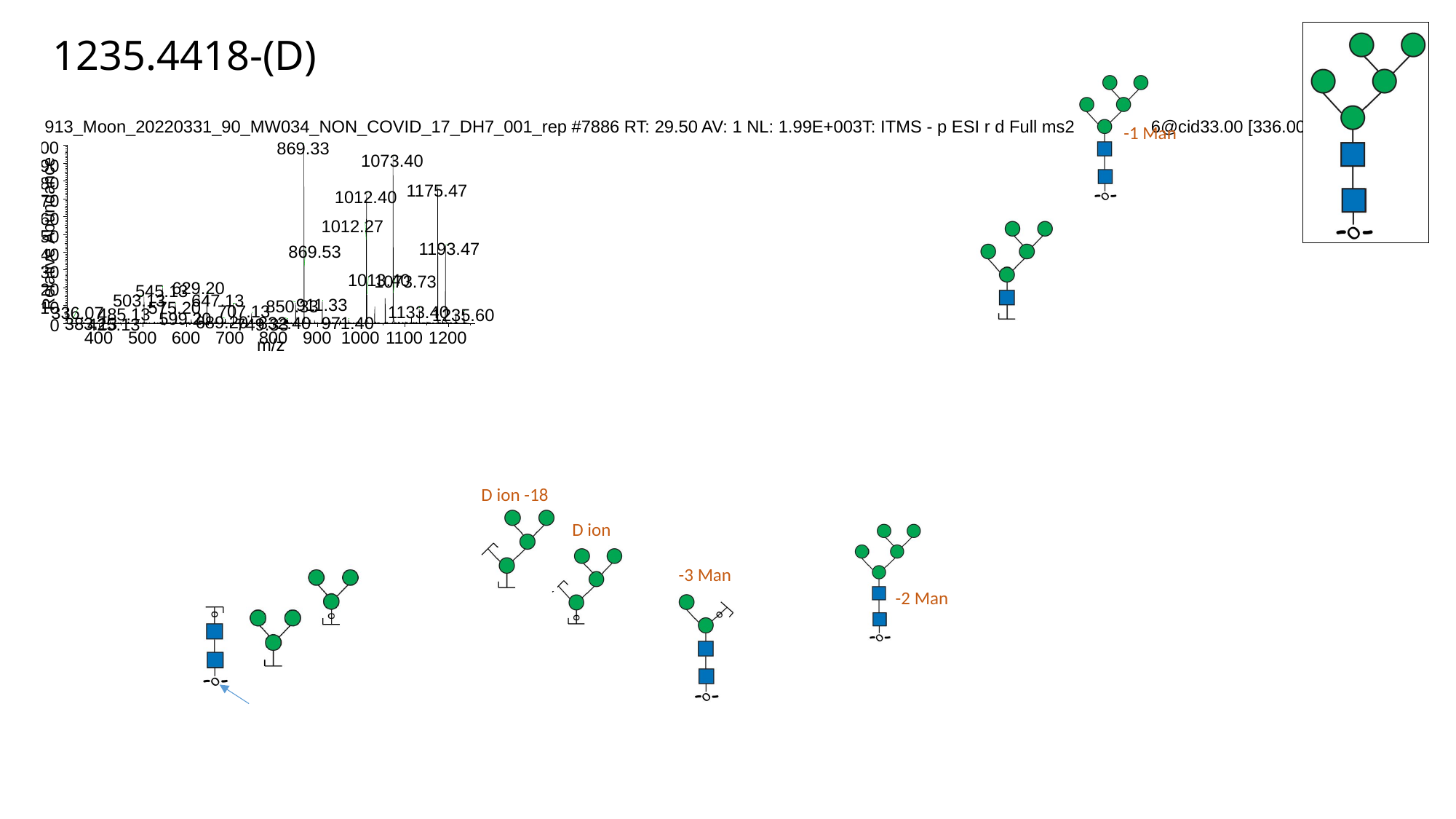

-1 Man
D ion -18
D ion
-3 Man
-2 Man
# 1235.4418-(D)

## Slide 27
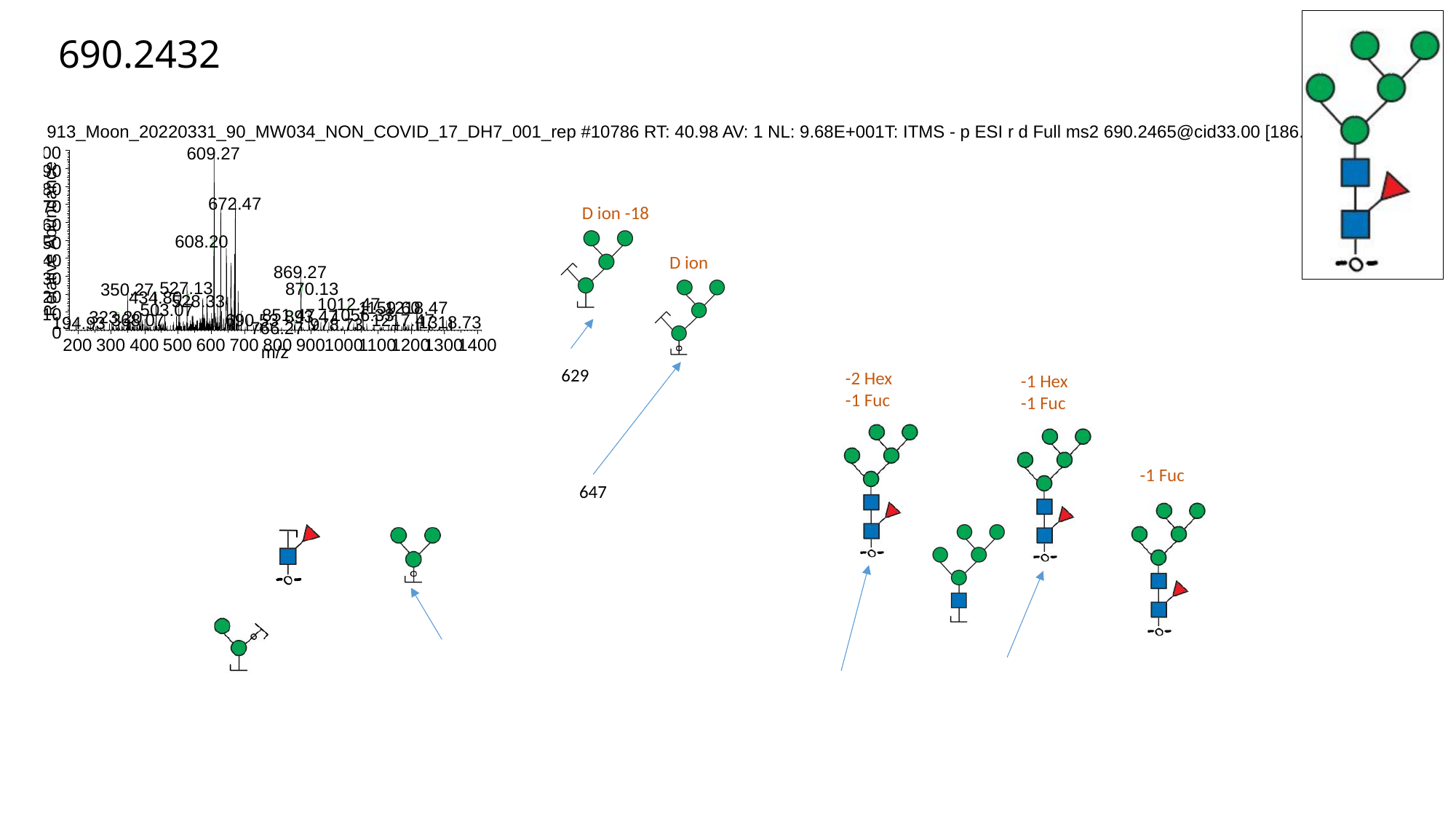

D ion -18
D ion
629
-1 Hex
-1 Fuc
-1 Fuc
647
# 690.2432
-2 Hex
-1 Fuc

## Slide 28
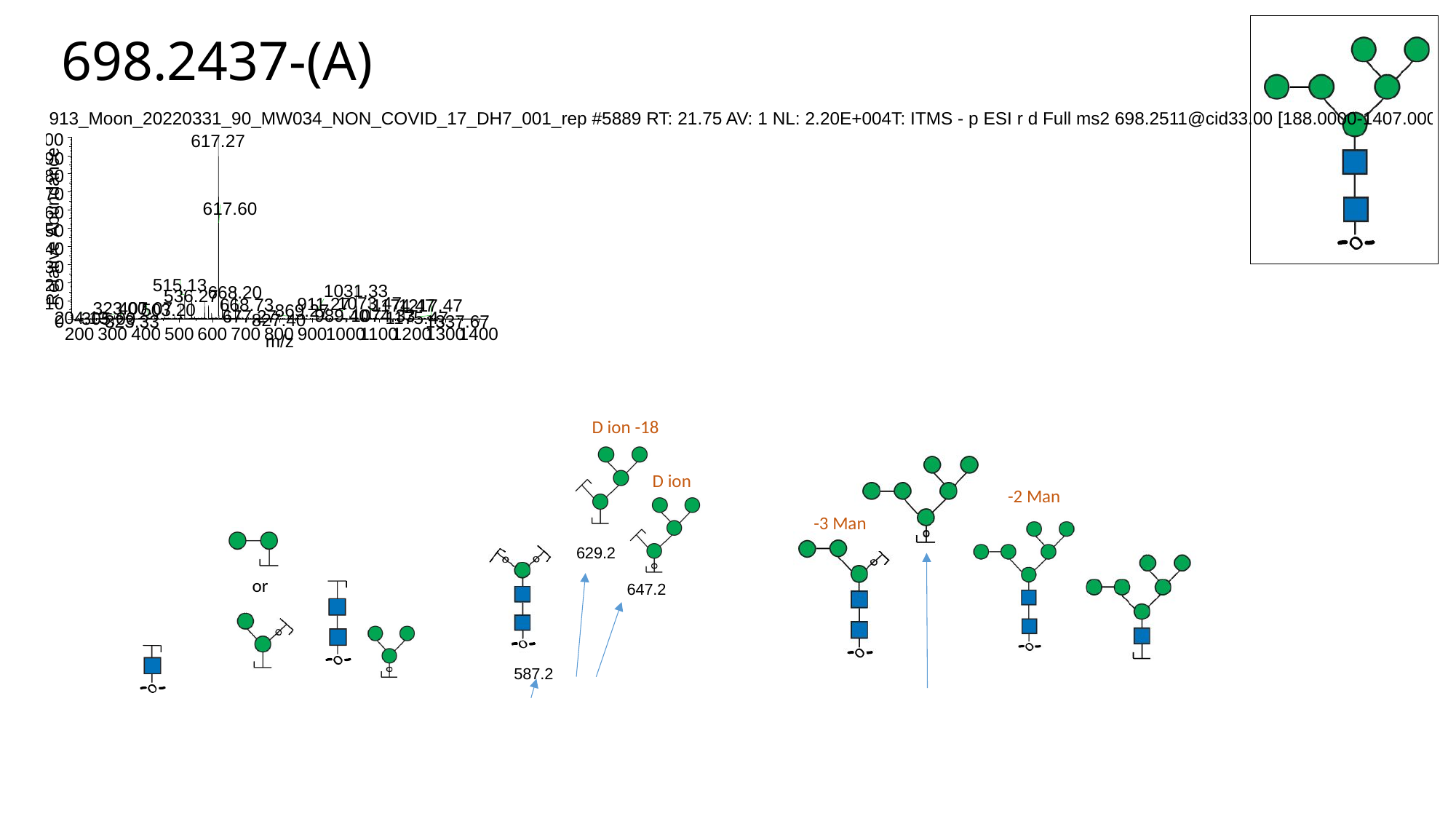

D ion -18
D ion
-2 Man
-3 Man
or
629.2
647.2
587.2
# 698.2437-(A)

## Slide 29
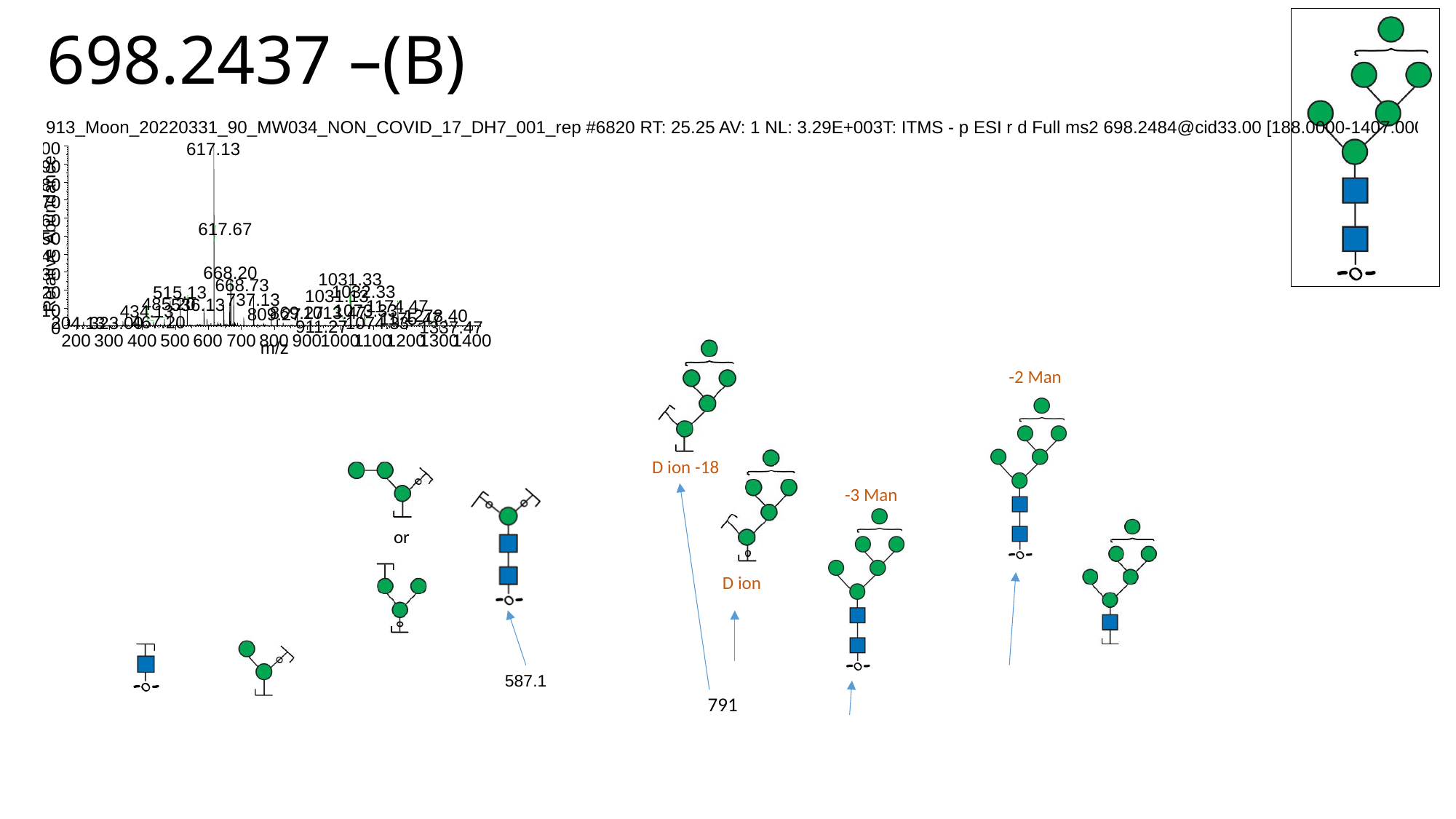

# 698.2437 –(B)
-2 Man
D ion -18
or
-3 Man
D ion
587.1
791

## Slide 30
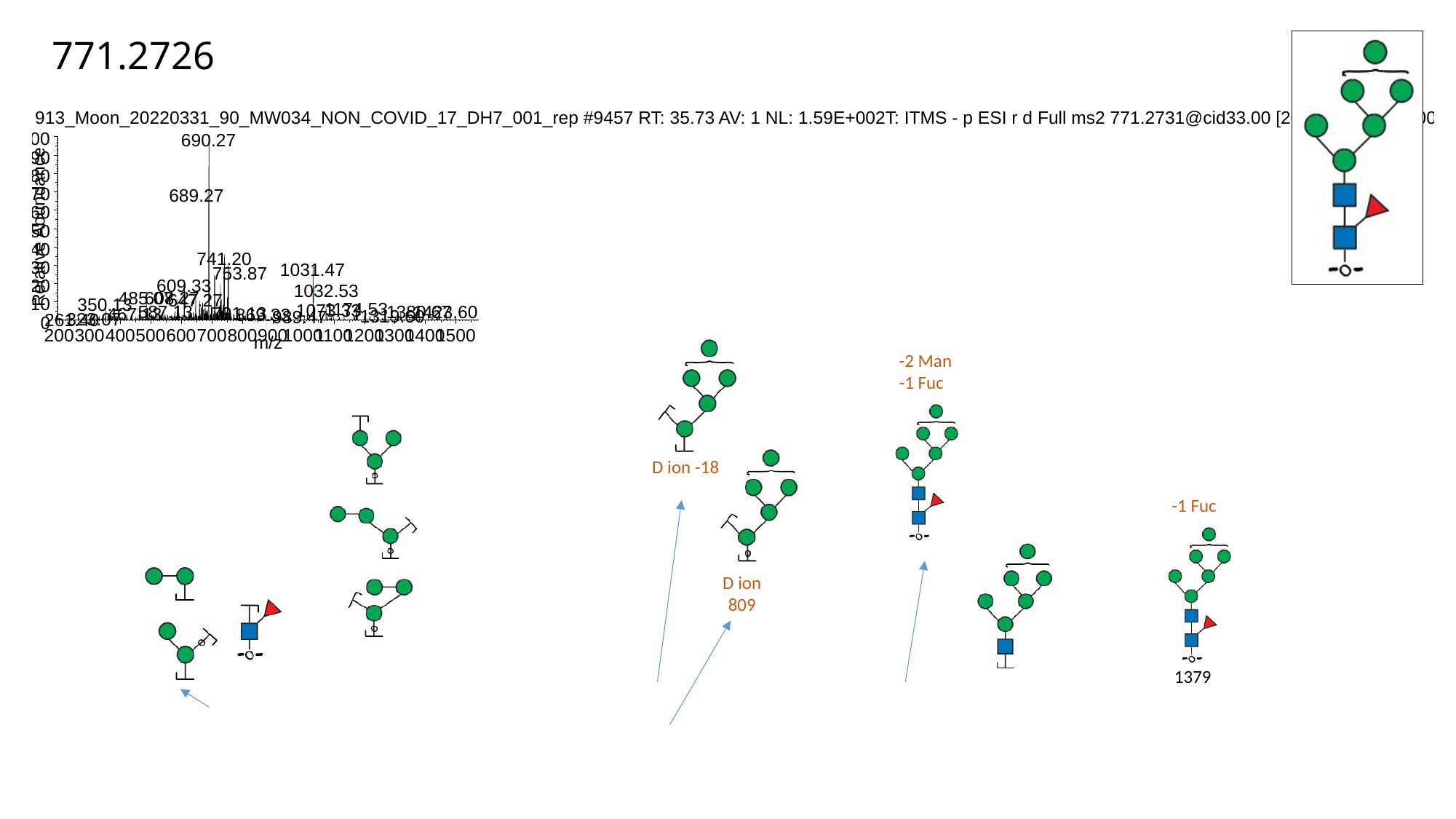

# 771.2726
-2 Man
-1 Fuc
D ion -18
-1 Fuc
D ion
809
1379

## Slide 31
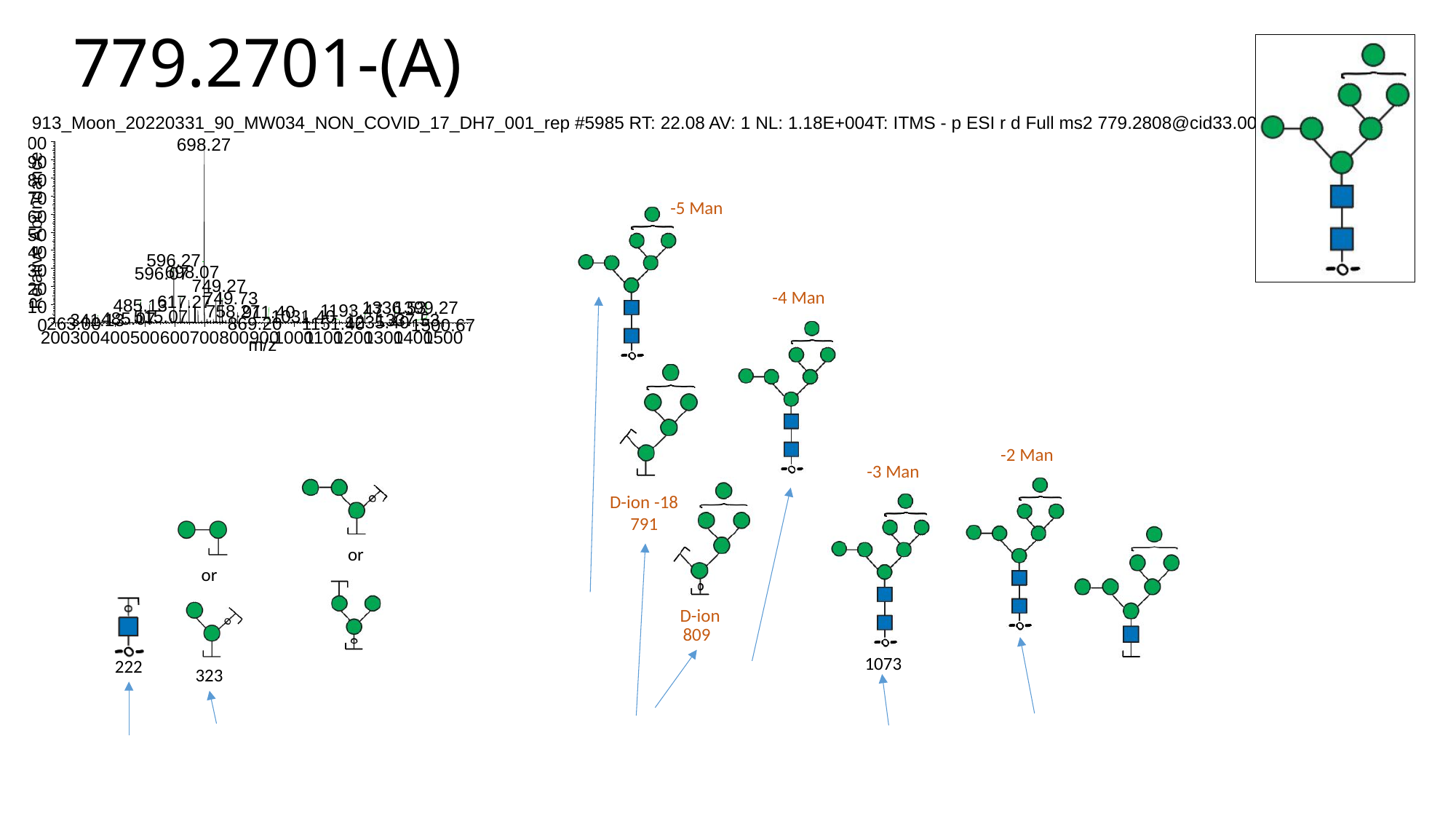

# 779.2701-(A)
-5 Man
-4 Man
-2 Man
-3 Man
or
D-ion -18
791
or
D-ion
809
1073
222
323

## Slide 32
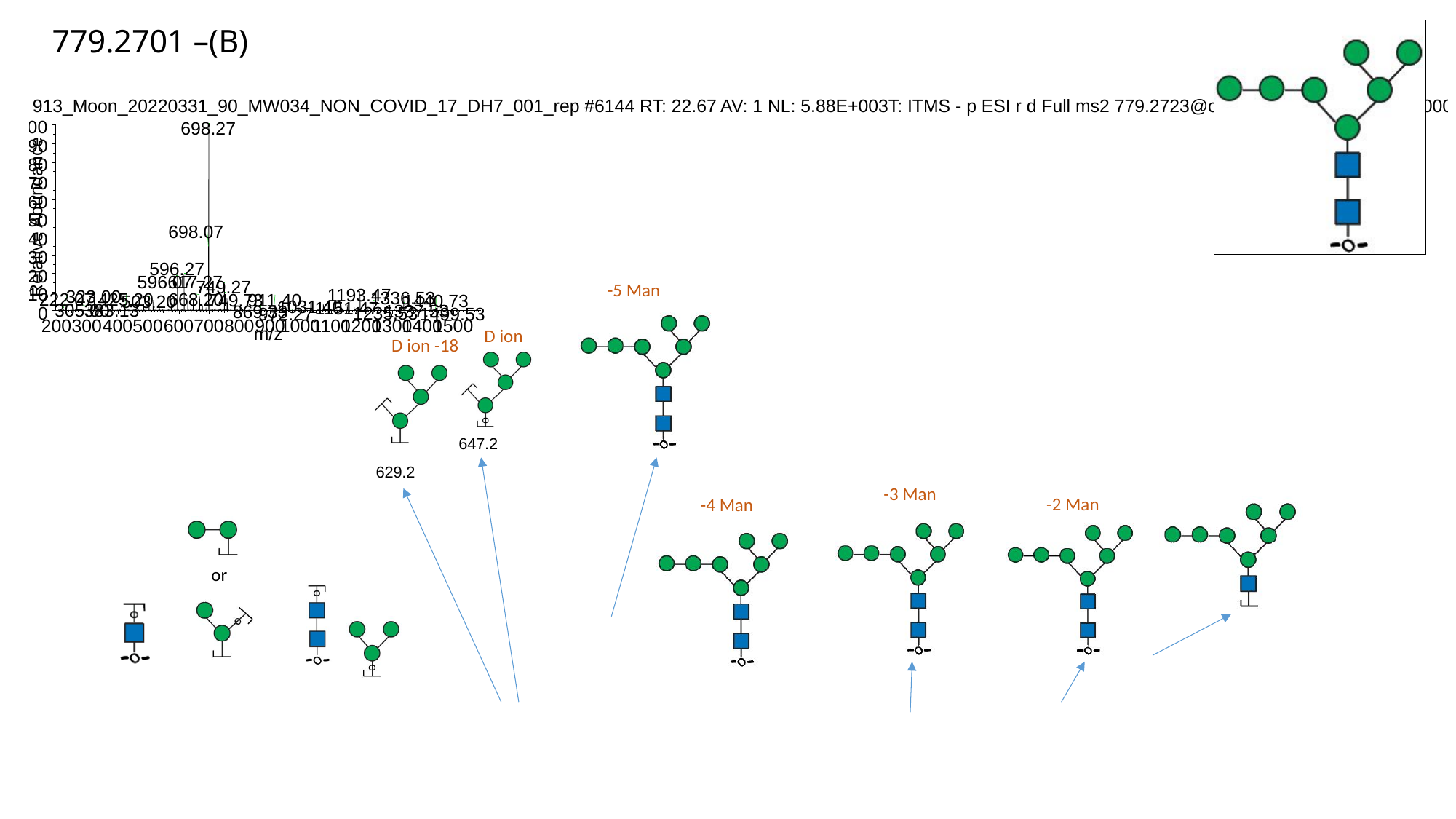

# 779.2701 –(B)
-5 Man
D ion
D ion -18
647.2
629.2
-3 Man
-2 Man
-4 Man
or

## Slide 33
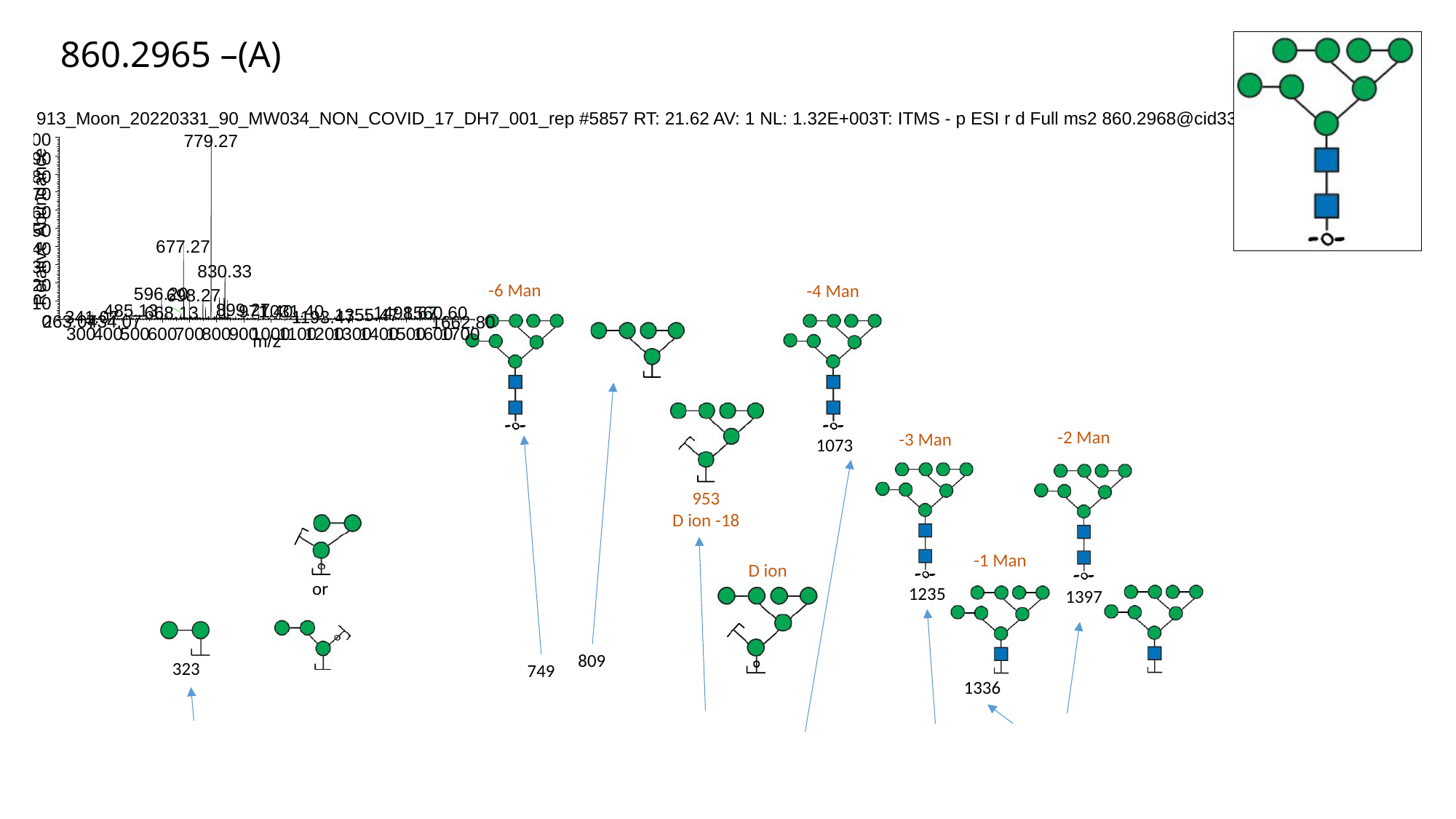

# 860.2965 –(A)
-6 Man
-4 Man
-2 Man
-3 Man
1073
953
D ion -18
-1 Man
D ion
or
1235
1397
809
323
749
1336

## Slide 34
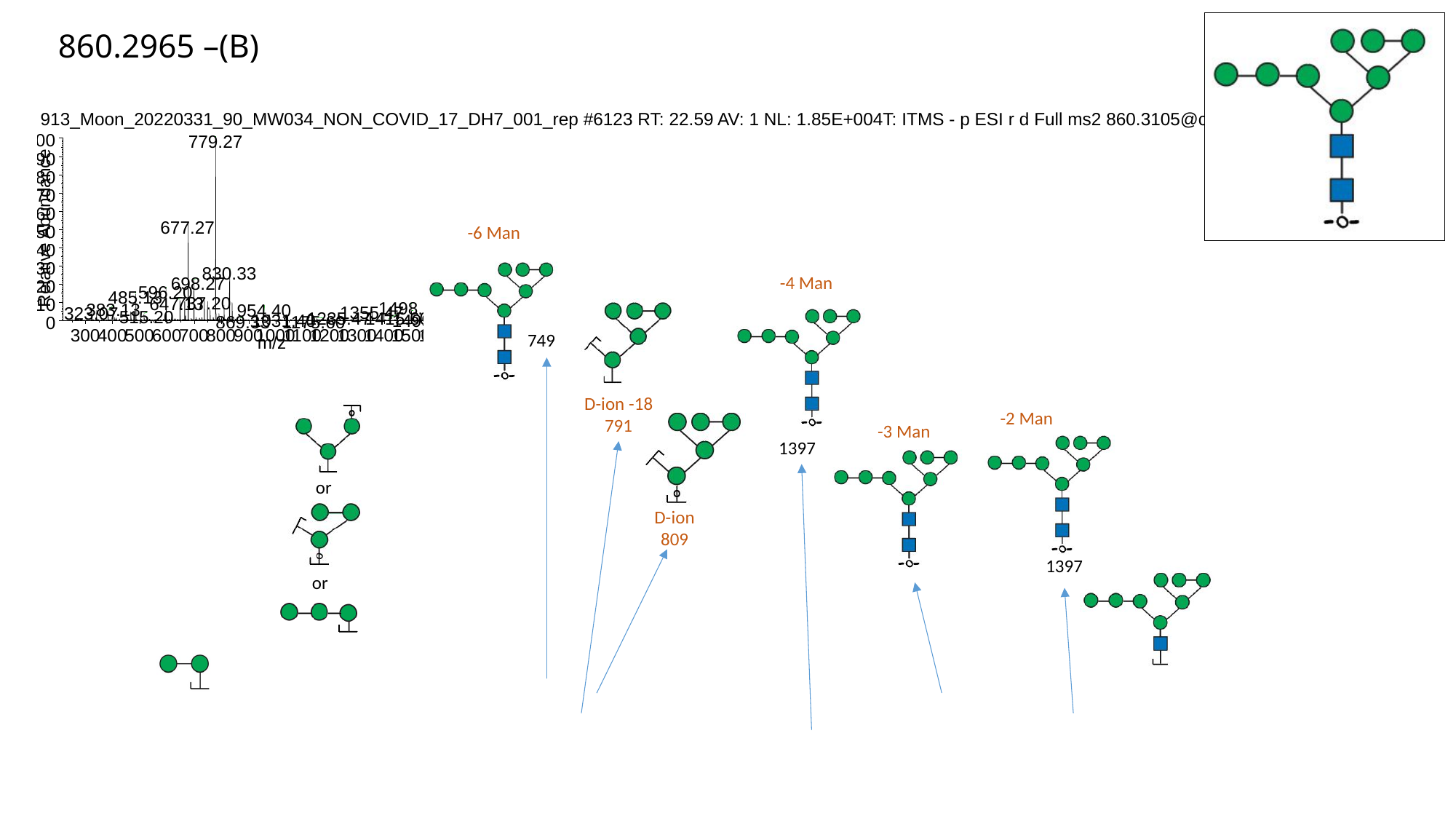

# 860.2965 –(B)
-6 Man
-4 Man
749
D-ion -18
791
or
or
-2 Man
-3 Man
1397
D-ion
809
1397

## Slide 35
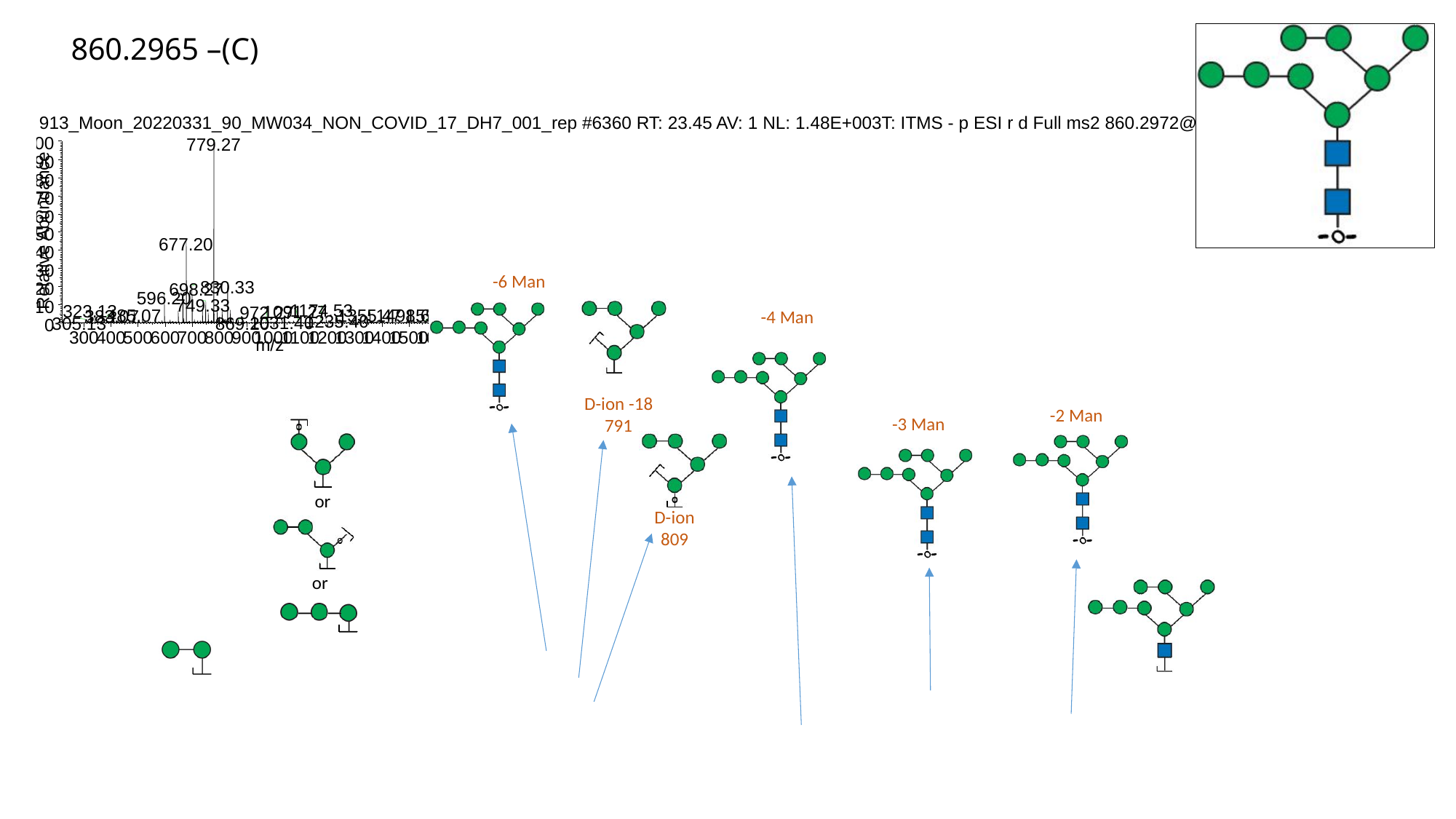

# 860.2965 –(C)
-6 Man
-4 Man
D-ion -18
791
-2 Man
-3 Man
or
or
D-ion
809

## Slide 36
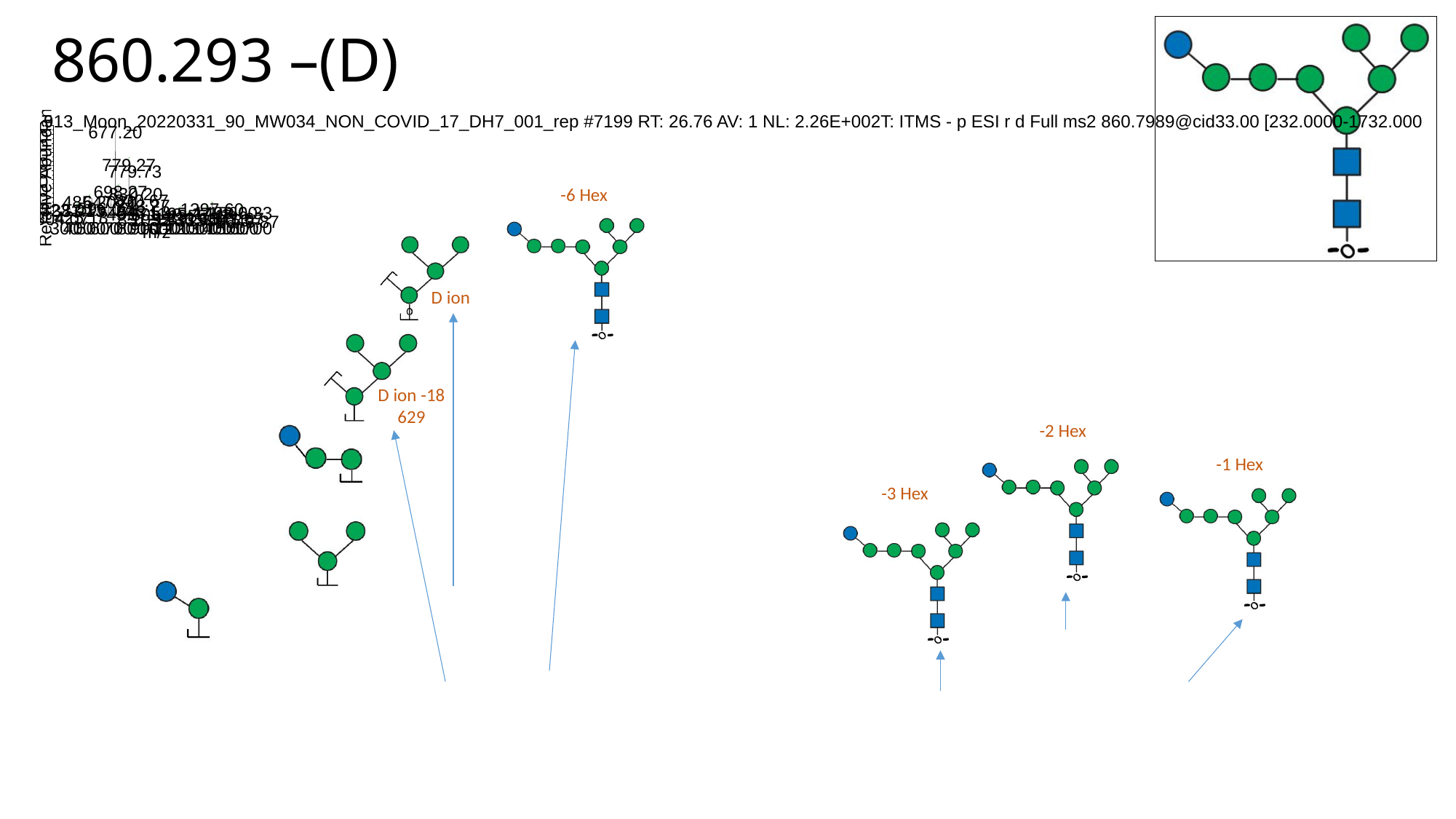

D ion
D ion -18
629
-2 Hex
-1 Hex
-3 Hex
# 860.293 –(D)
-6 Hex

## Slide 37
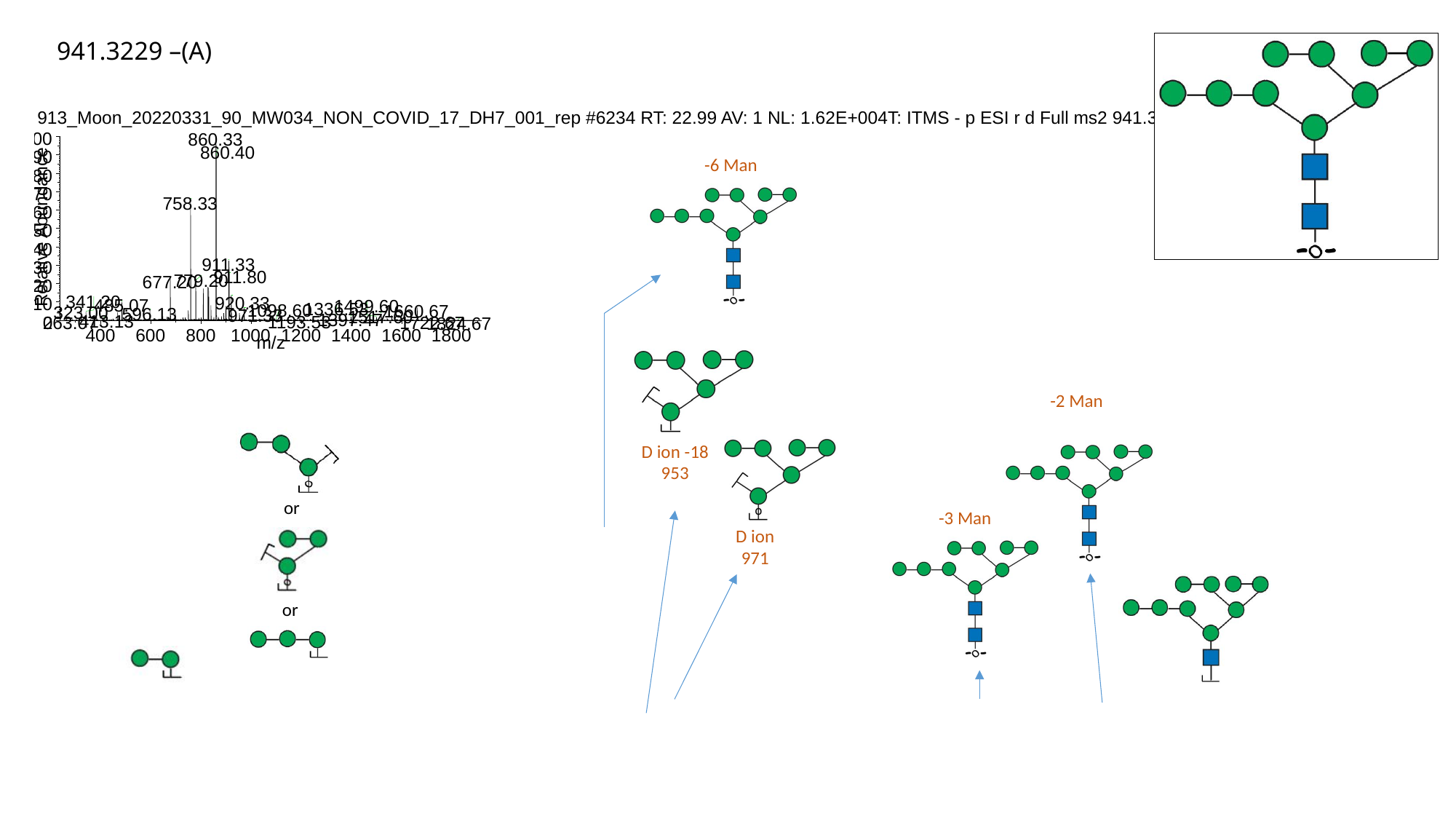

# 941.3229 –(A)
-6 Man
-2 Man
D ion -18
953
or
-3 Man
D ion
971
or

## Slide 38
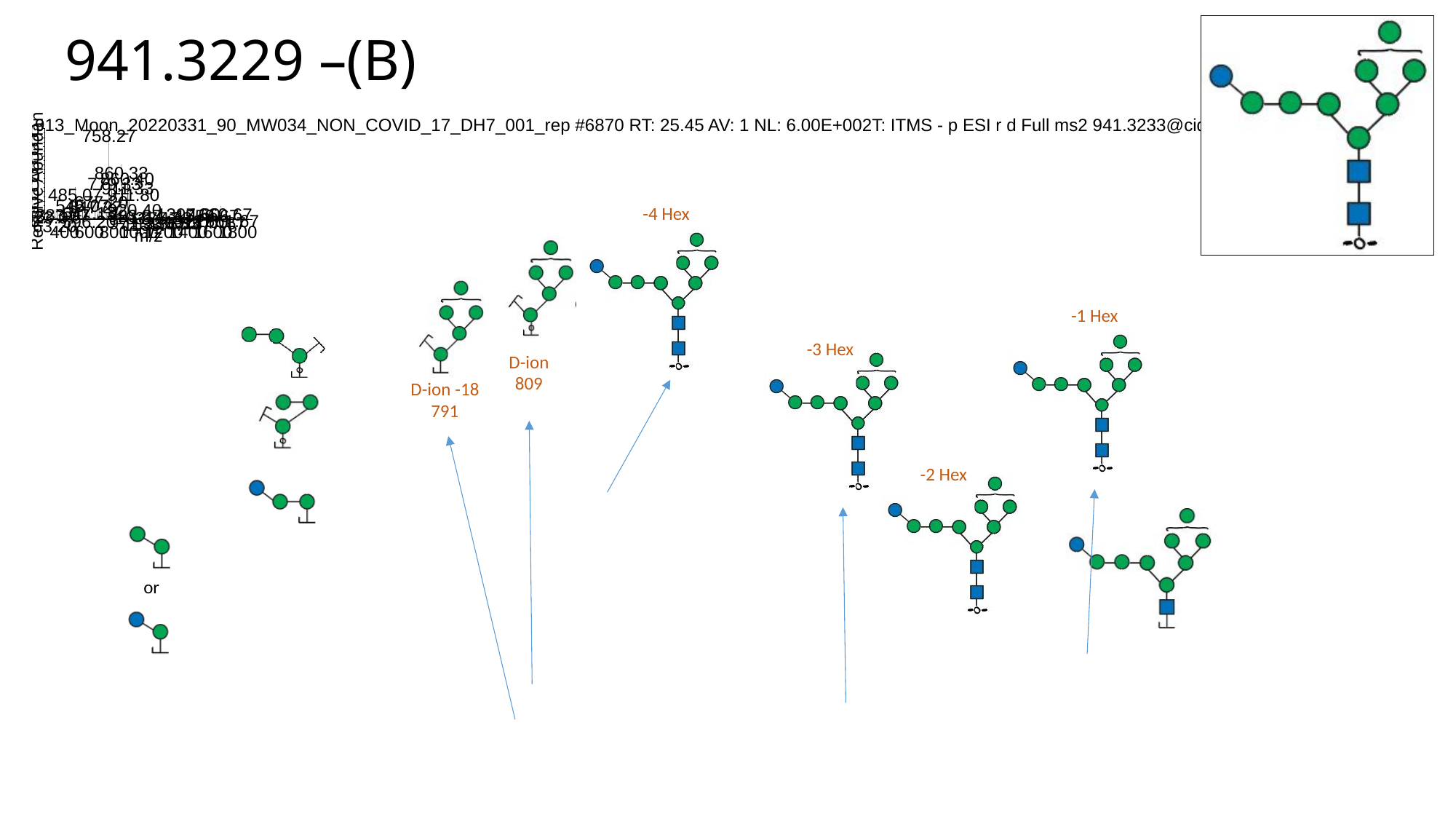

# 941.3229 –(B)
-4 Hex
-1 Hex
-3 Hex
D-ion
809
D-ion -18
791
-2 Hex
or

## Slide 39
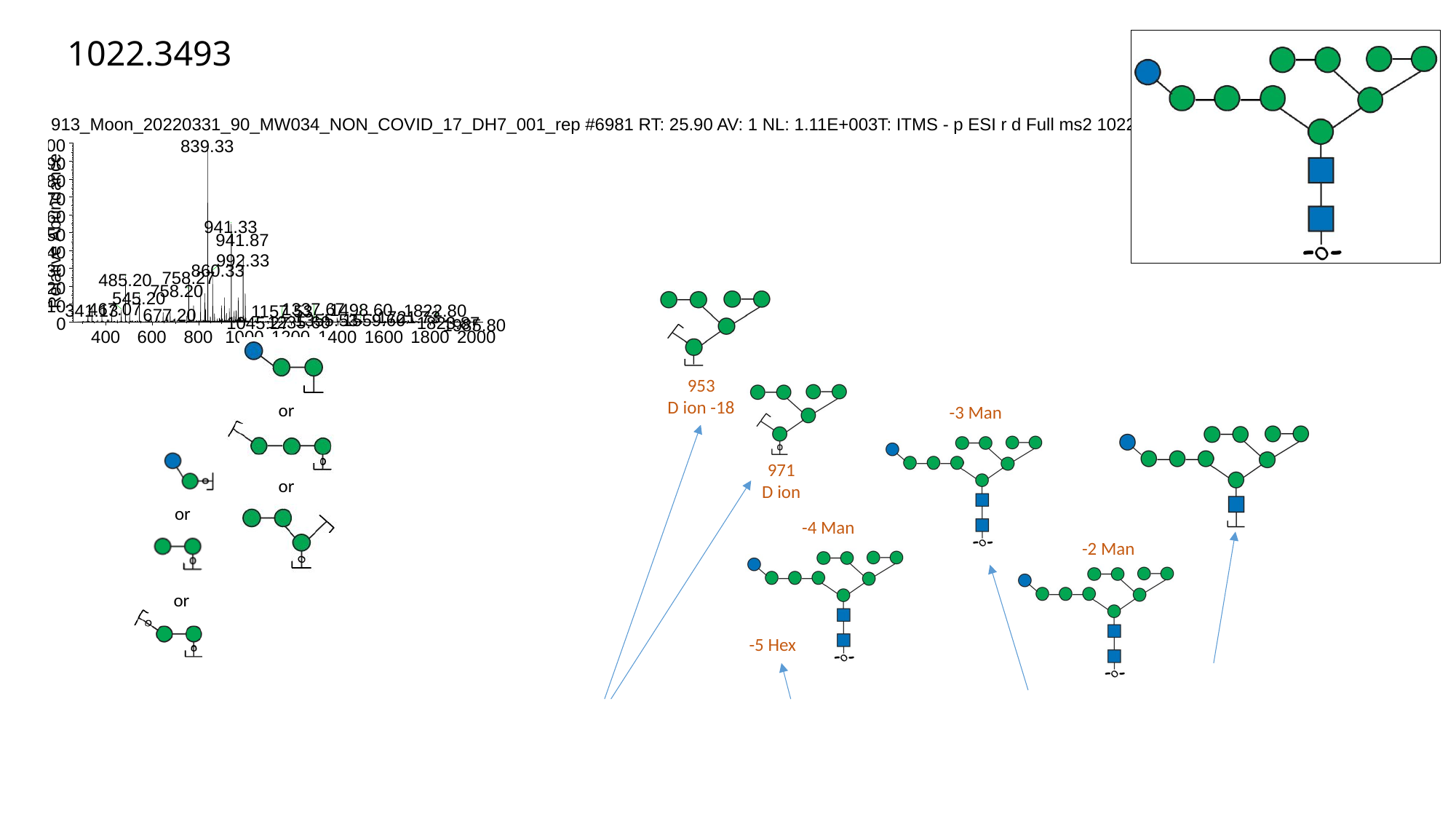

# 1022.3493
953
D ion -18
or
-3 Man
or
or
971
D ion
or
-4 Man
-2 Man
-5 Hex

## Slide 40
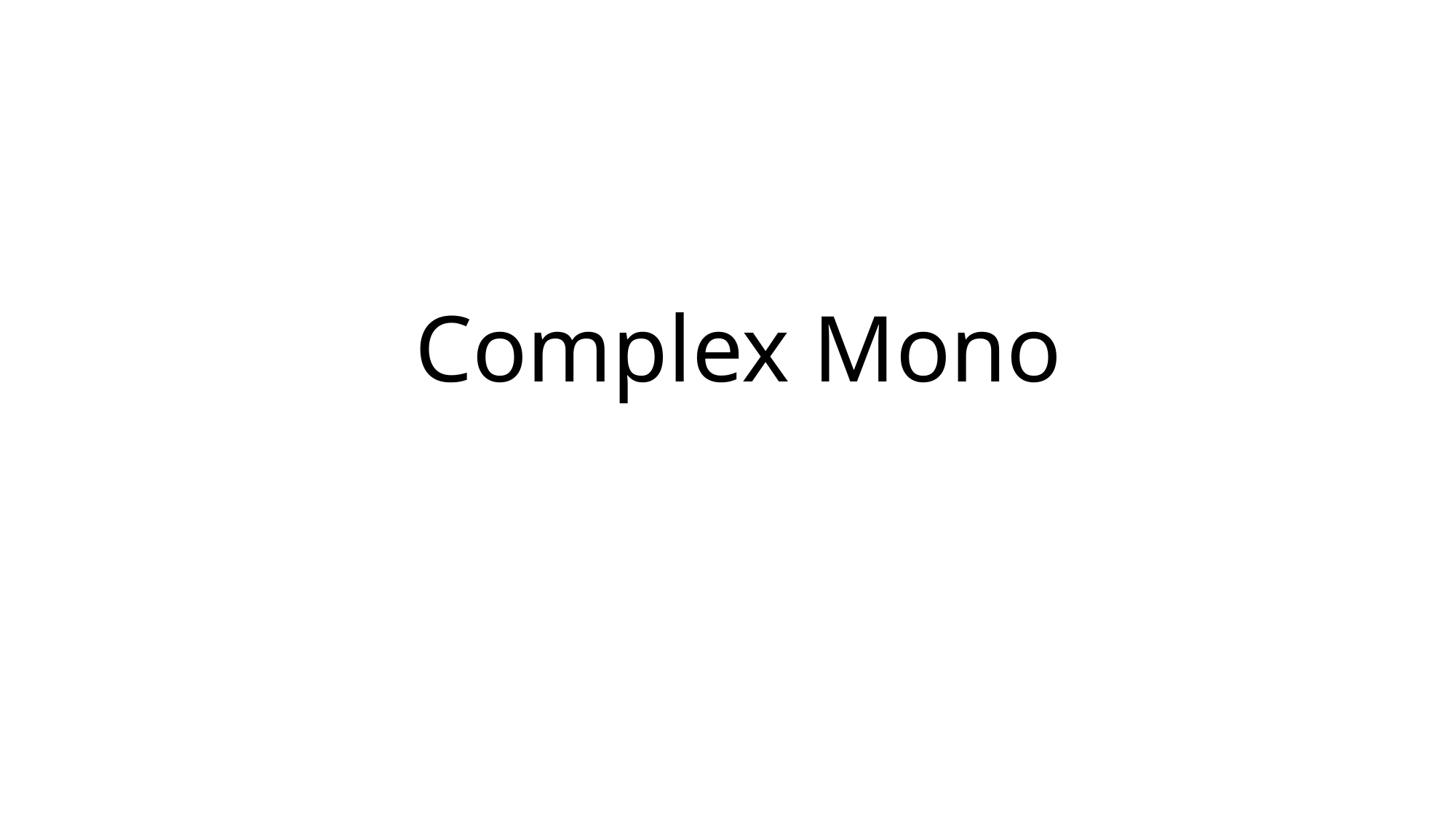

# Complex Mono

## Slide 41
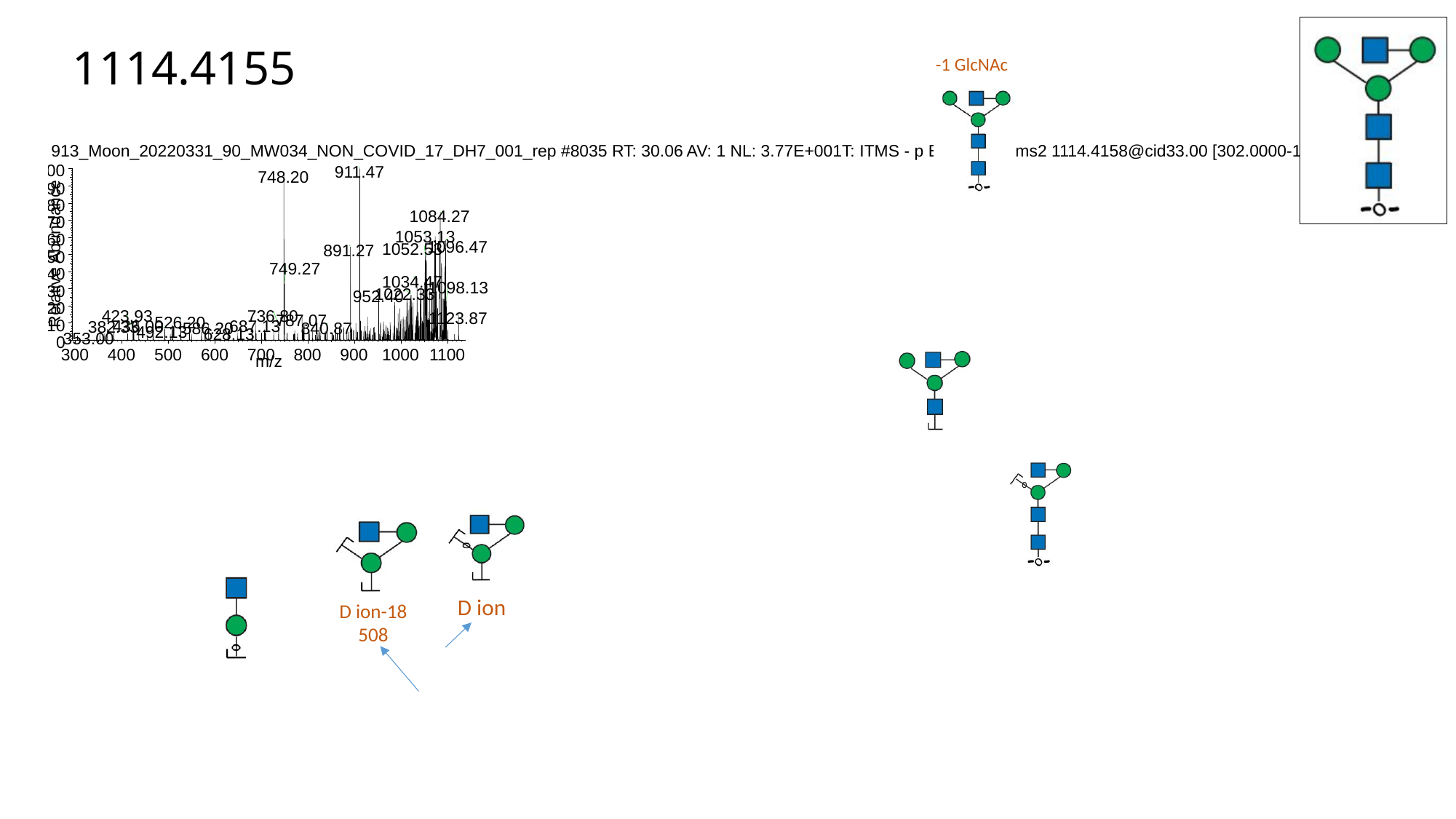

# 1114.4155
-1 GlcNAc
D ion
D ion-18
508

## Slide 42
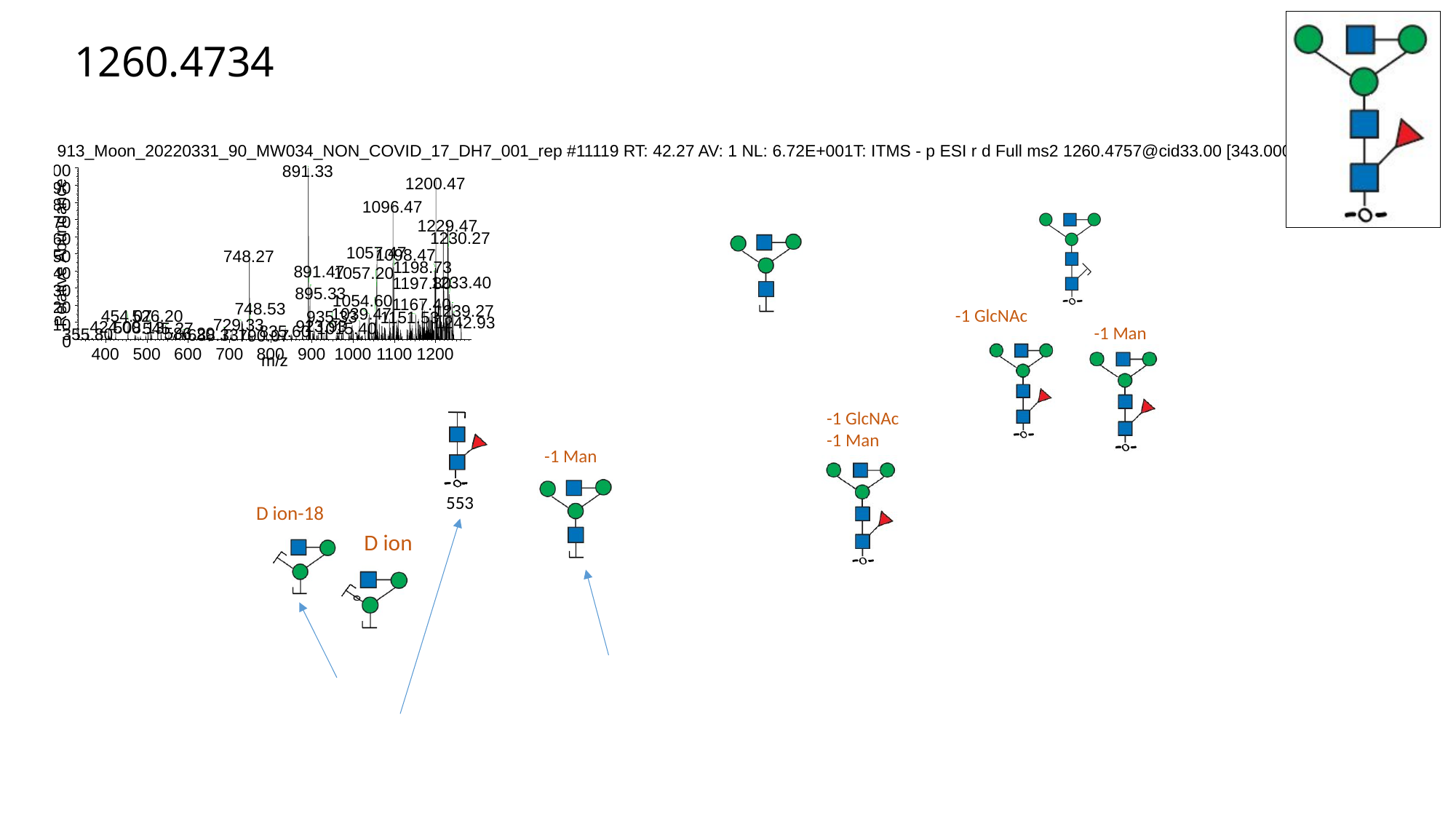

# 1260.4734
-1 GlcNAc
-1 Man
-1 GlcNAc
-1 Man
-1 Man
553
D ion-18
D ion

## Slide 43
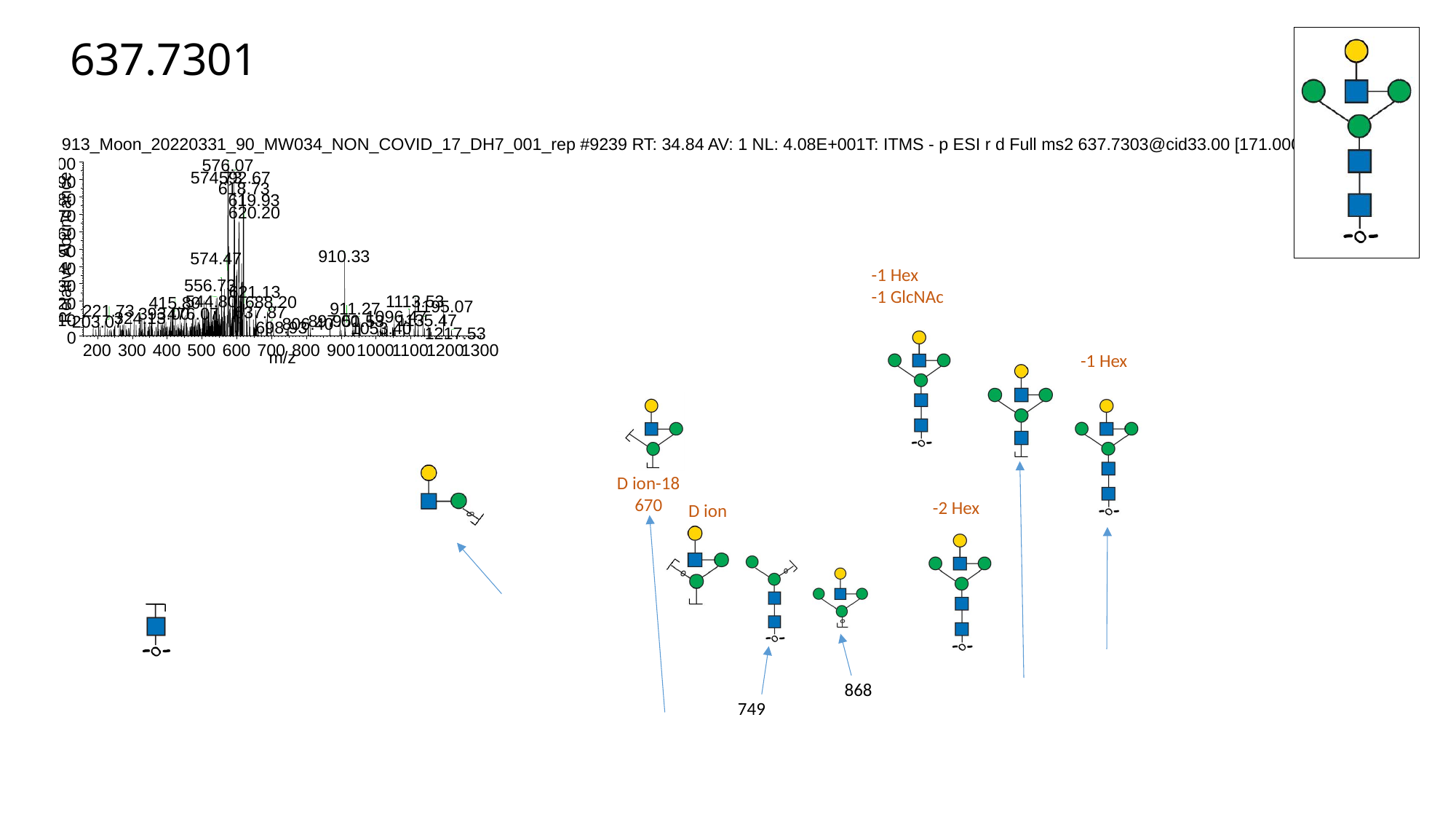

# 637.7301
-1 Hex
-1 GlcNAc
-1 Hex
D ion-18
670
-2 Hex
D ion
868
749

## Slide 44
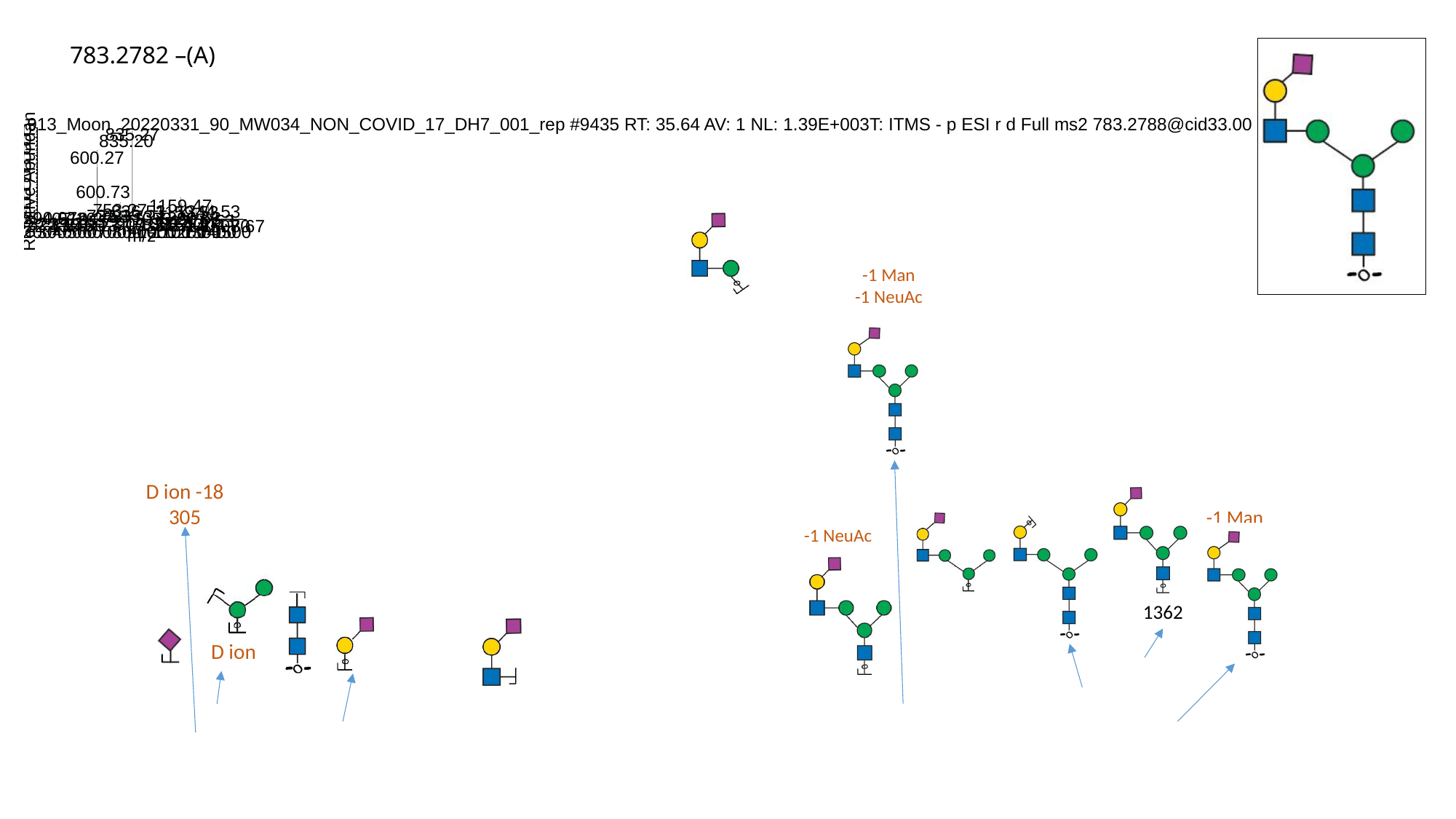

# 783.2782 –(A)
-1 Man
-1 NeuAc
D ion -18
305
-1 Man
-1 NeuAc
1362
D ion

## Slide 45
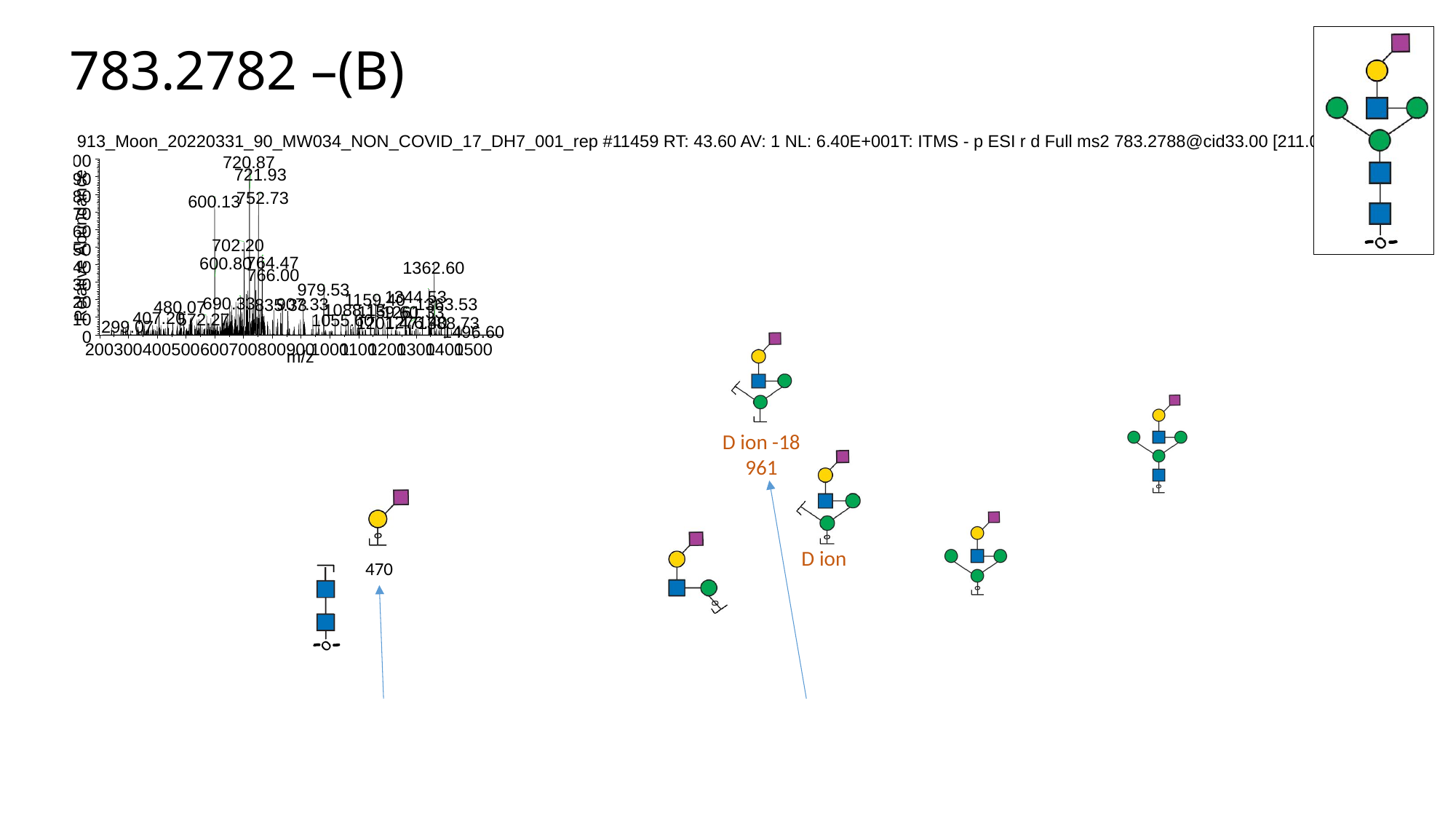

# 783.2782 –(B)
D ion -18
961
D ion
470

## Slide 46
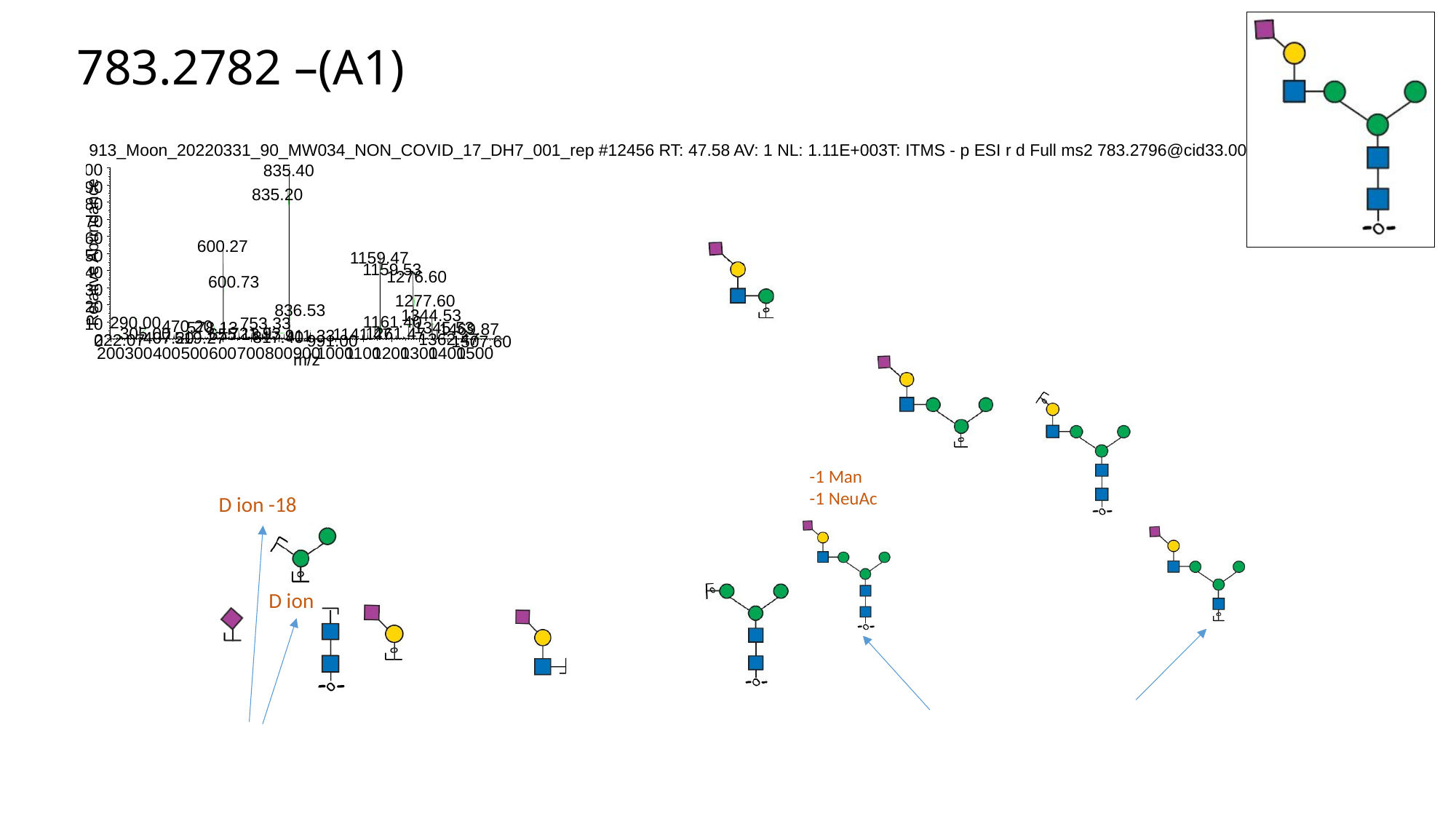

# 783.2782 –(A1)
-1 Man
-1 NeuAc
D ion -18
D ion

## Slide 47
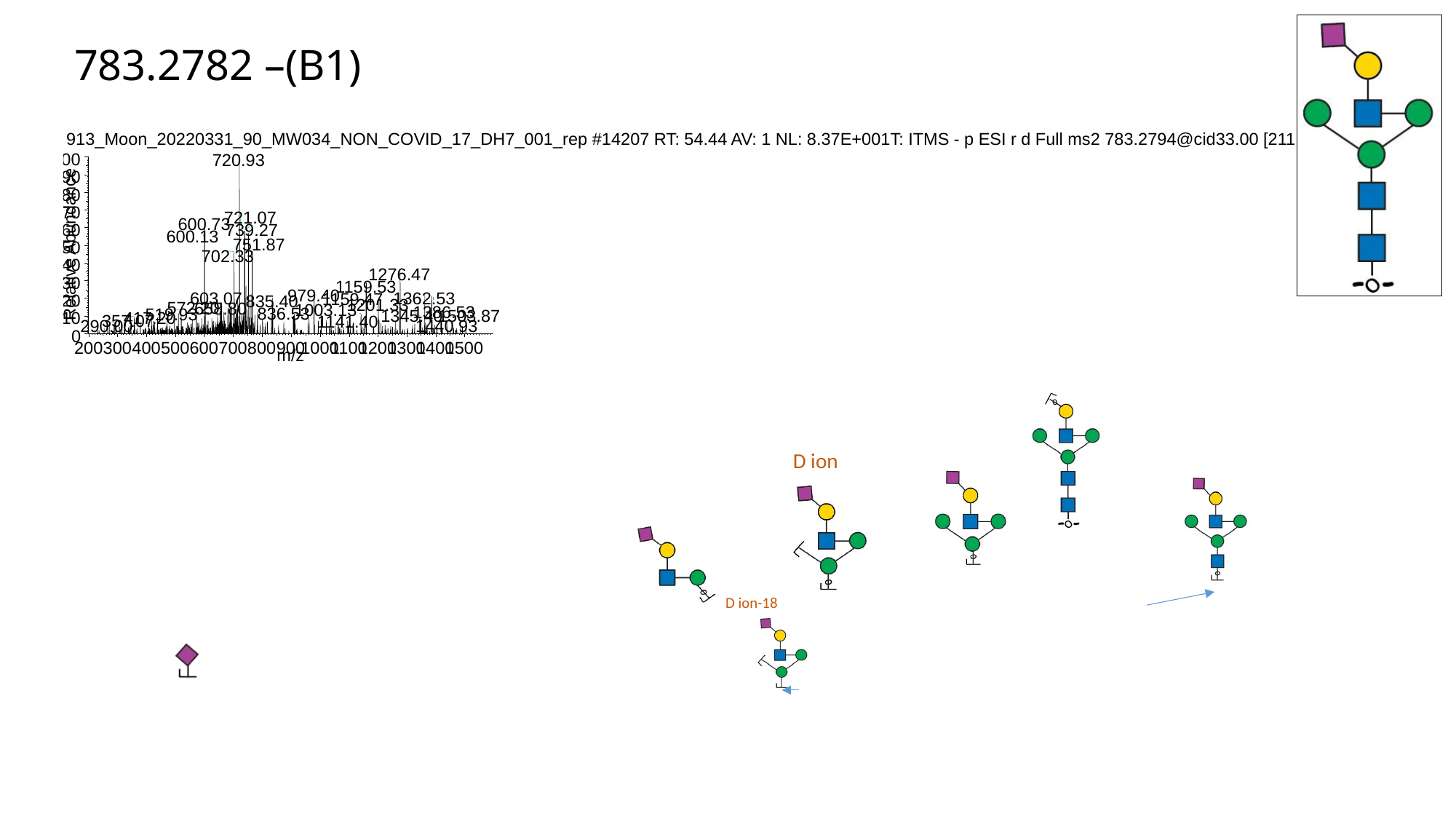

# 783.2782 –(B1)
D ion
D ion-18

## Slide 48
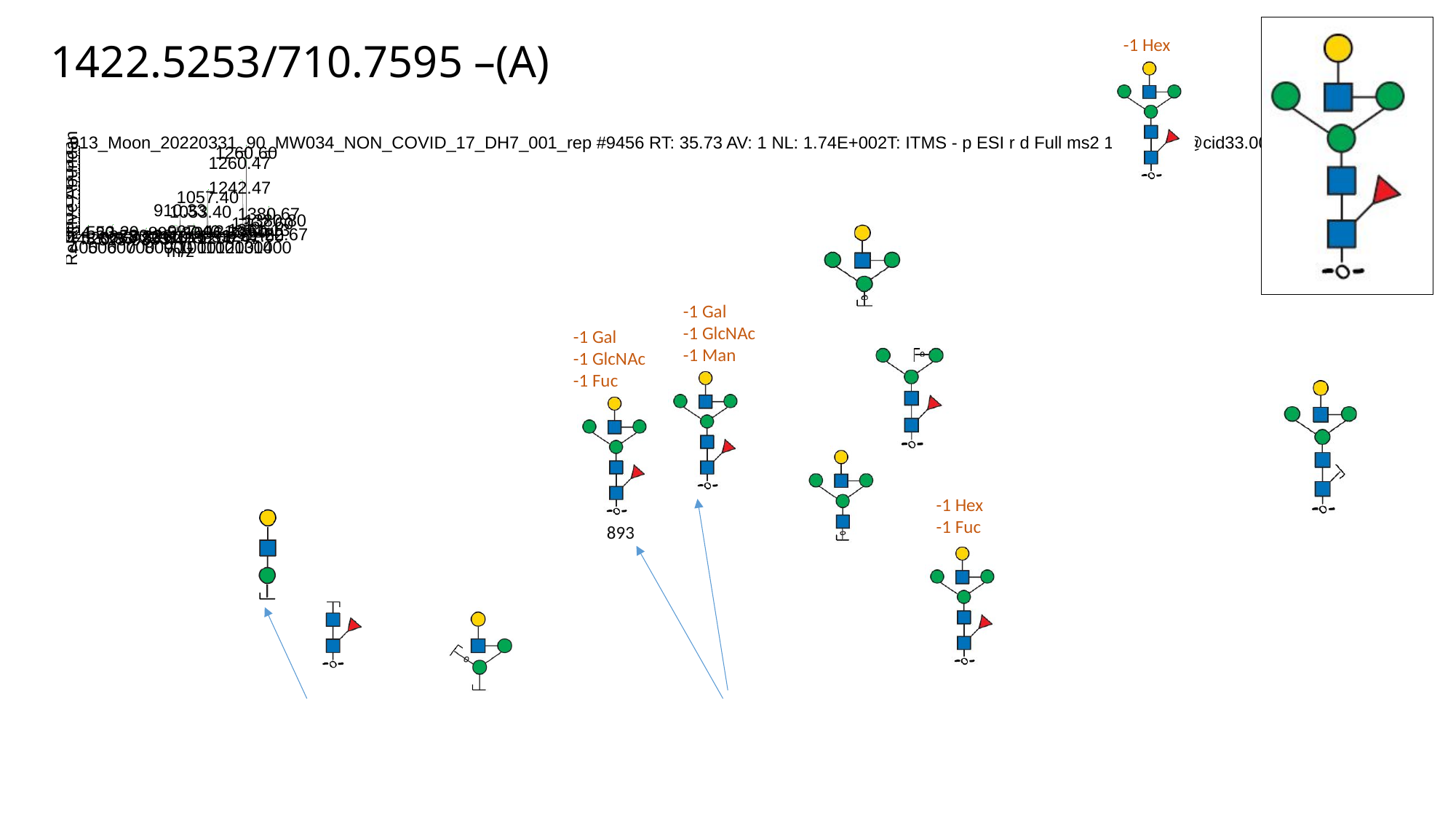

-1 Hex
# 1422.5253/710.7595 –(A)
-1 Gal
-1 GlcNAc
-1 Man
-1 Gal
-1 GlcNAc
-1 Fuc
-1 Hex
-1 Fuc
893

## Slide 49
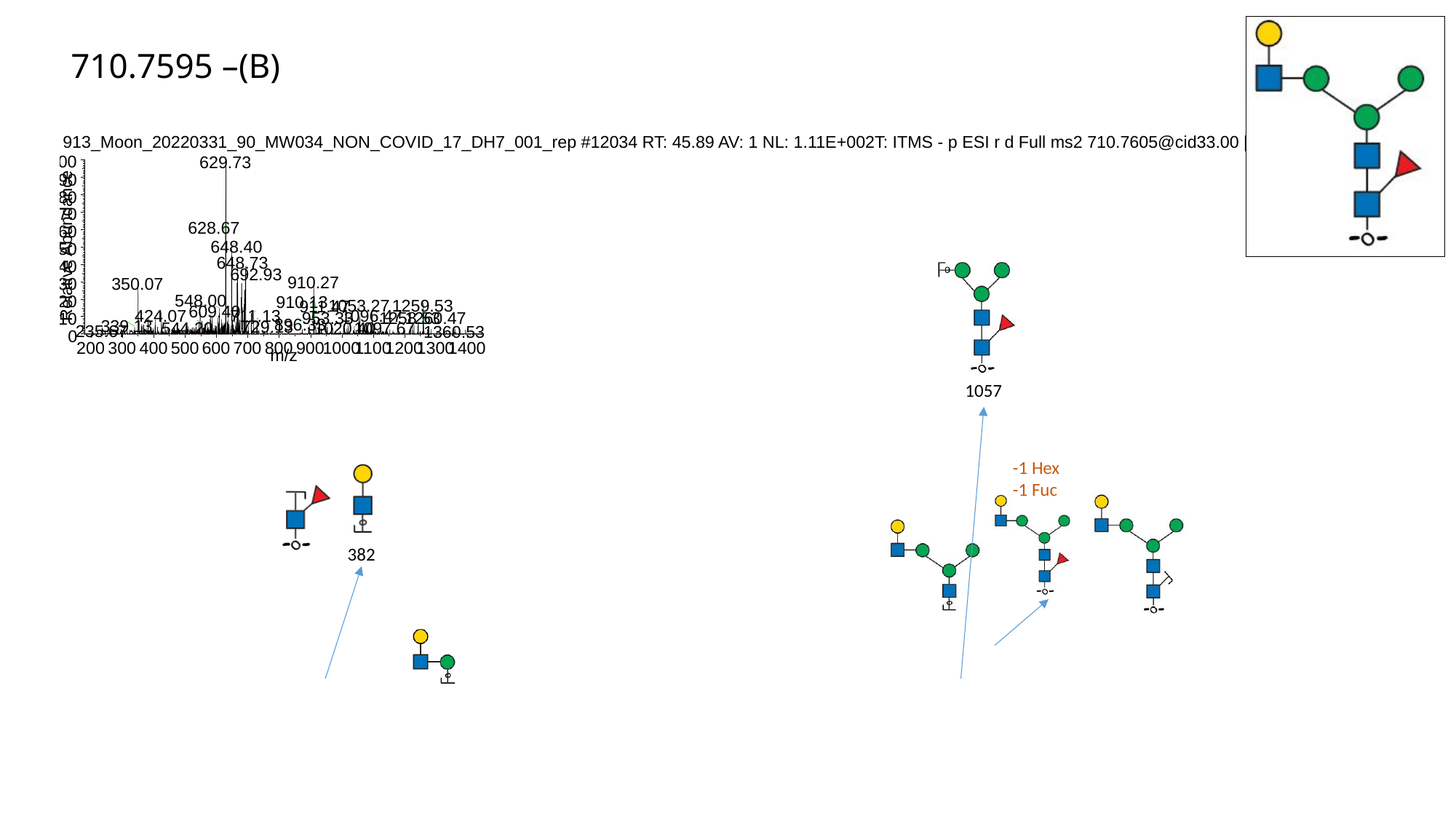

# 710.7595 –(B)
1057
-1 Hex
-1 Fuc
382

## Slide 50
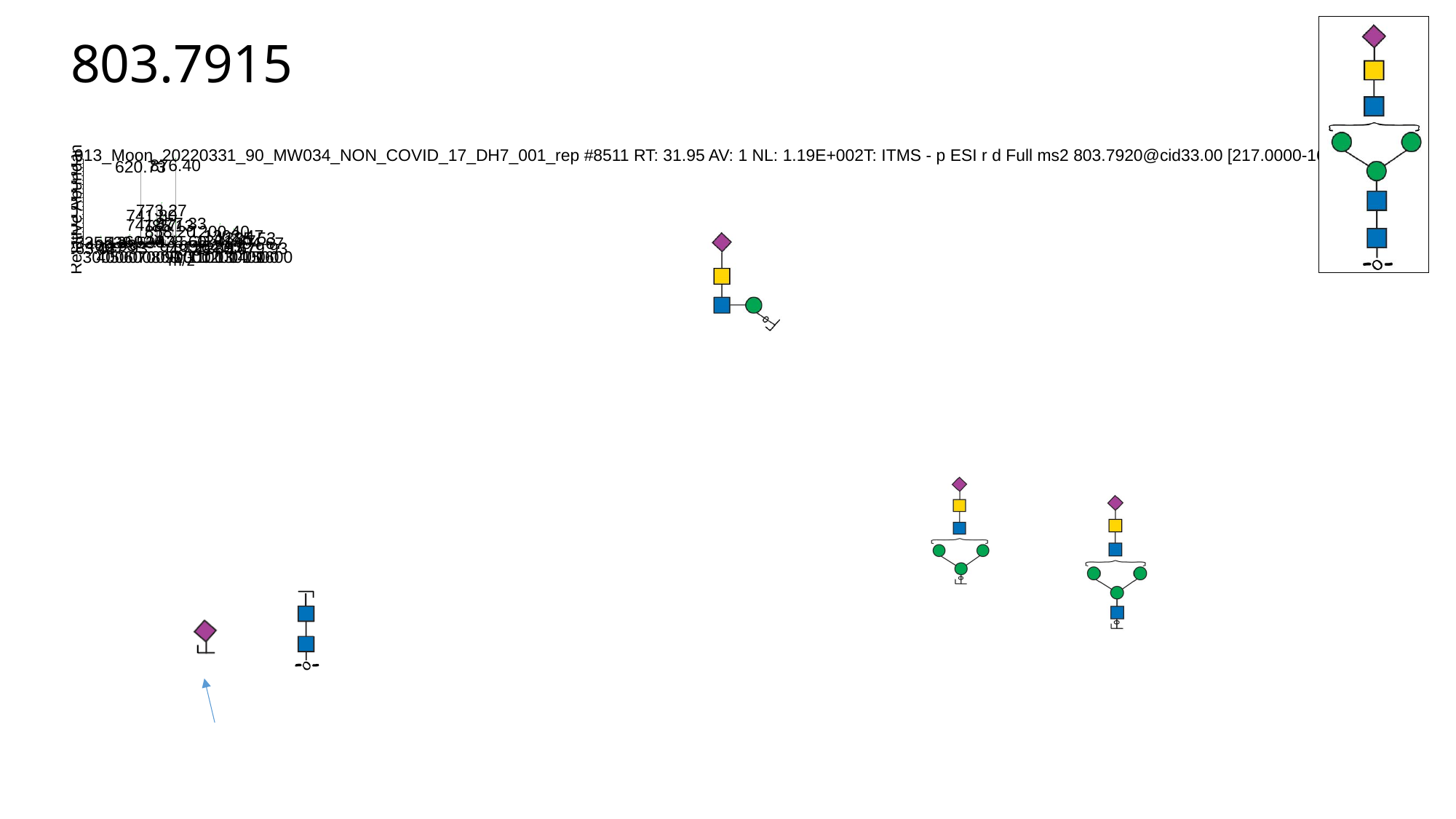

# 803.7915

## Slide 51
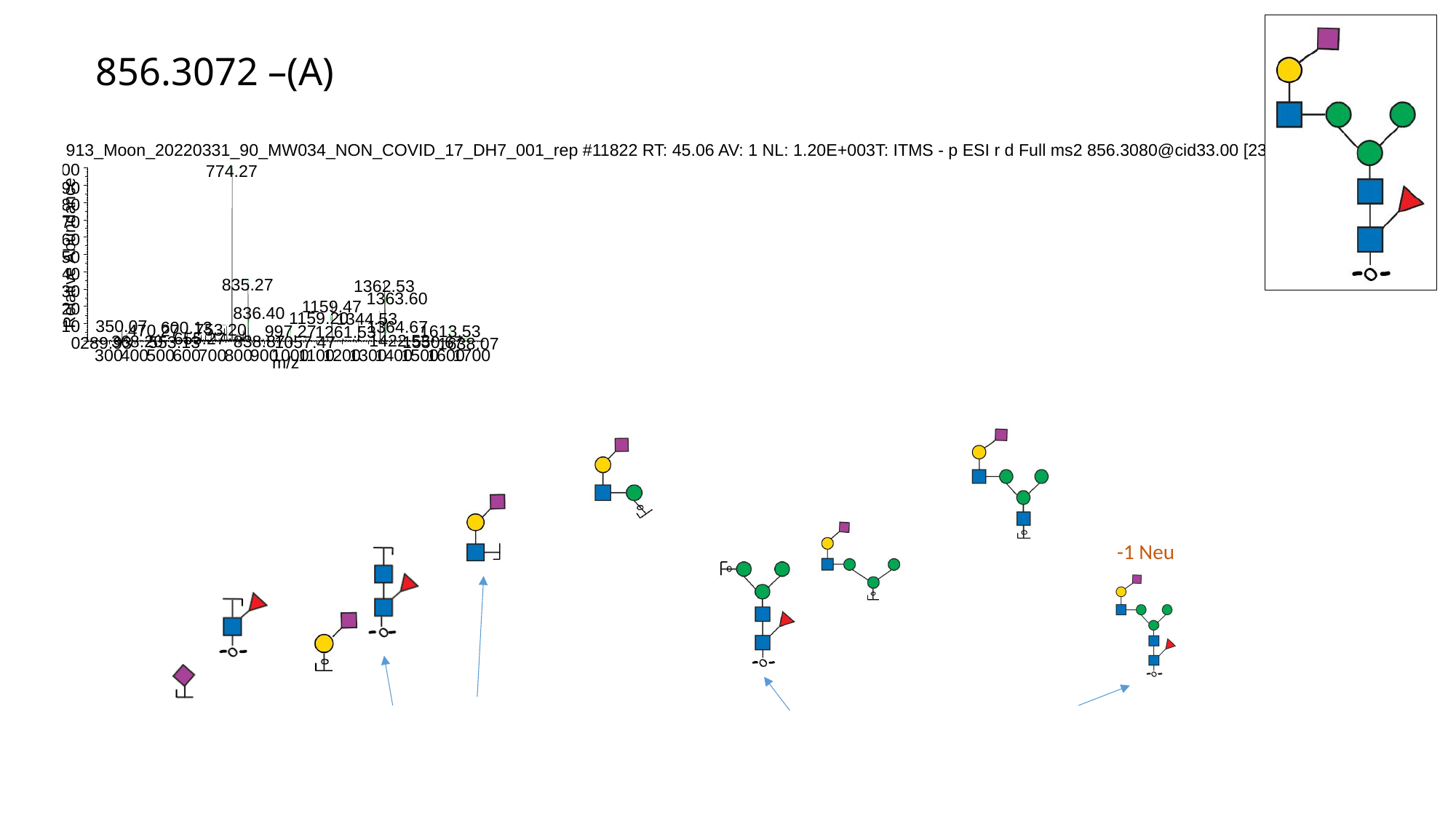

# 856.3072 –(A)
-1 Neu

## Slide 52
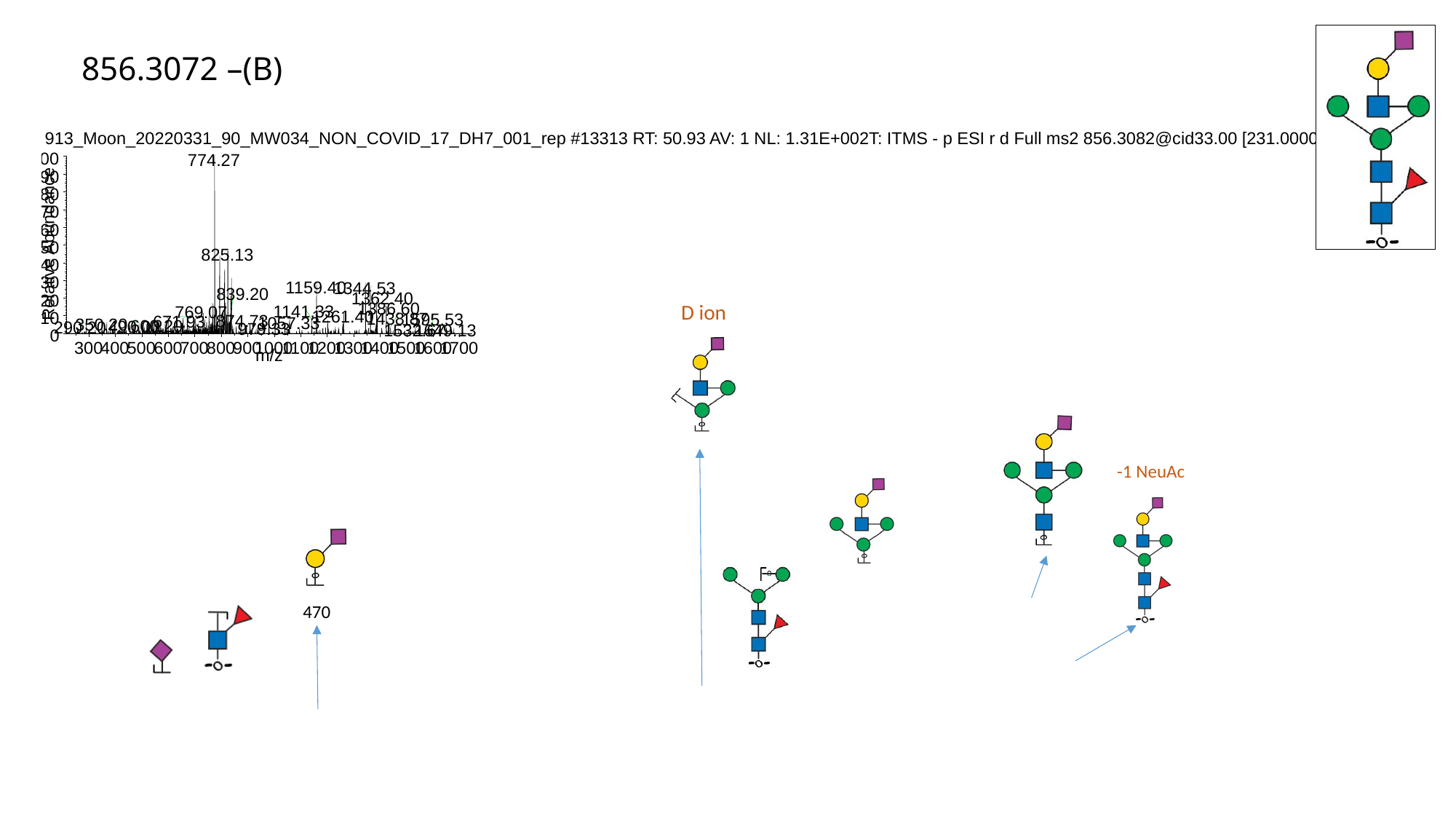

# 856.3072 –(B)
D ion
-1 NeuAc
470

## Slide 53
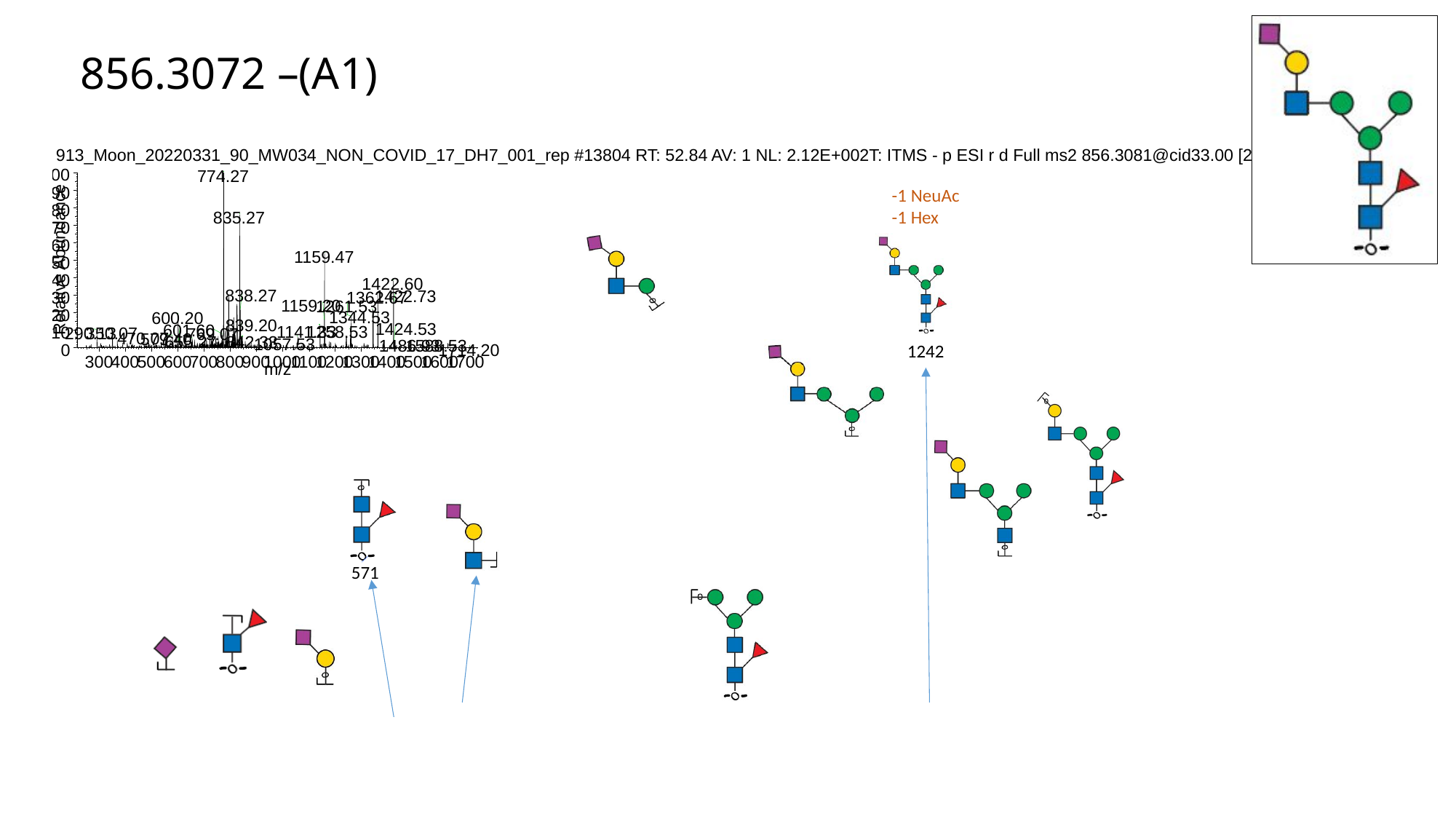

# 856.3072 –(A1)
-1 NeuAc
-1 Hex
1242
571

## Slide 54
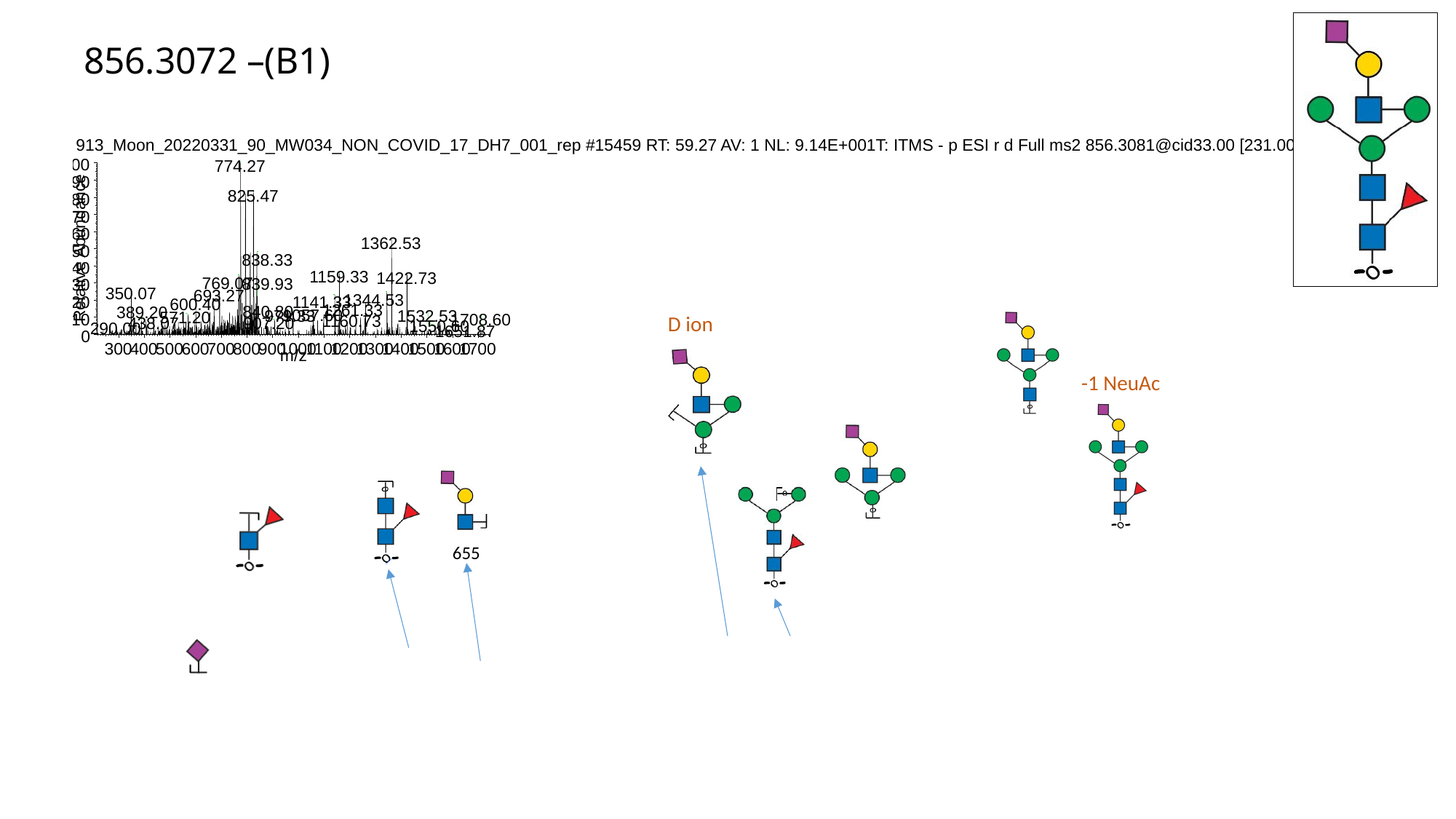

# 856.3072 –(B1)
D ion
-1 NeuAc
655

## Slide 55
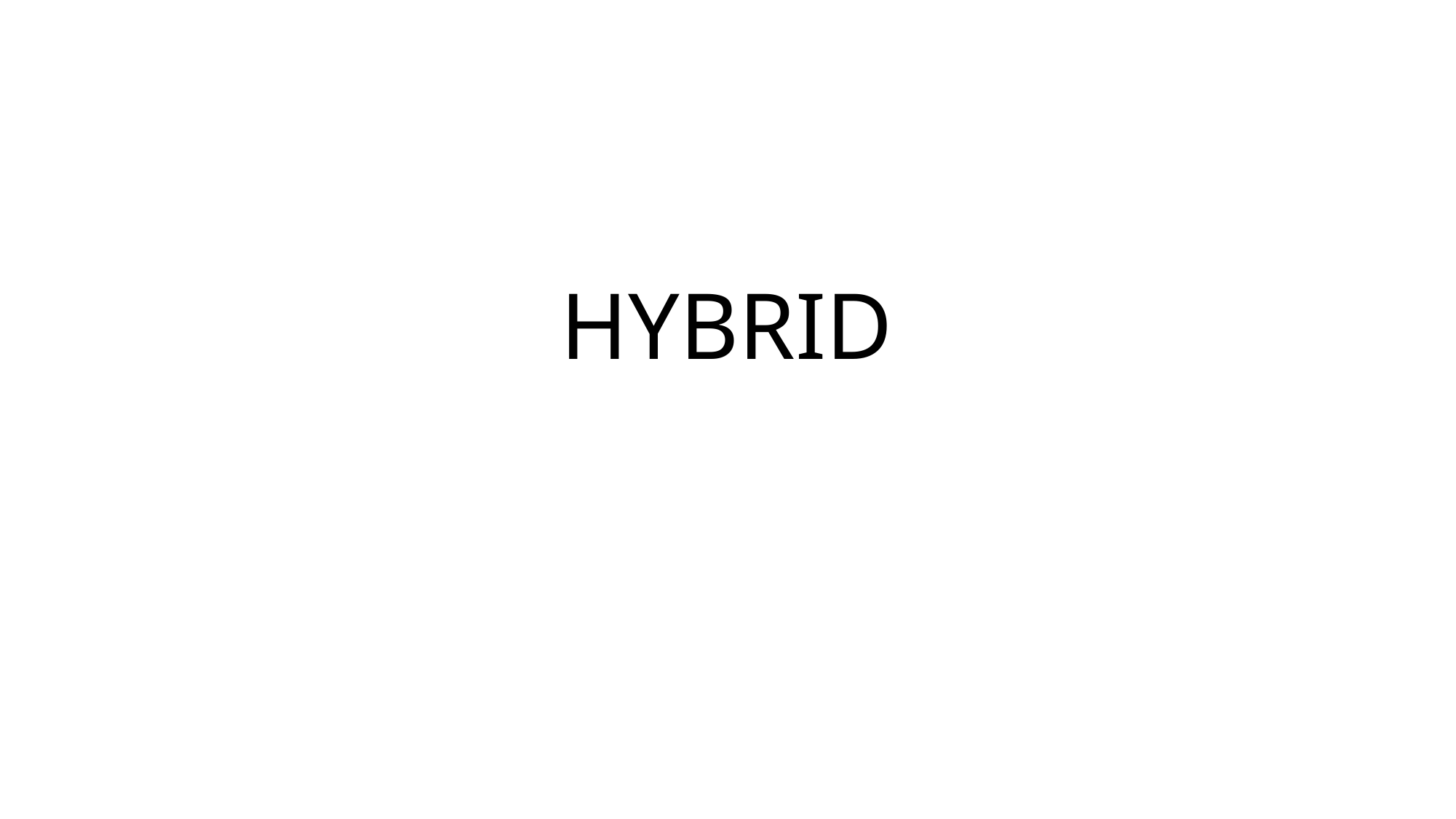

# HYBRID

## Slide 56
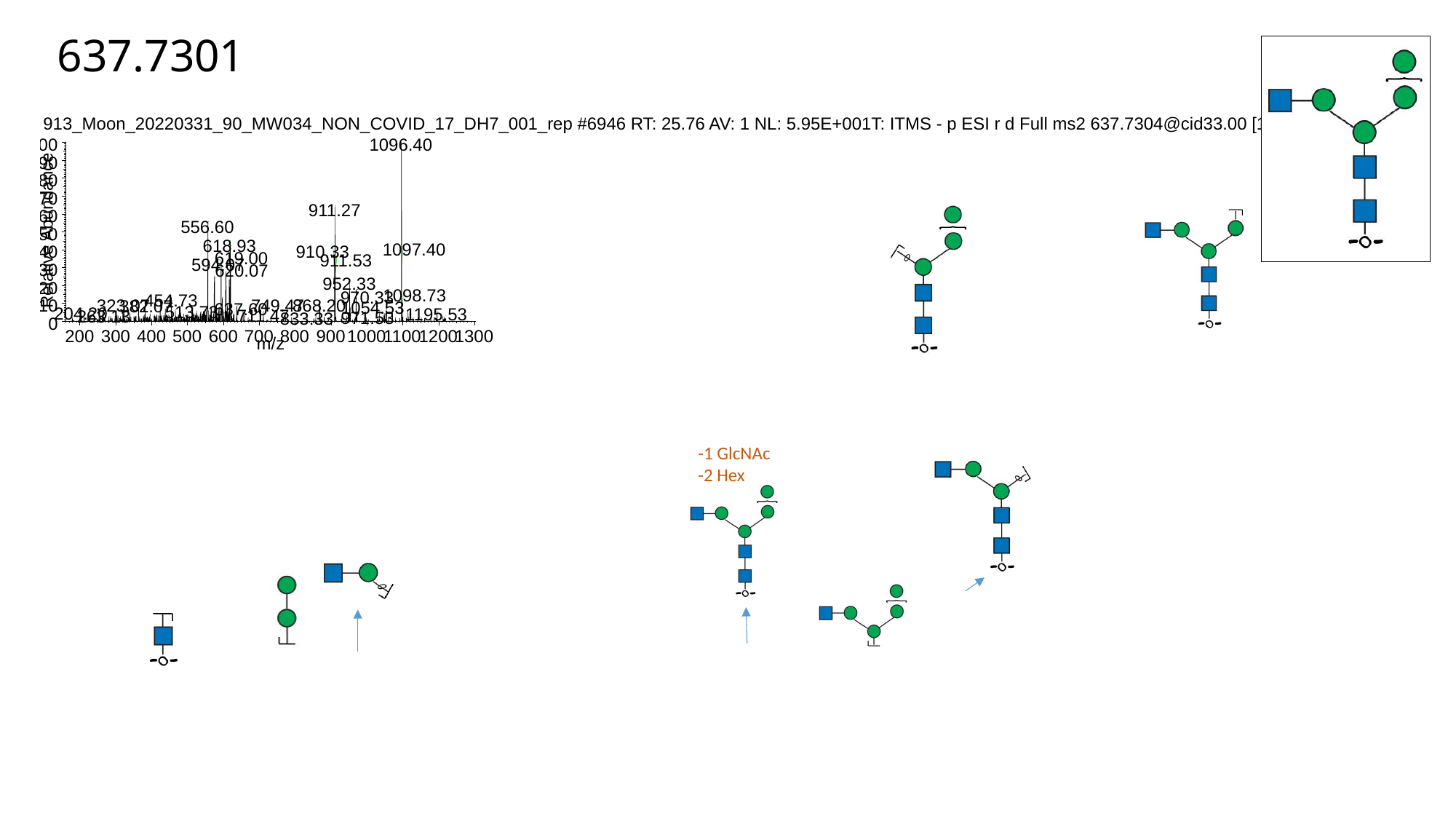

# 637.7301
-1 GlcNAc
-2 Hex

## Slide 57
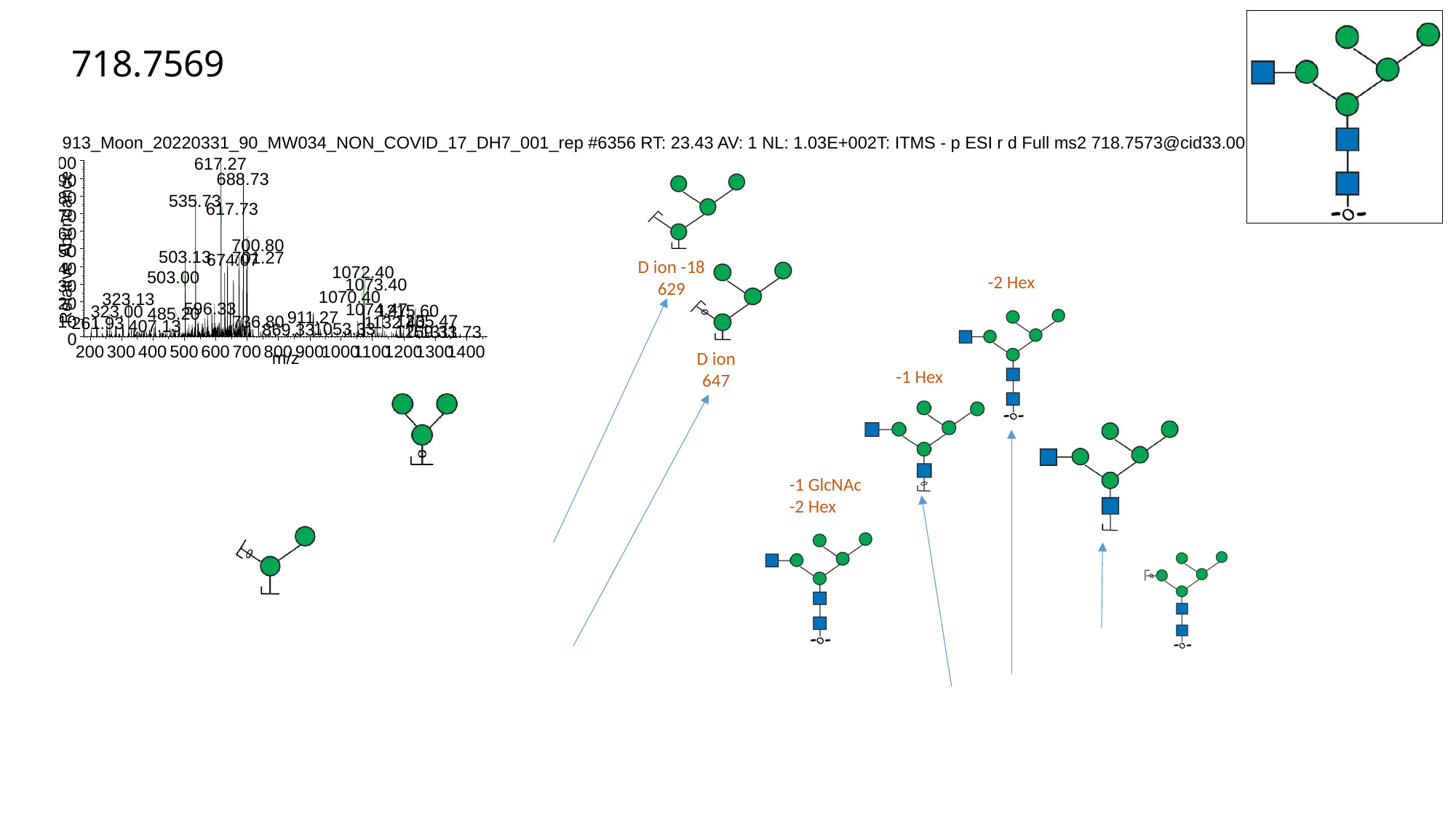

# 718.7569
D ion -18
629
-2 Hex
D ion
647
-1 Hex
-1 GlcNAc
-2 Hex

## Slide 58
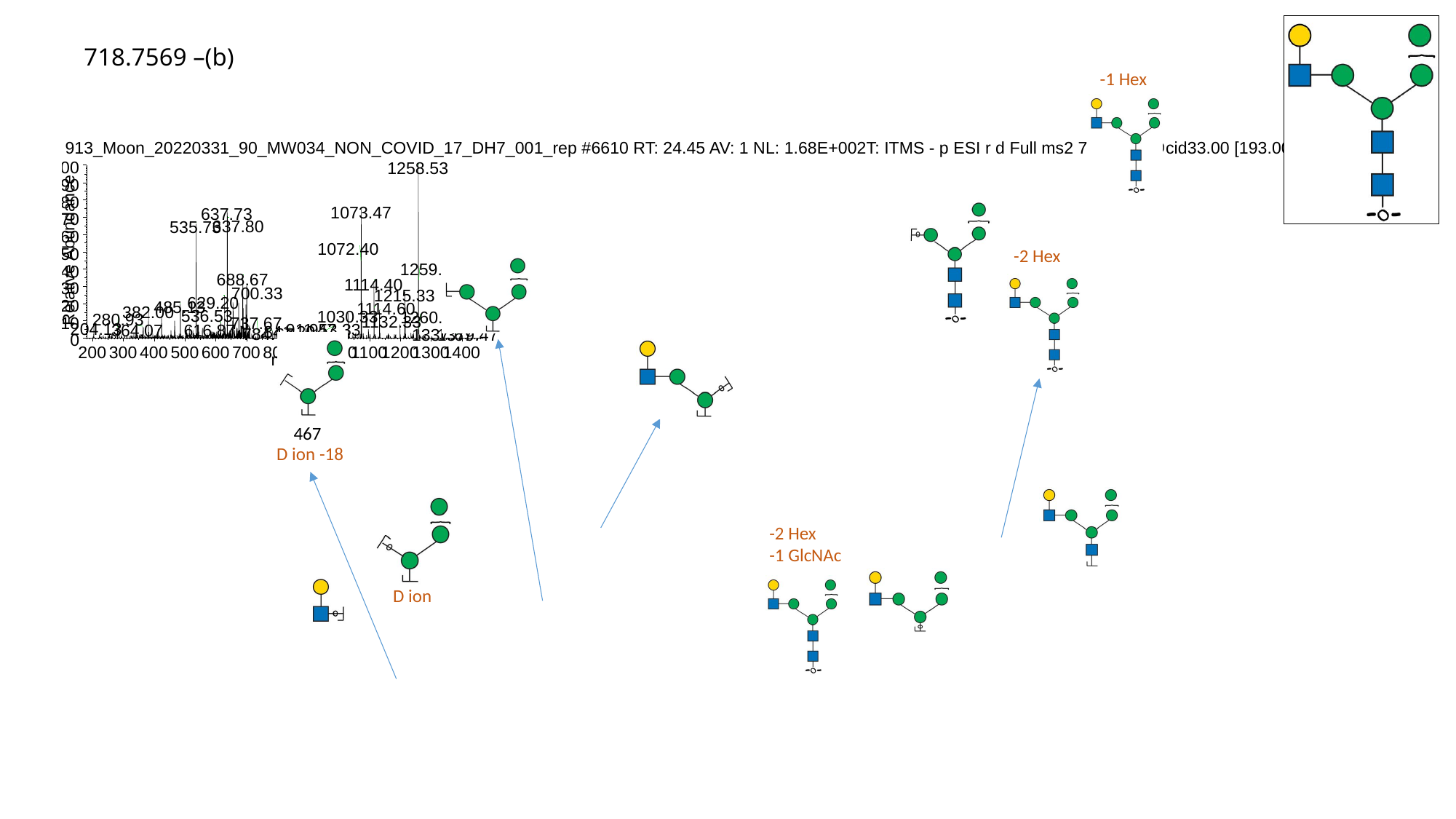

# 718.7569 –(b)
-1 Hex
-2 Hex
467
D ion -18
-2 Hex
-1 GlcNAc
D ion

## Slide 59
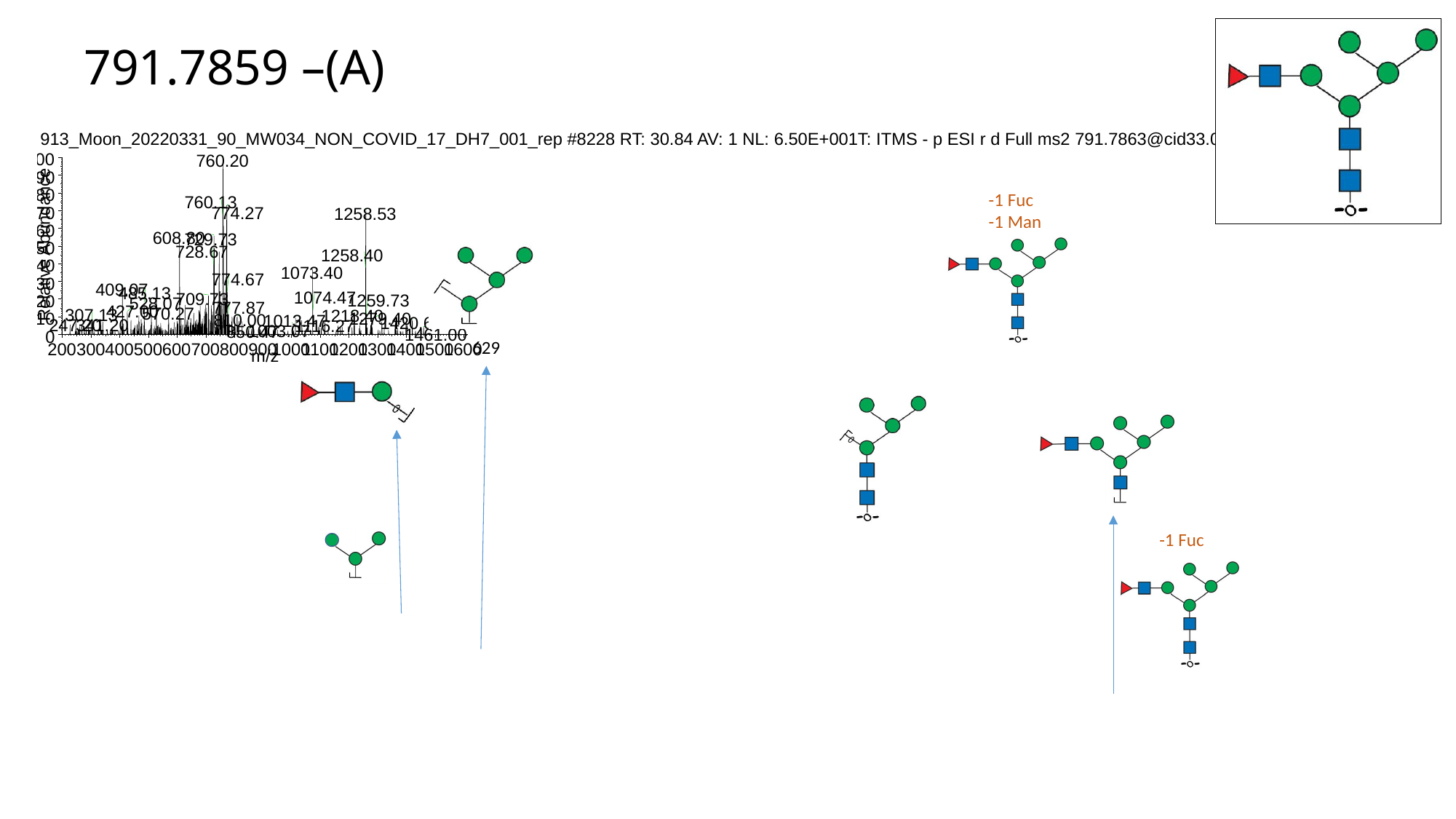

# 791.7859 –(A)
-1 Fuc
-1 Man
629
-1 Fuc

## Slide 60
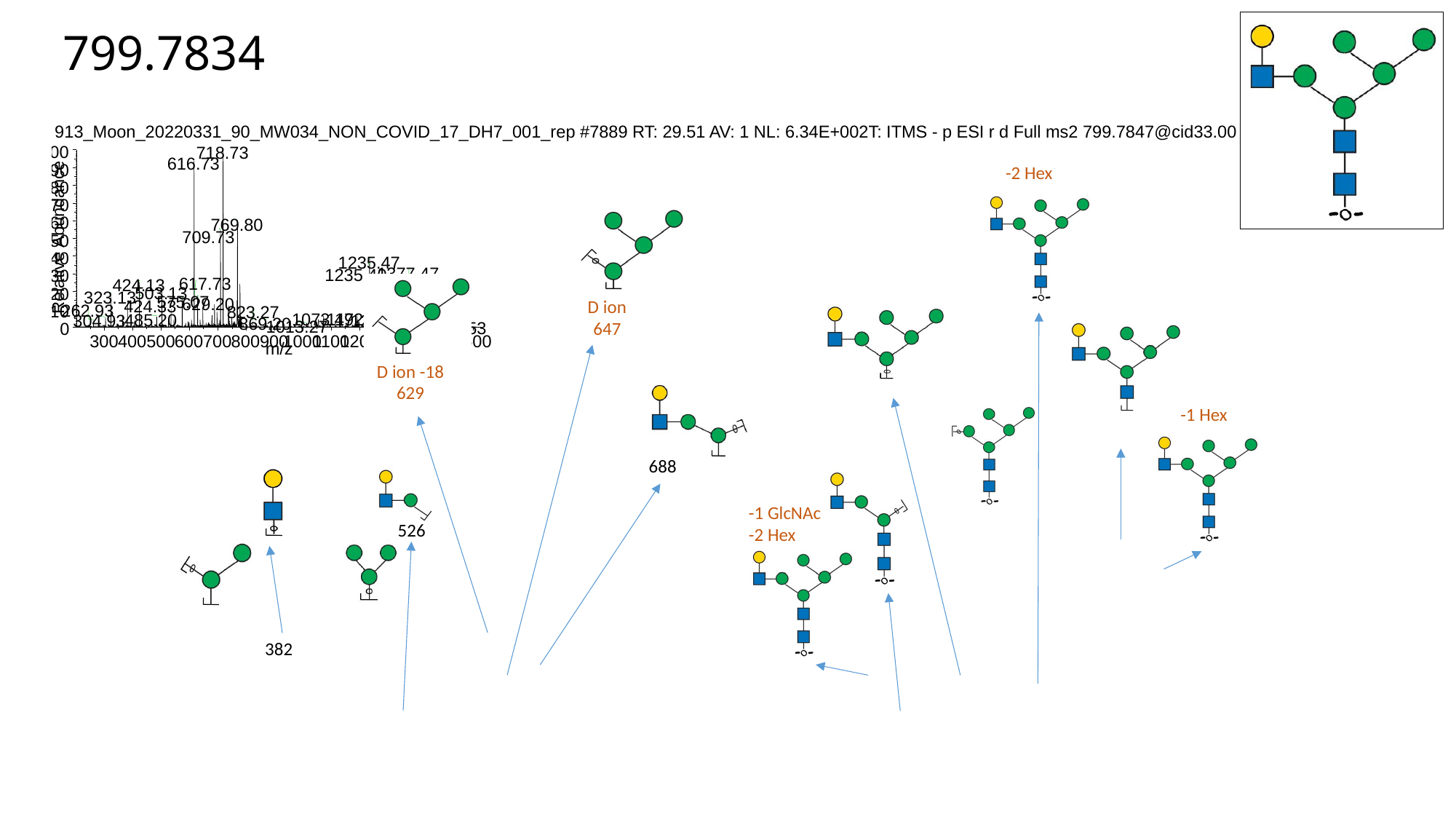

# 799.7834
-2 Hex
D ion
647
D ion -18
629
-1 Hex
688
-1 GlcNAc
-2 Hex
526
382

## Slide 61
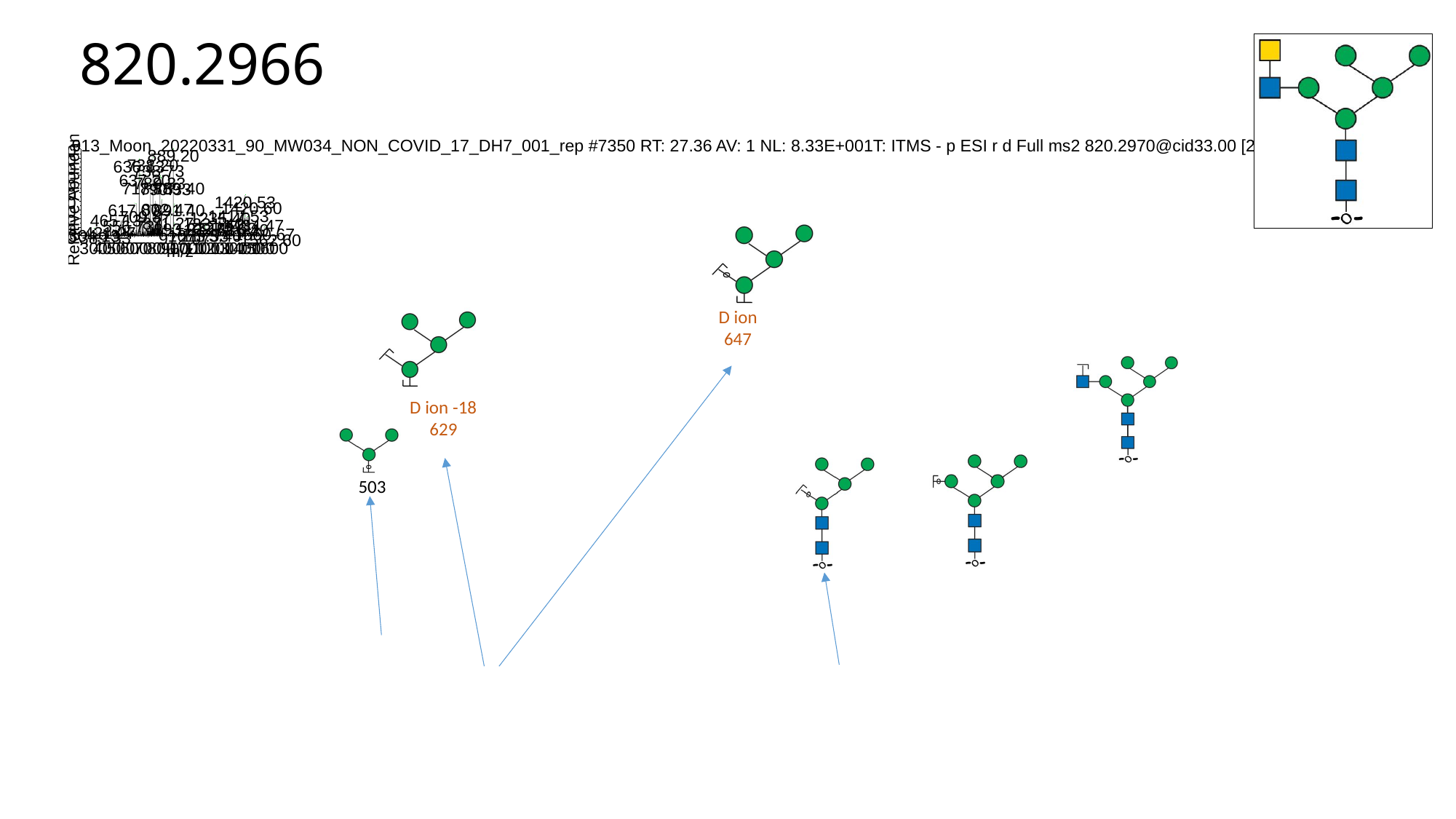

# 820.2966
D ion
647
D ion -18
629
503

## Slide 62
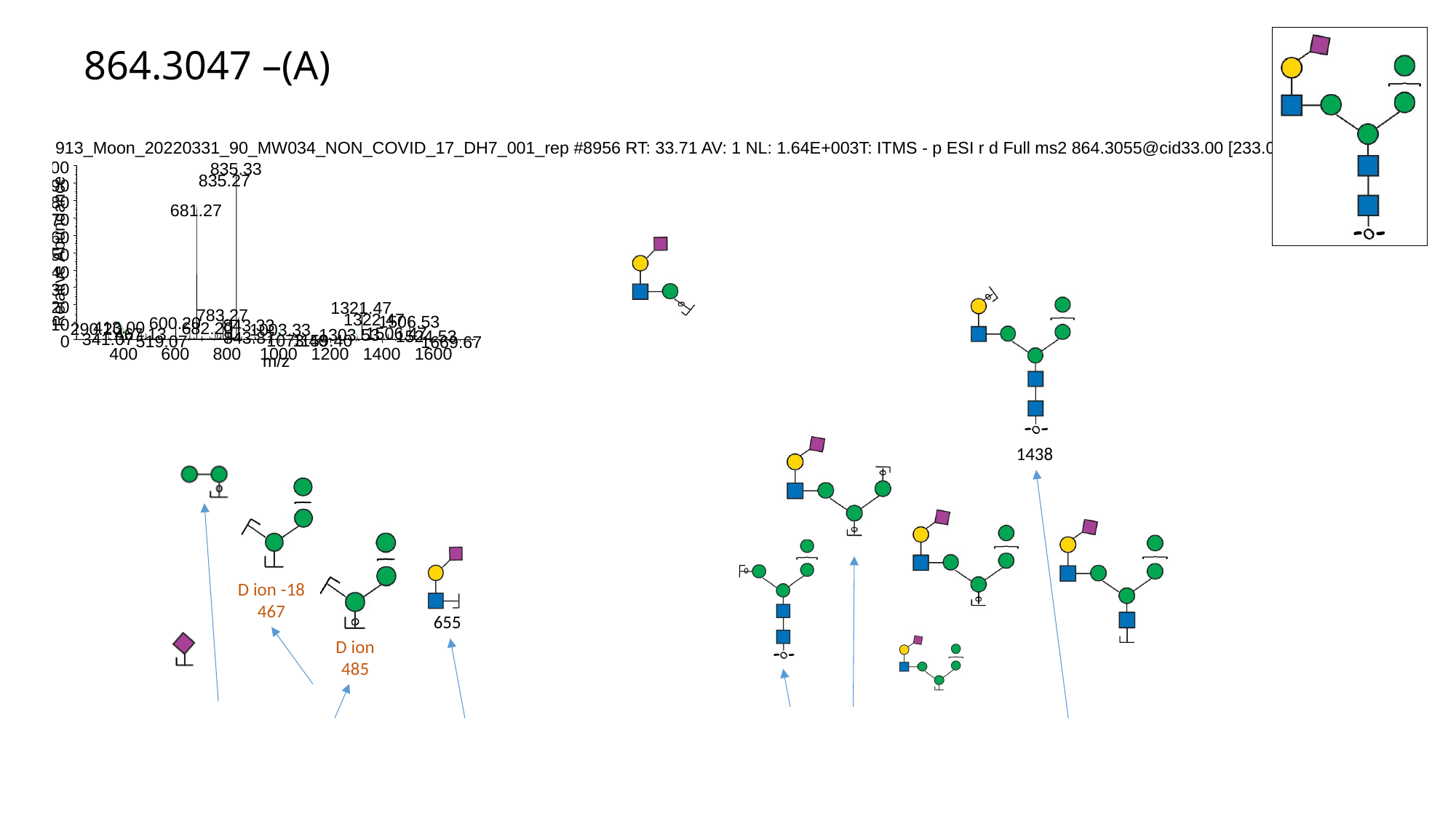

# 864.3047 –(A)
1438
D ion -18
467
655
D ion
485

## Slide 63
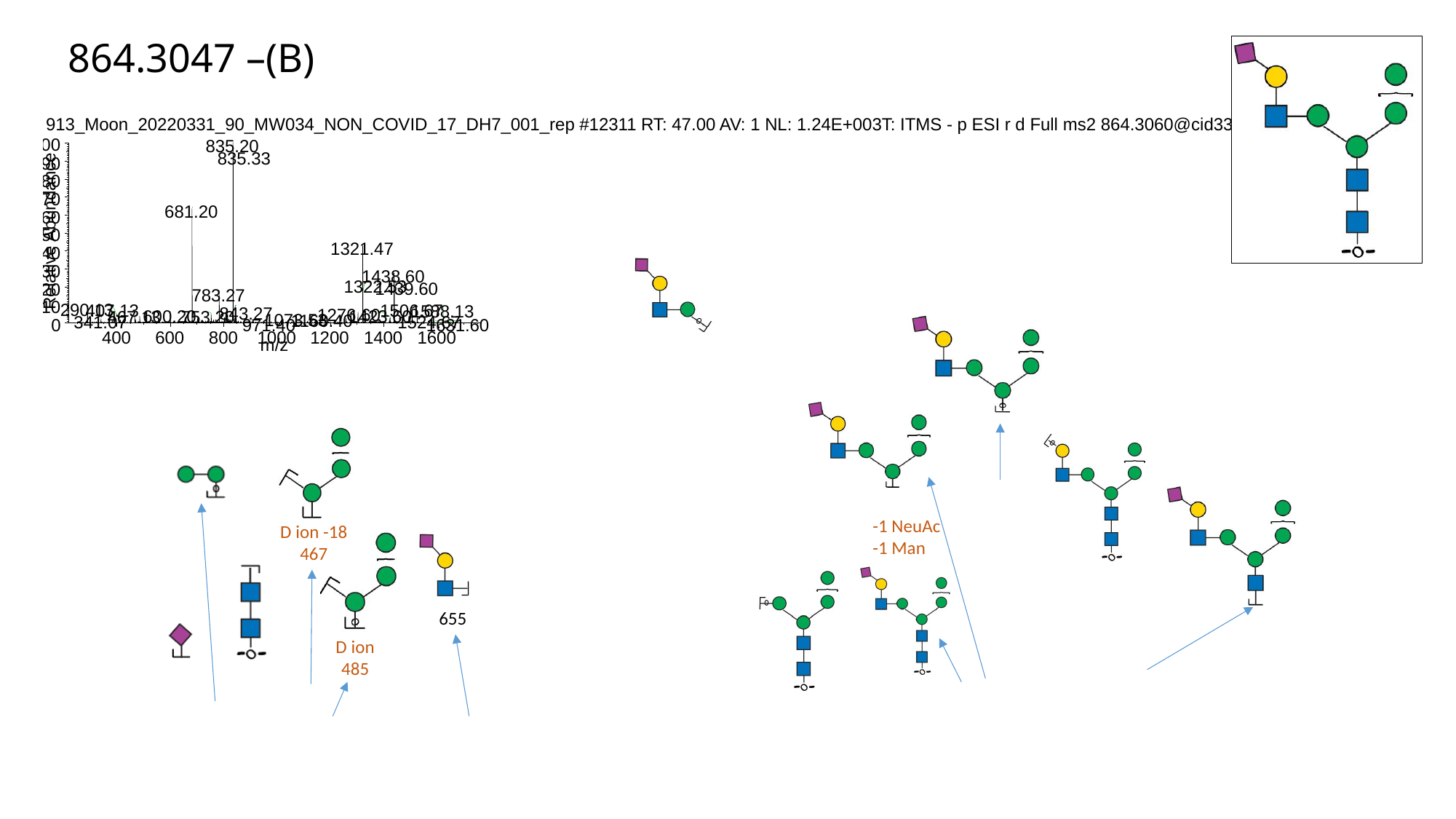

# 864.3047 –(B)
-1 NeuAc
-1 Man
D ion -18
467
655
D ion
485

## Slide 64
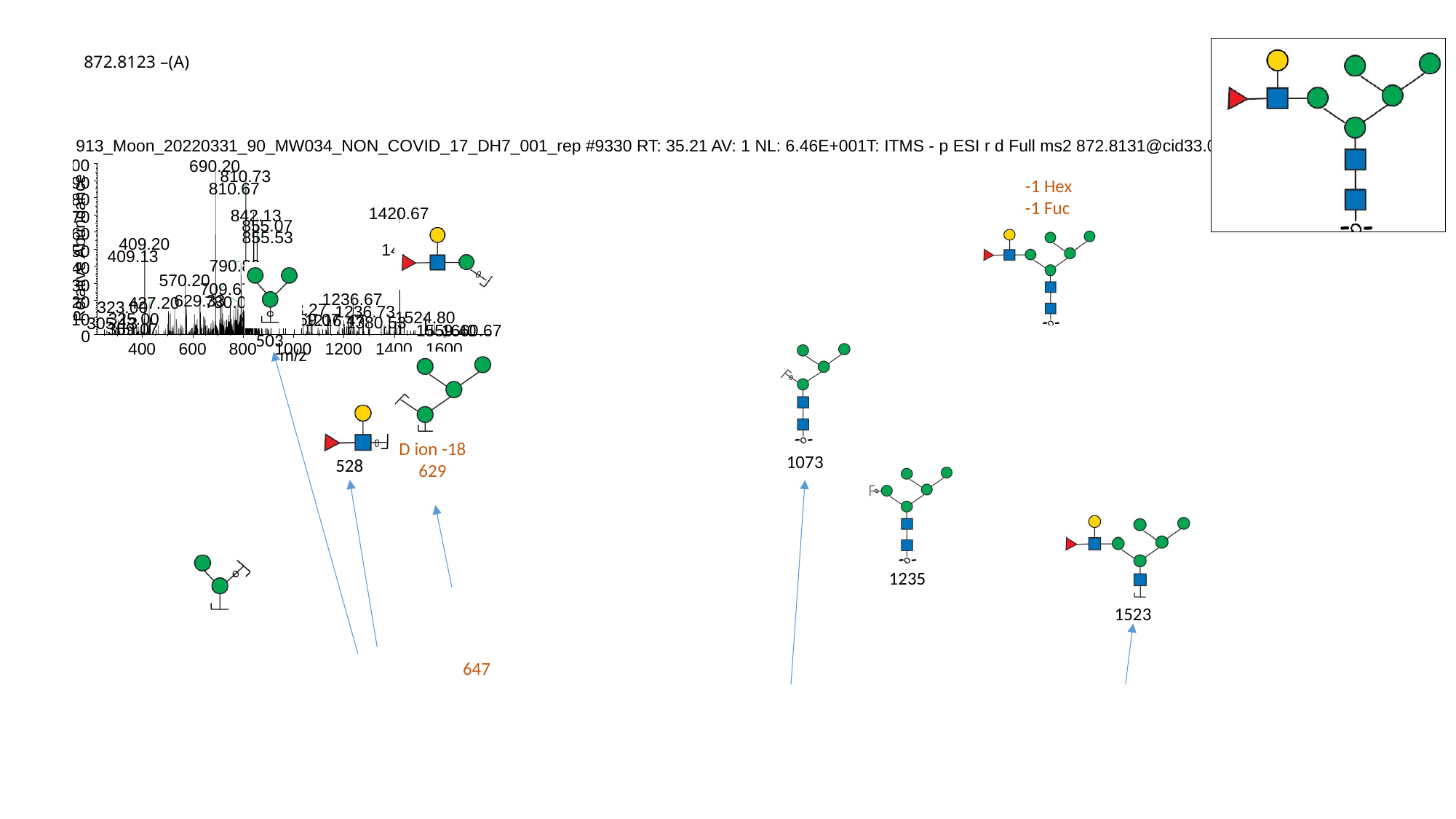

# 872.8123 –(A)
-1 Hex
-1 Fuc
503
D ion -18
629
1073
528
1235
1523
647

## Slide 65
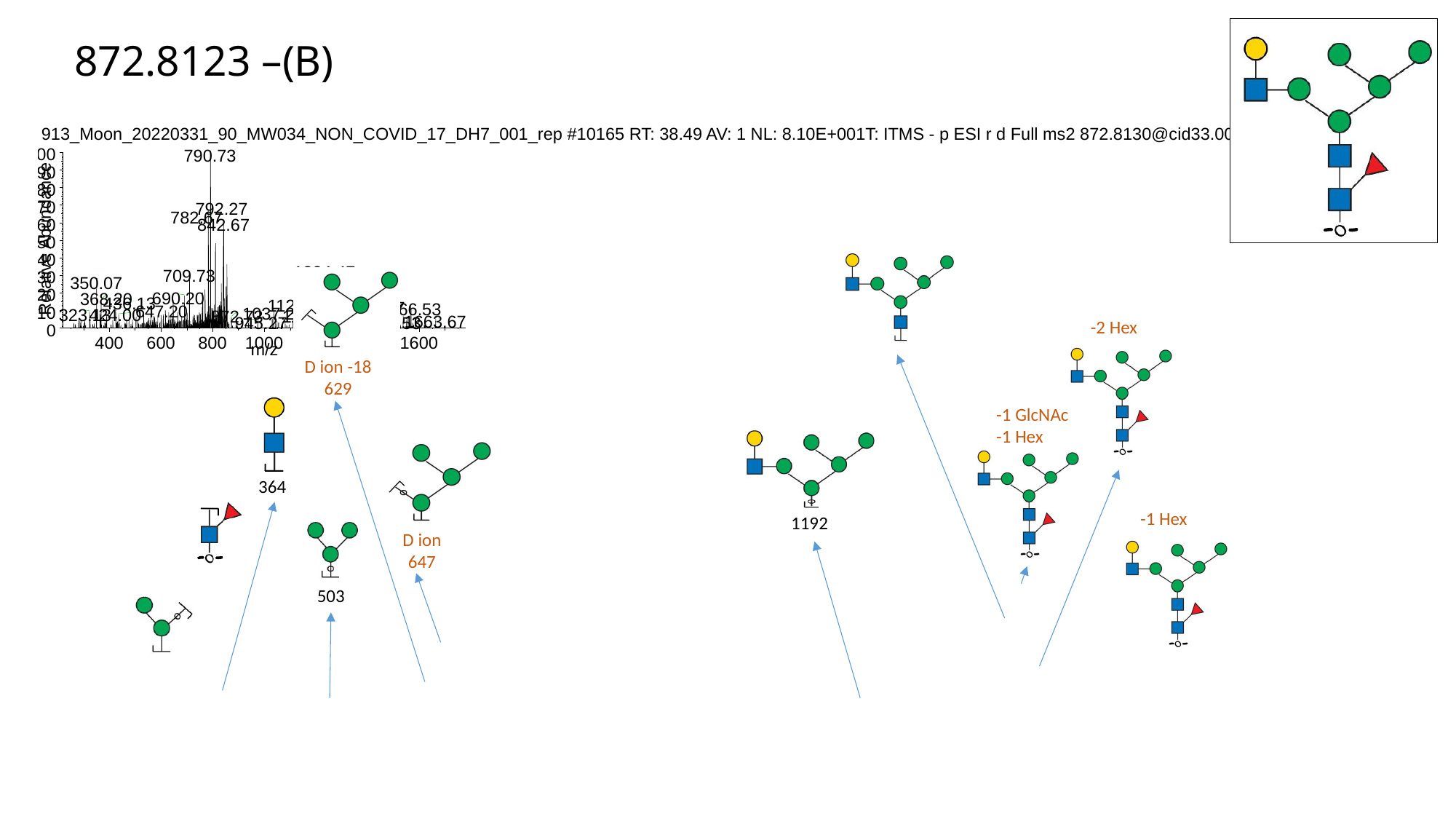

# 872.8123 –(B)
-2 Hex
D ion -18
629
-1 GlcNAc
-1 Hex
364
-1 Hex
1192
D ion
647
503

## Slide 66
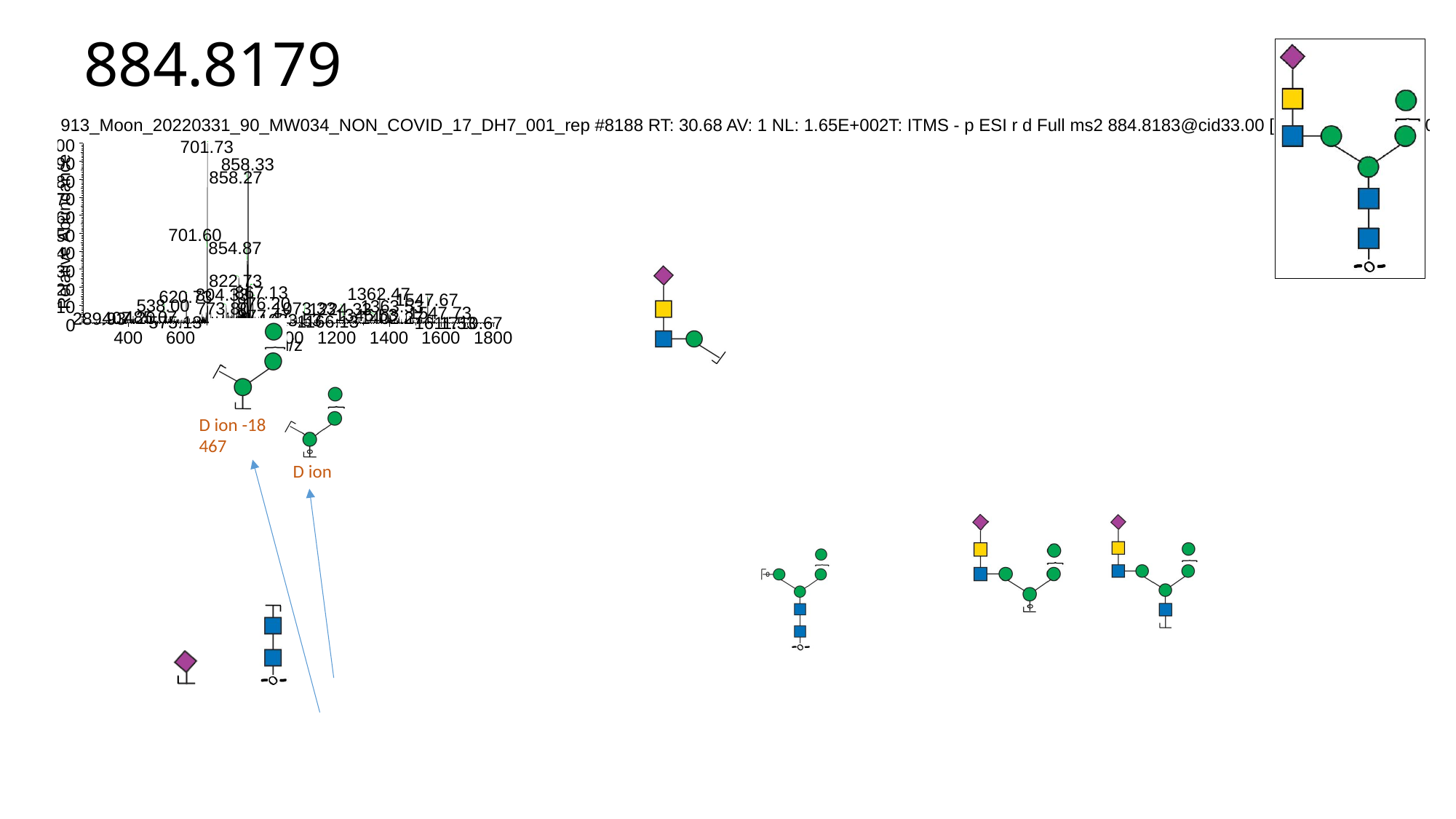

884.8179
#
D ion -18
467
D ion

## Slide 67
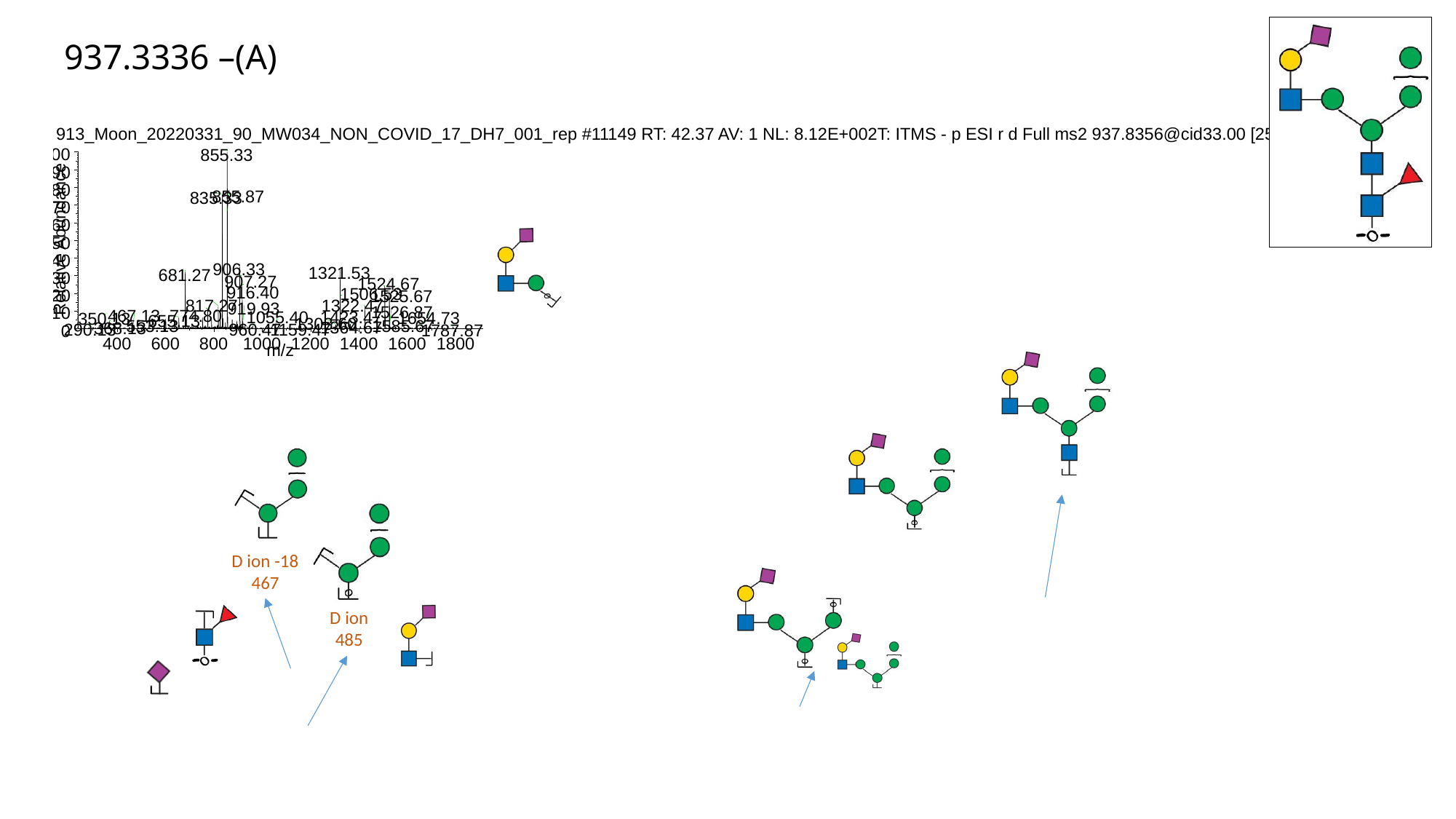

# 937.3336 –(A)
D ion -18
467
D ion
485

## Slide 68
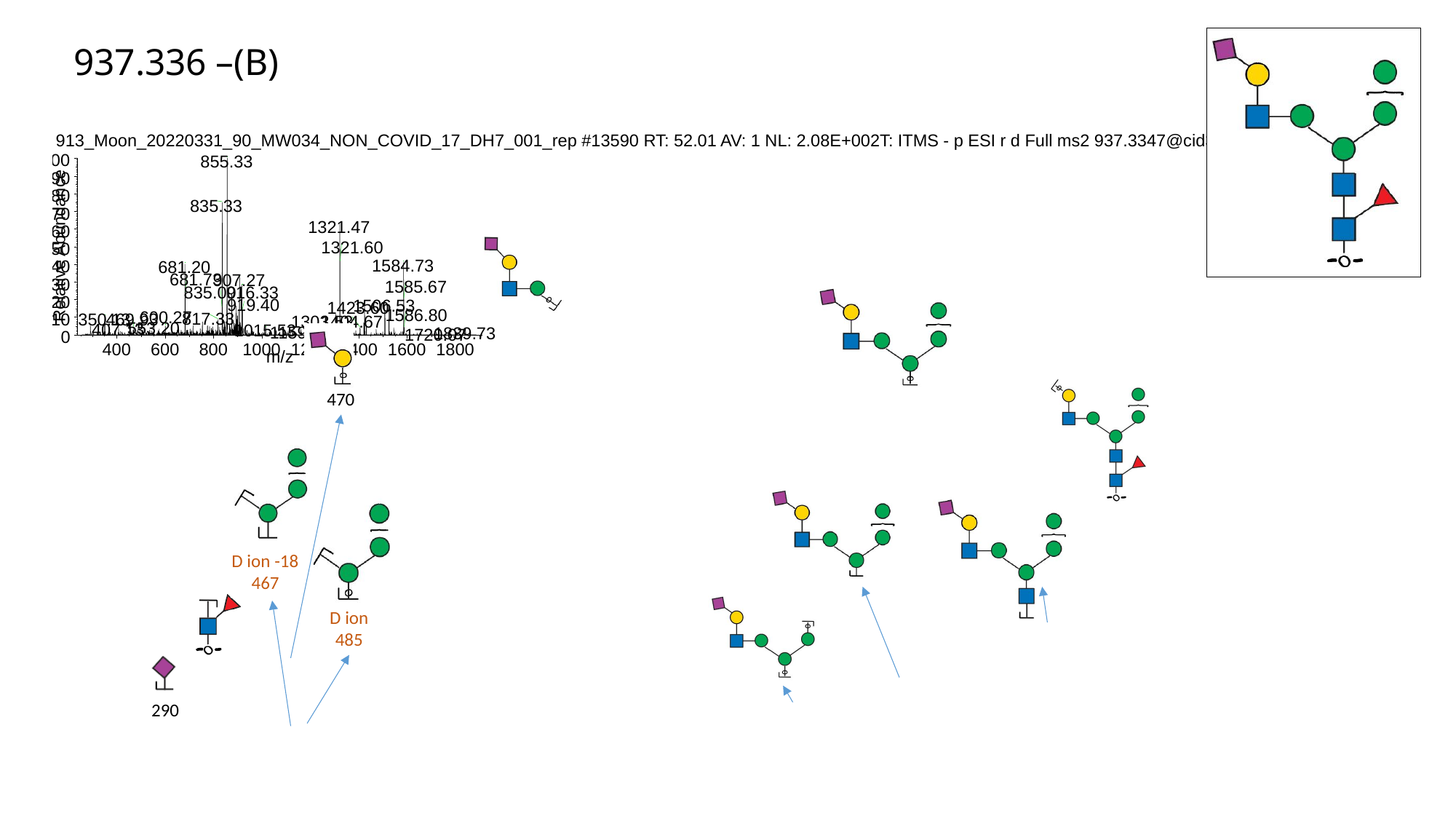

# 937.336 –(B)
470
D ion -18
467
D ion
485
290

## Slide 69
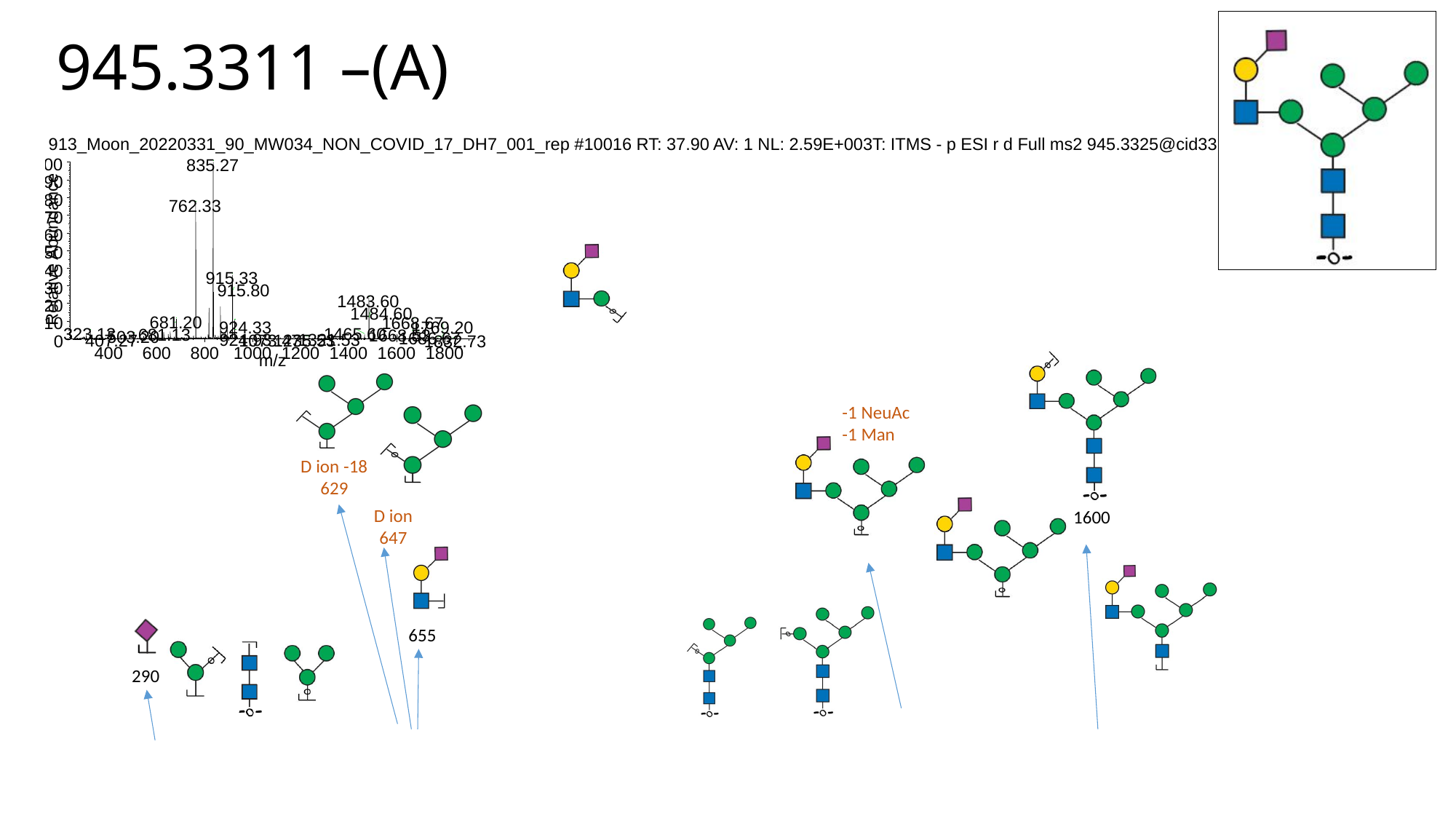

# 945.3311 –(A)
-1 NeuAc
-1 Man
D ion -18
629
D ion
647
1600
655
290

## Slide 70
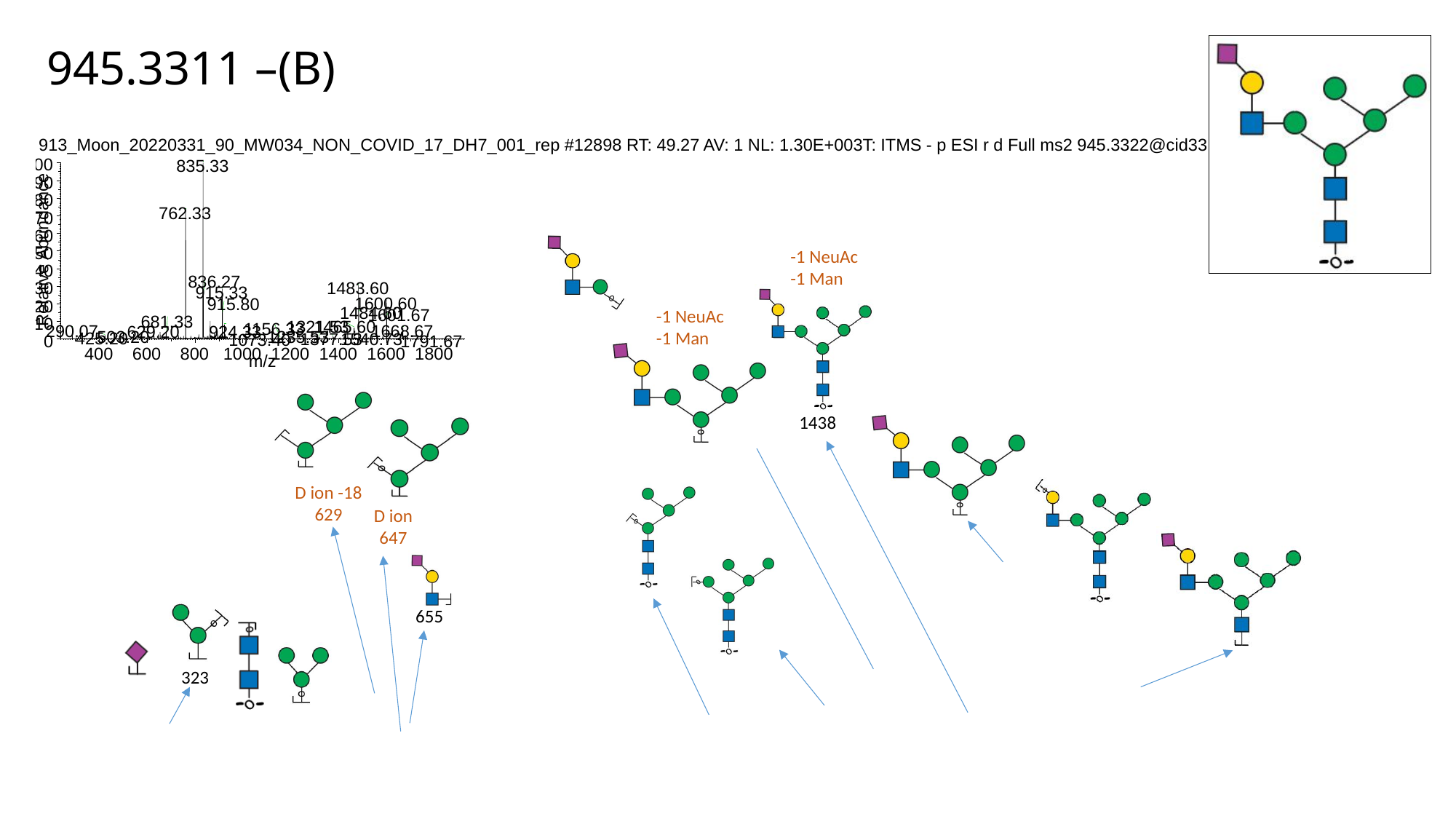

# 945.3311 –(B)
-1 NeuAc
-1 Man
-1 NeuAc
-1 Man
1438
D ion -18
629
D ion
647
655
323

## Slide 71
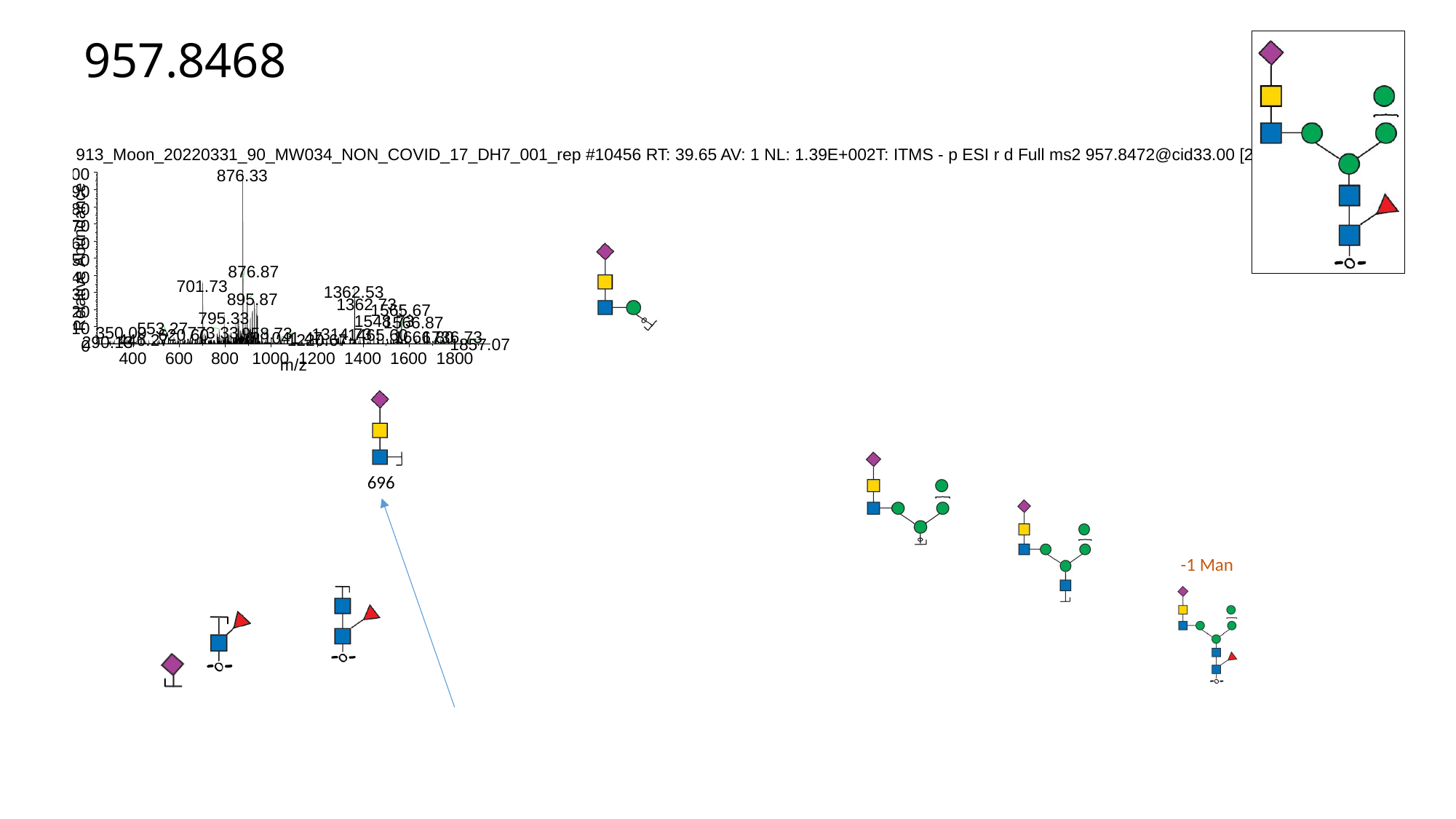

# 957.8468
696
-1 Man

## Slide 72
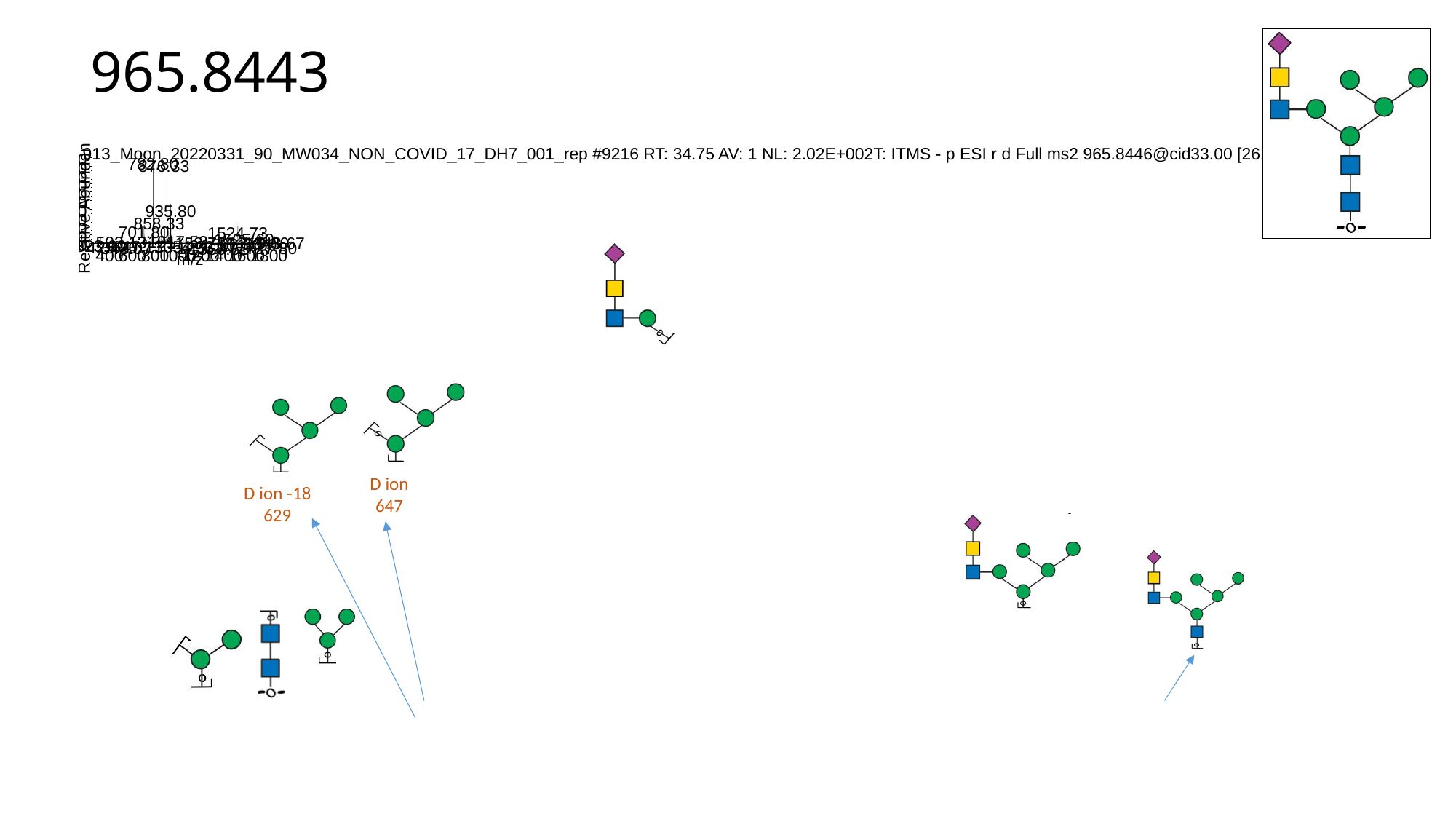

# 965.8443
D ion
647
D ion -18
629

## Slide 73
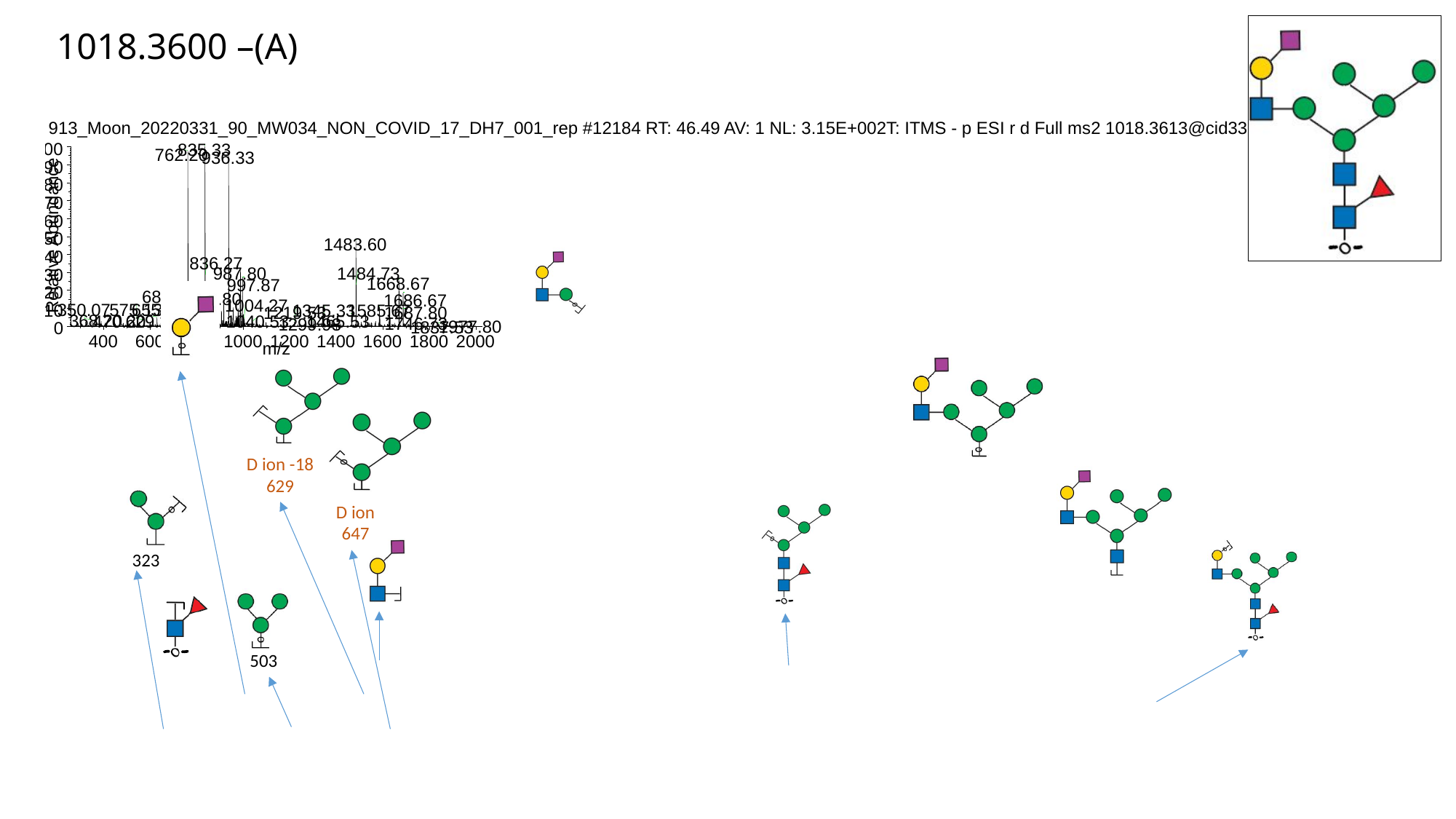

# 1018.3600 –(A)
D ion -18
629
D ion
647
323
503

## Slide 74
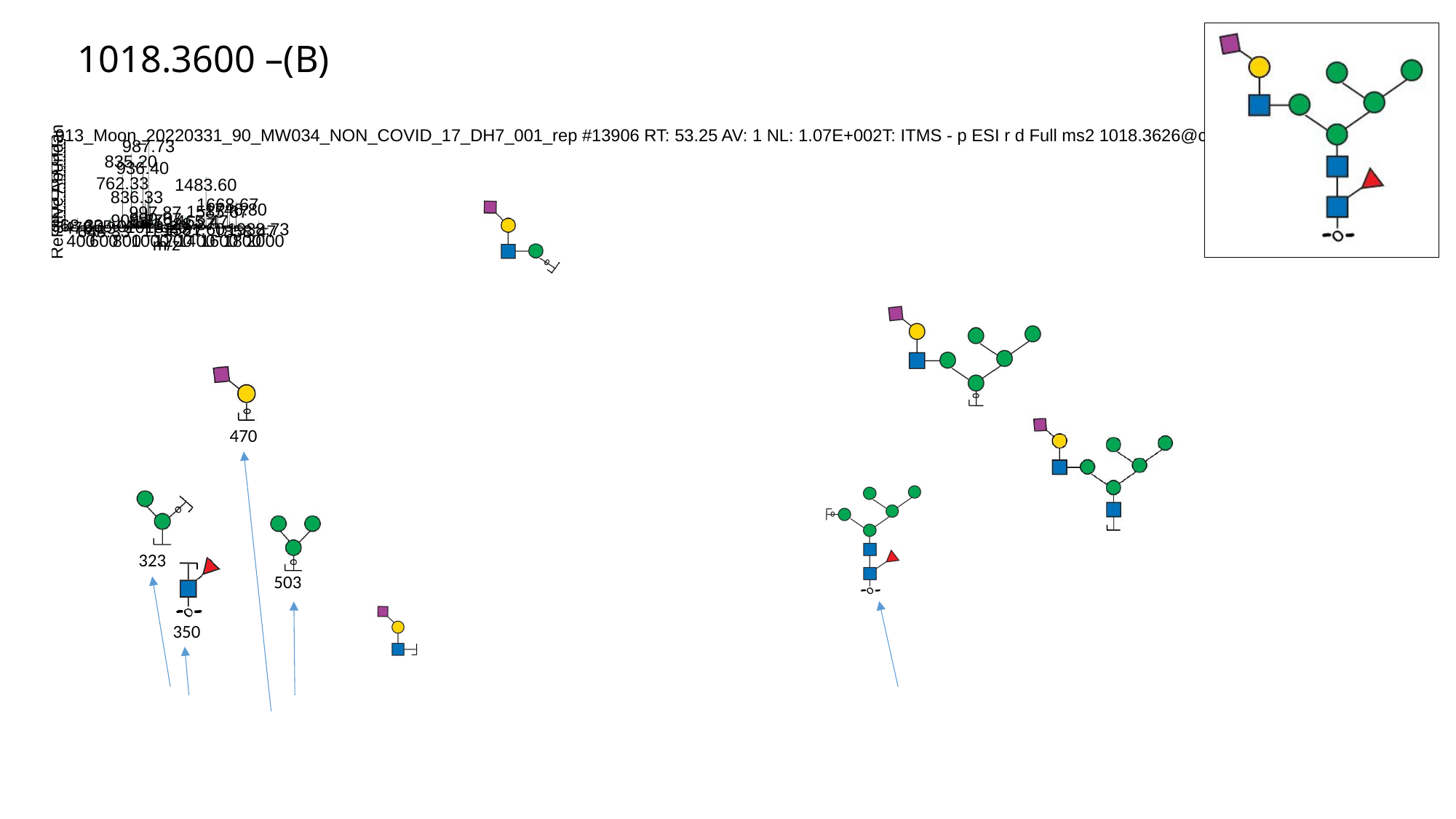

# 1018.3600 –(B)
470
323
503
350

## Slide 75
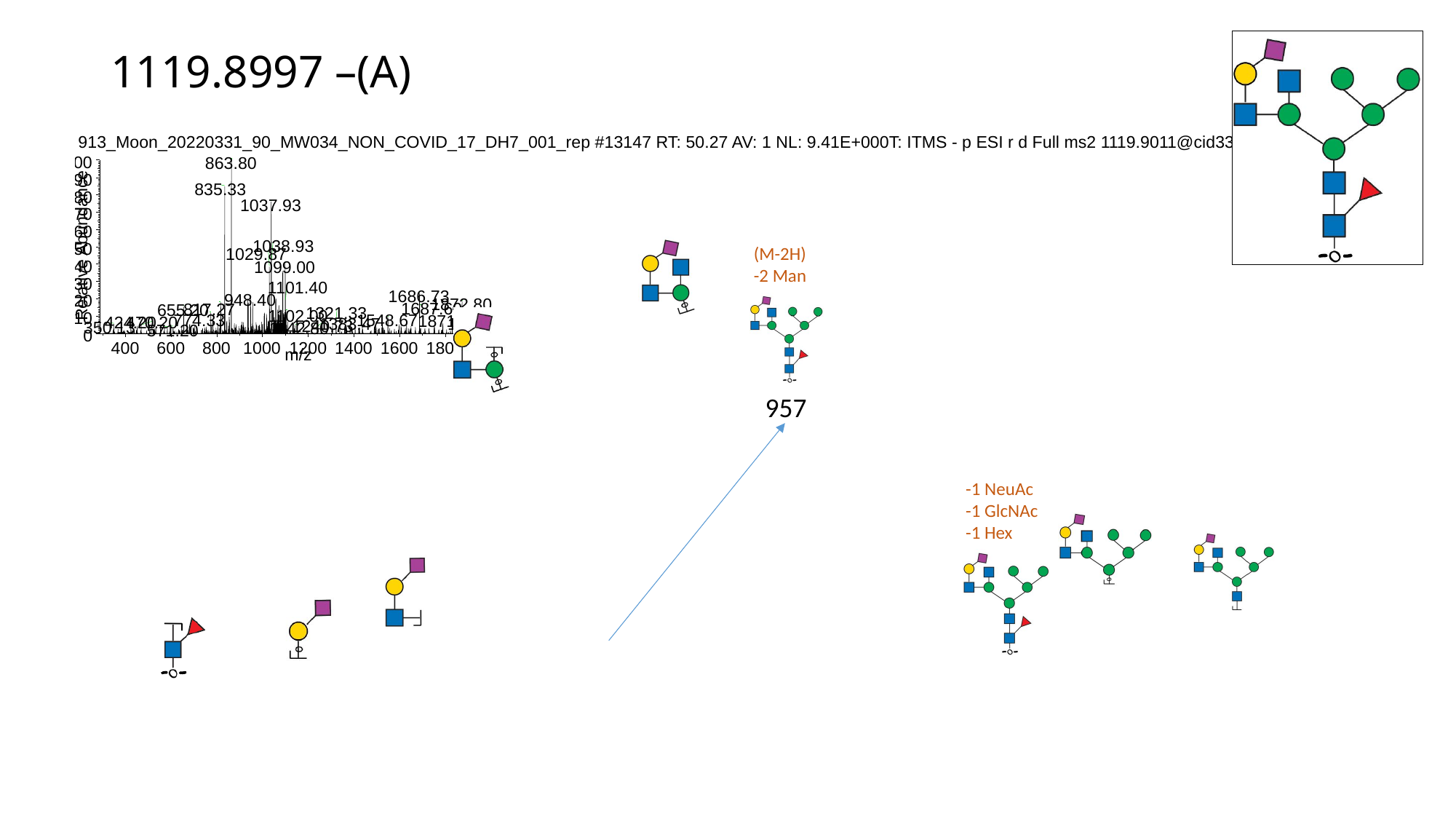

# 1119.8997 –(A)
(M-2H)
-2 Man
957
-1 NeuAc
-1 GlcNAc
-1 Hex

## Slide 76
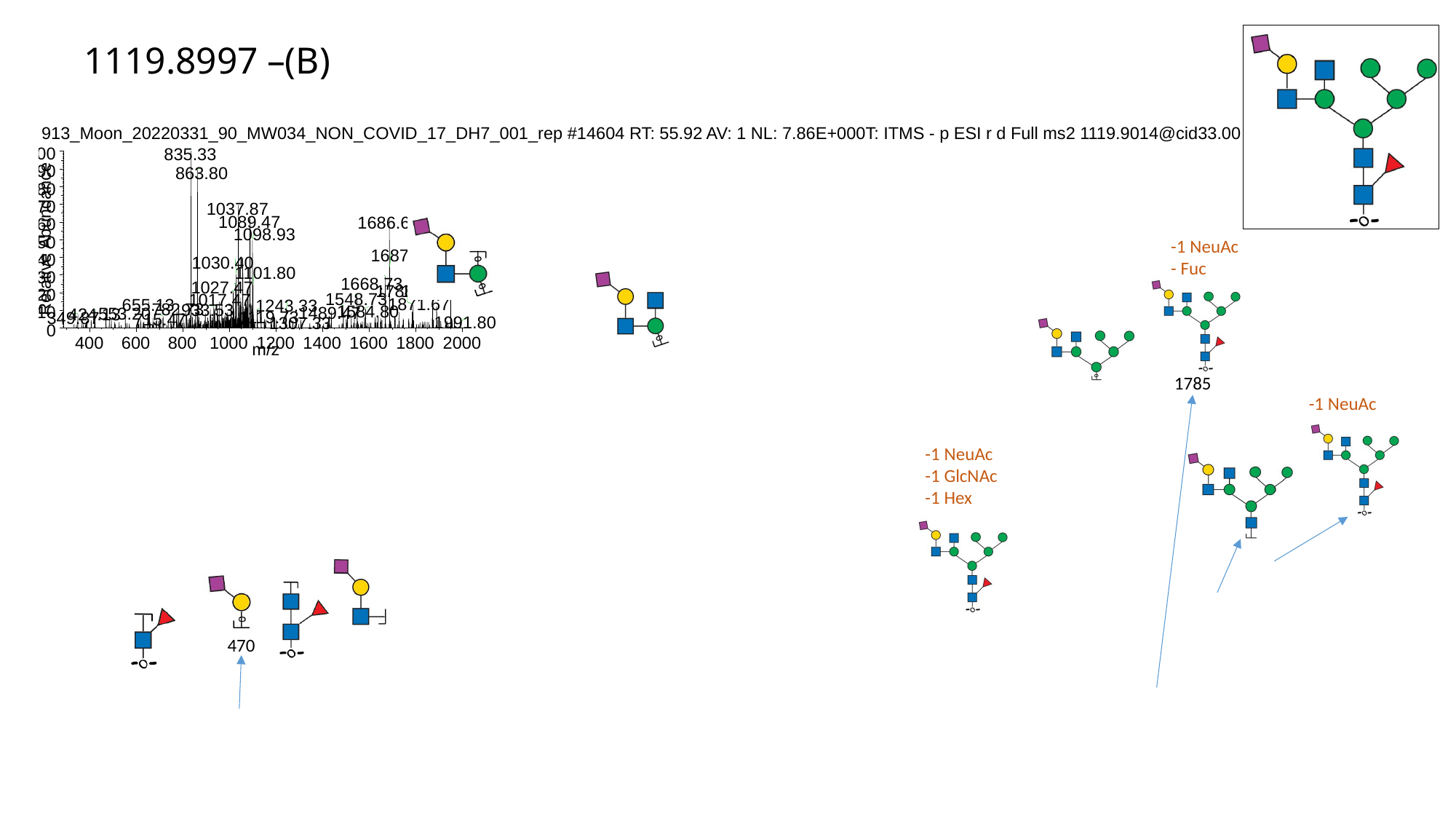

# 1119.8997 –(B)
-1 NeuAc
- Fuc
1785
-1 NeuAc
-1 NeuAc
-1 GlcNAc
-1 Hex
470

## Slide 77
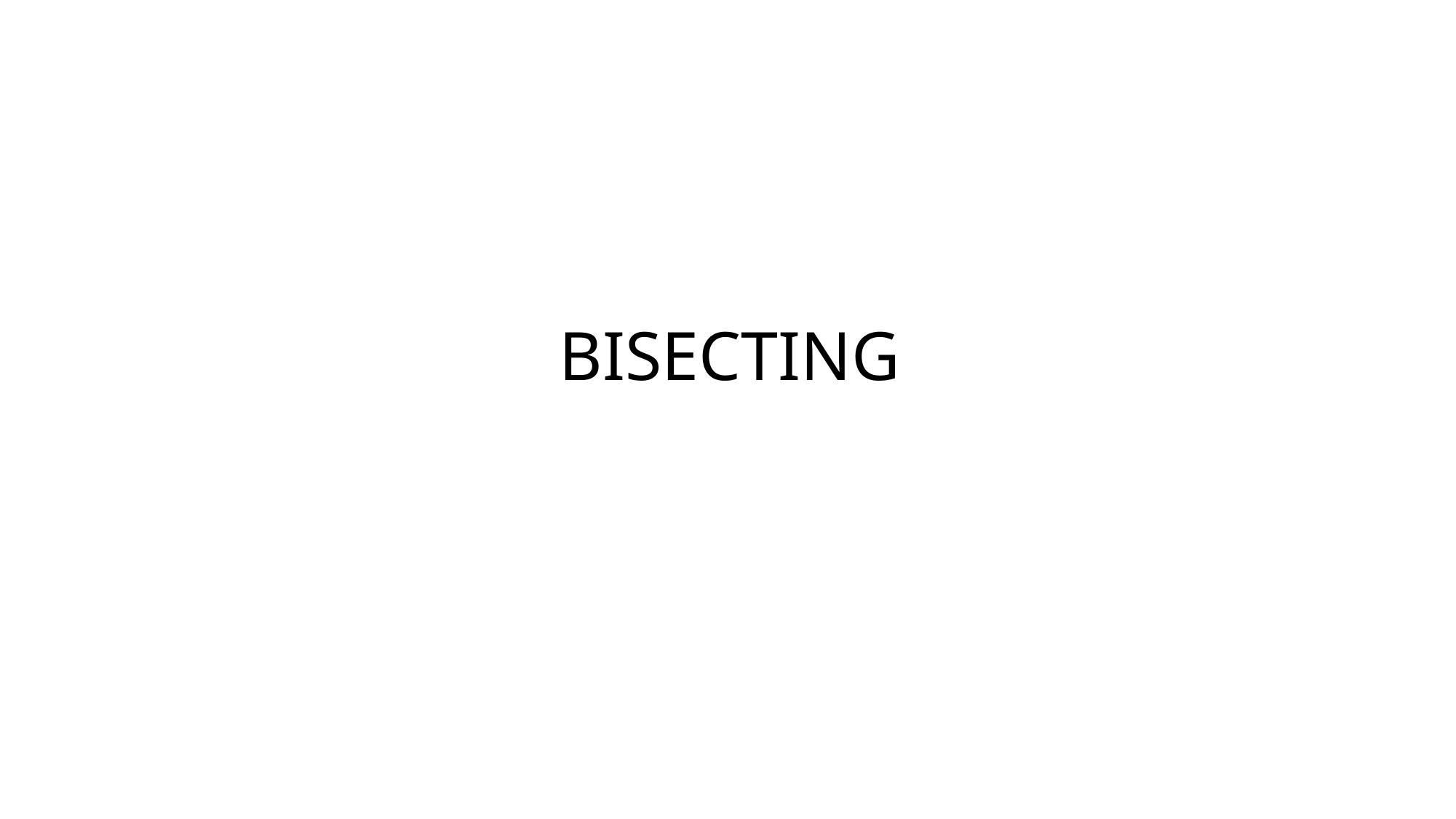

# BISECTING

## Slide 78
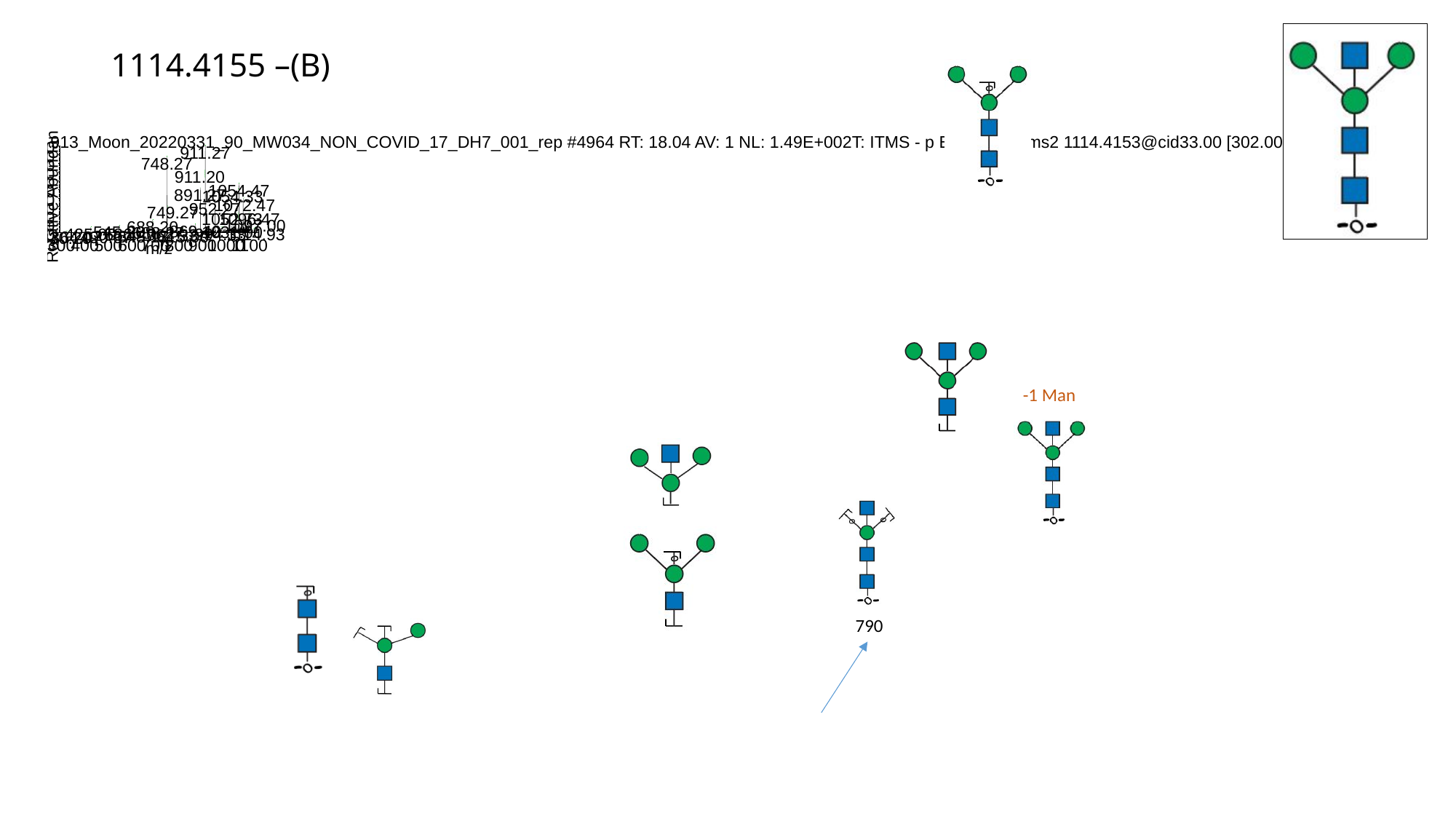

# 1114.4155 –(B)
-1 Man
790

## Slide 79
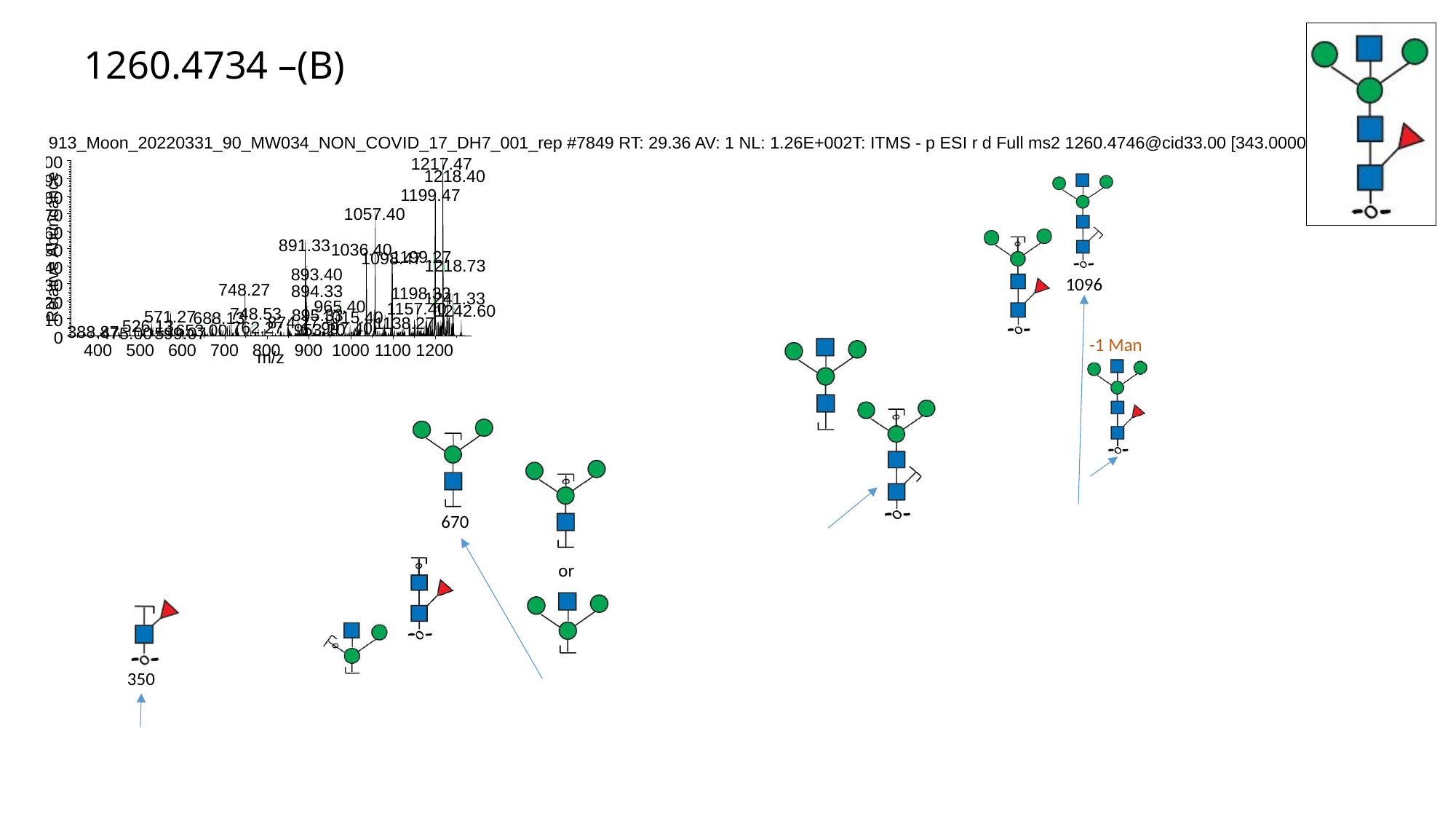

# 1260.4734 –(B)
1096
-1 Man
670
or
350

## Slide 80
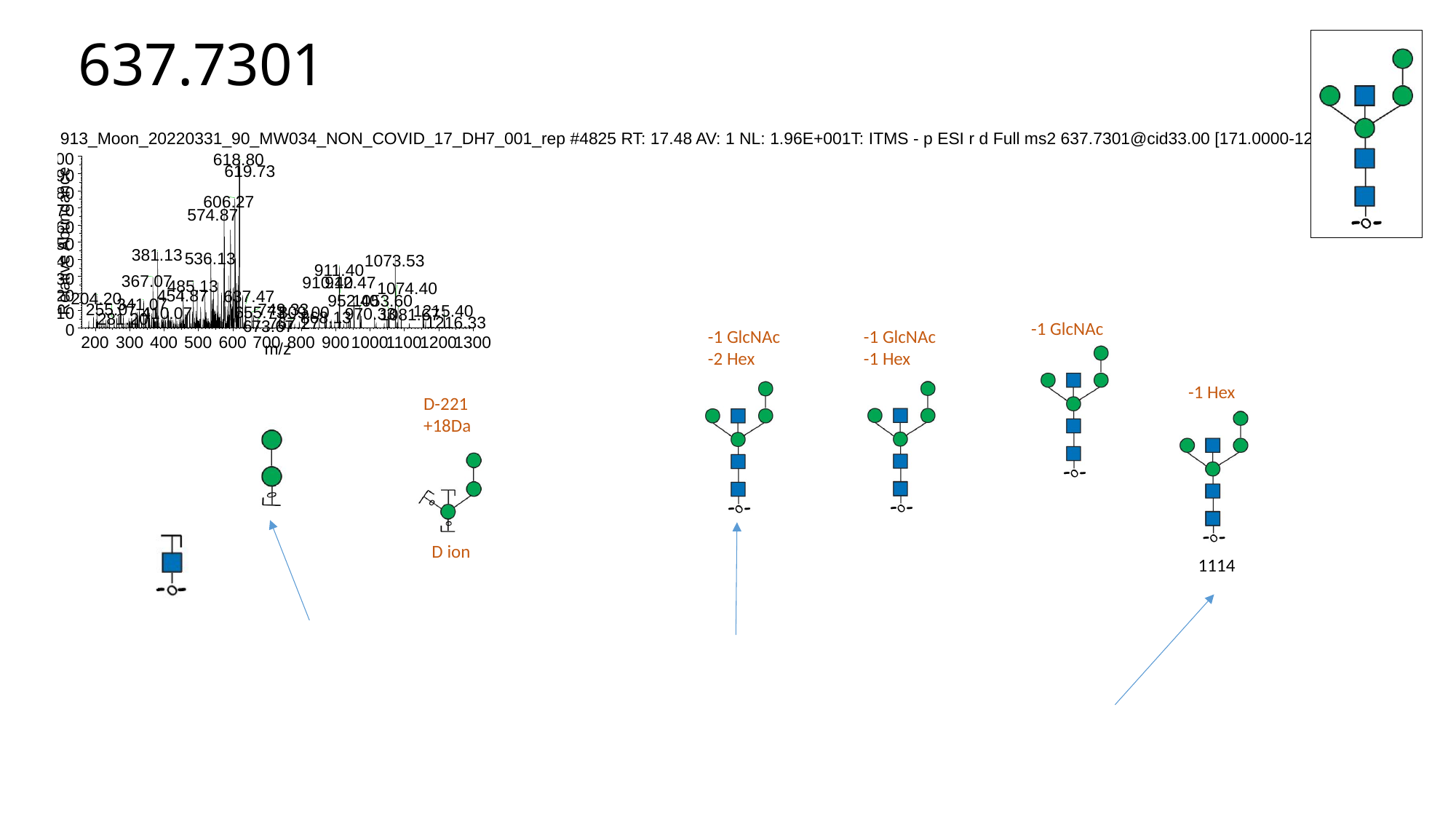

# 637.7301
-1 GlcNAc
-1 GlcNAc
-2 Hex
-1 GlcNAc
-1 Hex
-1 Hex
D-221
+18Da
D ion
1114

## Slide 81
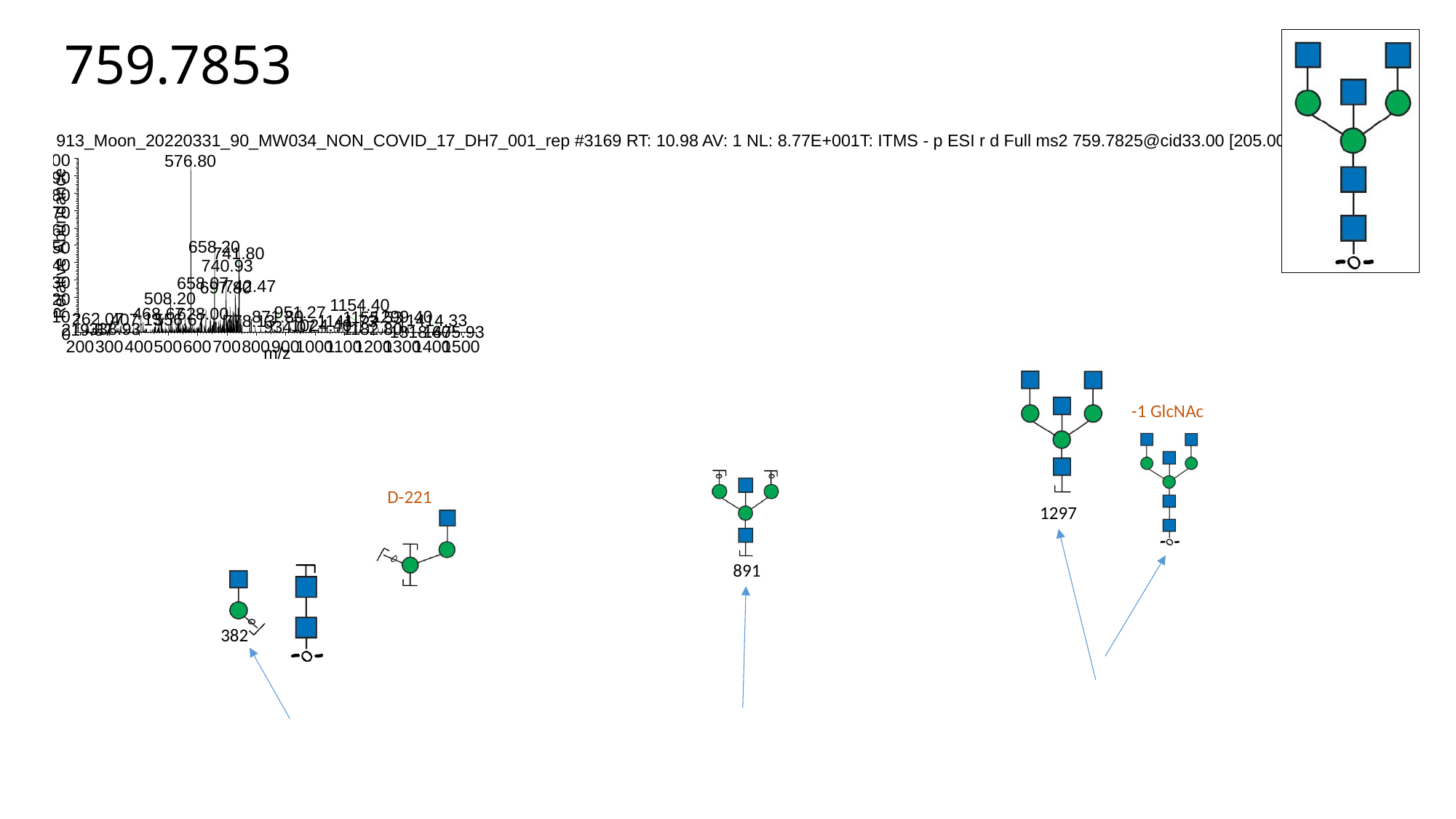

# 759.7853
-1 GlcNAc
D-221
1297
891
382

## Slide 82
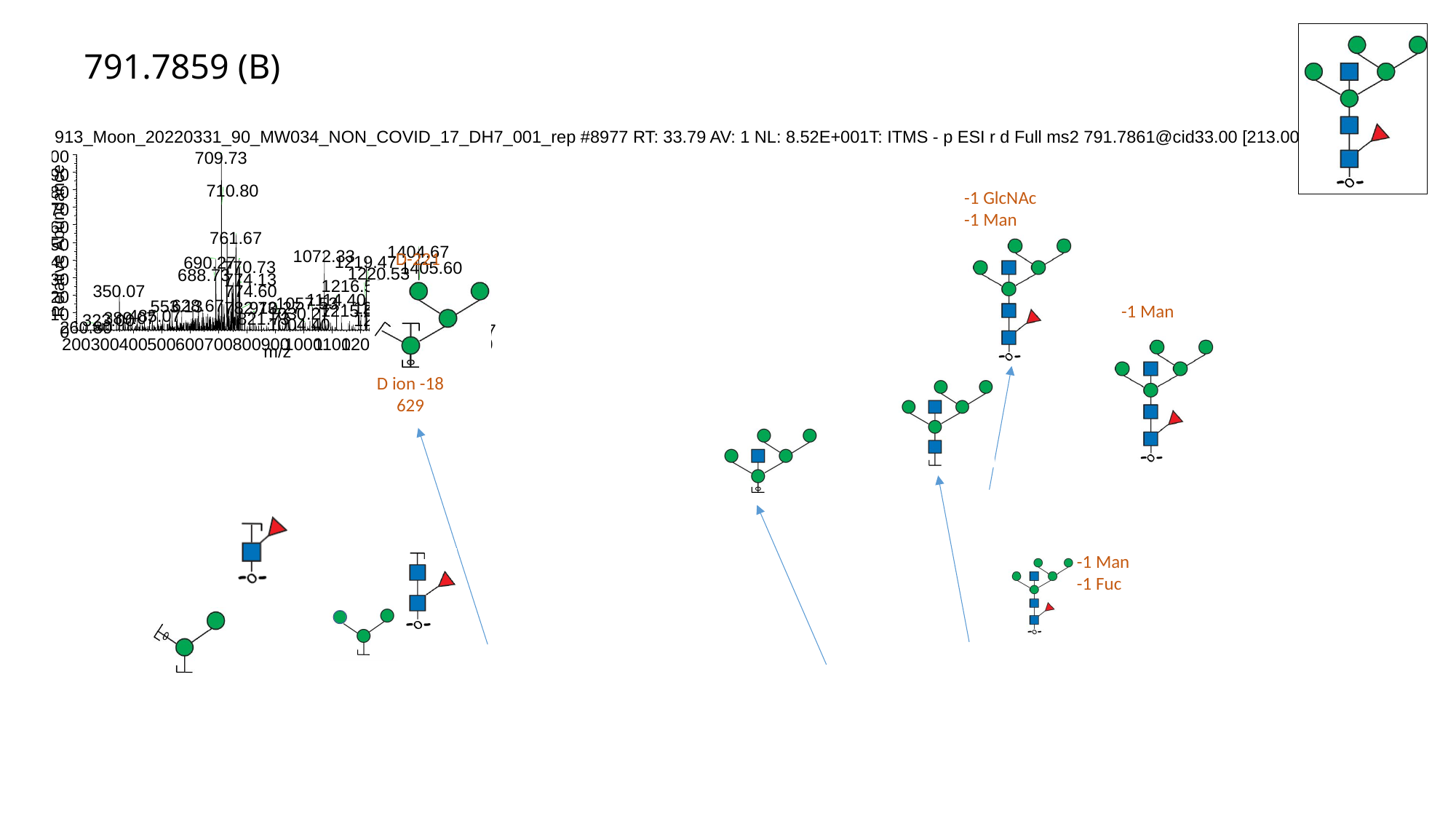

# 791.7859 (B)
-1 GlcNAc
-1 Man
D-221
-1 Man
D ion -18
629
-1 Man
-1 Fuc

## Slide 83
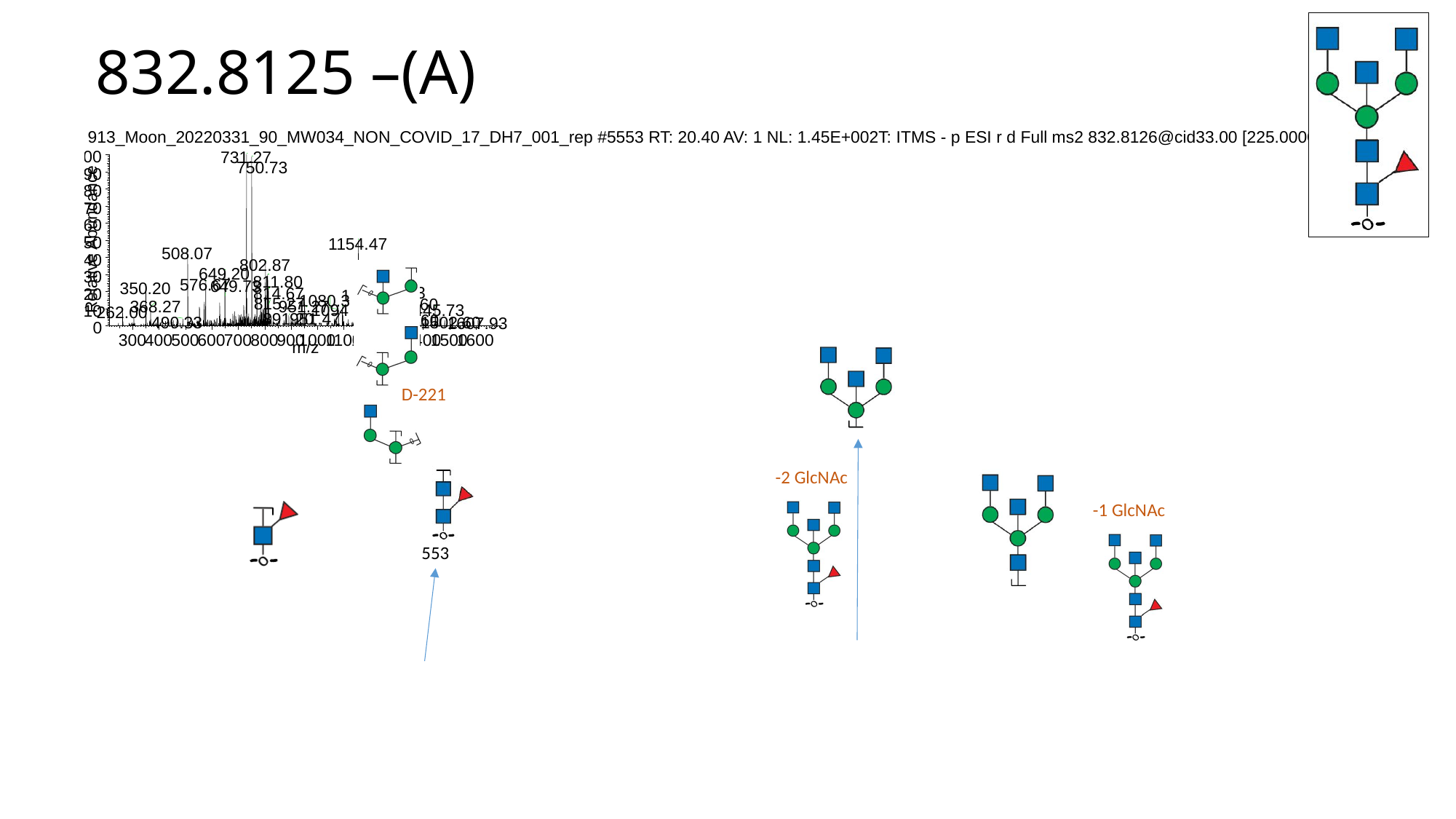

# 832.8125 –(A)
D-221
-2 GlcNAc
-1 GlcNAc
553

## Slide 84
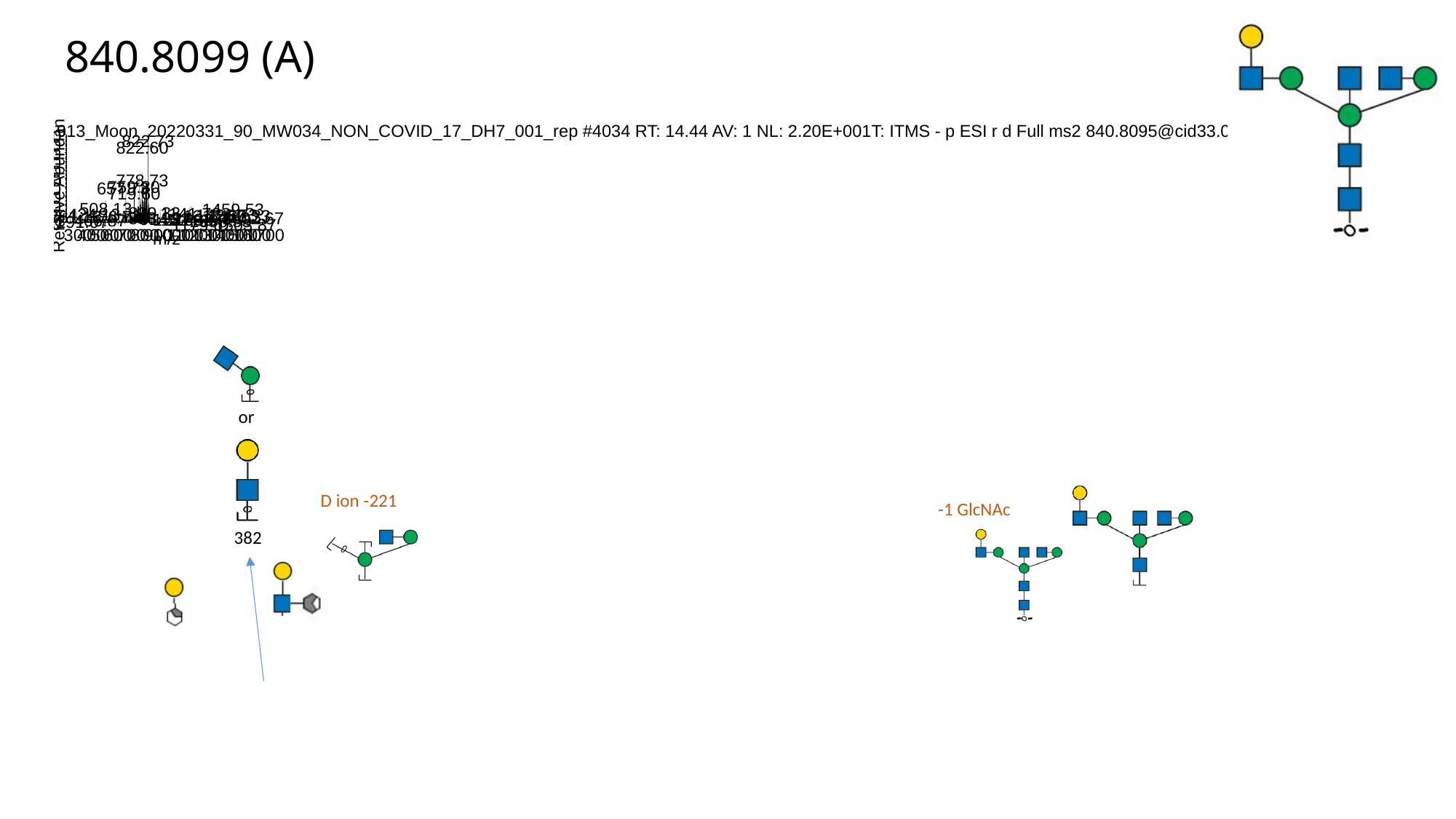

# 840.8099 (A)
or
D ion -221
-1 GlcNAc
382

## Slide 85
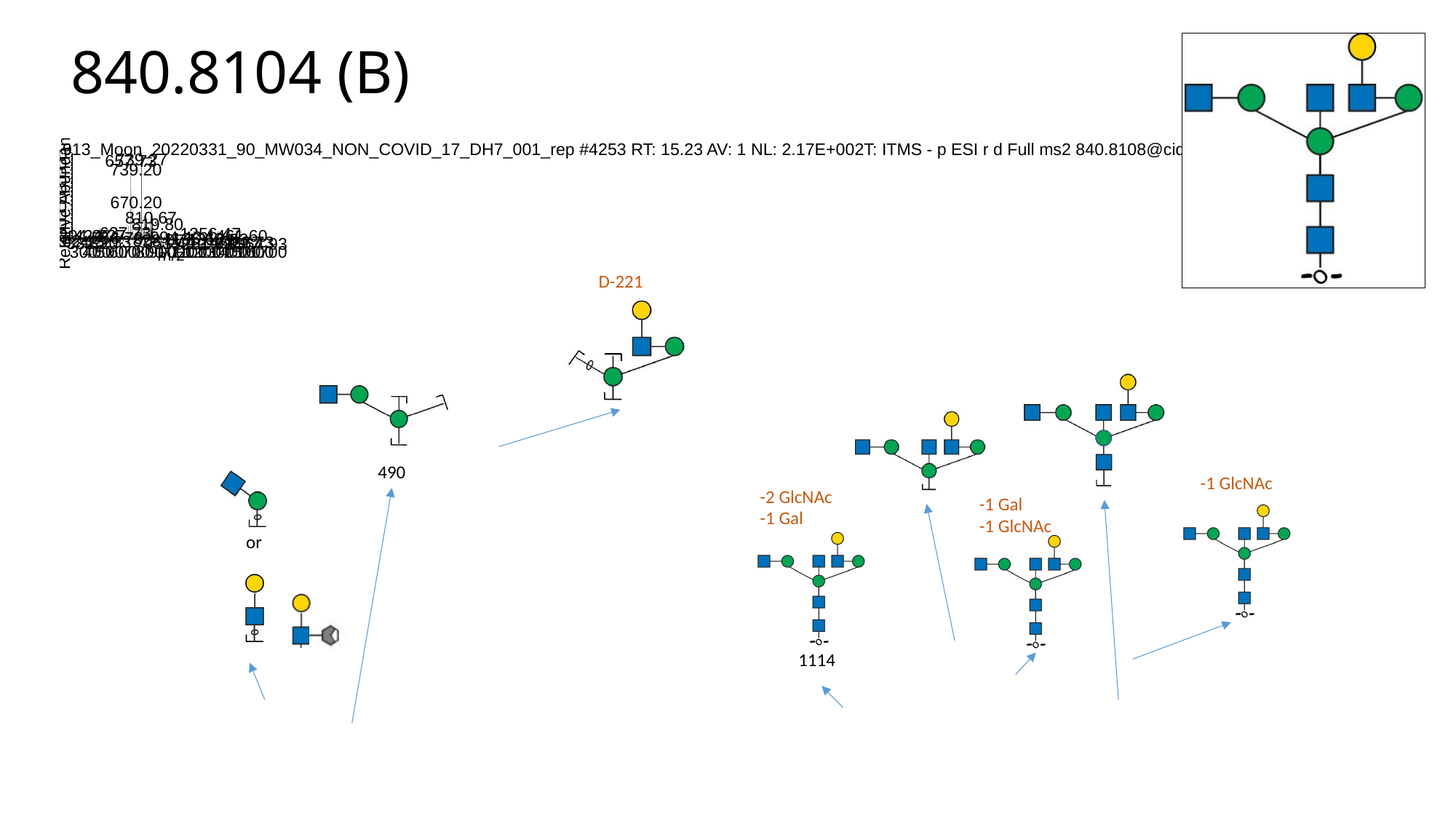

# 840.8104 (B)
D-221
490
-1 GlcNAc
-2 GlcNAc
-1 Gal
-1 Gal
-1 GlcNAc
or
1114

## Slide 86
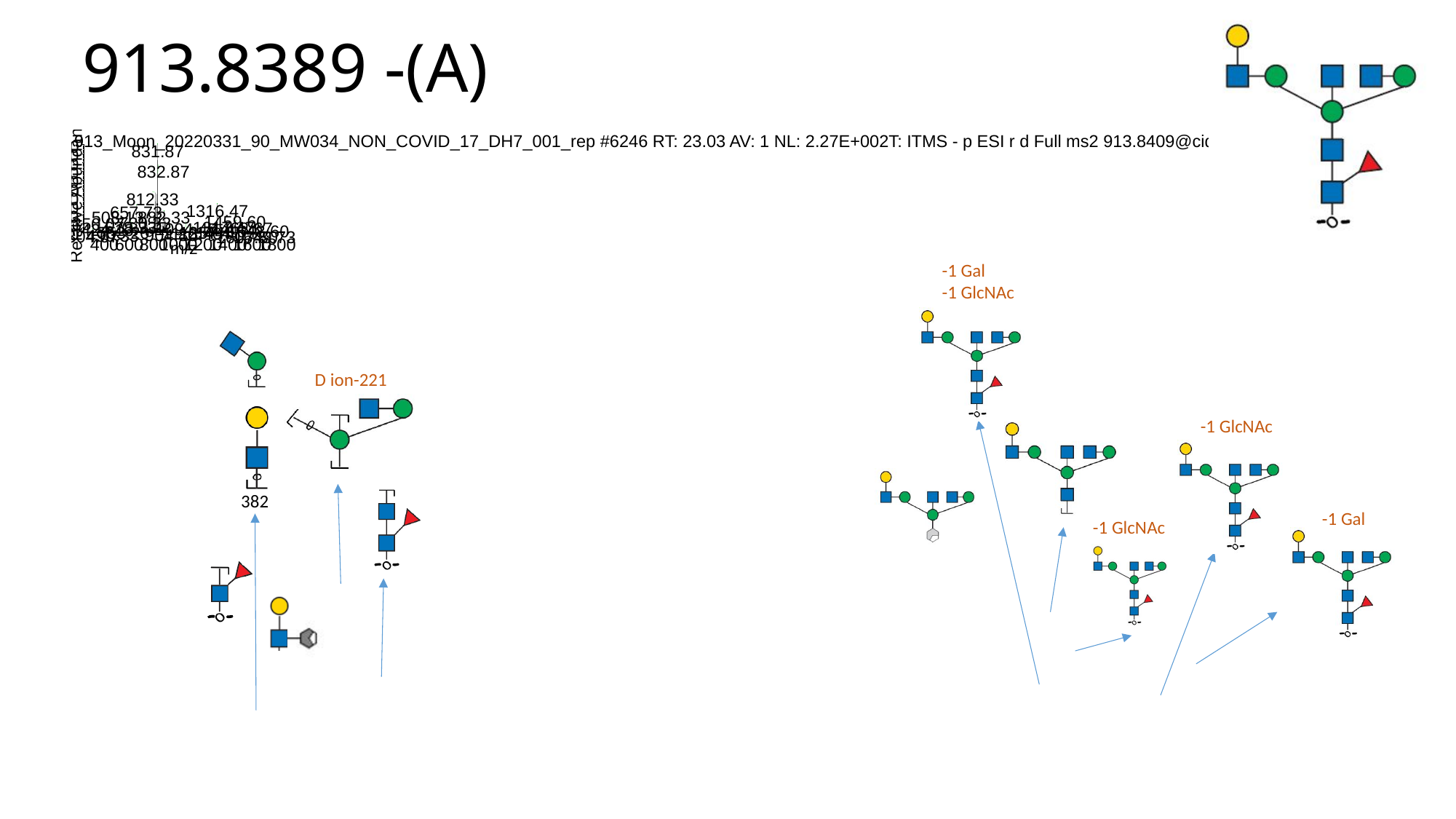

# 913.8389 -(A)
-1 Gal
-1 GlcNAc
D ion-221
-1 GlcNAc
382
-1 Gal
-1 GlcNAc

## Slide 87
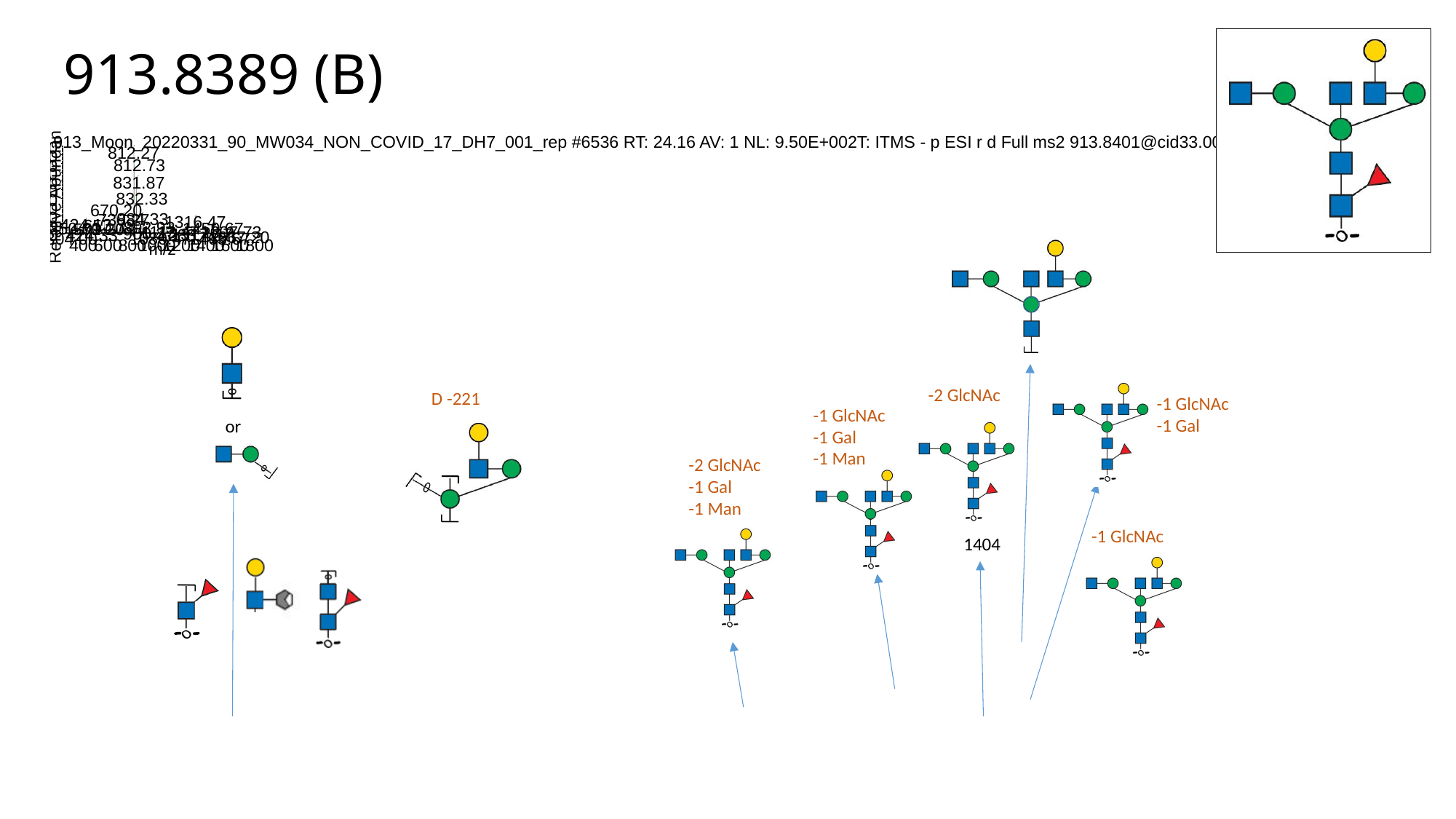

# 913.8389 (B)
-2 GlcNAc
D -221
-1 GlcNAc
-1 Gal
-1 GlcNAc
-1 Gal
-1 Man
or
-2 GlcNAc
-1 Gal
-1 Man
-1 GlcNAc
1404

## Slide 88
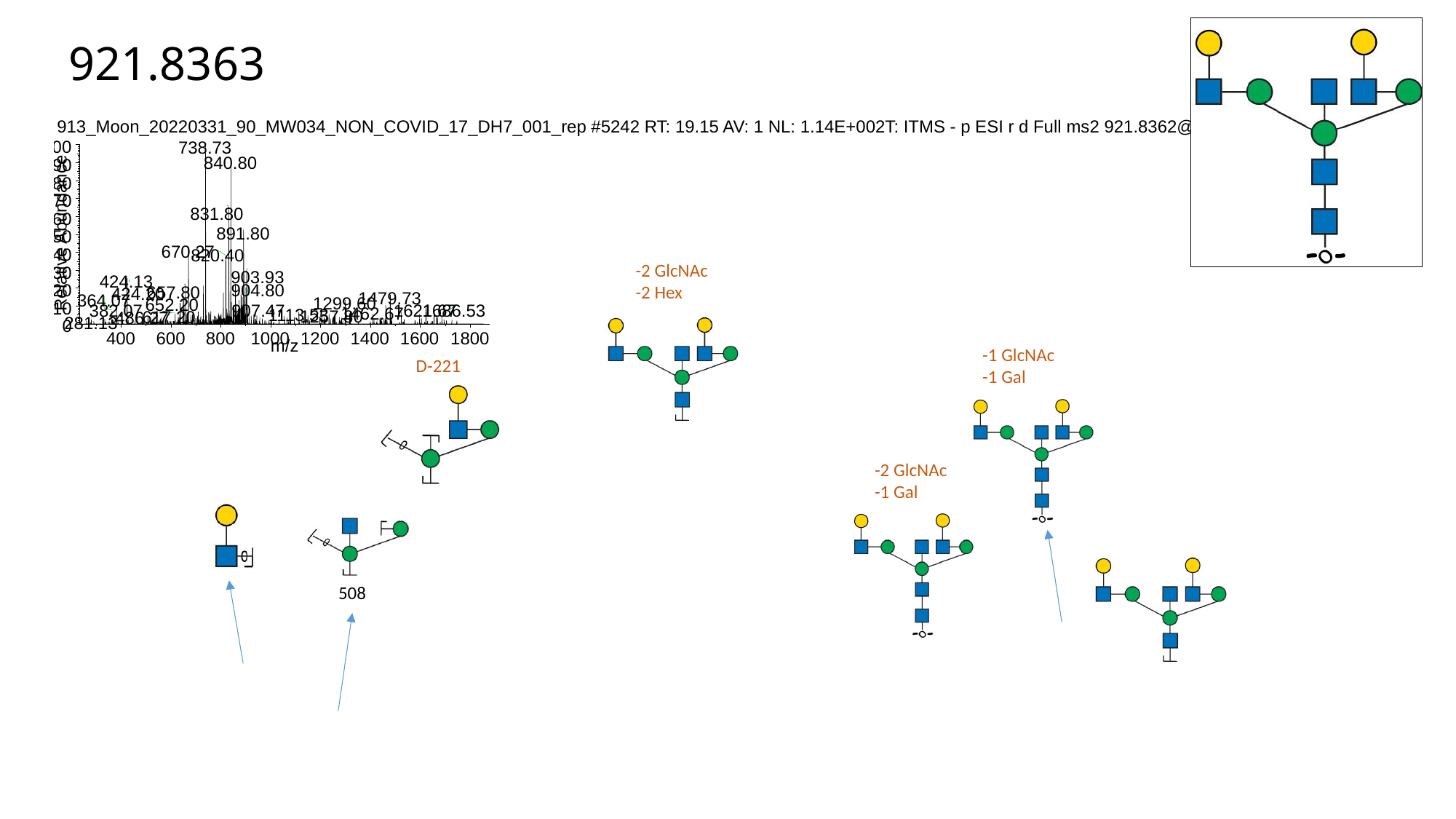

# 921.8363
-2 GlcNAc
-2 Hex
-1 GlcNAc
-1 Gal
D-221
-2 GlcNAc
-1 Gal
508

## Slide 89
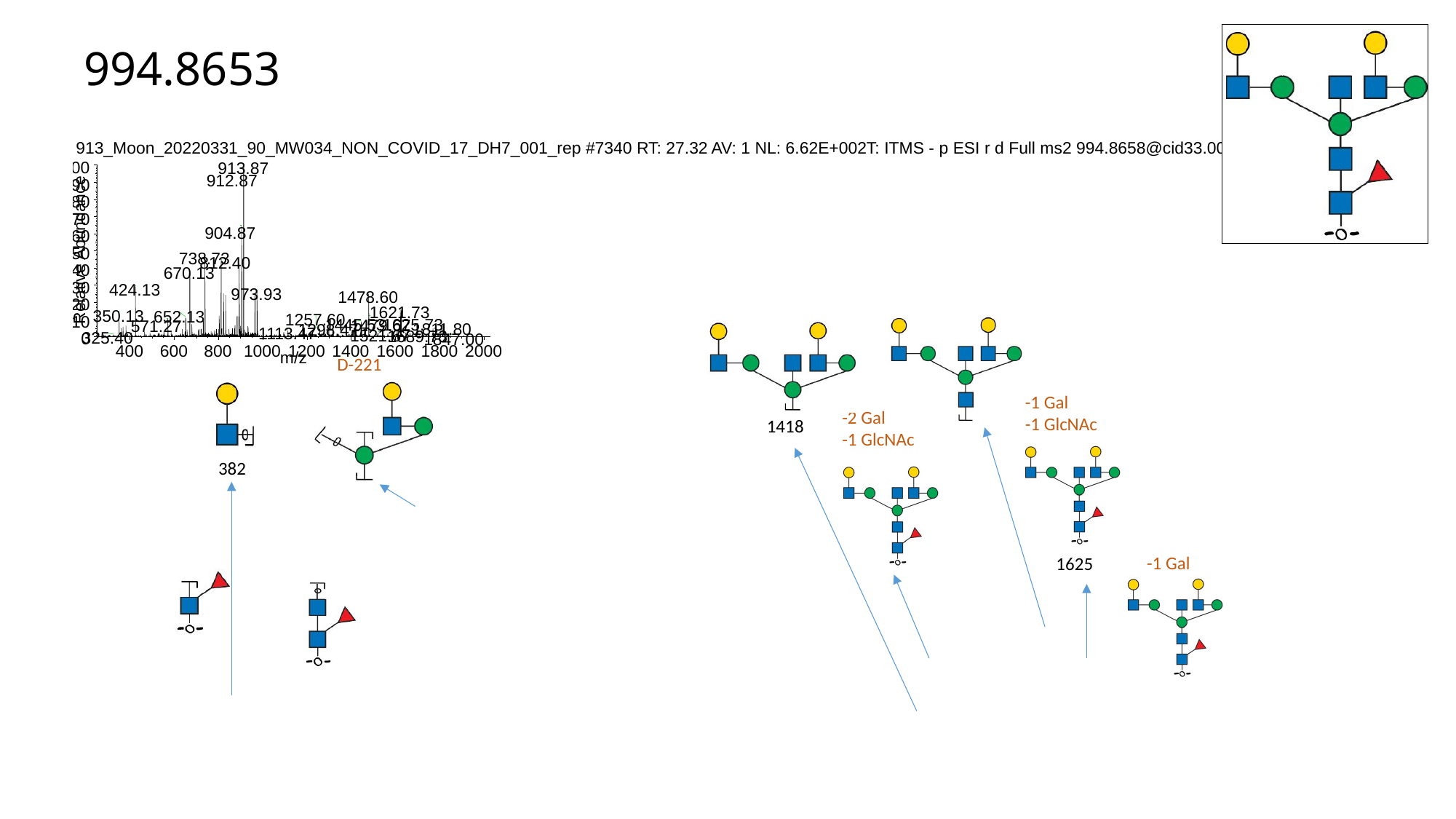

# 994.8653
D-221
-1 Gal
-1 GlcNAc
-2 Gal
-1 GlcNAc
1418
382
-1 Gal
1625

## Slide 90
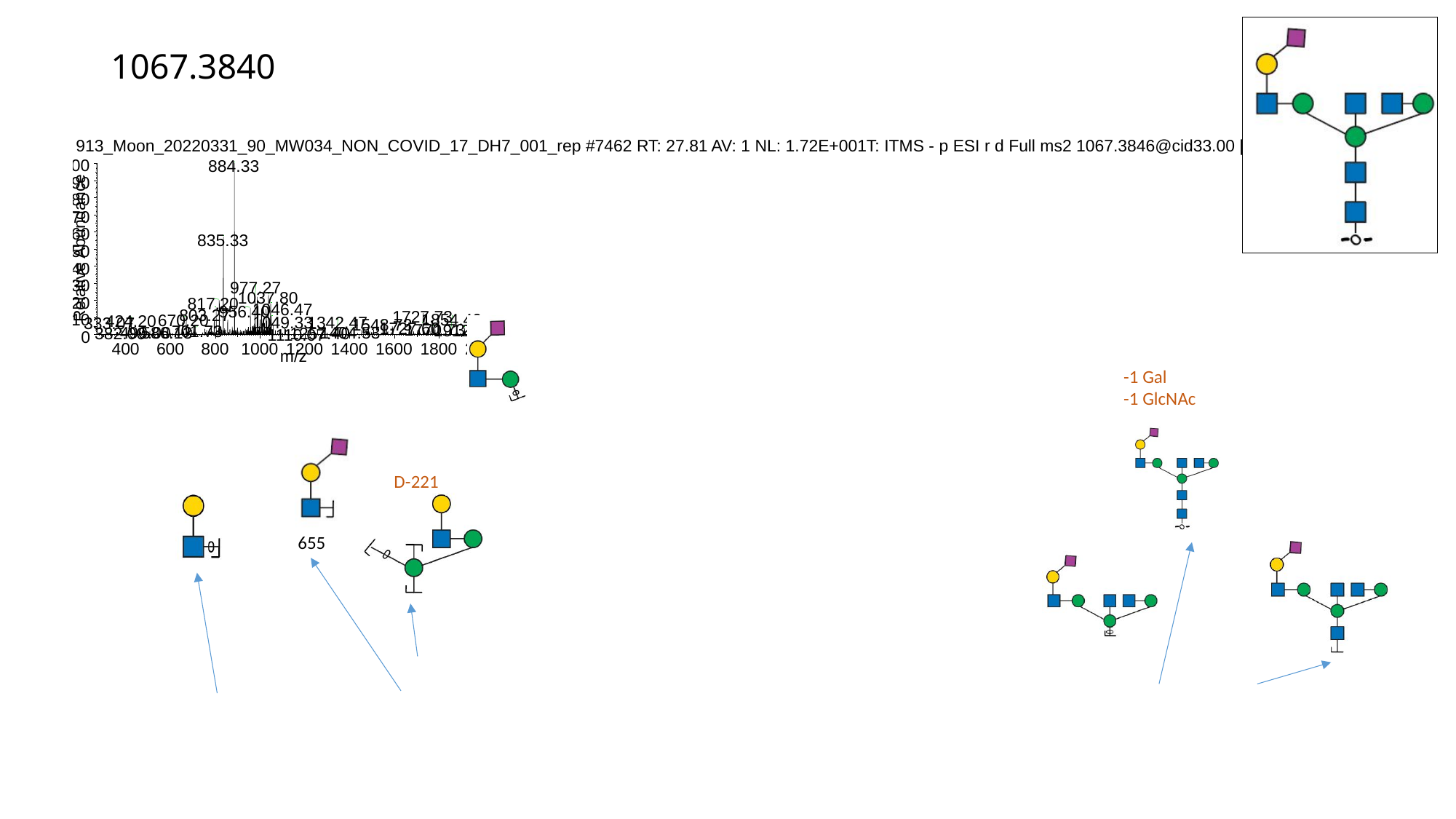

# 1067.3840
-1 Gal
-1 GlcNAc
D-221
655

## Slide 91
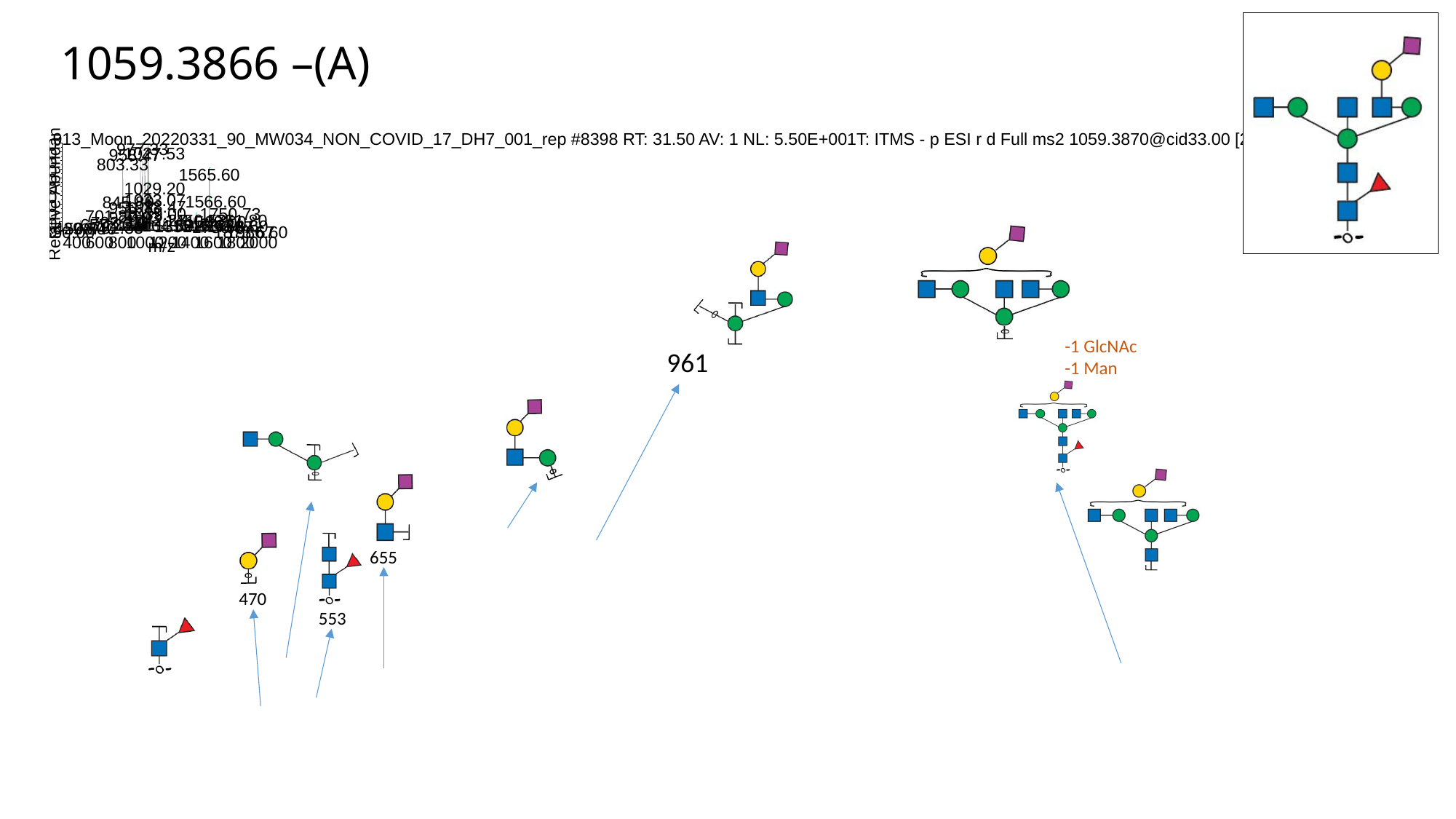

# 1059.3866 –(A)
-1 GlcNAc
-1 Man
961
655
470
553

## Slide 92
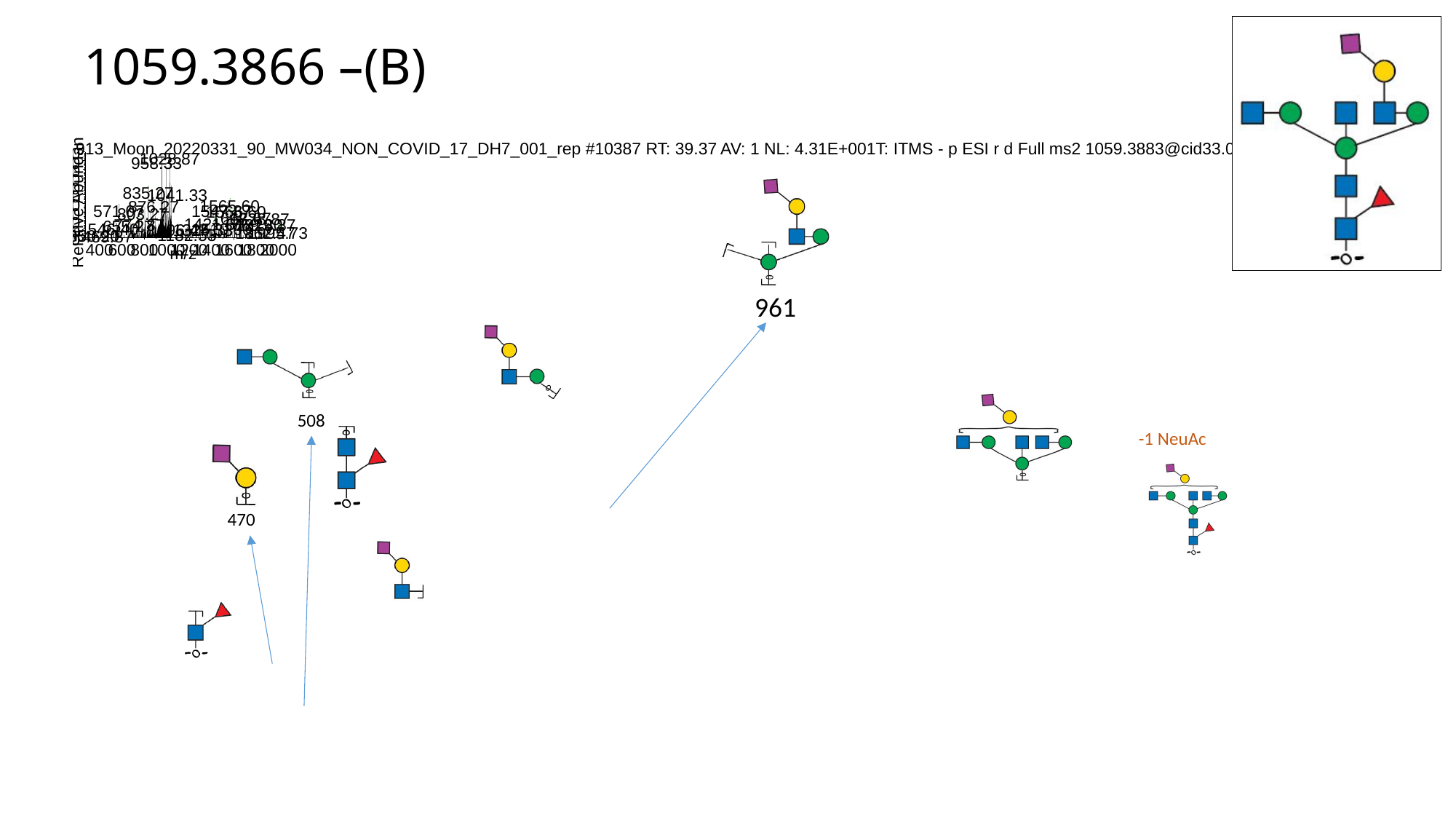

# 1059.3866 –(B)
961
508
-1 NeuAc
470

## Slide 93
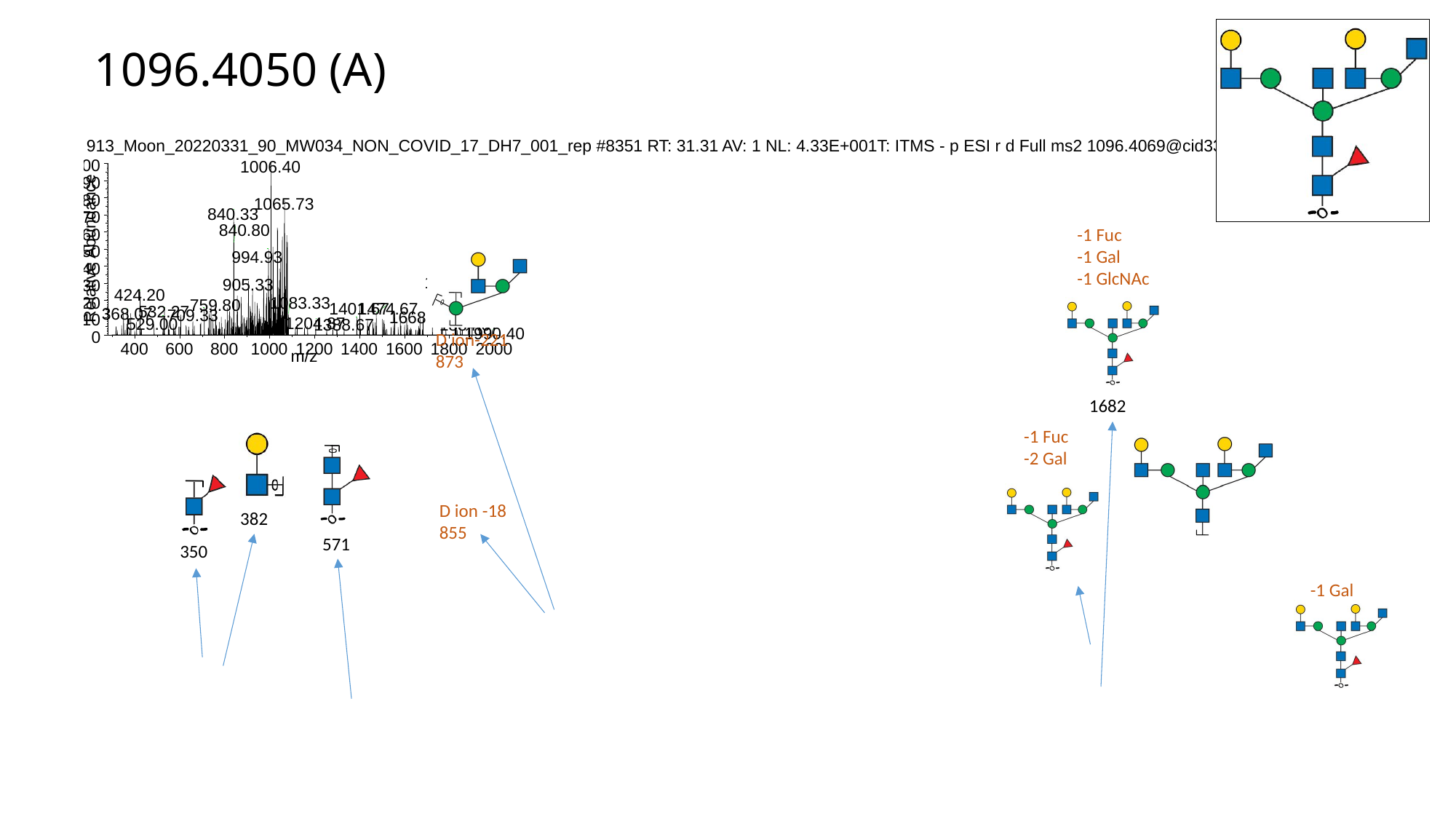

# 1096.4050 (A)
-1 Fuc
-1 Gal
-1 GlcNAc
D ion-221
873
1682
-1 Fuc
-2 Gal
D ion -18
855
382
571
350
-1 Gal

## Slide 94
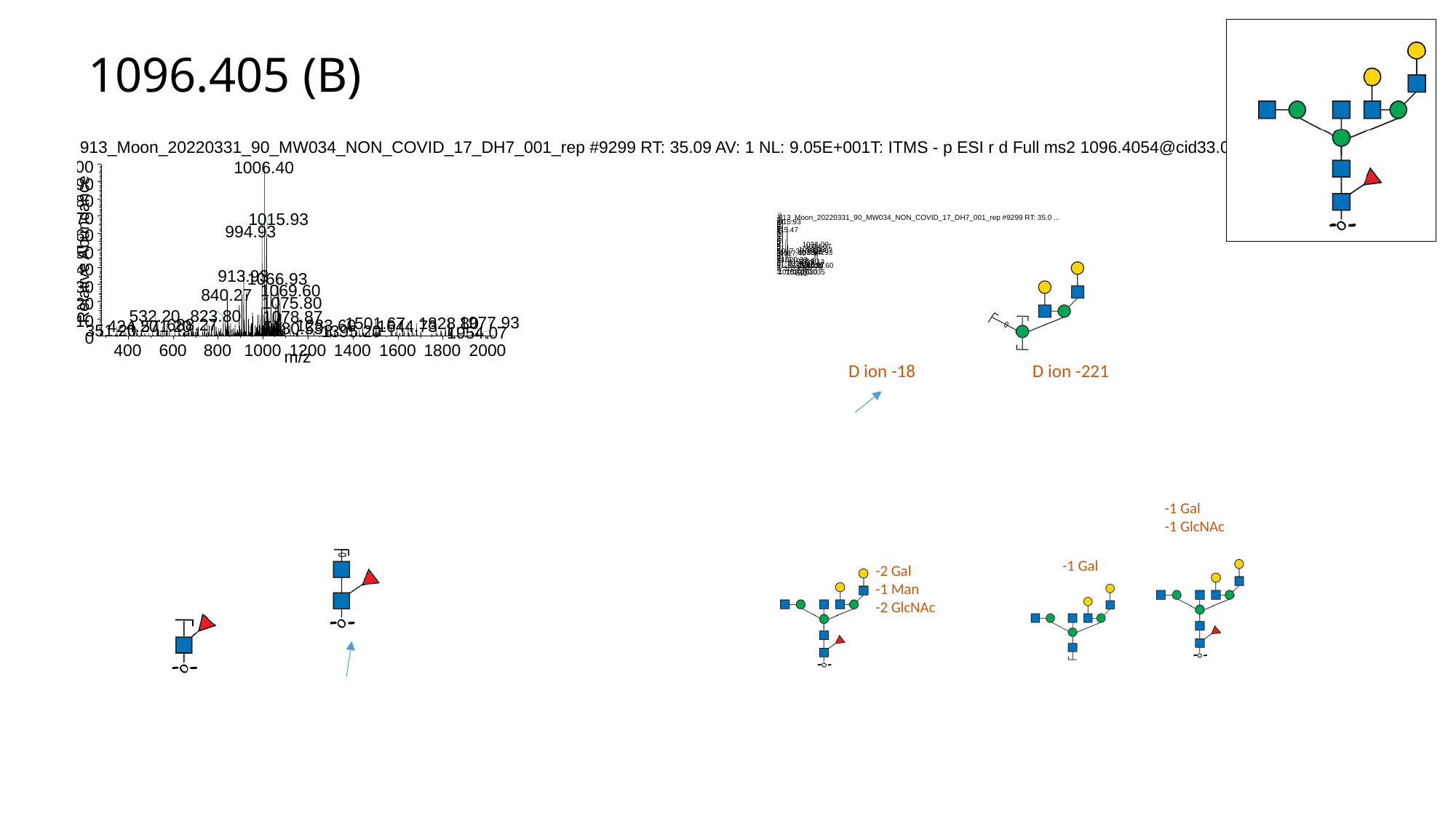

# 1096.405 (B)
D ion -221
D ion -18
-1 Gal
-1 GlcNAc
-1 Gal
-2 Gal
-1 Man
-2 GlcNAc

## Slide 95
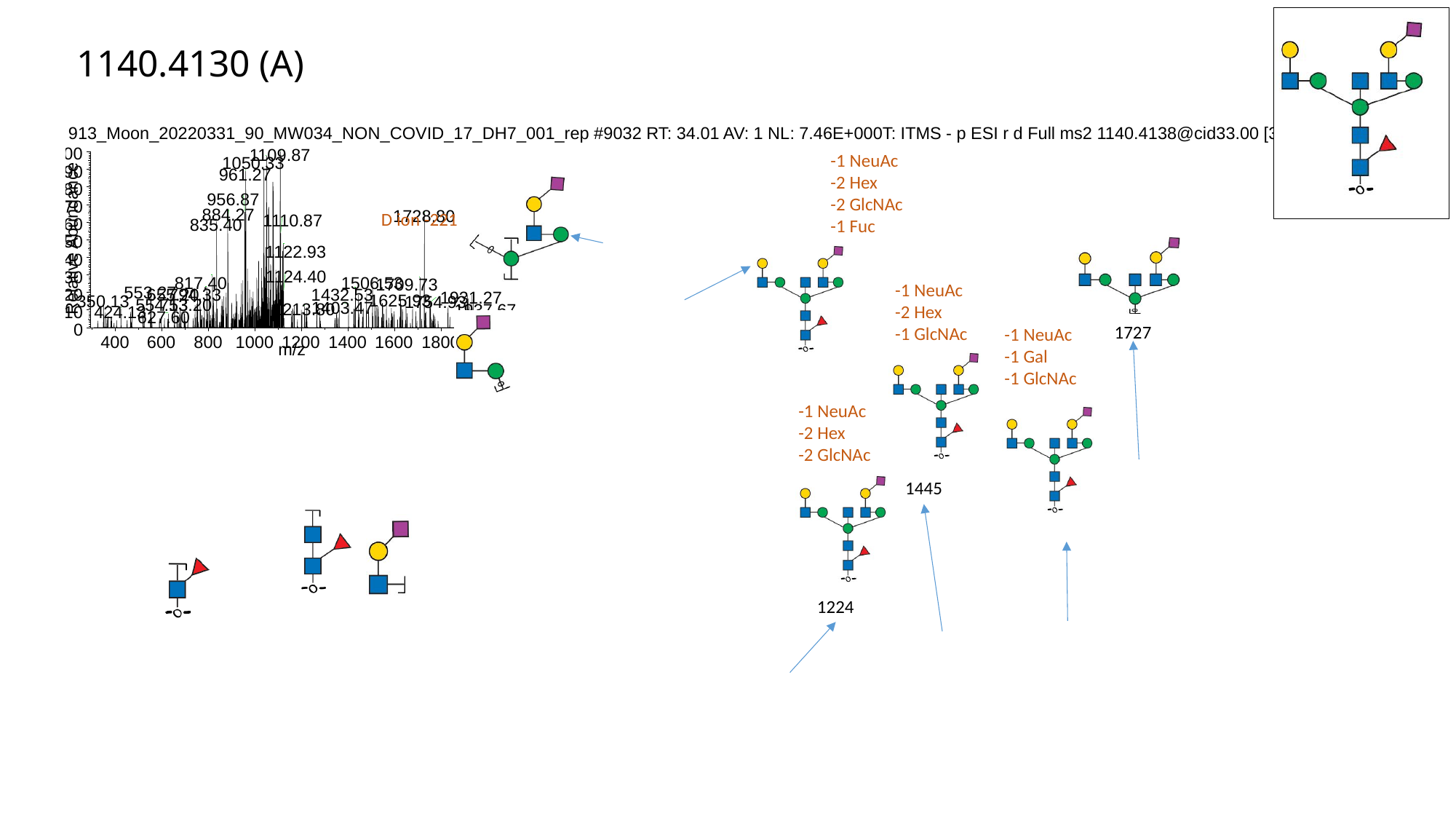

# 1140.4130 (A)
-1 NeuAc
-2 Hex
-2 GlcNAc
-1 Fuc
D ion -221
-1 NeuAc
-2 Hex
-1 GlcNAc
1727
-1 NeuAc
-1 Gal
-1 GlcNAc
-1 NeuAc
-2 Hex
-2 GlcNAc
1445
1224

## Slide 96
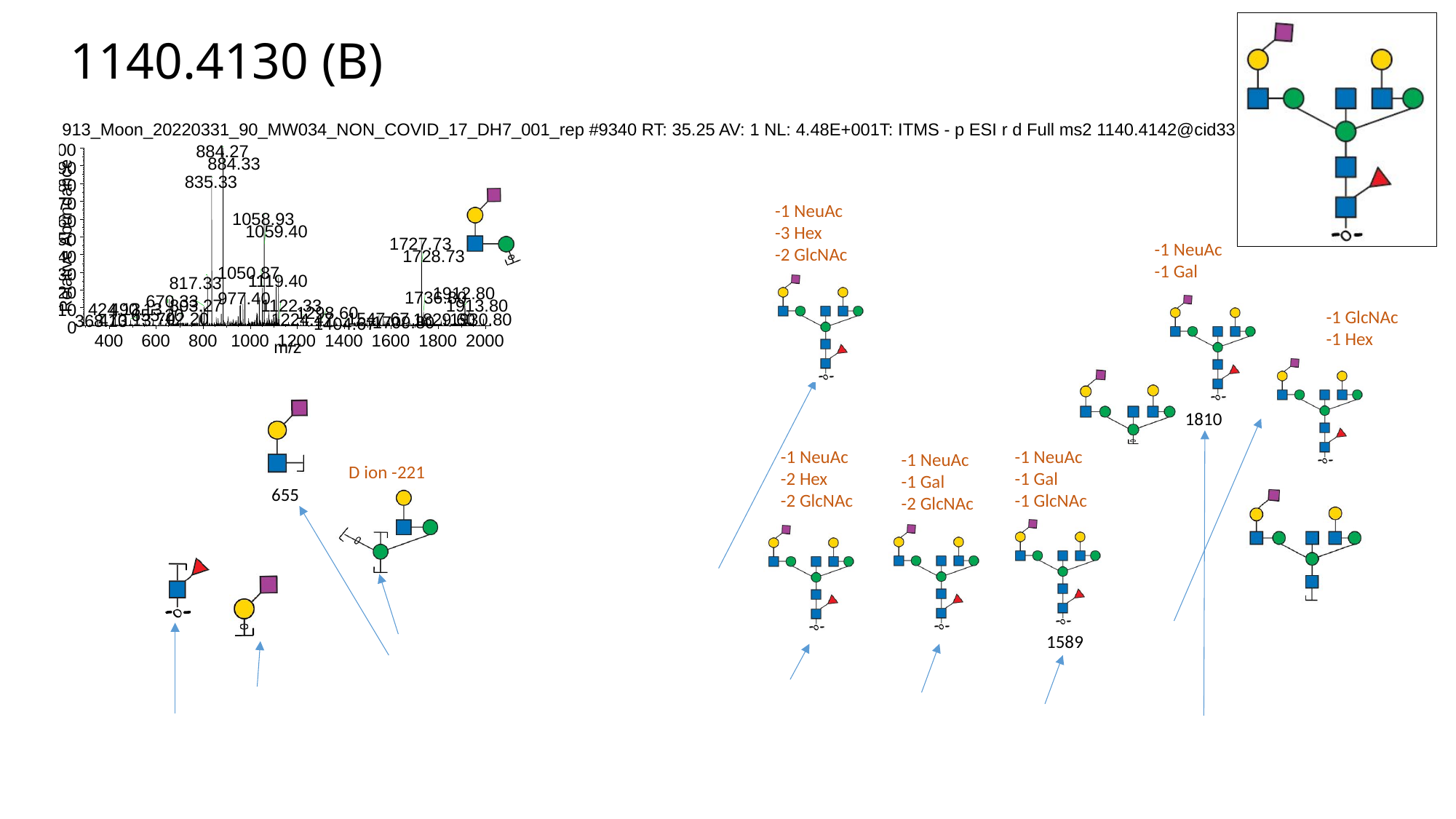

# 1140.4130 (B)
-1 NeuAc
-3 Hex
-2 GlcNAc
-1 NeuAc
-1 Gal
-1 GlcNAc
-1 Hex
1810
-1 NeuAc
-2 Hex
-2 GlcNAc
-1 NeuAc
-1 Gal
-1 GlcNAc
-1 NeuAc
-1 Gal
-2 GlcNAc
D ion -221
655
1589

## Slide 97
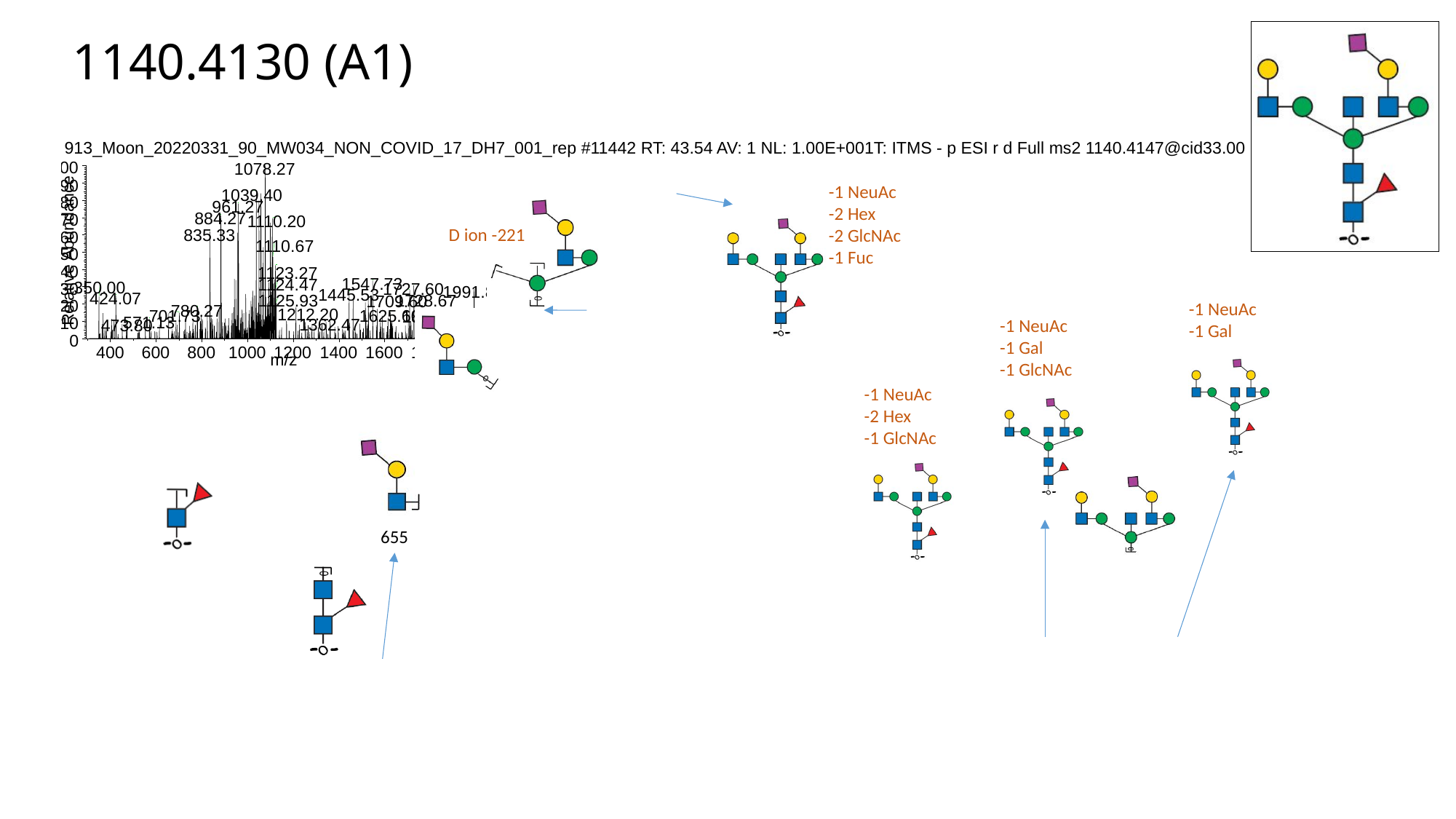

# 1140.4130 (A1)
-1 NeuAc
-2 Hex
-2 GlcNAc
-1 Fuc
D ion -221
-1 NeuAc
-1 Gal
-1 NeuAc
-1 Gal
-1 GlcNAc
-1 NeuAc
-2 Hex
-1 GlcNAc
655

## Slide 98
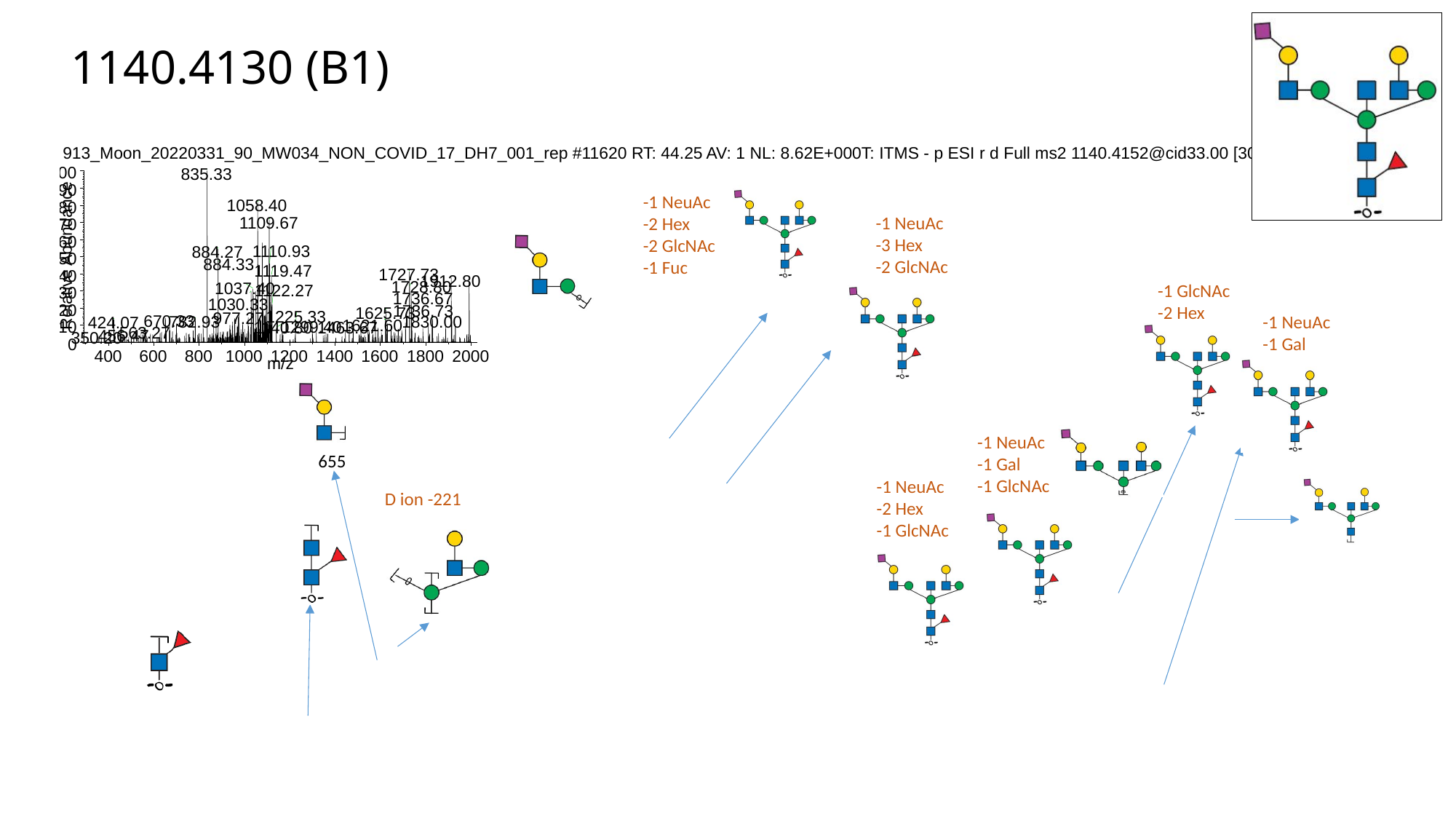

# 1140.4130 (B1)
-1 NeuAc
-2 Hex
-2 GlcNAc
-1 Fuc
-1 NeuAc
-3 Hex
-2 GlcNAc
-1 GlcNAc
-2 Hex
-1 NeuAc
-1 Gal
-1 NeuAc
-1 Gal
-1 GlcNAc
655
-1 NeuAc
-2 Hex
-1 GlcNAc
D ion -221

## Slide 99
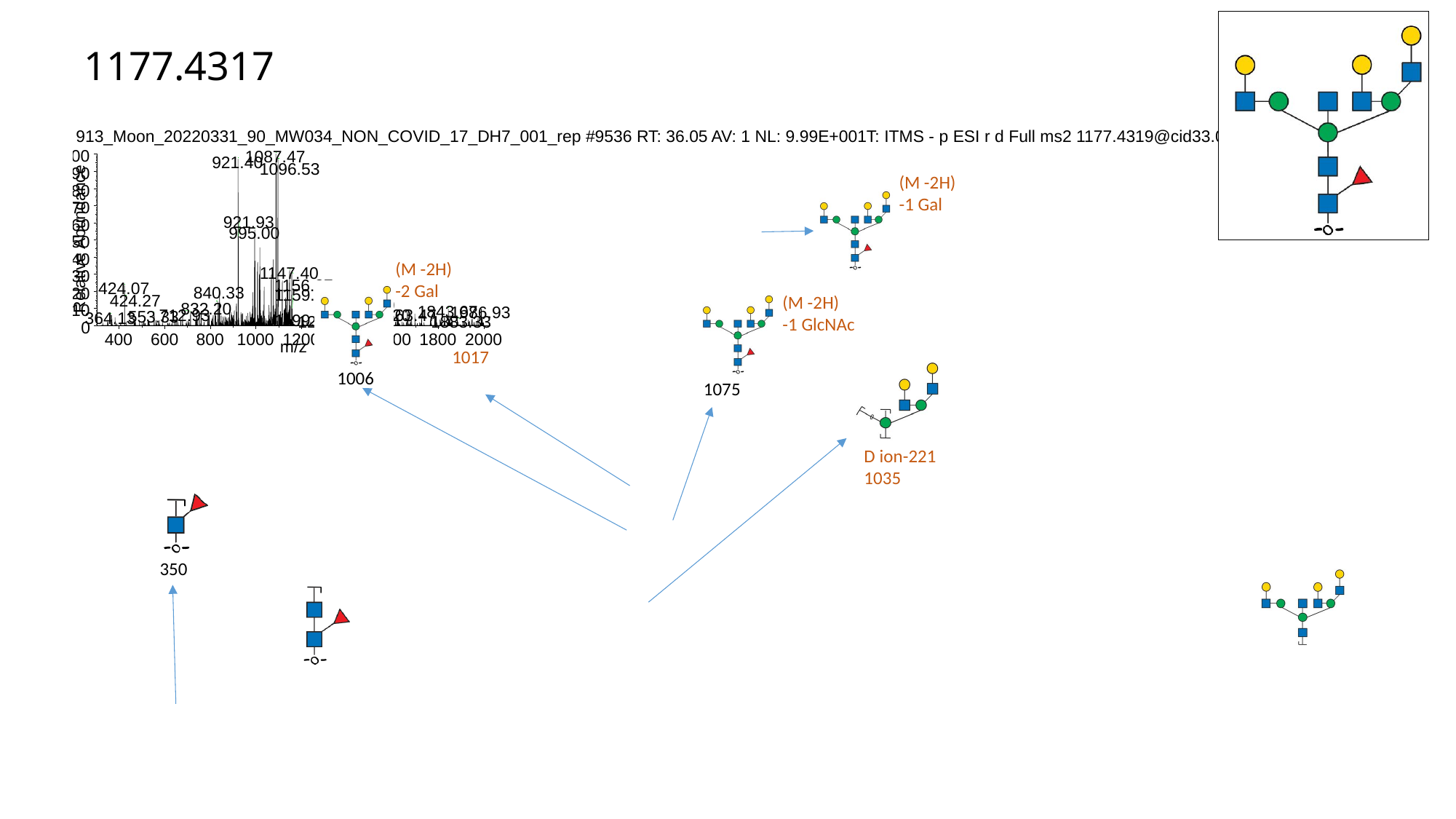

# 1177.4317
(M -2H)
-1 Gal
(M -2H)
-2 Gal
(M -2H)
-1 GlcNAc
1017
1006
1075
D ion-221
1035
350

## Slide 100
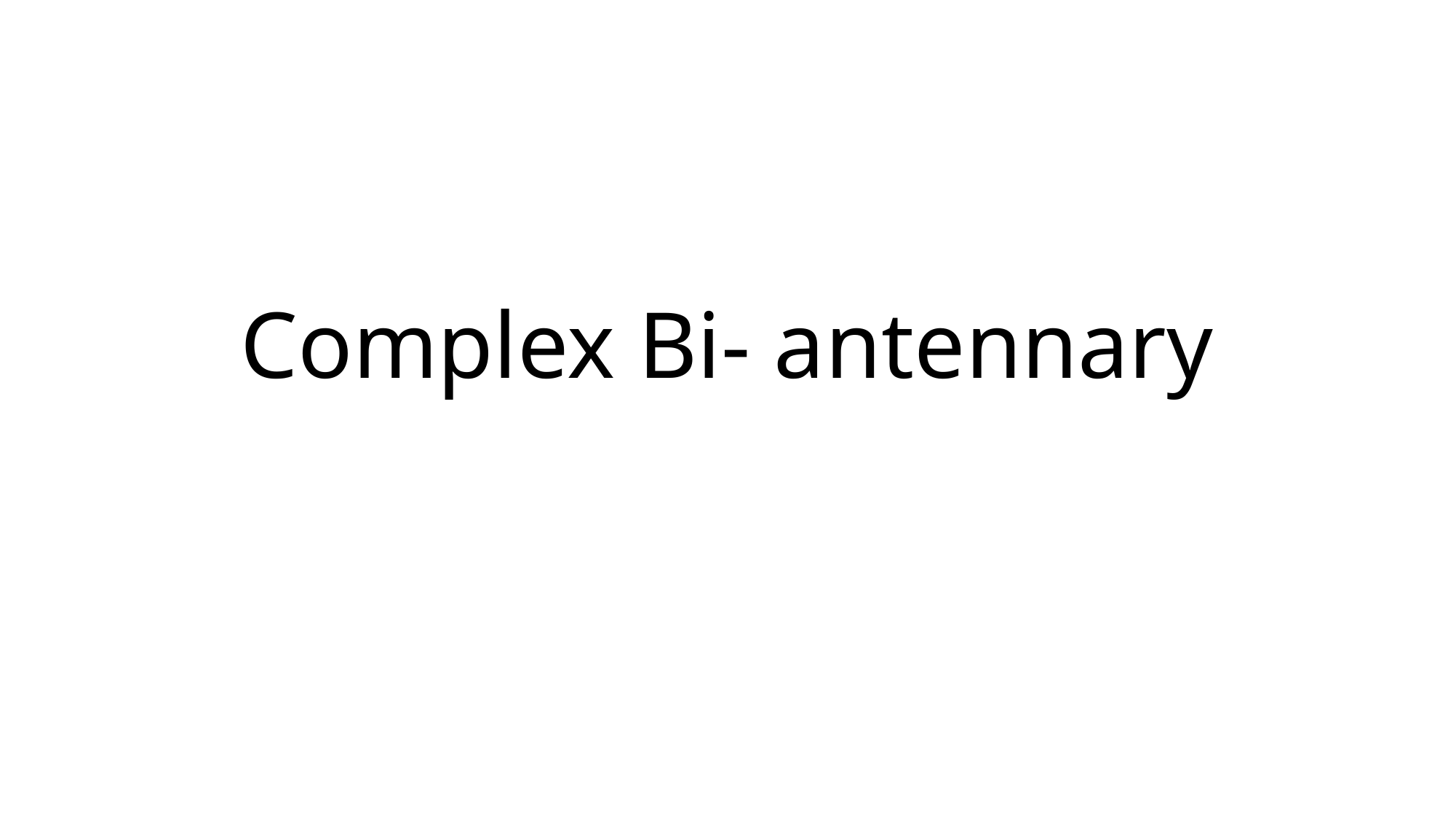

# Complex Bi- antennary

## Slide 101
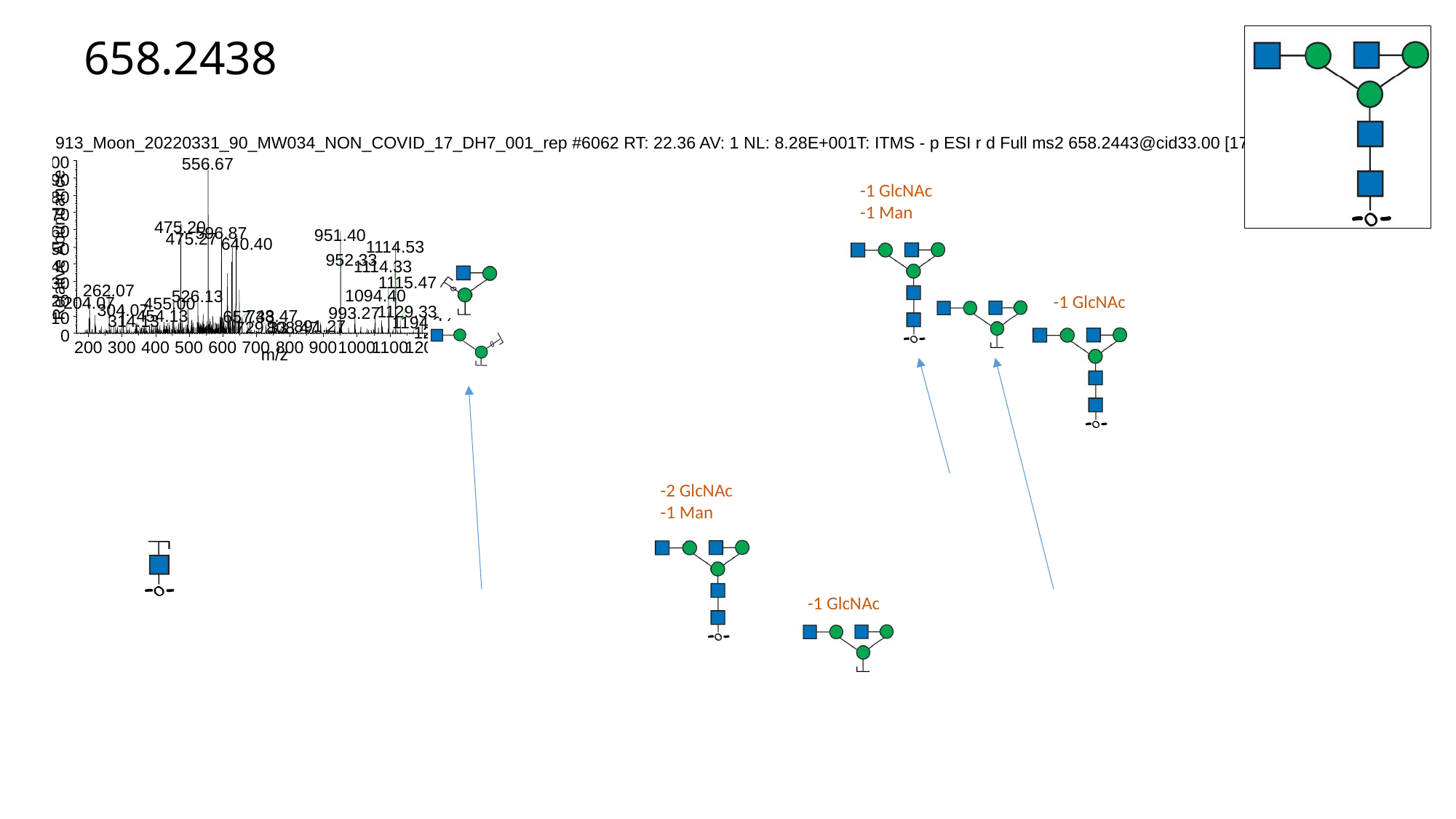

# 658.2438
-1 GlcNAc
-1 Man
-1 GlcNAc
-2 GlcNAc
-1 Man
-1 GlcNAc

## Slide 102
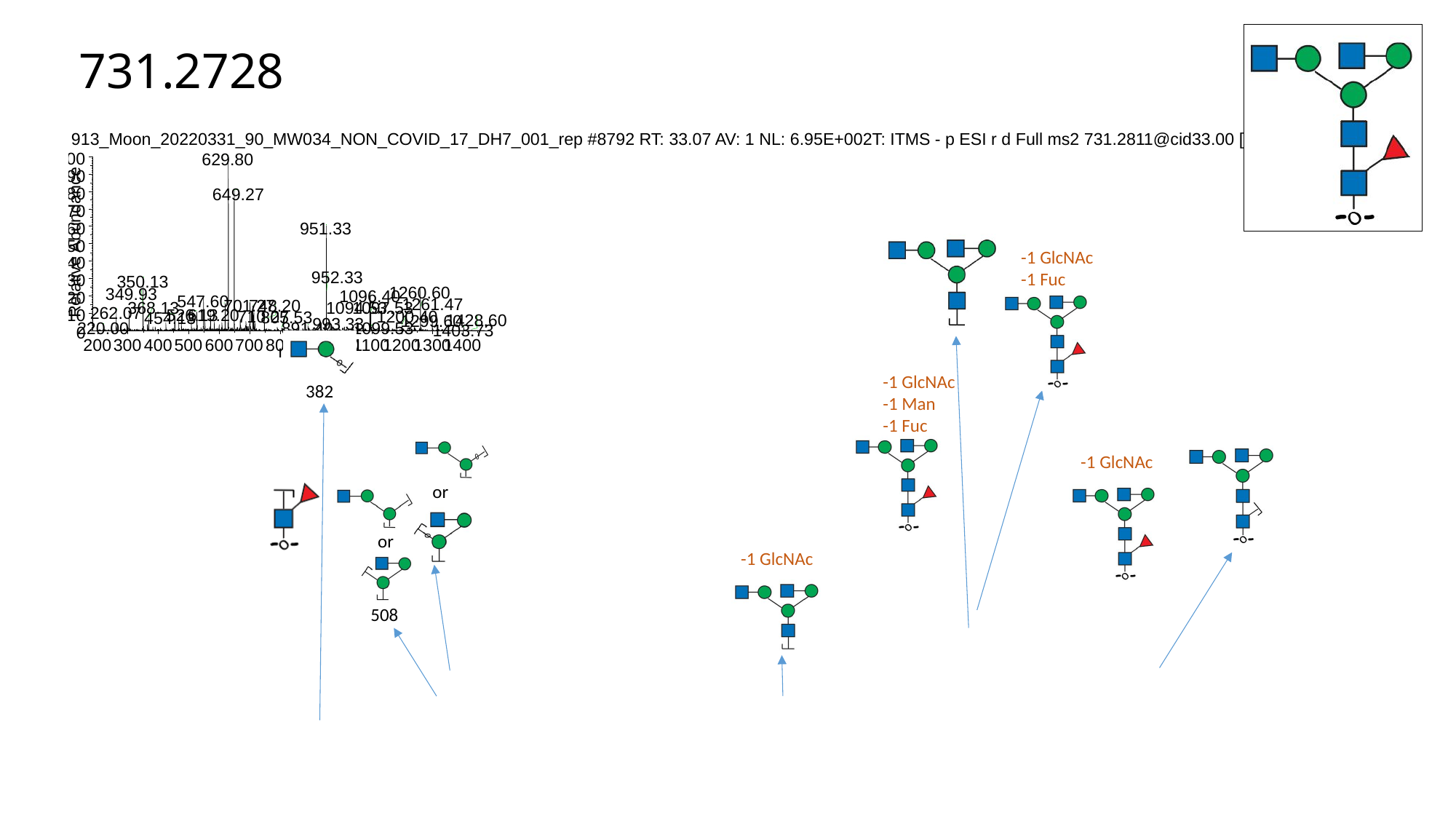

# 731.2728
-1 GlcNAc
-1 Fuc
-1 GlcNAc
-1 Man
-1 Fuc
382
-1 GlcNAc
or
or
-1 GlcNAc
508

## Slide 103
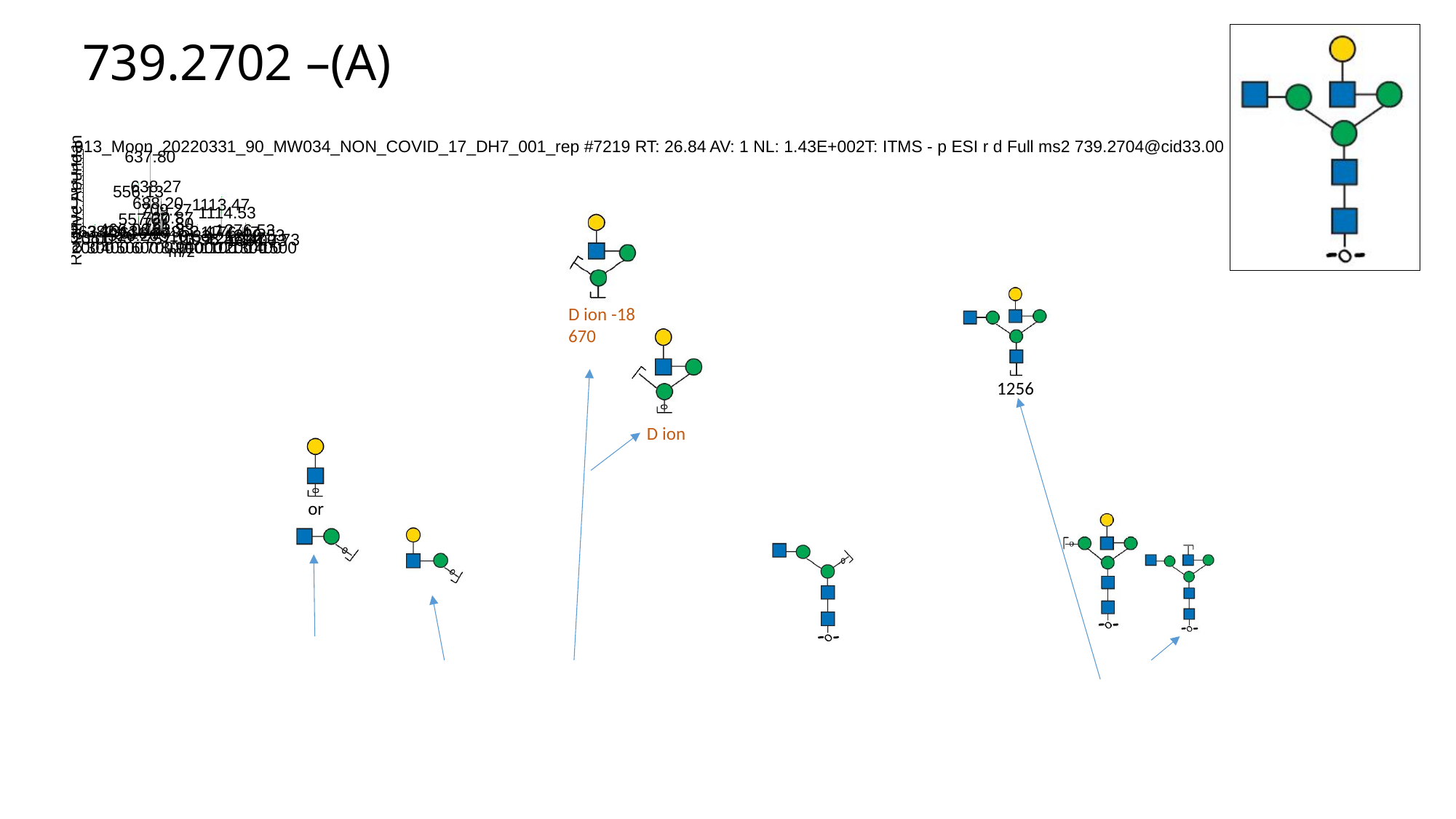

# 739.2702 –(A)
D ion -18
670
1256
D ion
or

## Slide 104
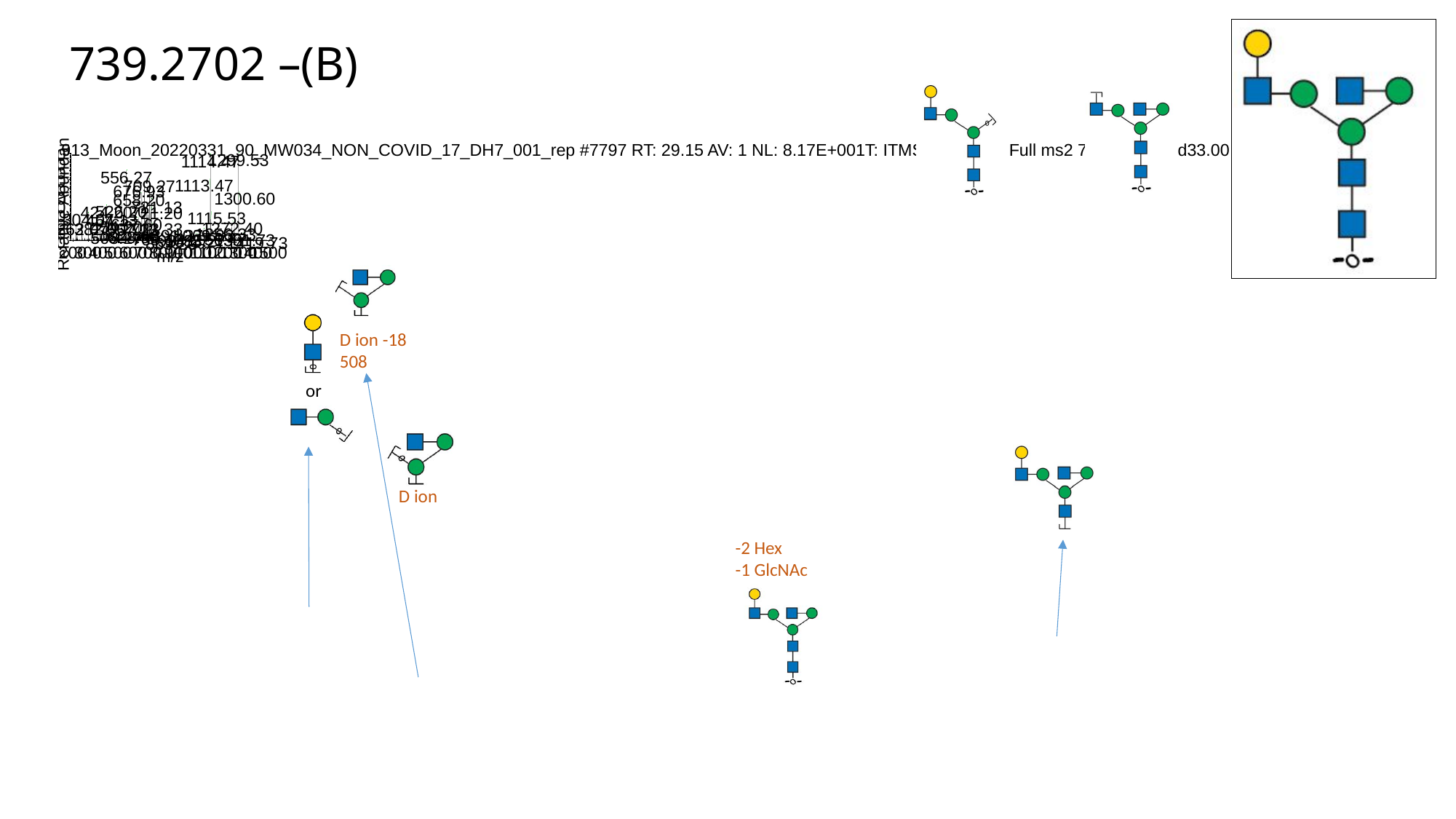

# 739.2702 –(B)
D ion -18
508
or
D ion
-2 Hex
-1 GlcNAc

## Slide 105
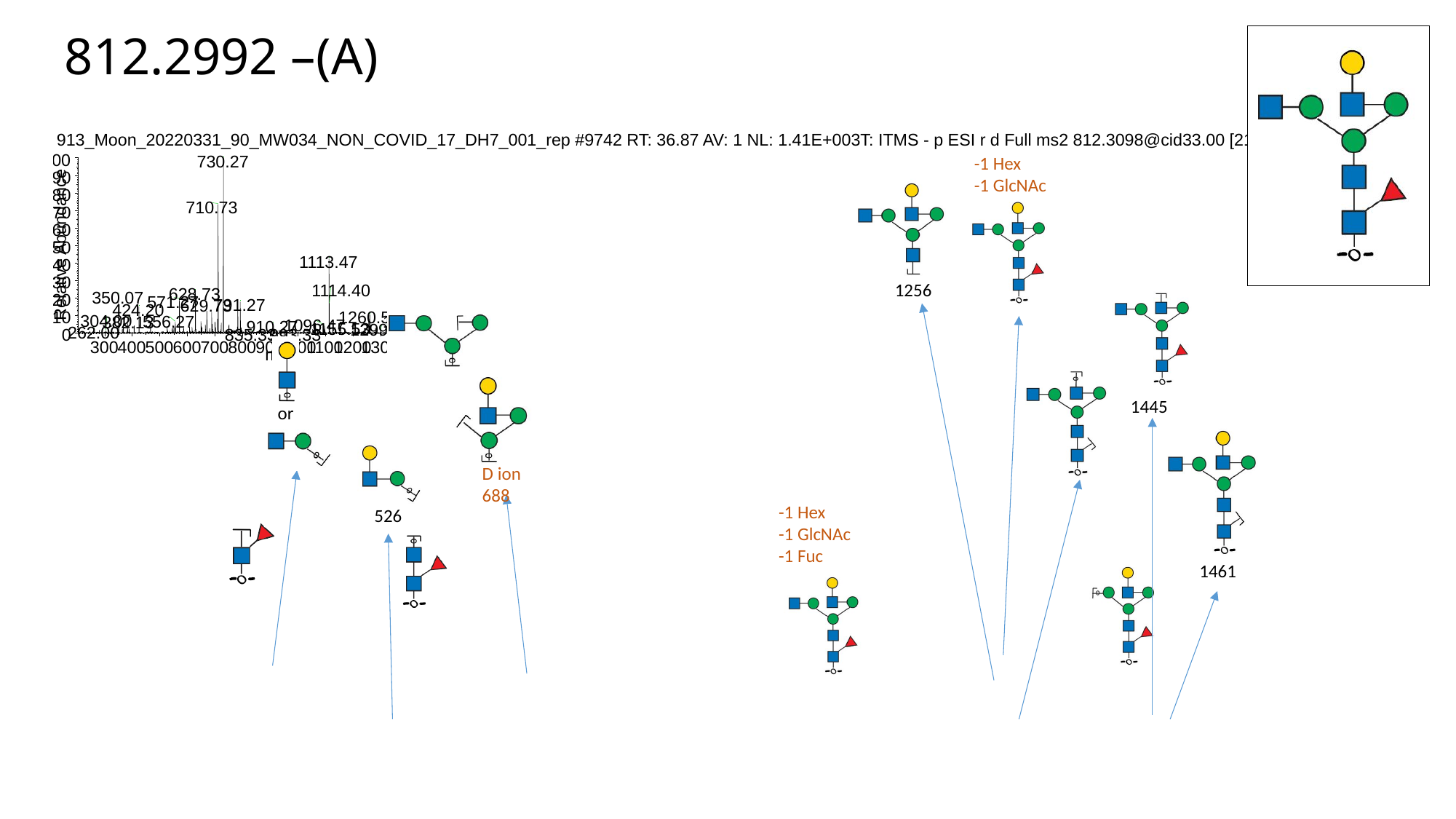

# 812.2992 –(A)
-1 Hex
-1 GlcNAc
1256
1445
or
D ion
688
-1 Hex
-1 GlcNAc
-1 Fuc
526
1461

## Slide 106
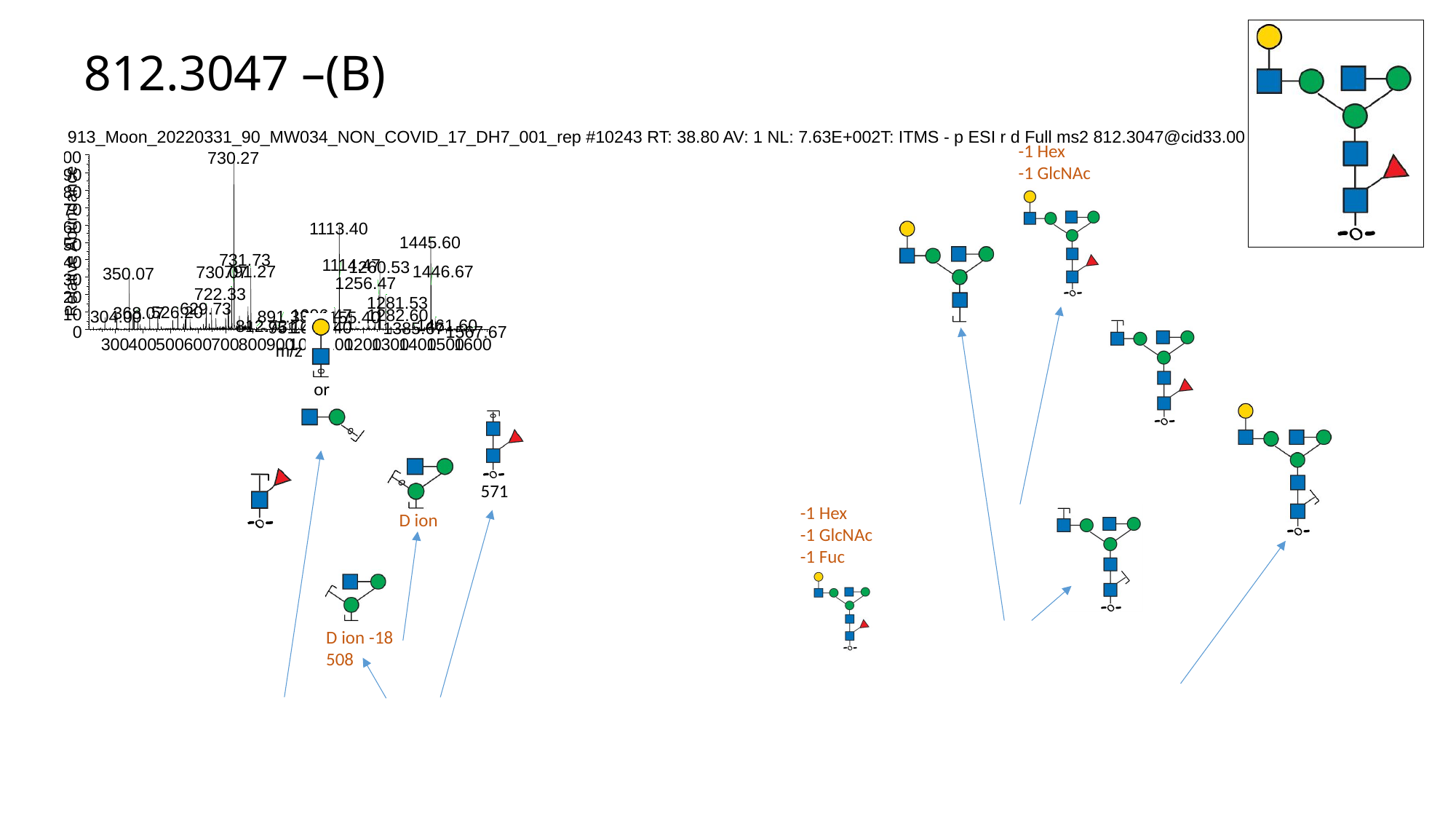

# 812.3047 –(B)
-1 Hex
-1 GlcNAc
or
571
-1 Hex
-1 GlcNAc
-1 Fuc
D ion
D ion -18
508

## Slide 107
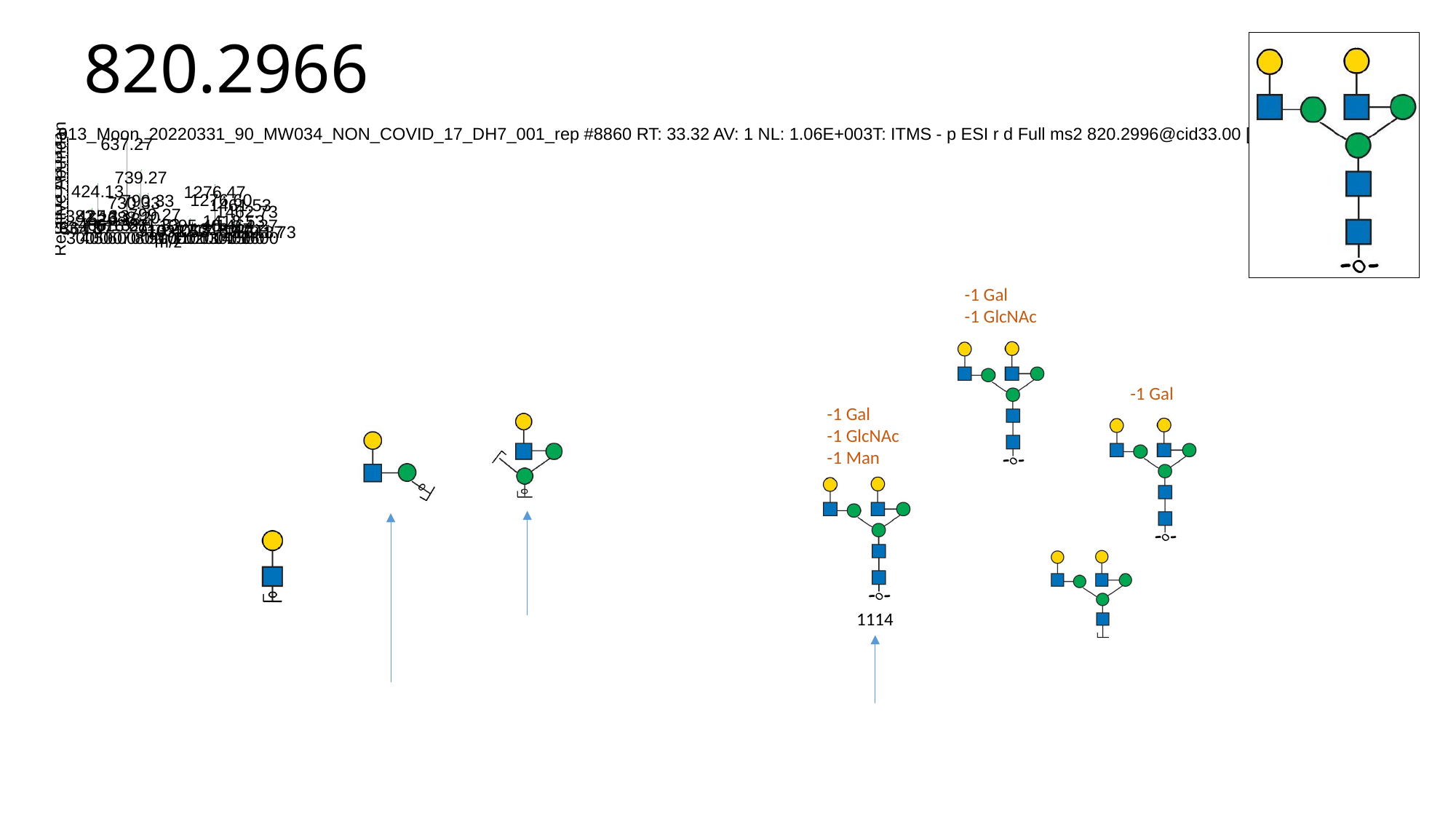

# 820.2966
-1 Gal
-1 GlcNAc
-1 Gal
-1 Gal
-1 GlcNAc
-1 Man
1114

## Slide 108
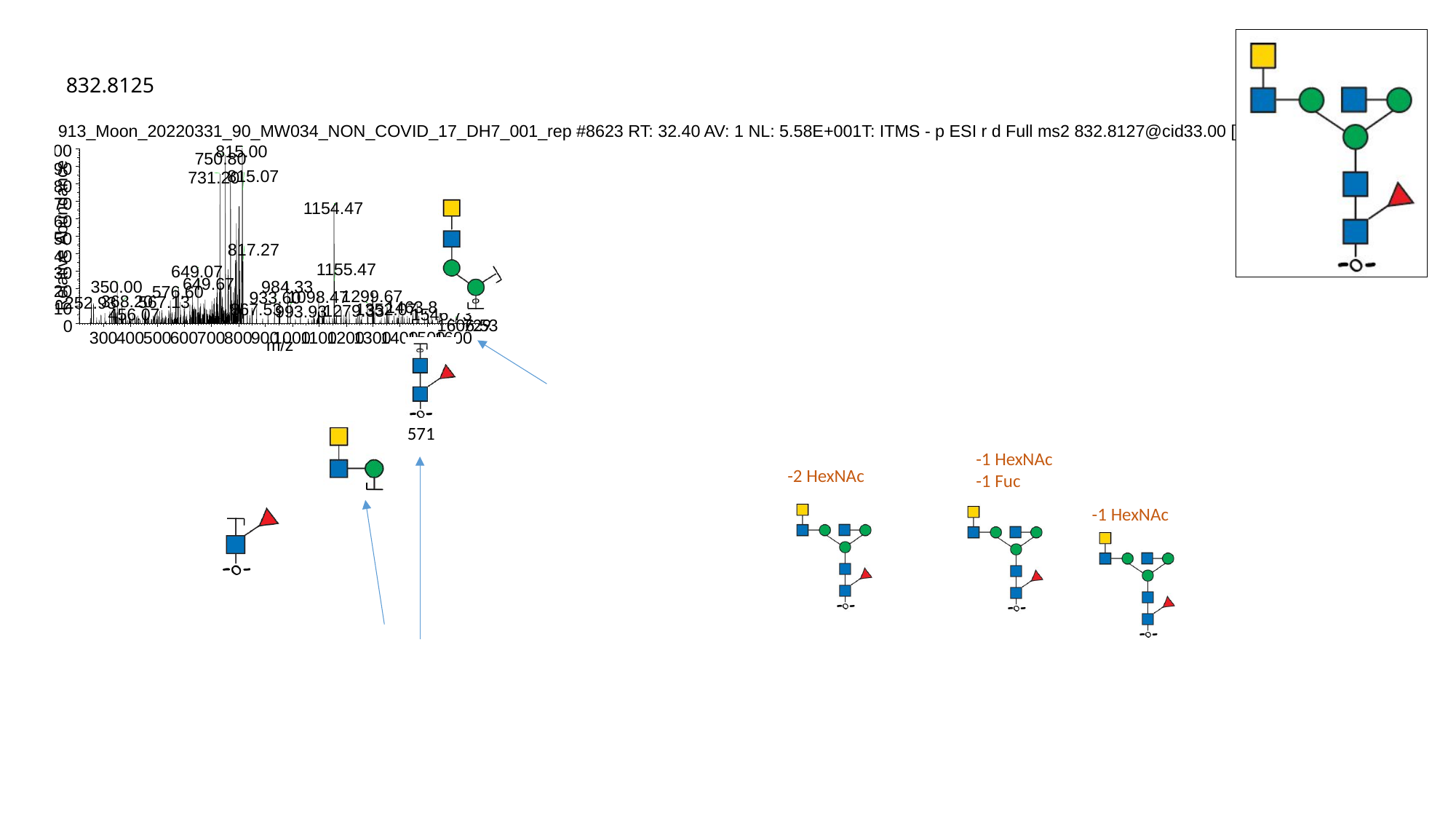

# 832.8125
729
571
-1 HexNAc
-1 Fuc
-2 HexNAc
-1 HexNAc

## Slide 109
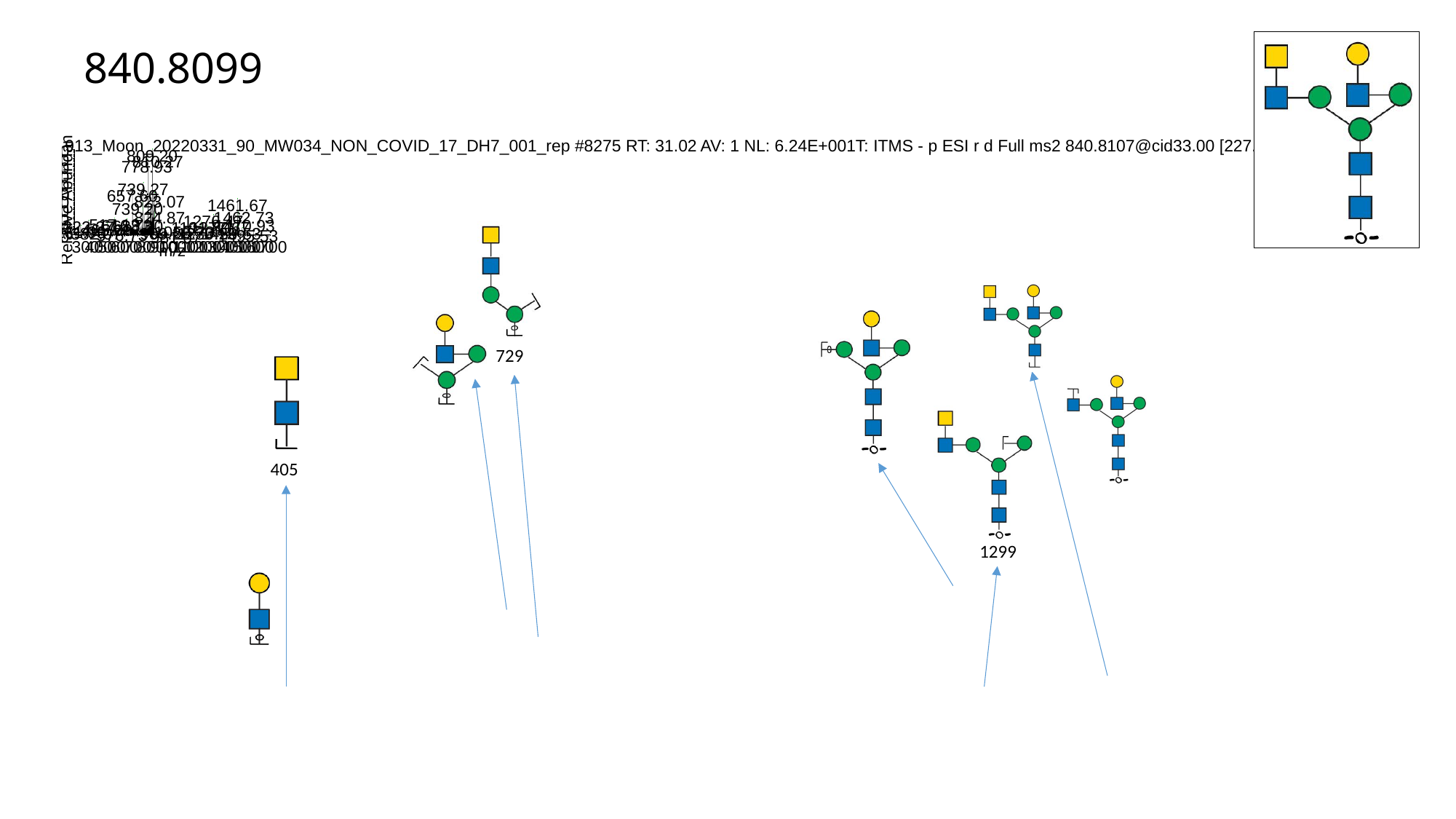

# 840.8099
729
405
1299

## Slide 110
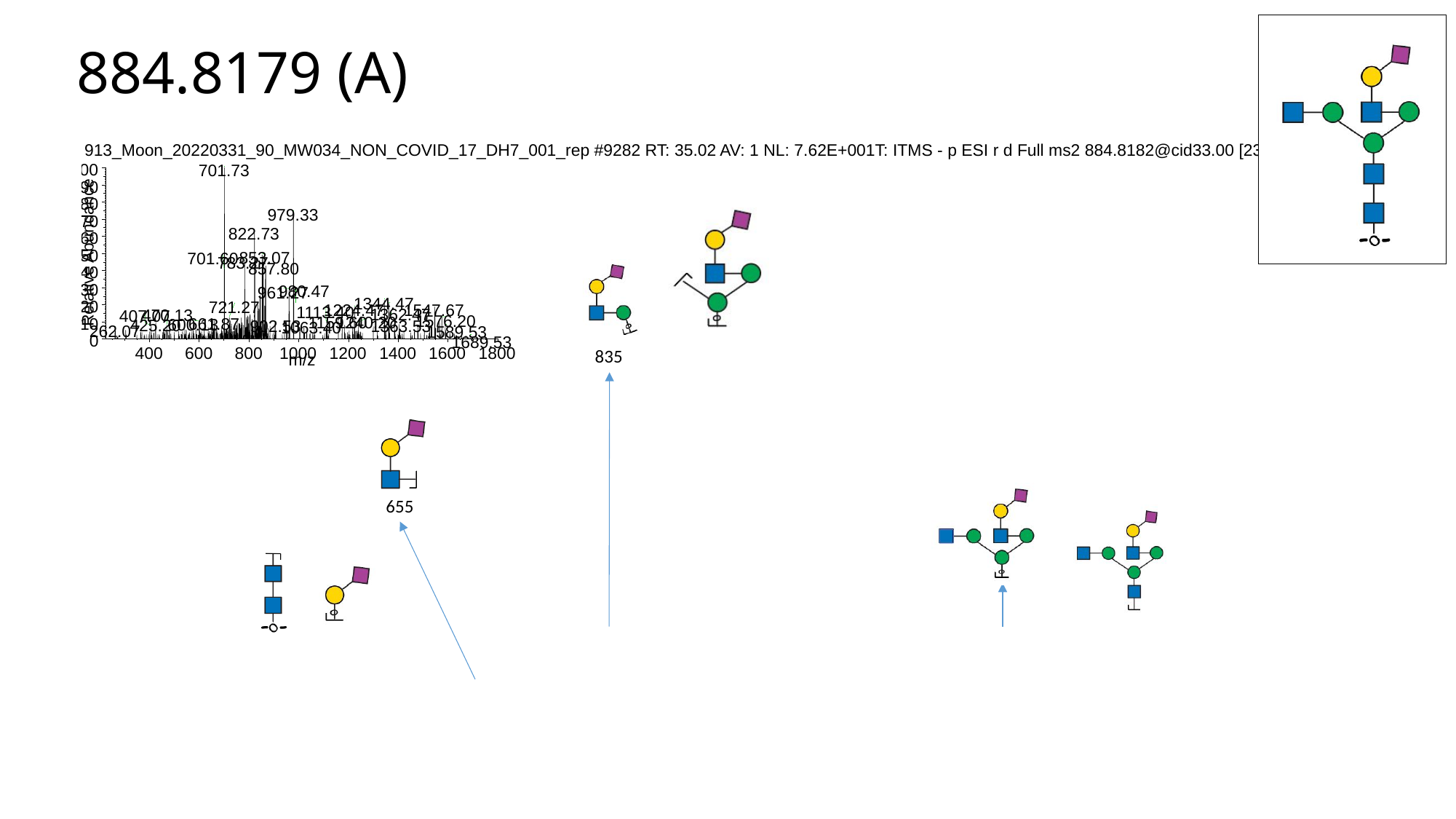

# 884.8179 (A)
835
655

## Slide 111
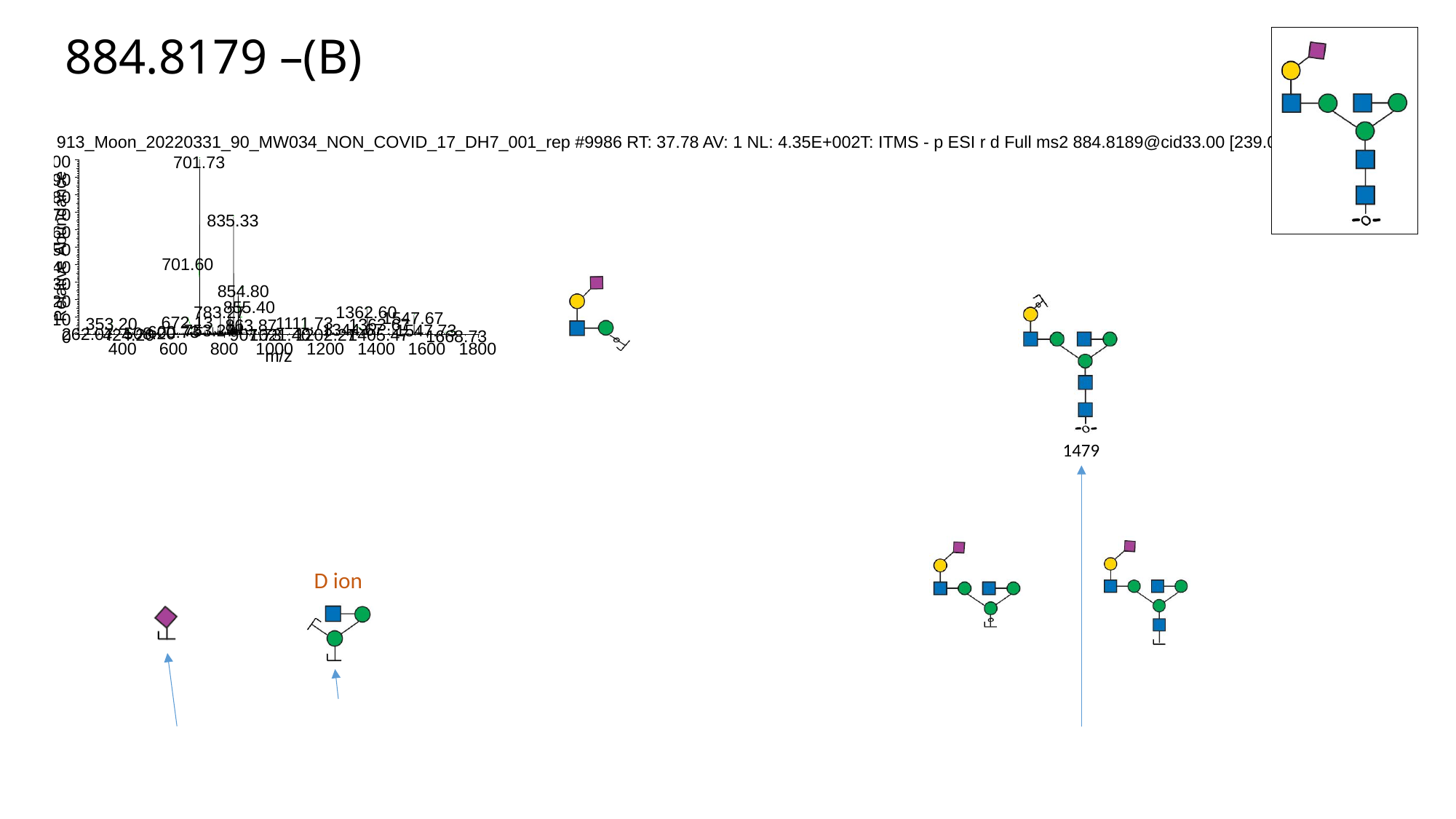

# 884.8179 –(B)
1479
D ion

## Slide 112
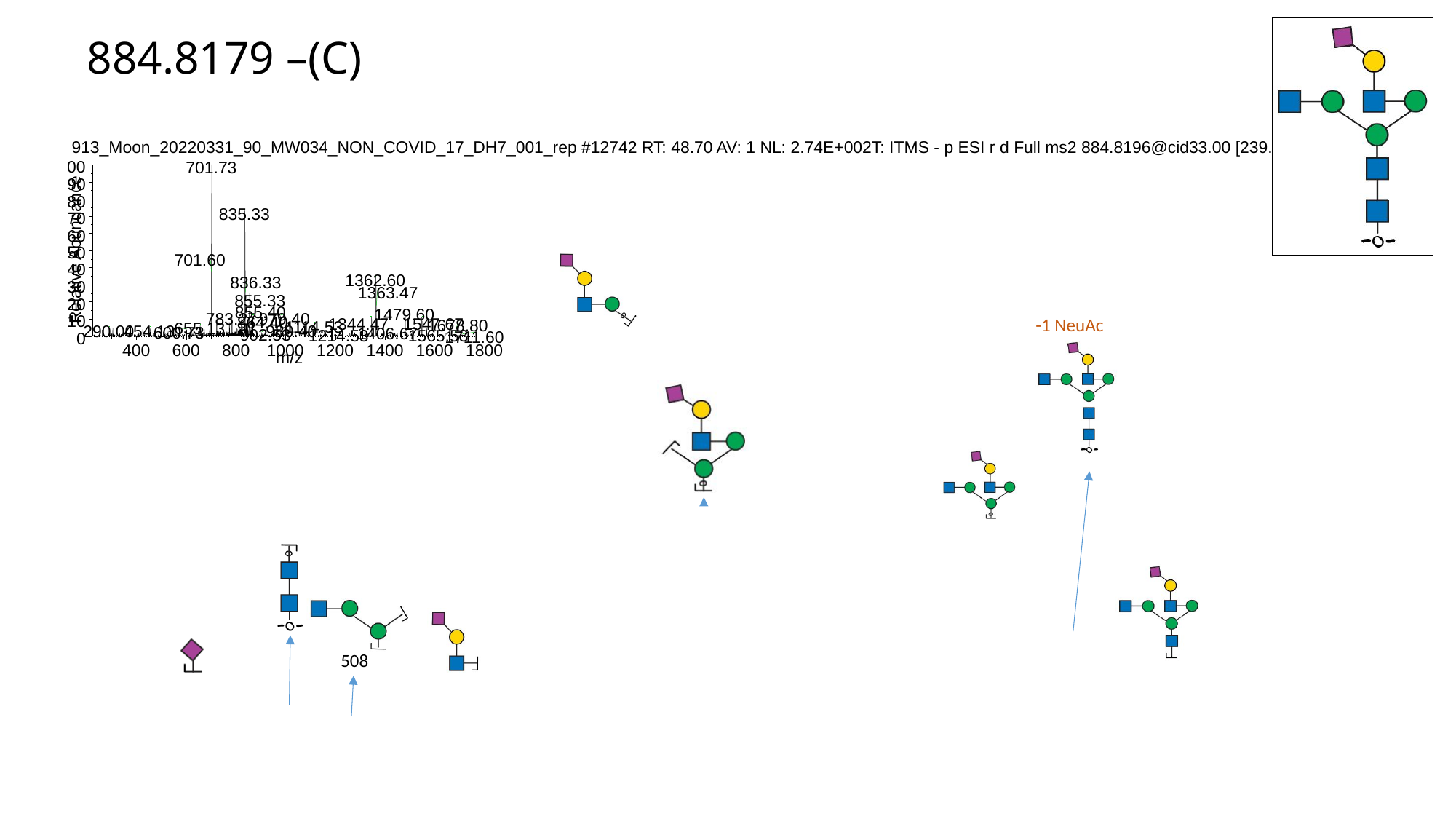

# 884.8179 –(C)
-1 NeuAc
508

## Slide 113
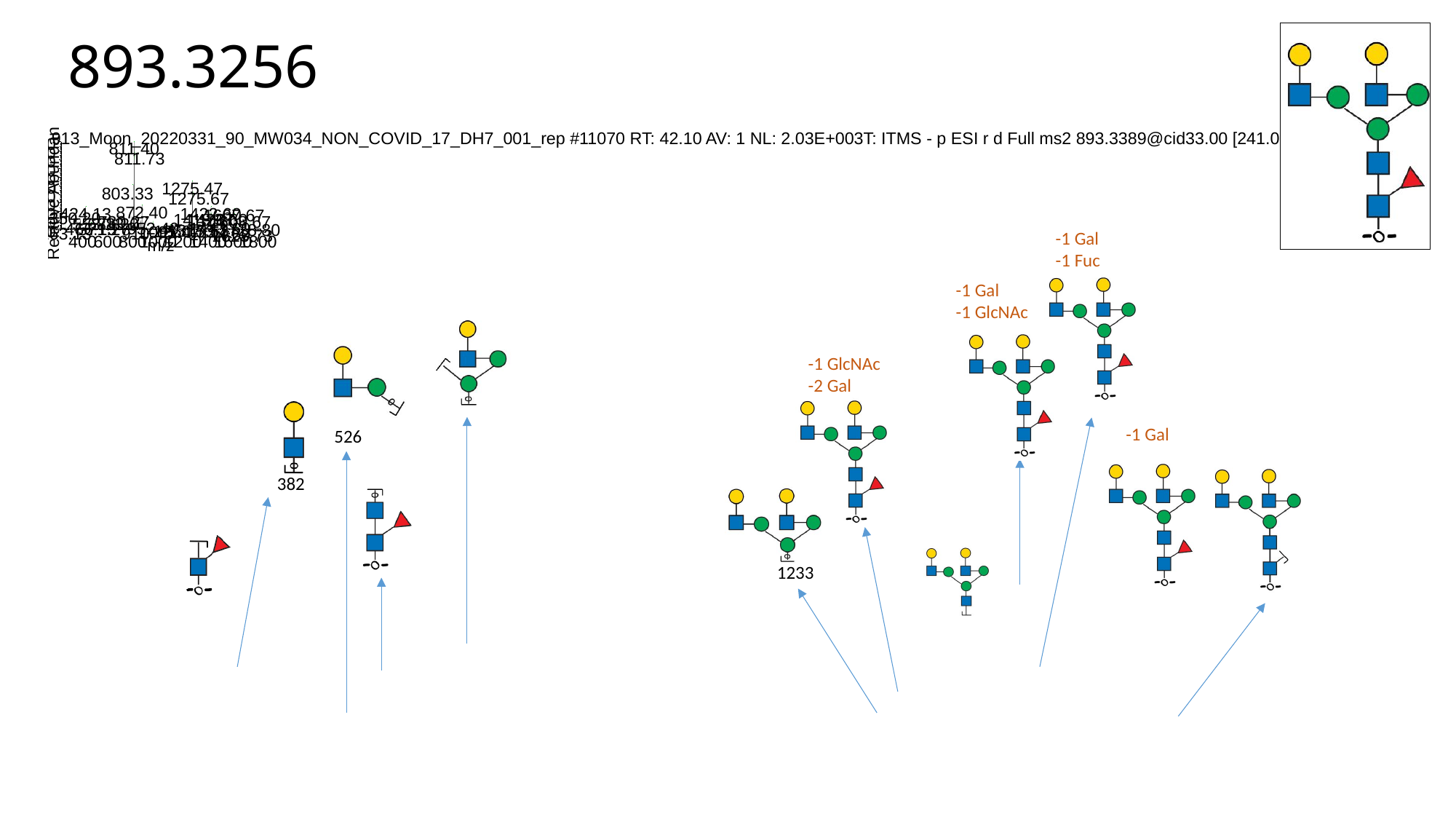

# 893.3256
-1 Gal
-1 Fuc
-1 Gal
-1 GlcNAc
-1 GlcNAc
-2 Gal
-1 Gal
526
382
1233

## Slide 114
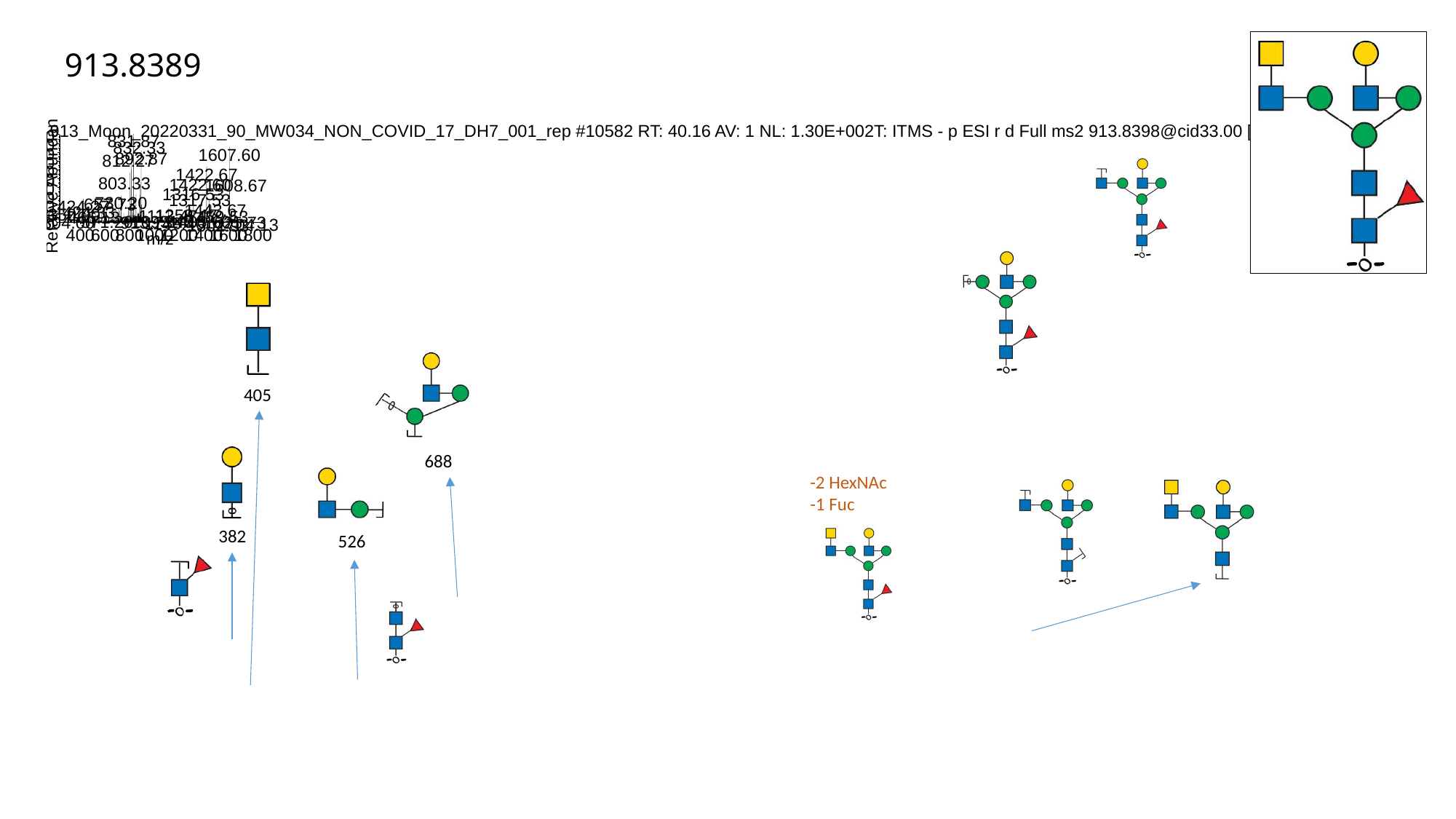

# 913.8389
405
688
-2 HexNAc
-1 Fuc
382
526

## Slide 115
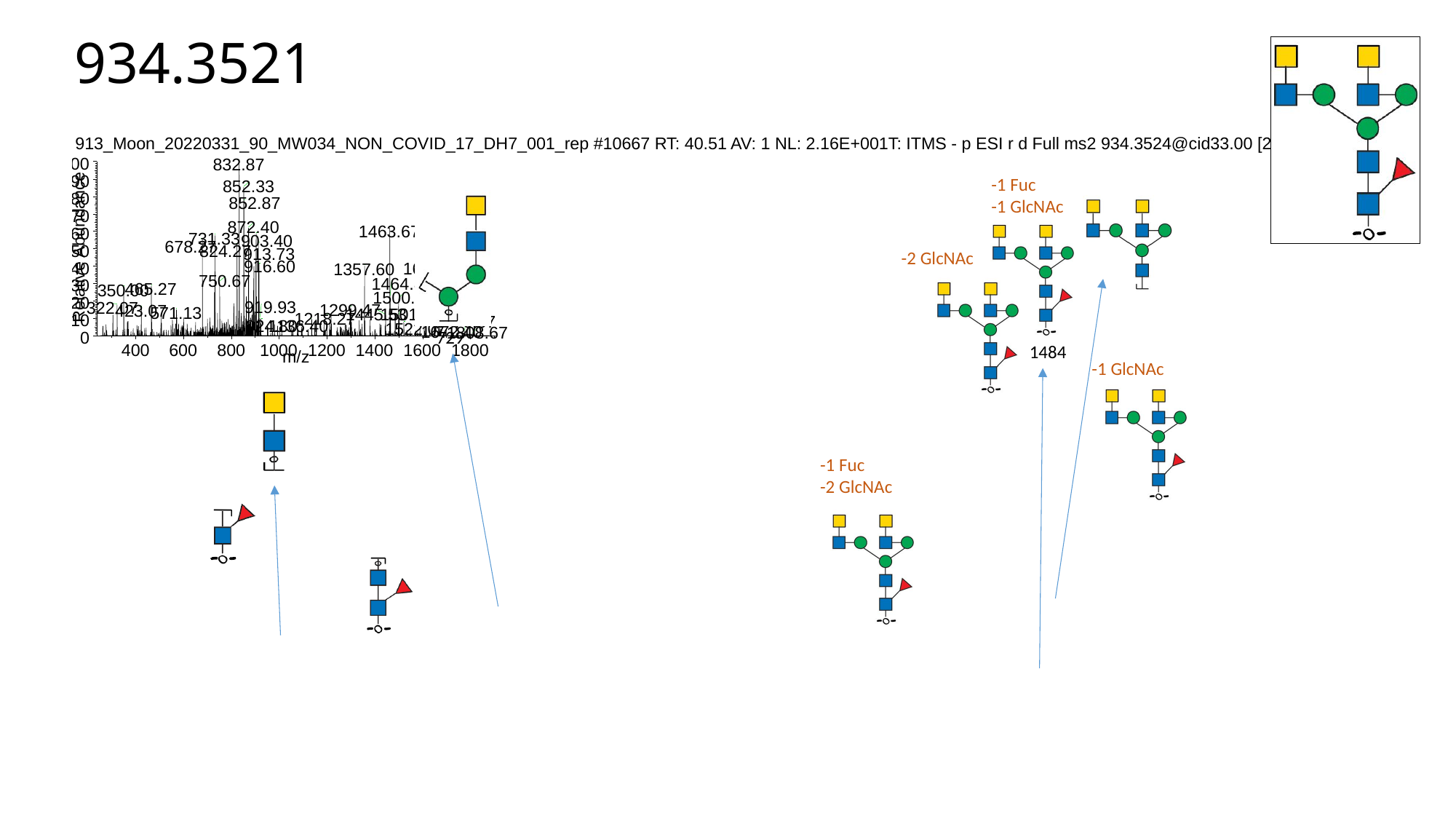

# 934.3521
-1 Fuc
-1 GlcNAc
-2 GlcNAc
729
1484
-1 GlcNAc
-1 Fuc
-2 GlcNAc

## Slide 116
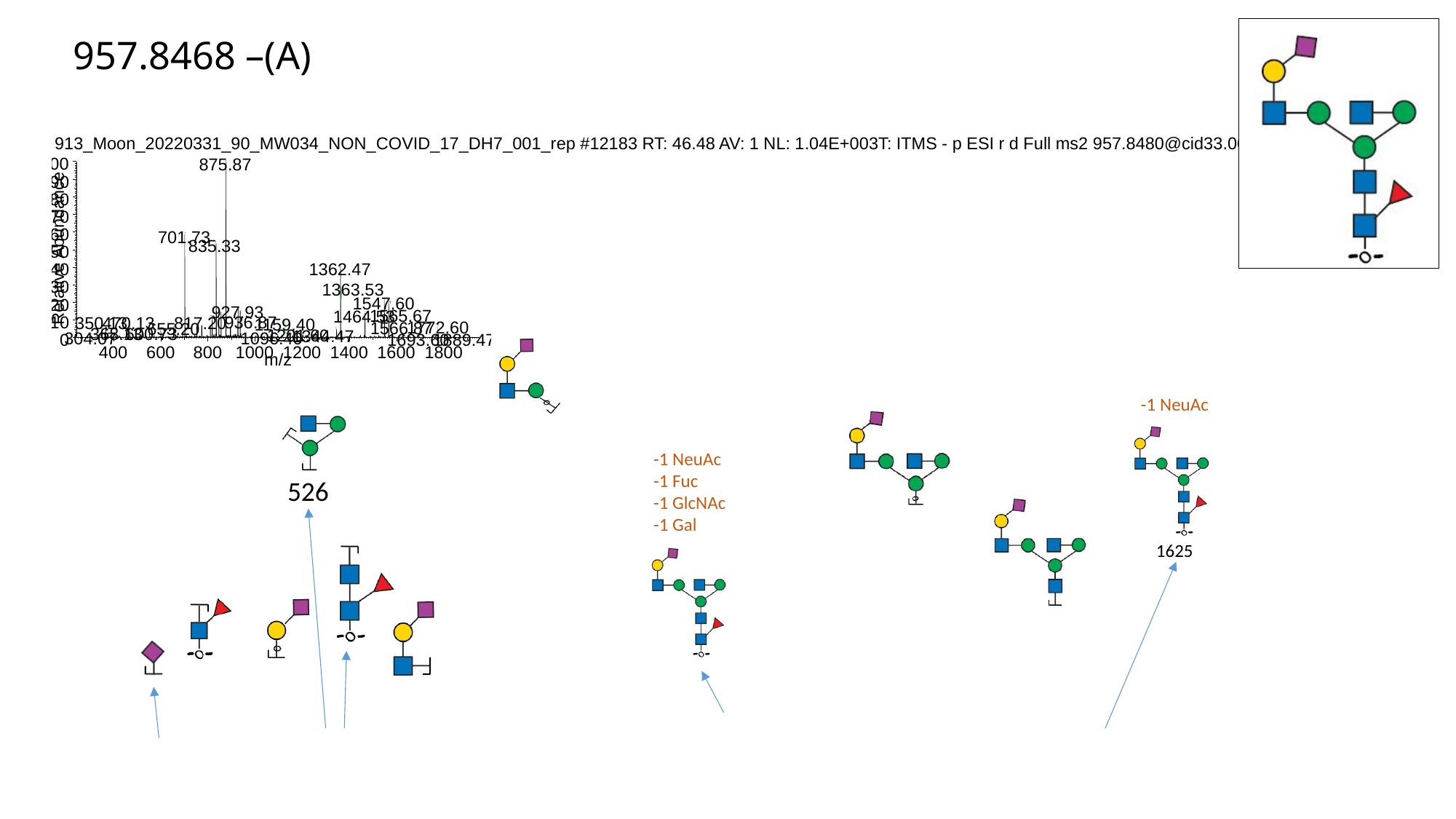

# 957.8468 –(A)
-1 NeuAc
-1 NeuAc
-1 Fuc
-1 GlcNAc
-1 Gal
526
1625

## Slide 117
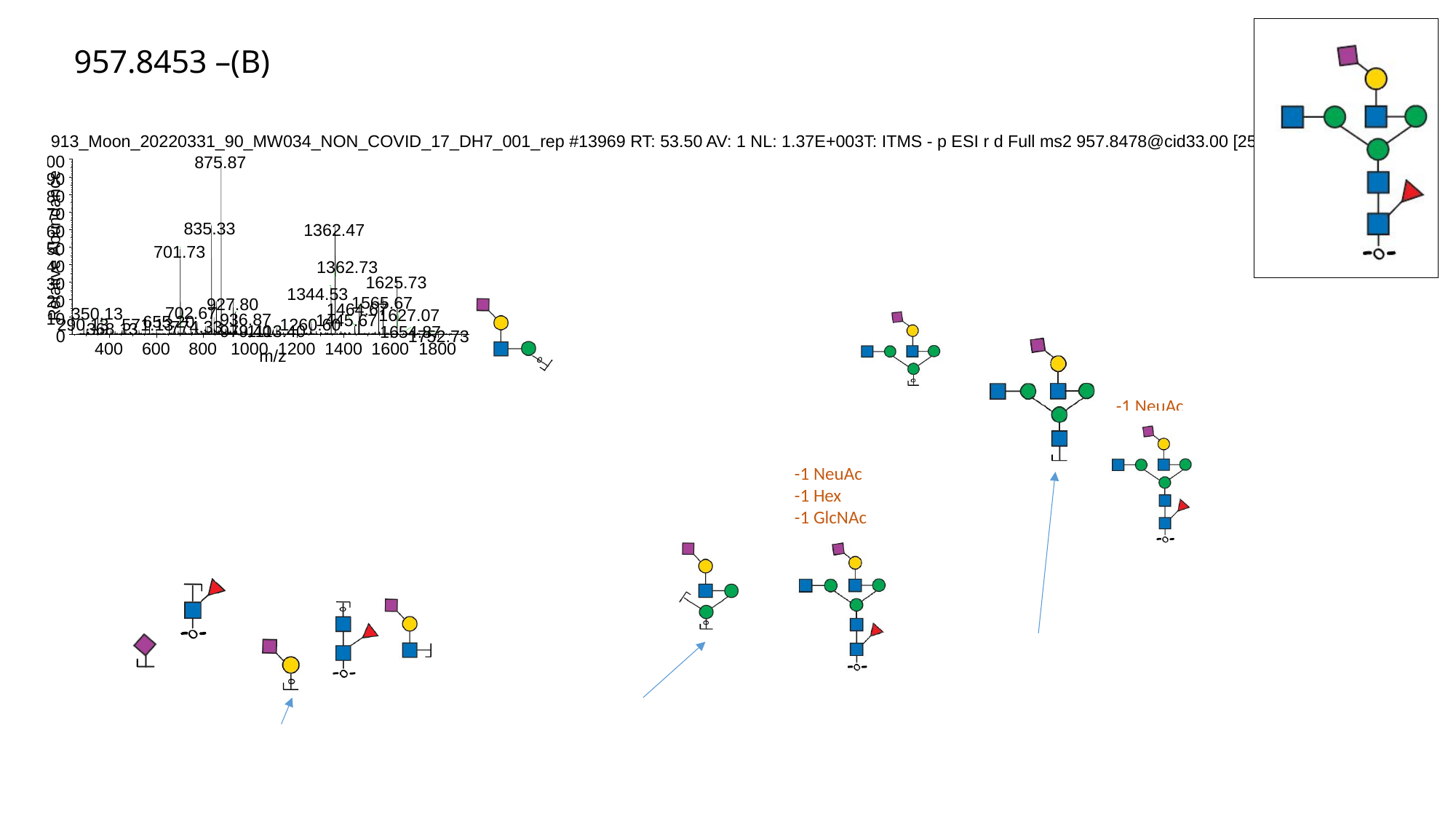

# 957.8453 –(B)
-1 NeuAc
-1 NeuAc
-1 Hex
-1 GlcNAc

## Slide 118
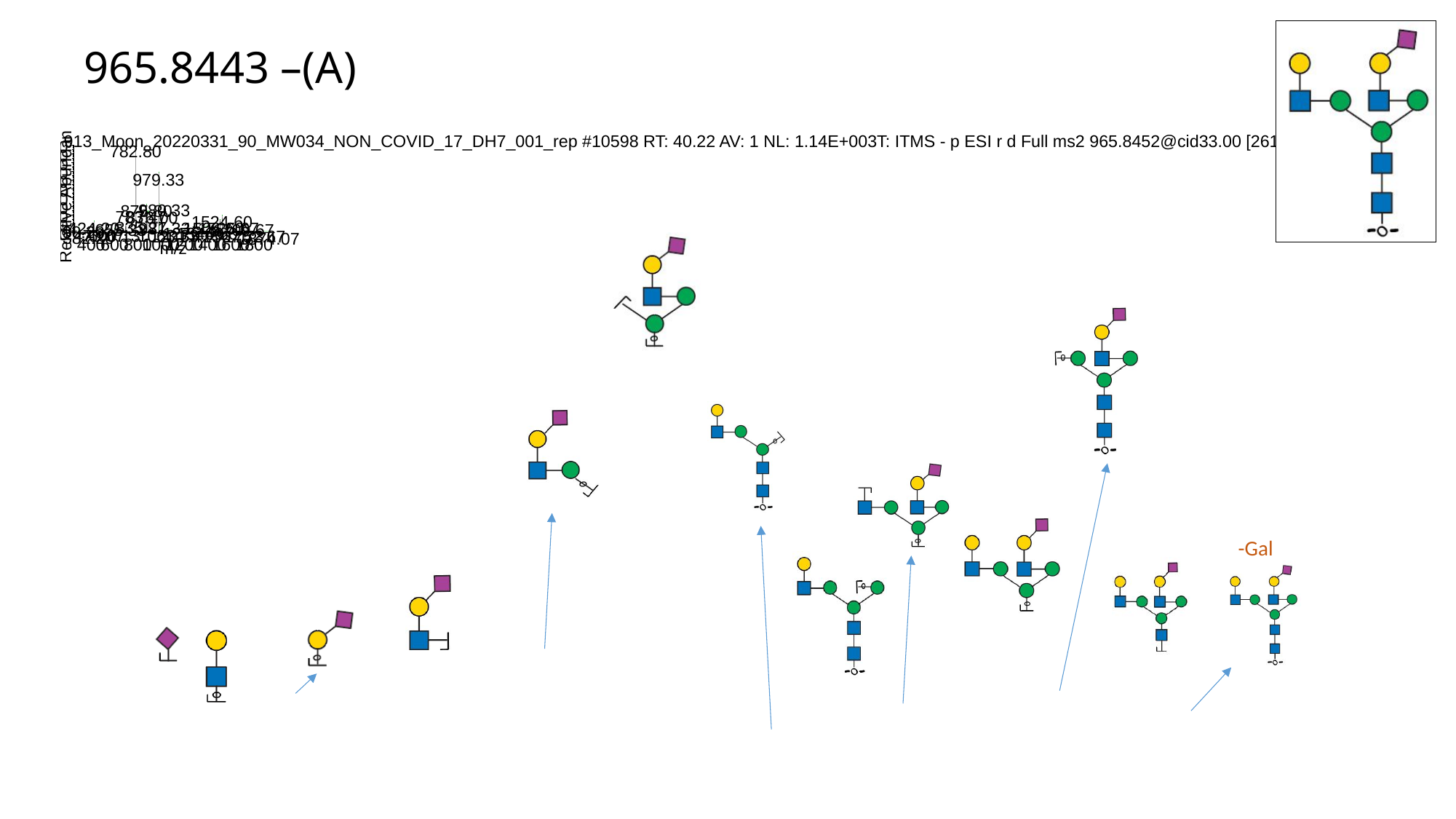

# 965.8443 –(A)
-Gal

## Slide 119
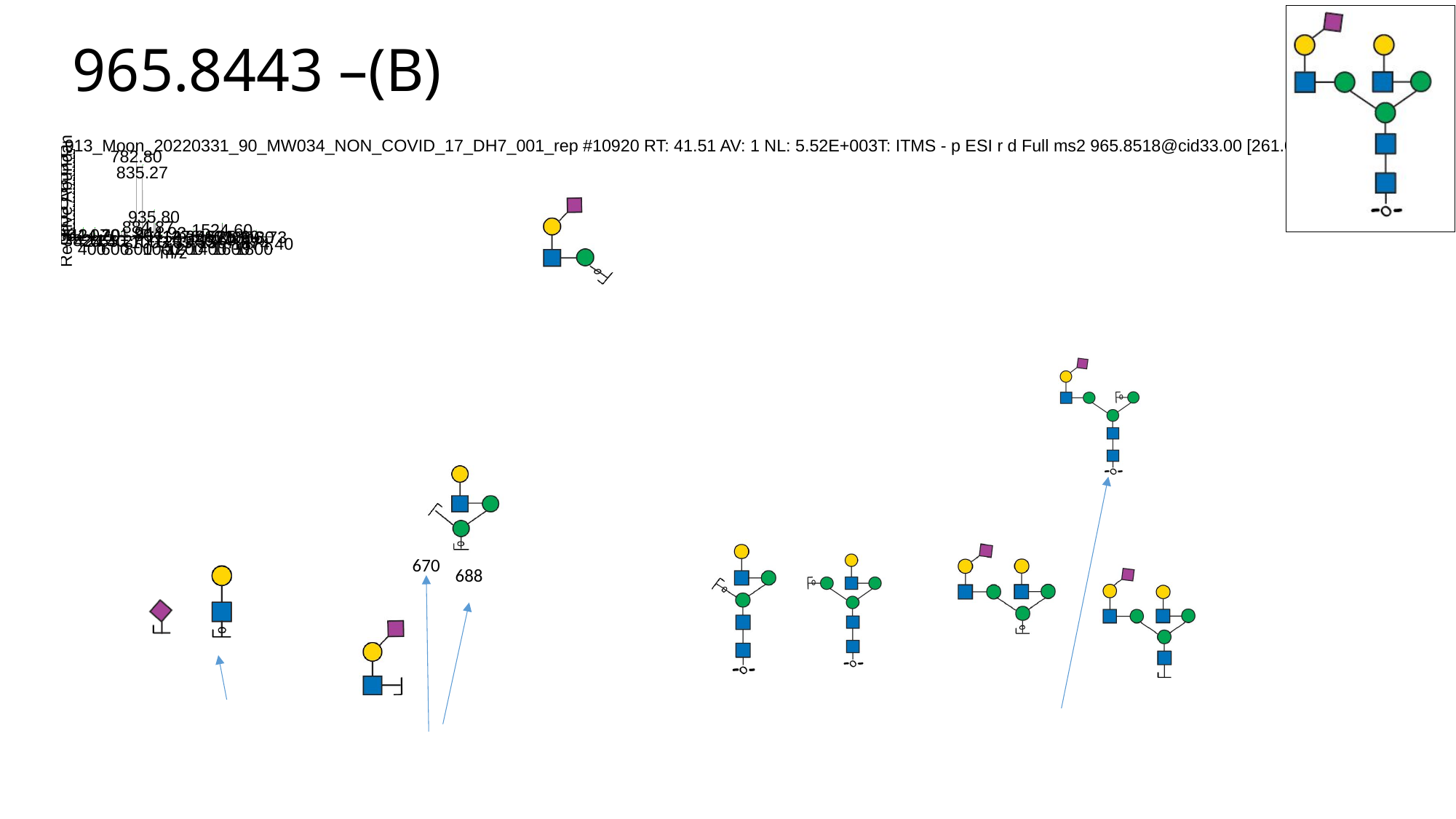

# 965.8443 –(B)
670
688

## Slide 120
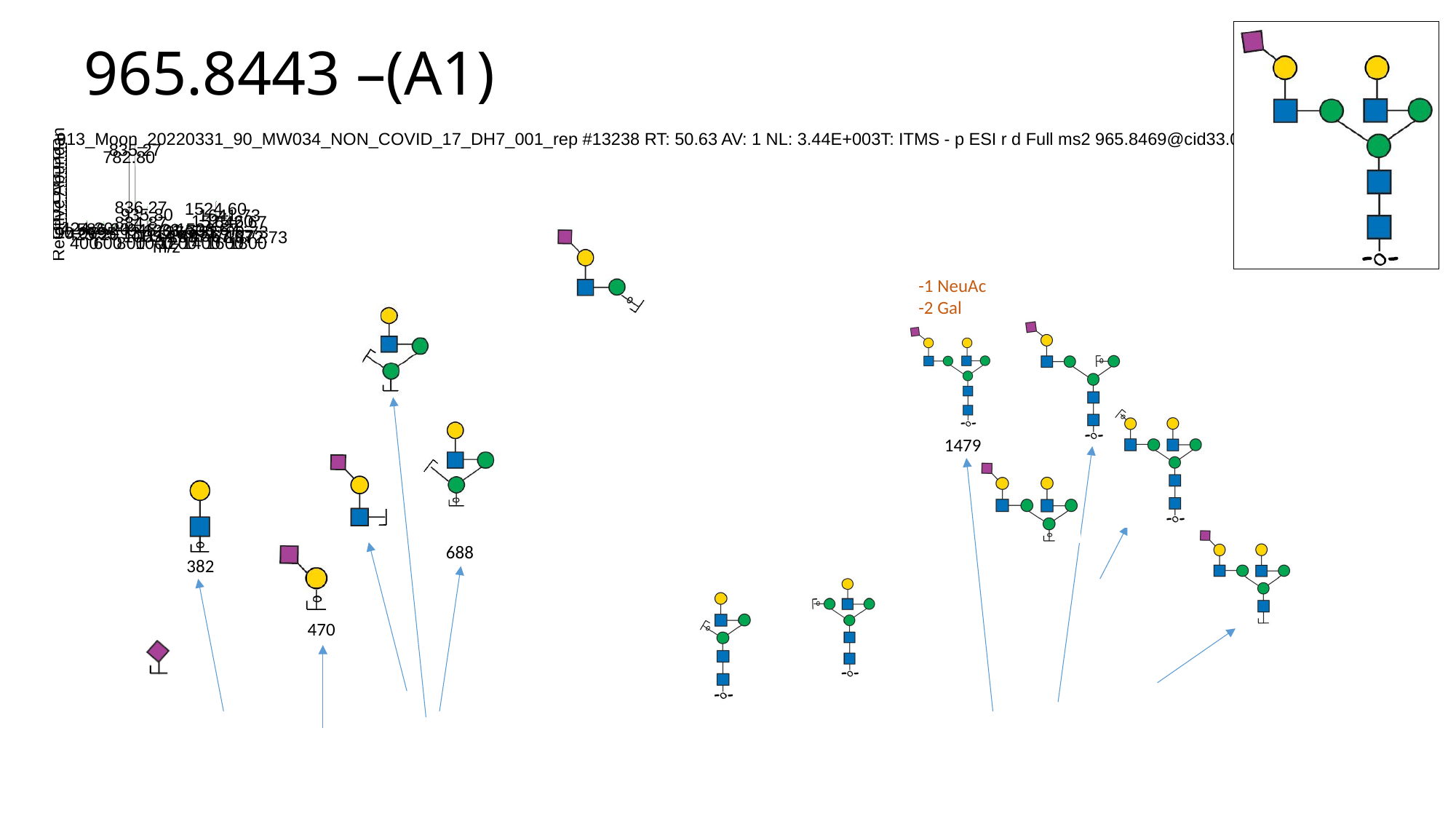

# 965.8443 –(A1)
-1 NeuAc
-2 Gal
1479
688
382
470

## Slide 121
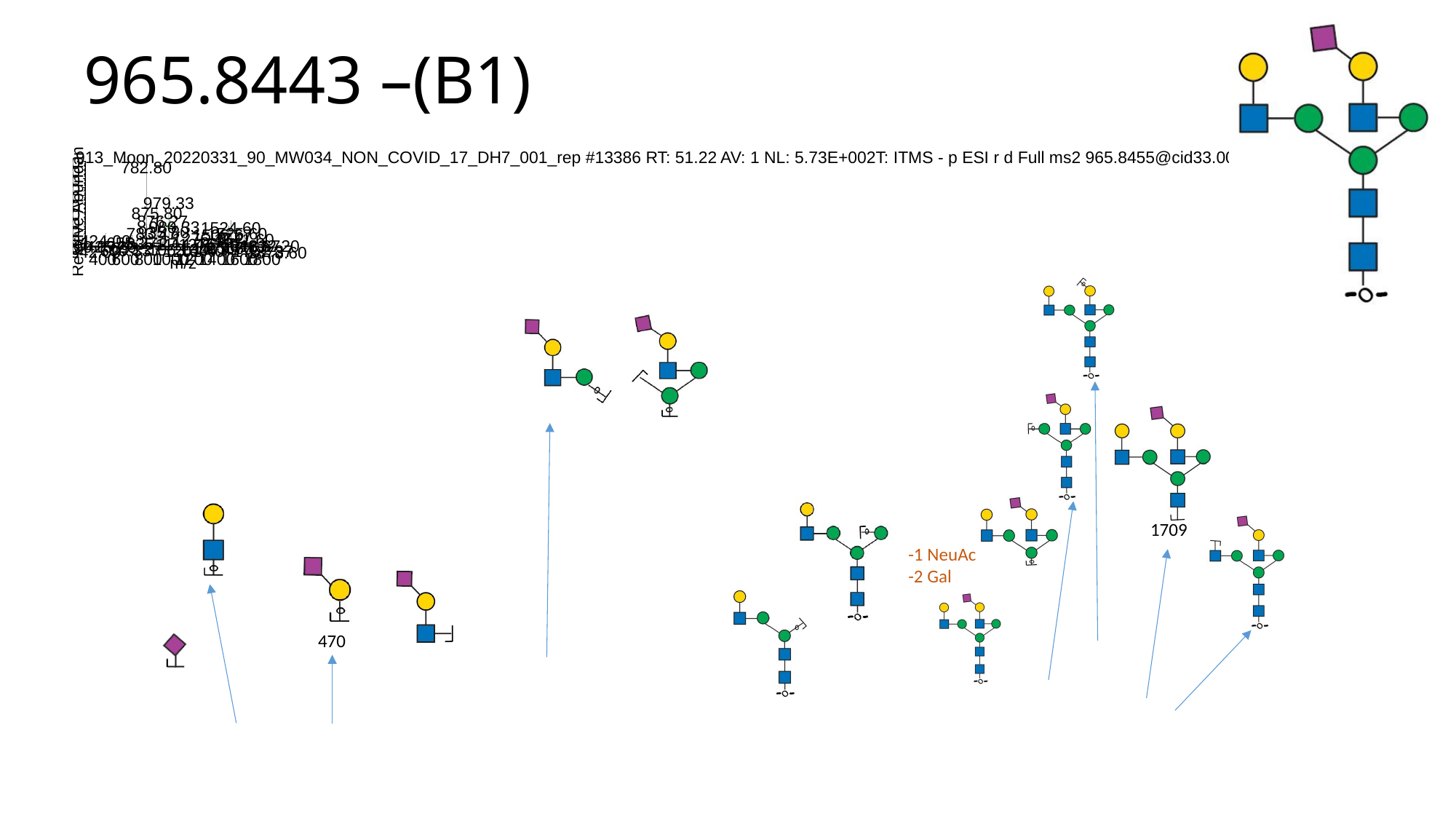

# 965.8443 –(B1)
1709
-1 NeuAc
-2 Gal
470

## Slide 122
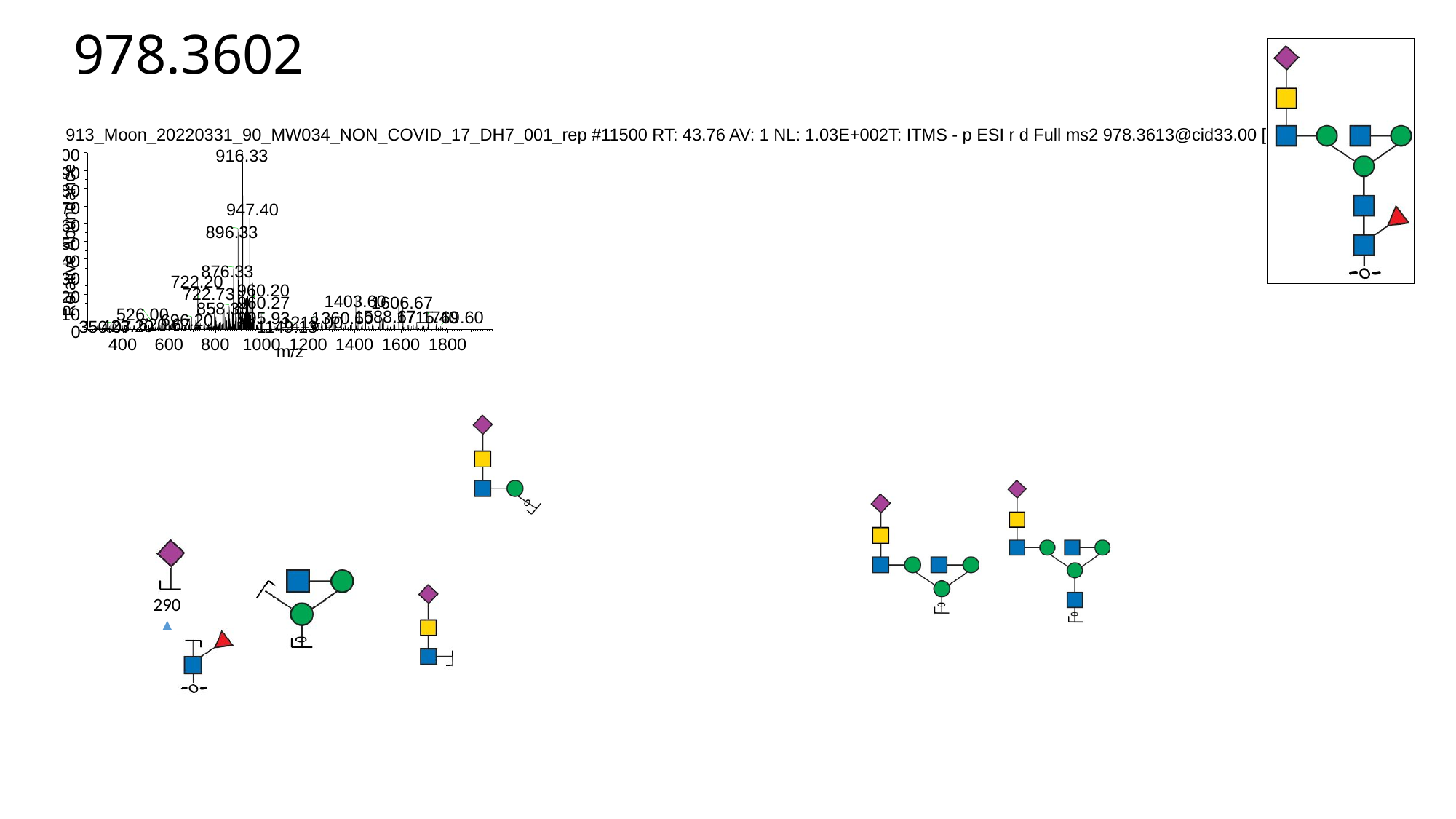

# 978.3602
290

## Slide 123
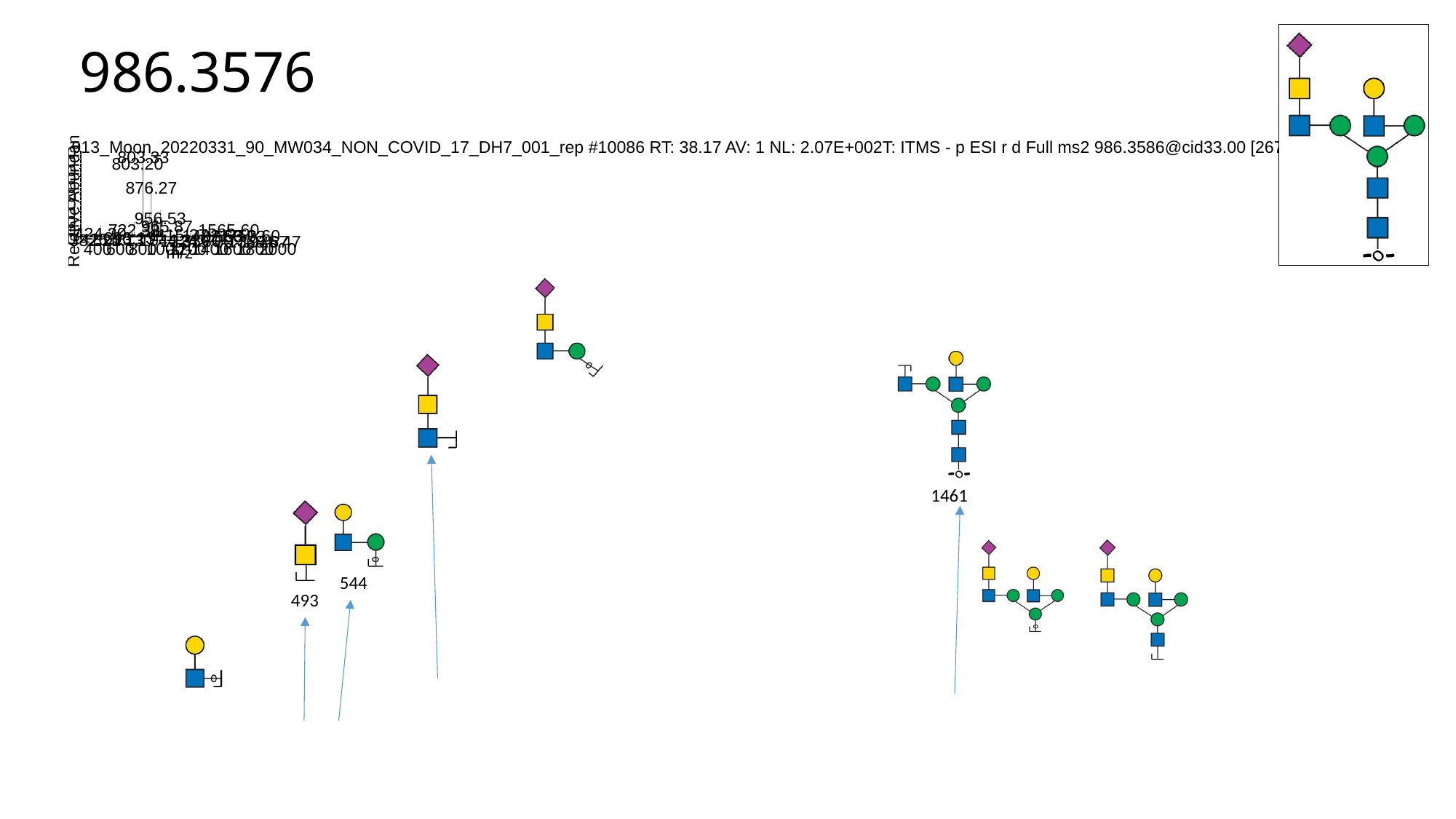

# 986.3576
1461
544
493

## Slide 124
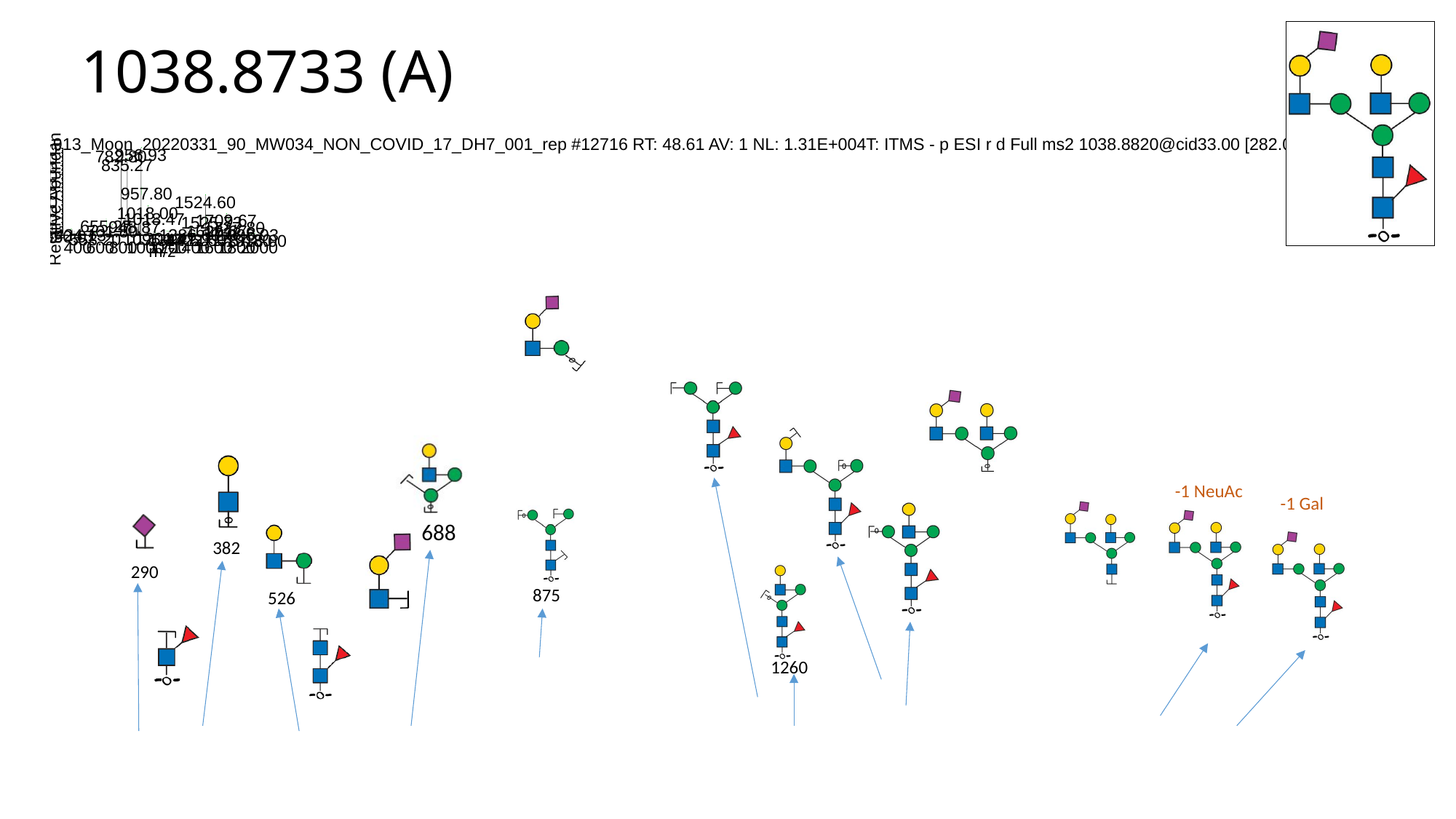

# 1038.8733 (A)
-1 NeuAc
-1 Gal
688
382
290
875
526
1260

## Slide 125
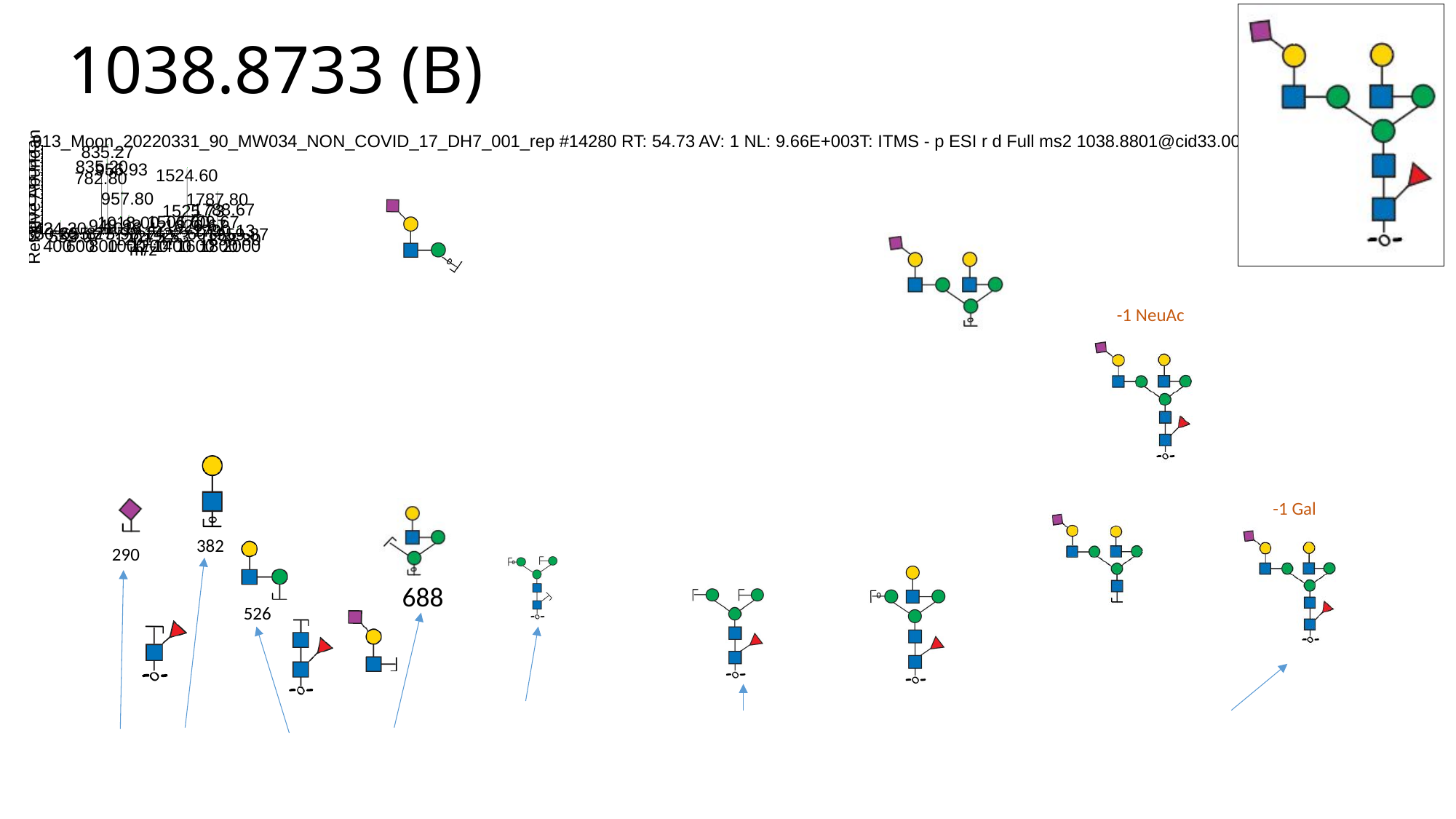

# 1038.8733 (B)
-1 NeuAc
-1 Gal
382
290
688
526

## Slide 126
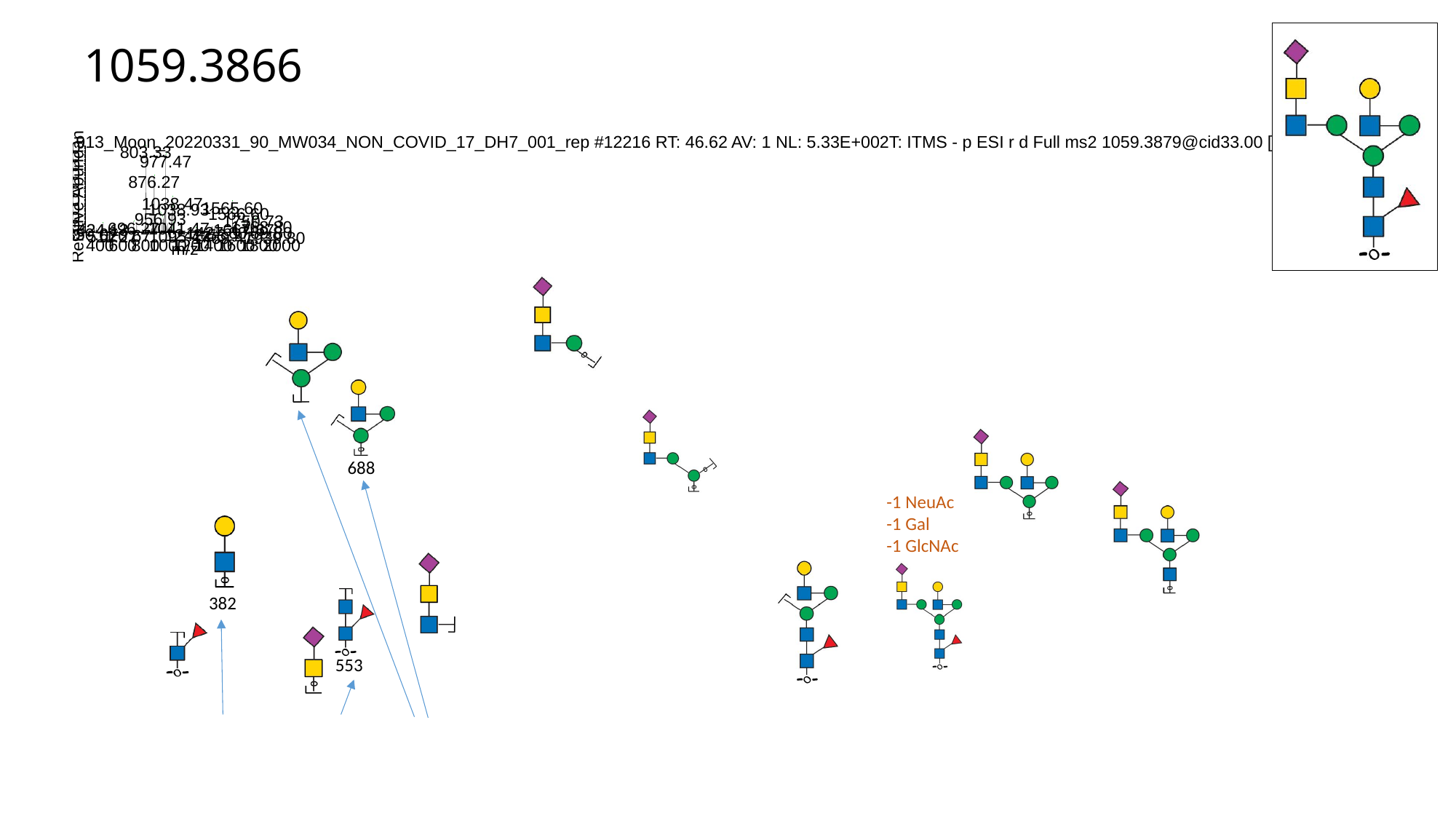

# 1059.3866
688
-1 NeuAc
-1 Gal
-1 GlcNAc
382
553

## Slide 127
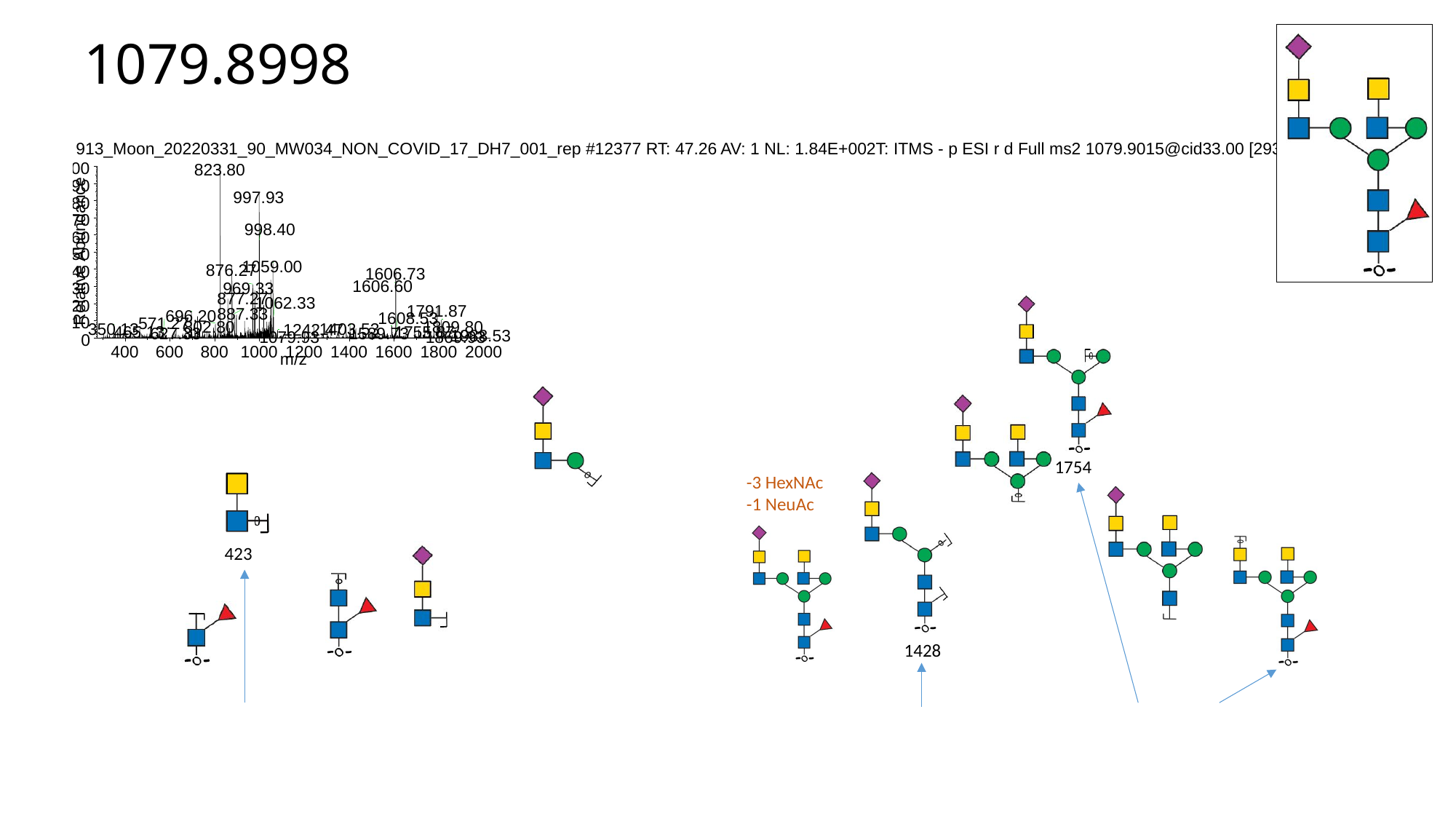

# 1079.8998
1754
-3 HexNAc
-1 NeuAc
423
1428

## Slide 128
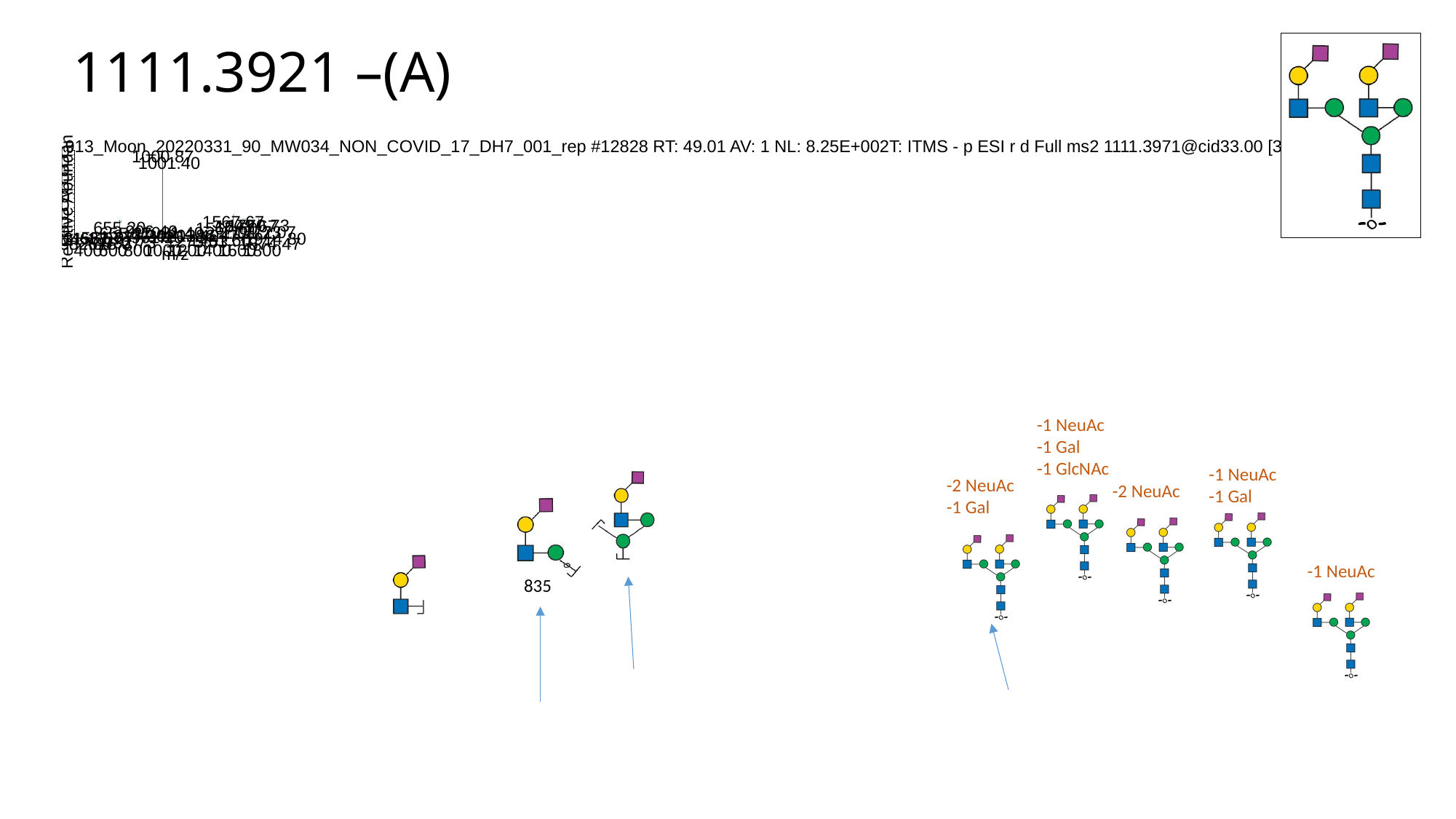

# 1111.3921 –(A)
-1 NeuAc
-1 Gal
-1 GlcNAc
-1 NeuAc
-1 Gal
-2 NeuAc
-1 Gal
-2 NeuAc
-1 NeuAc
835

## Slide 129
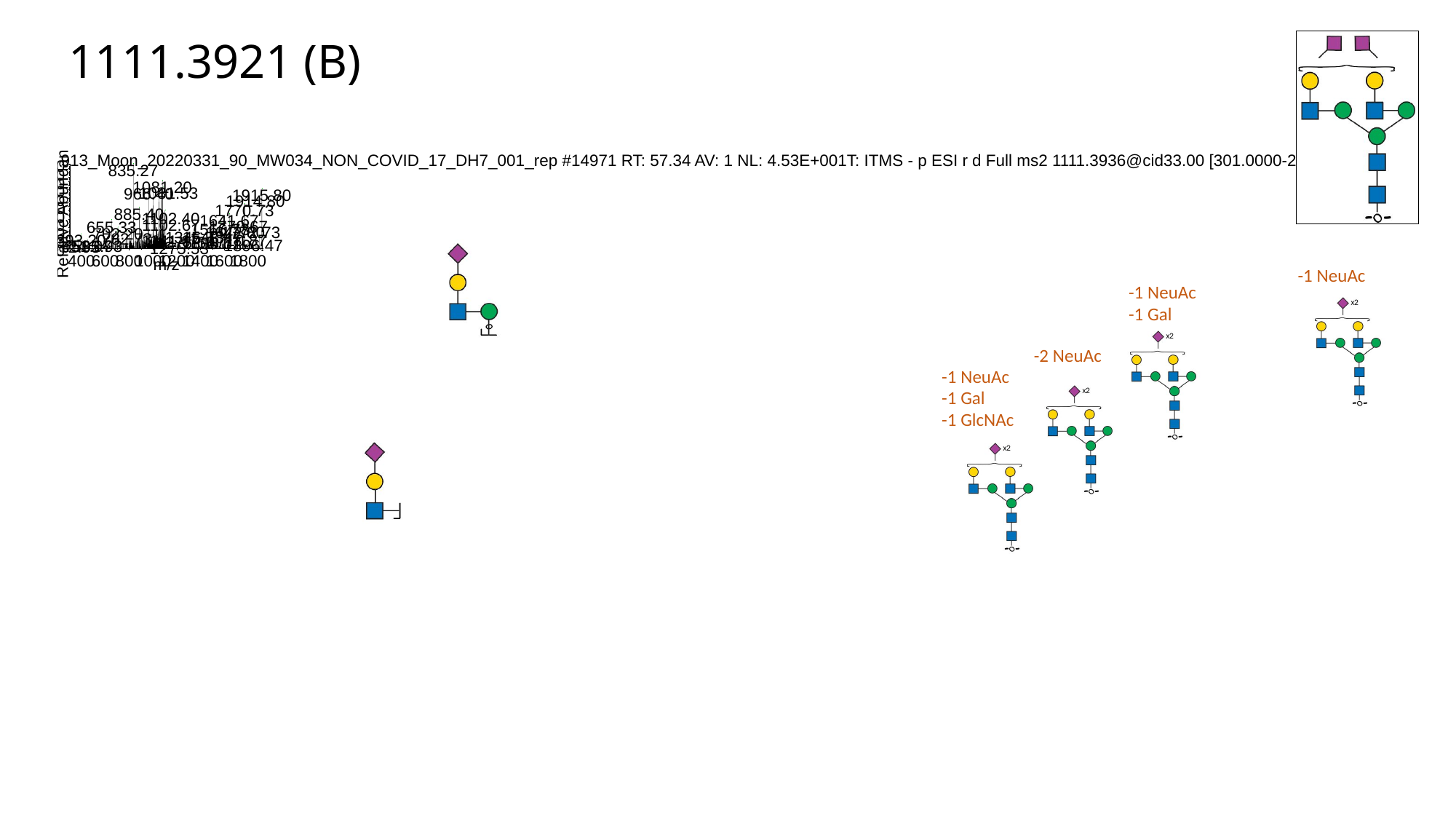

# 1111.3921 (B)
-1 NeuAc
-1 NeuAc
-1 Gal
-2 NeuAc
-1 NeuAc
-1 Gal
-1 GlcNAc

## Slide 130
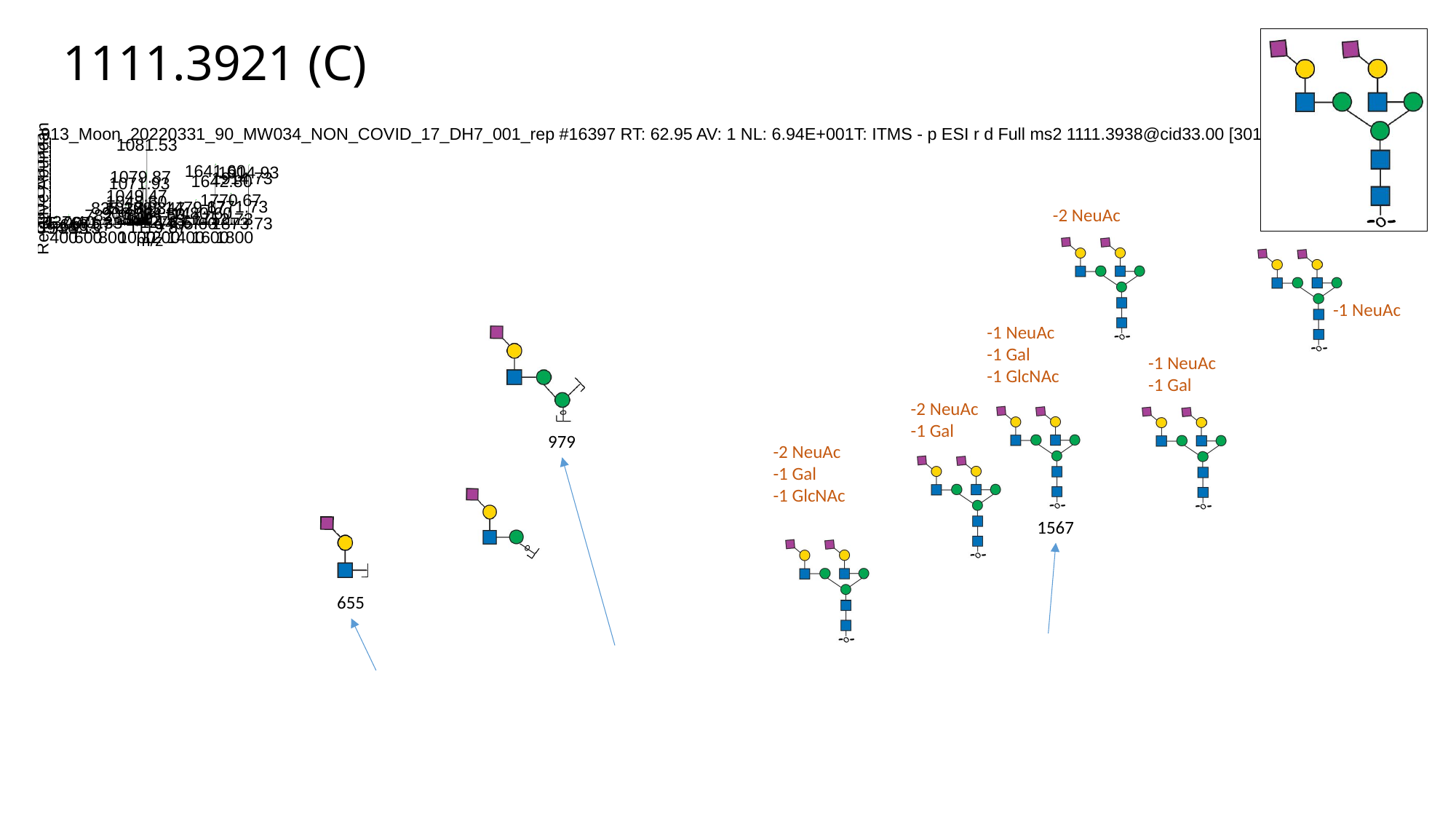

# 1111.3921 (C)
-2 NeuAc
-1 NeuAc
-1 NeuAc
-1 Gal
-1 GlcNAc
-1 NeuAc
-1 Gal
-2 NeuAc
-1 Gal
979
-2 NeuAc
-1 Gal
-1 GlcNAc
1567
655

## Slide 131
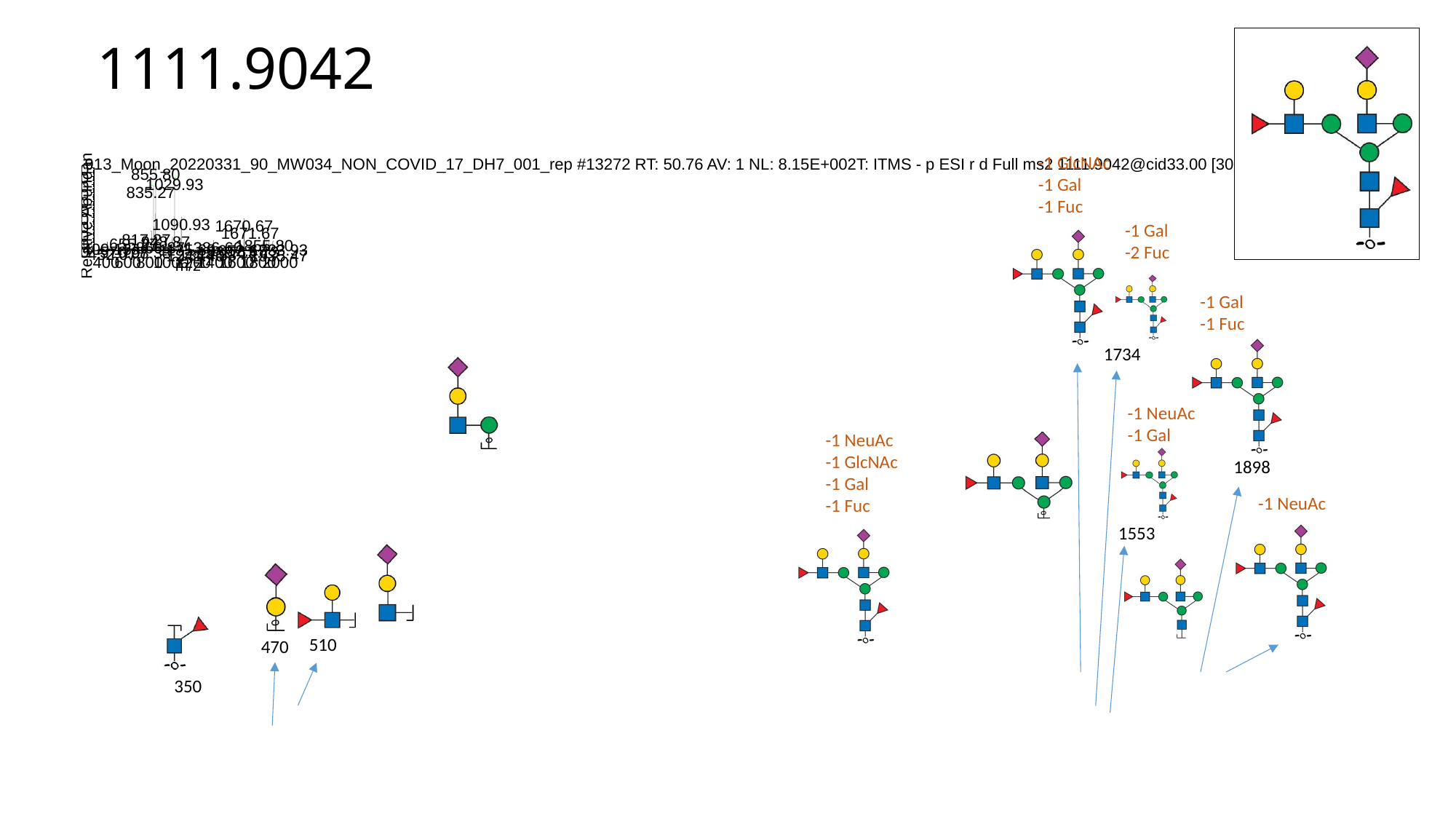

# 1111.9042
-1 GlcNAc
-1 Gal
-1 Fuc
-1 Gal
-2 Fuc
-1 Gal
-1 Fuc
1734
-1 NeuAc
-1 Gal
-1 NeuAc
-1 GlcNAc
-1 Gal
-1 Fuc
1898
-1 NeuAc
1553
510
470
350

## Slide 132
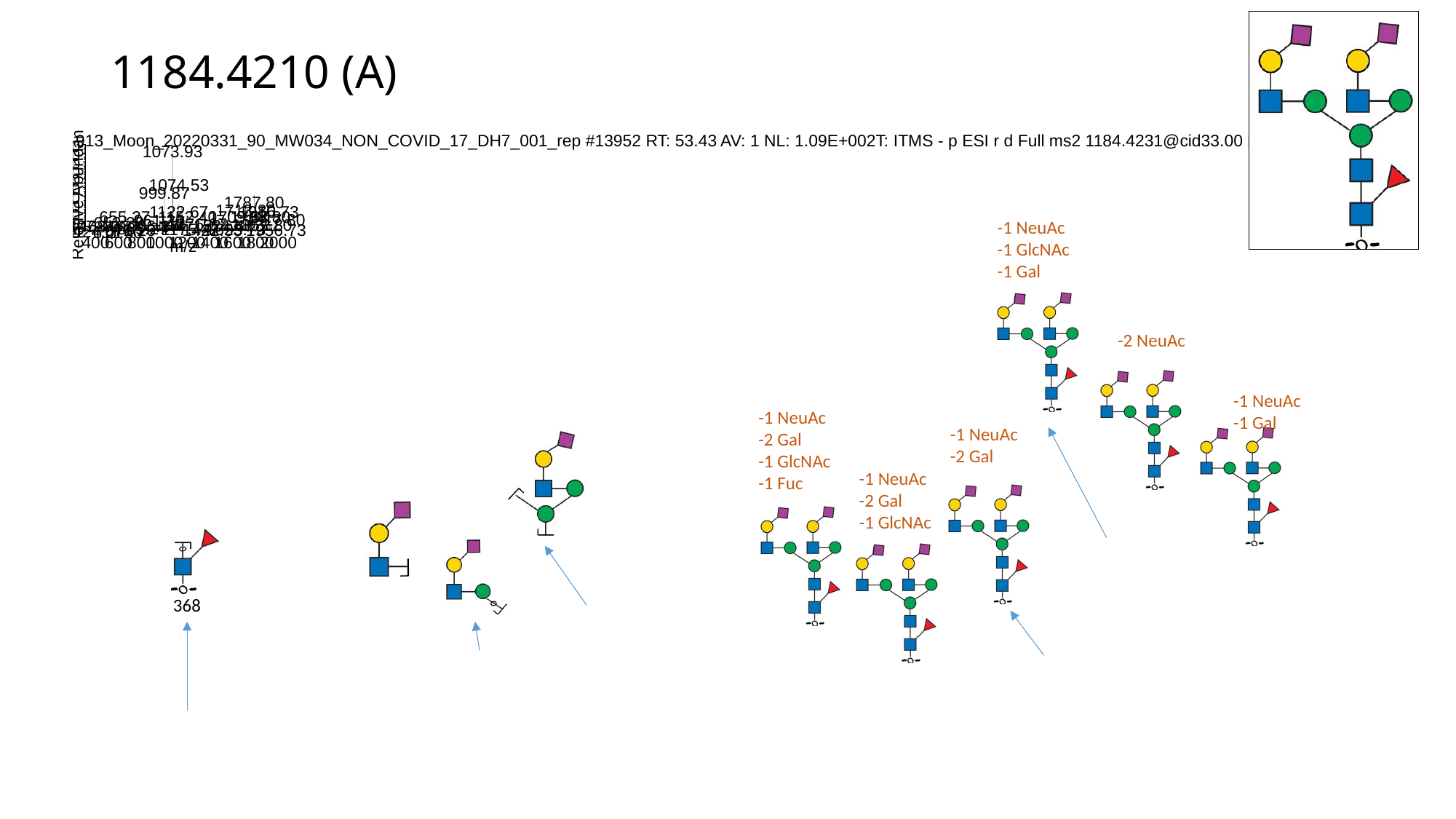

# 1184.4210 (A)
-1 NeuAc
-1 GlcNAc
-1 Gal
-2 NeuAc
-1 NeuAc
-1 Gal
-1 NeuAc
-2 Gal
-1 GlcNAc
-1 Fuc
-1 NeuAc
-2 Gal
-1 NeuAc
-2 Gal
-1 GlcNAc
368

## Slide 133
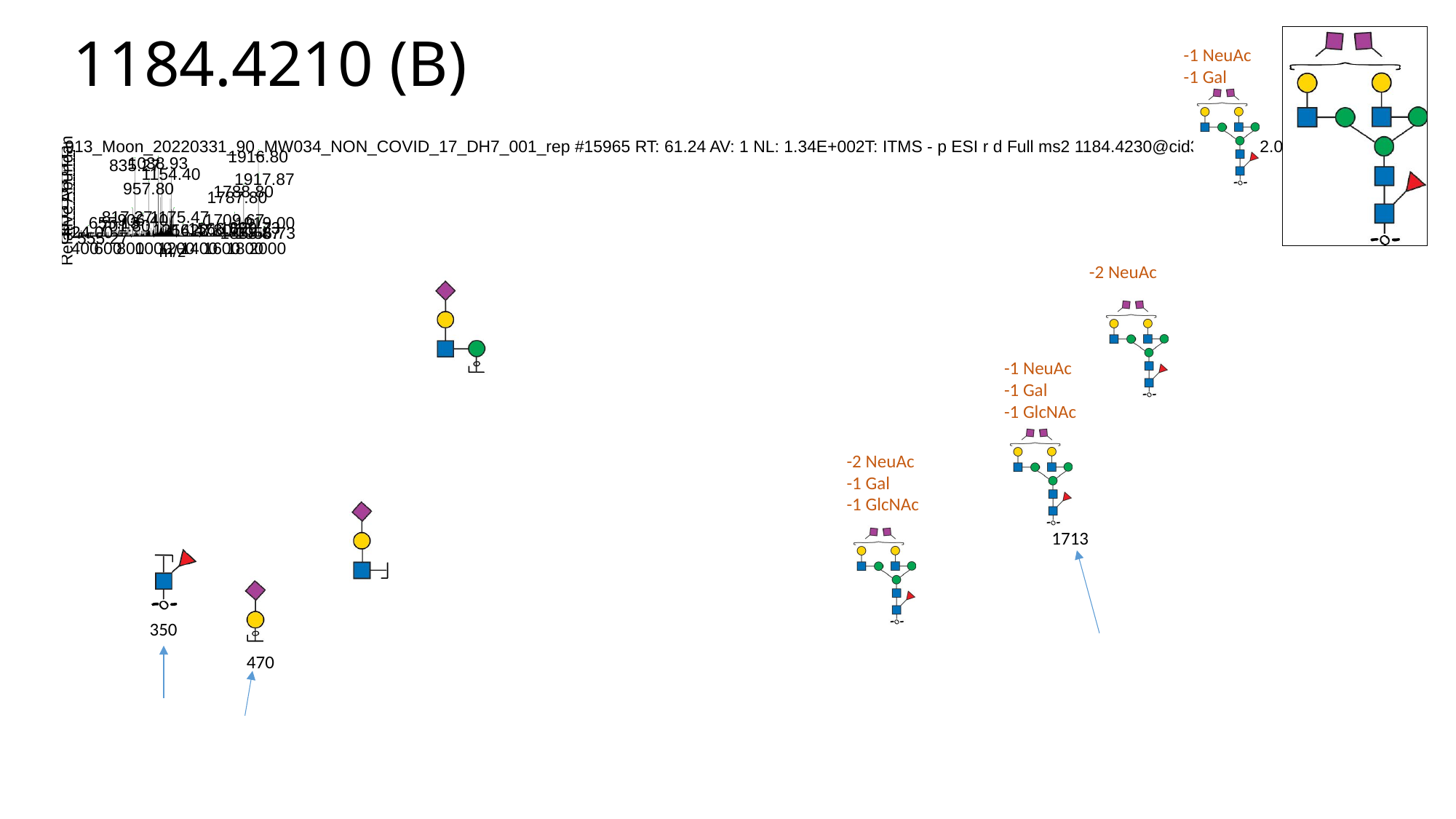

# 1184.4210 (B)
-1 NeuAc
-1 Gal
-2 NeuAc
-1 NeuAc
-1 Gal
-1 GlcNAc
-2 NeuAc
-1 Gal
-1 GlcNAc
1713
350
470

## Slide 134
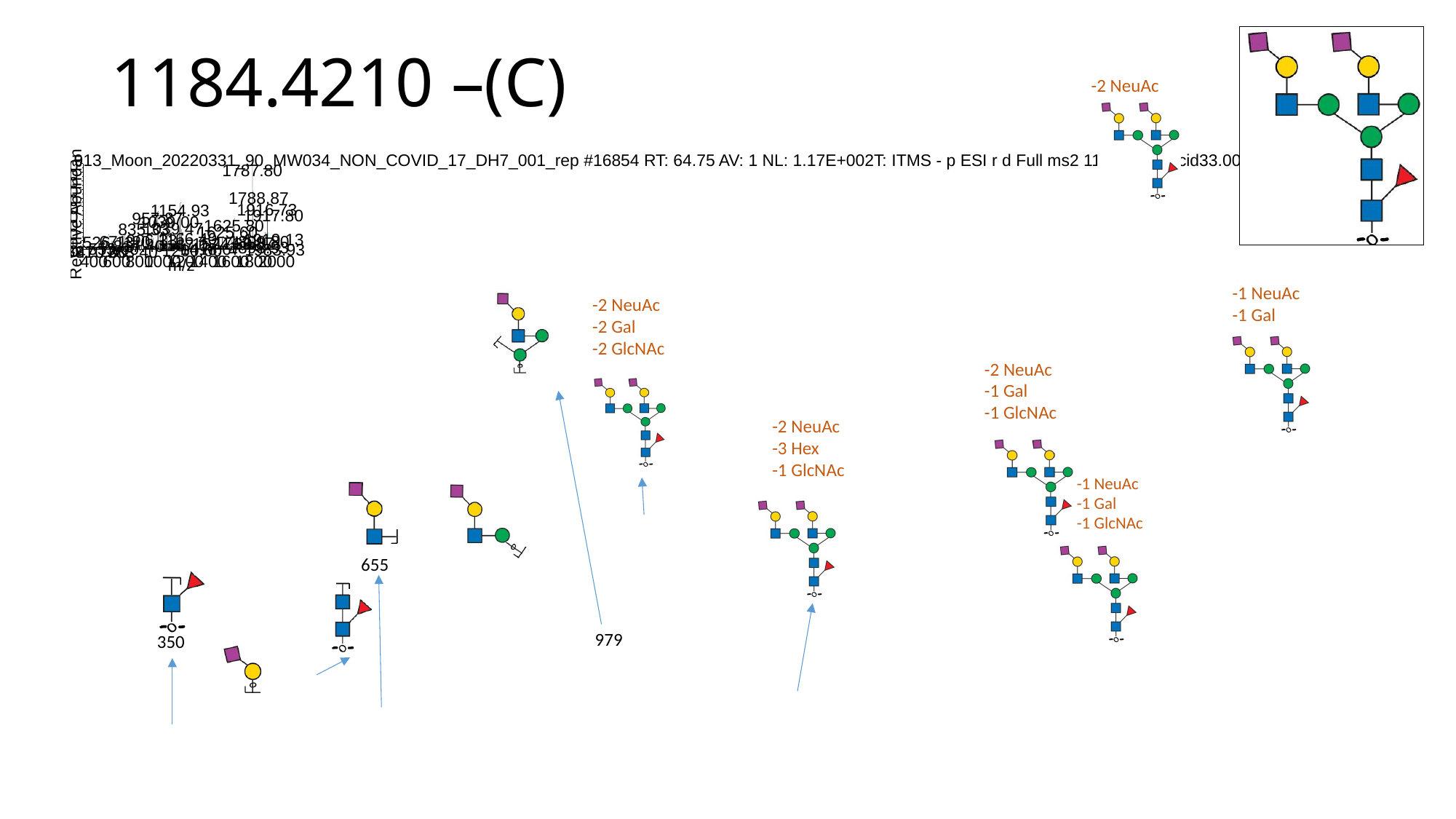

# 1184.4210 –(C)
-2 NeuAc
-1 NeuAc
-1 Gal
-2 NeuAc
-2 Gal
-2 GlcNAc
-2 NeuAc
-1 Gal
-1 GlcNAc
-2 NeuAc
-3 Hex
-1 GlcNAc
-1 NeuAc
-1 Gal
-1 GlcNAc
655
979
350

## Slide 135
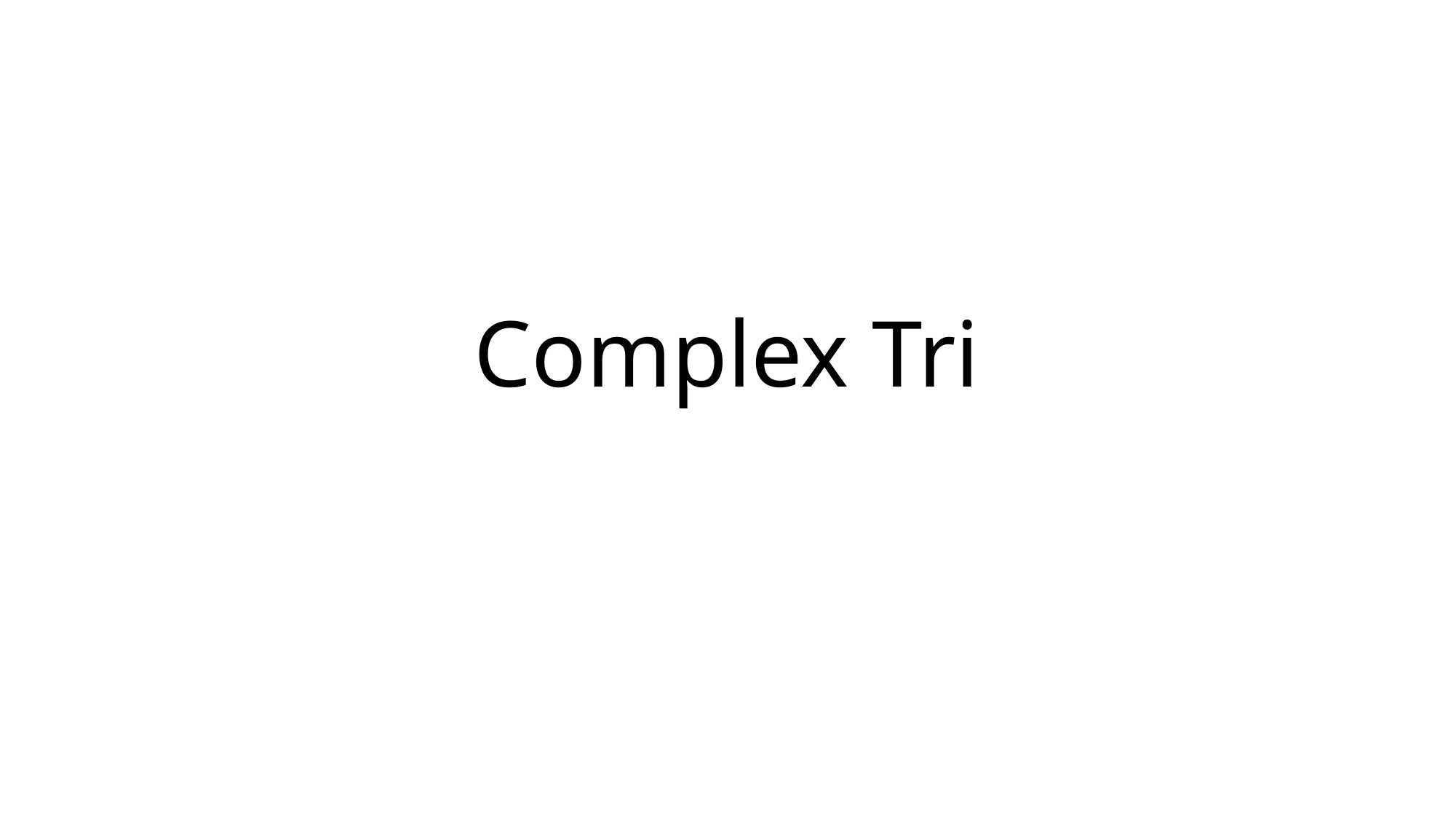

# Complex Tri

## Slide 136
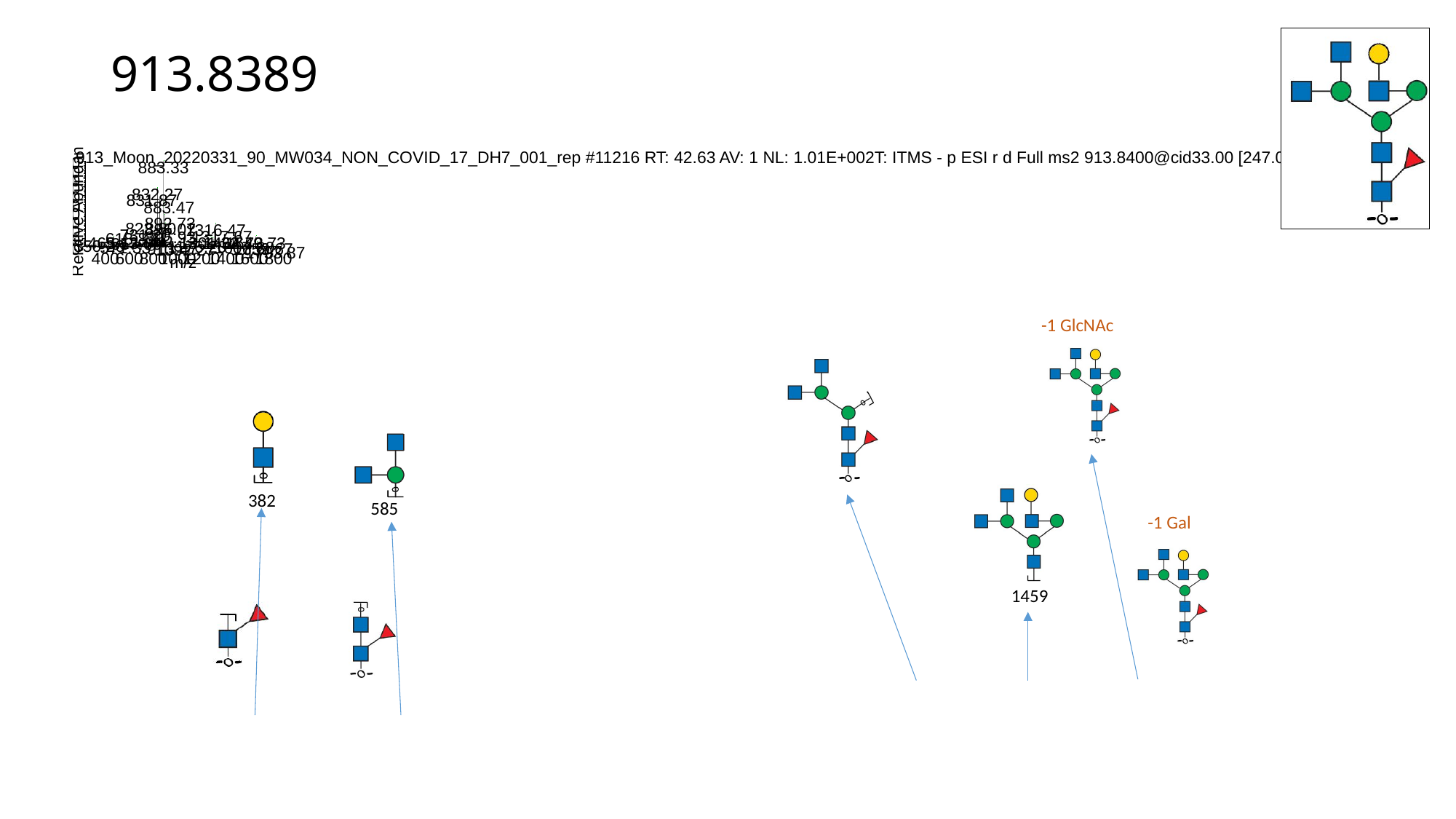

# 913.8389
-1 GlcNAc
382
585
-1 Gal
1459

## Slide 137
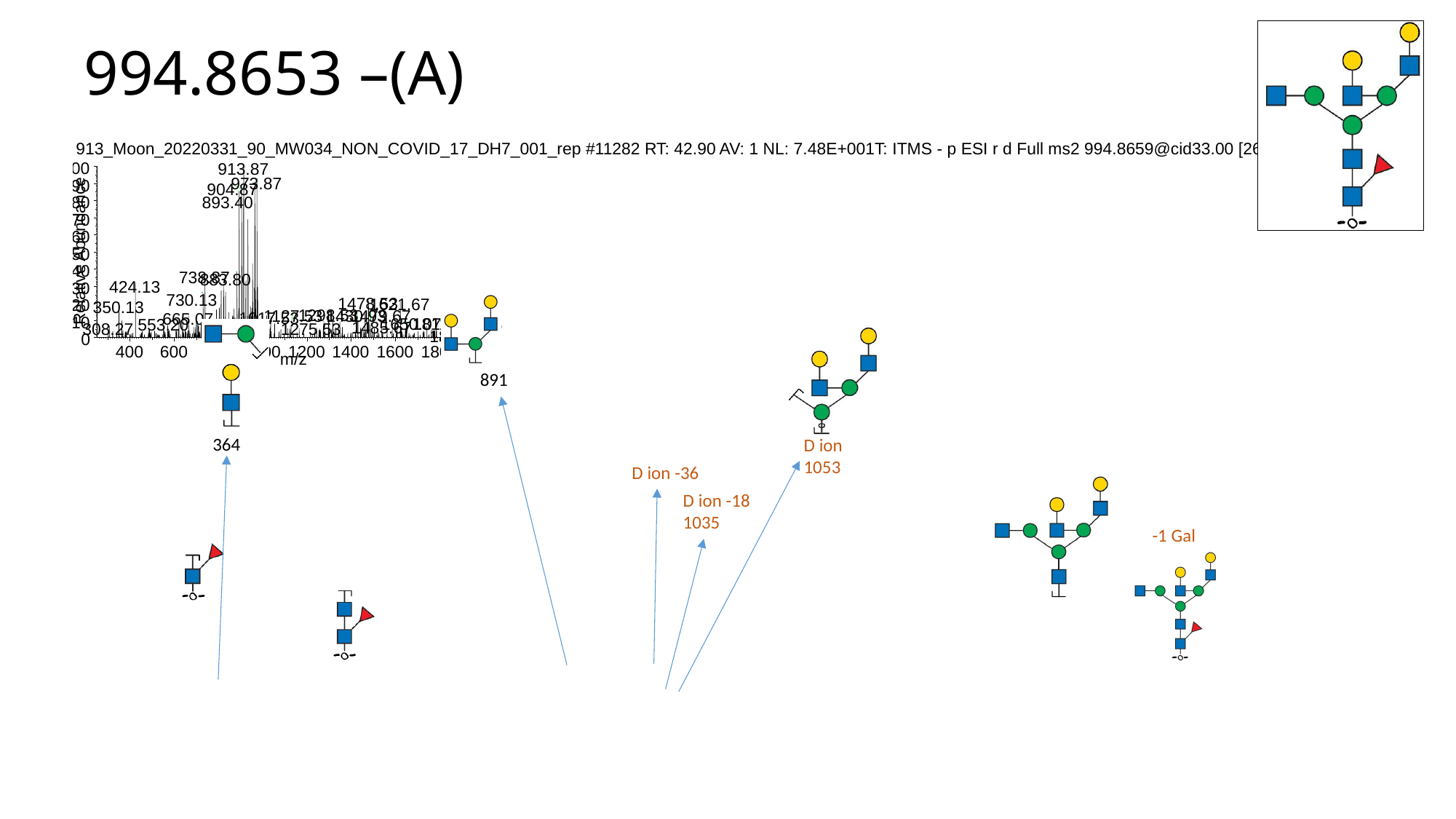

# 994.8653 –(A)
891
364
D ion
1053
D ion -36
D ion -18
1035
-1 Gal

## Slide 138
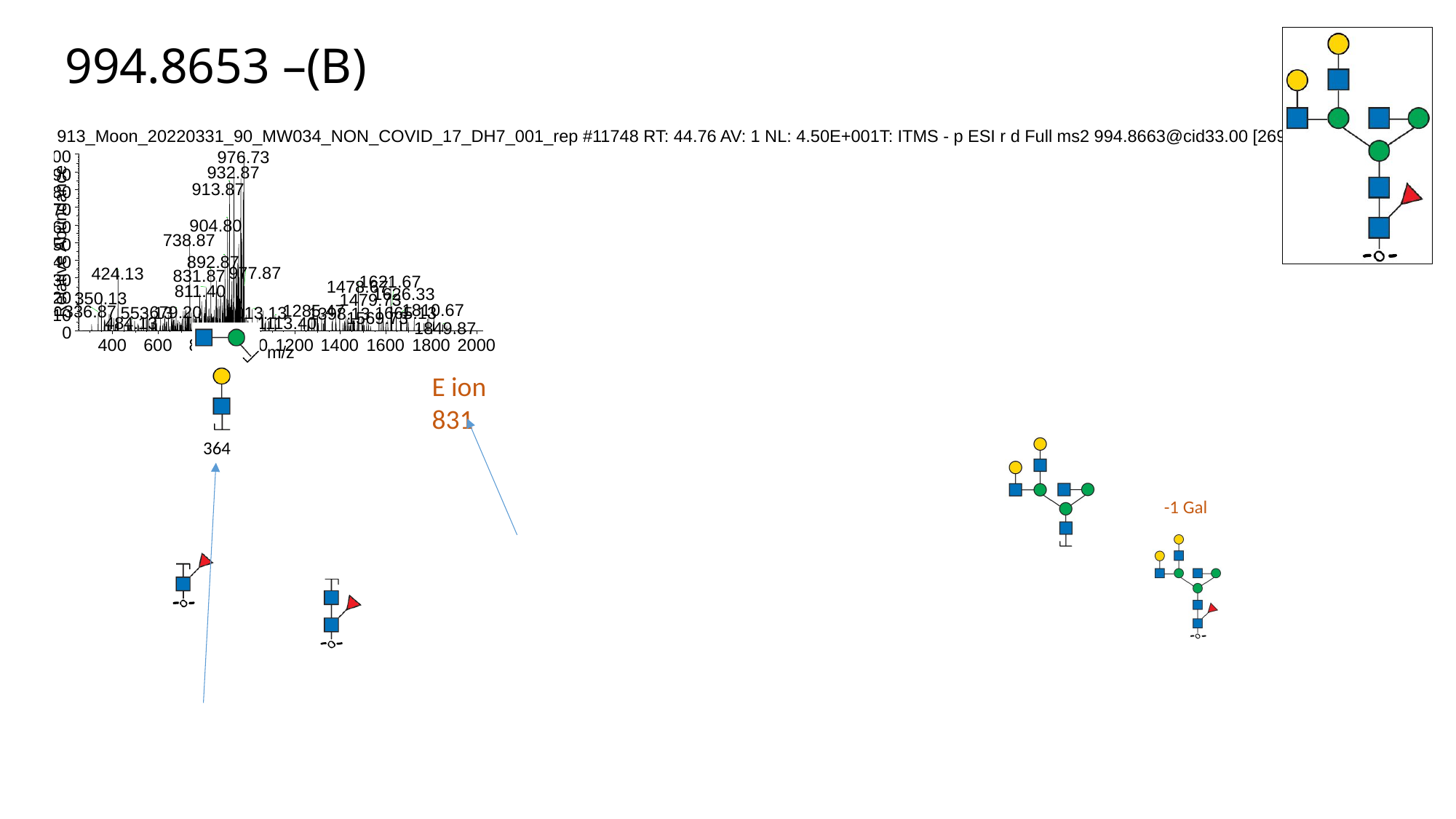

# 994.8653 –(B)
E ion
831
364
-1 Gal

## Slide 139
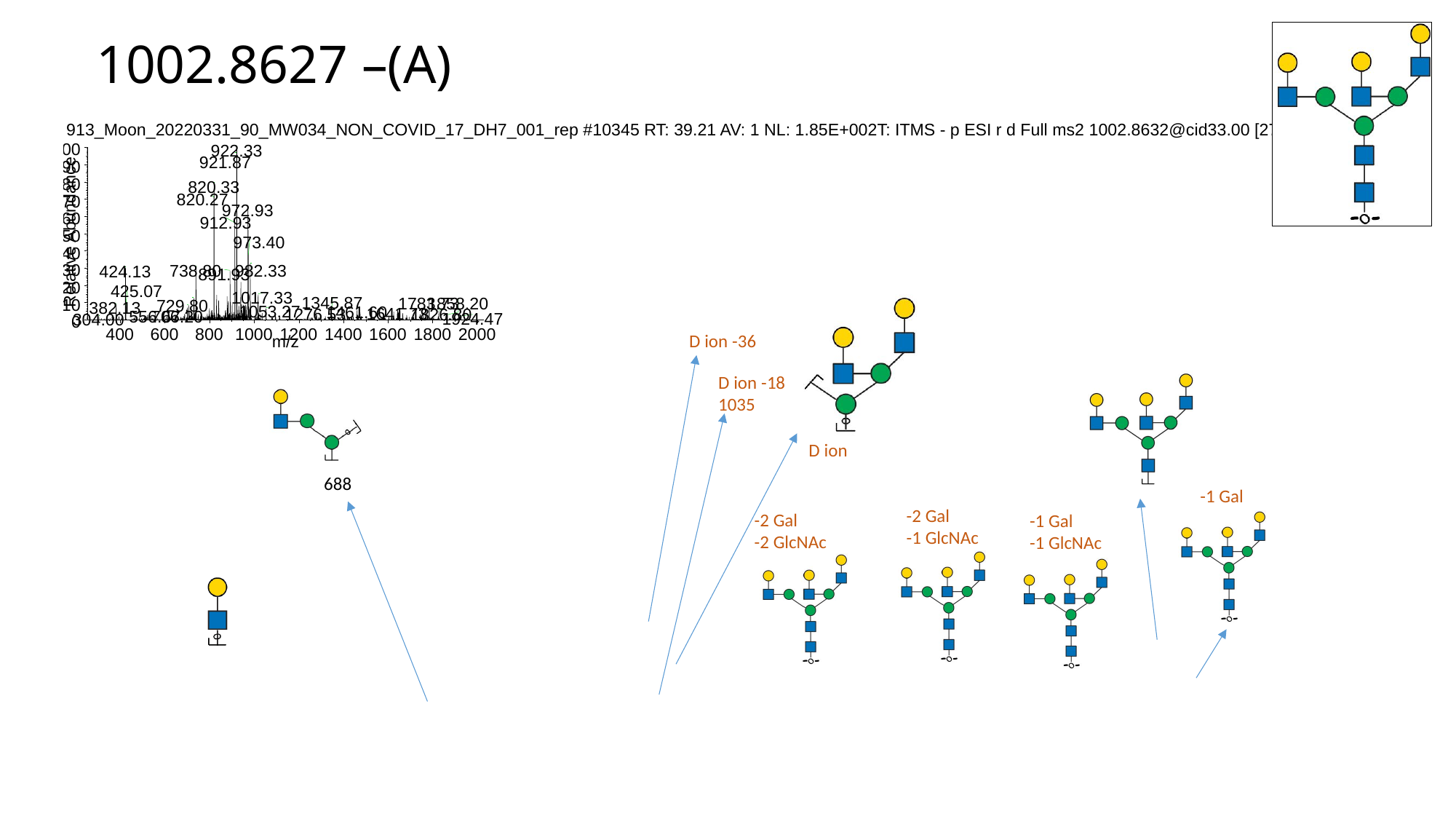

# 1002.8627 –(A)
D ion -36
D ion -18
1035
D ion
688
-1 Gal
-2 Gal
-1 GlcNAc
-2 Gal
-2 GlcNAc
-1 Gal
-1 GlcNAc

## Slide 140
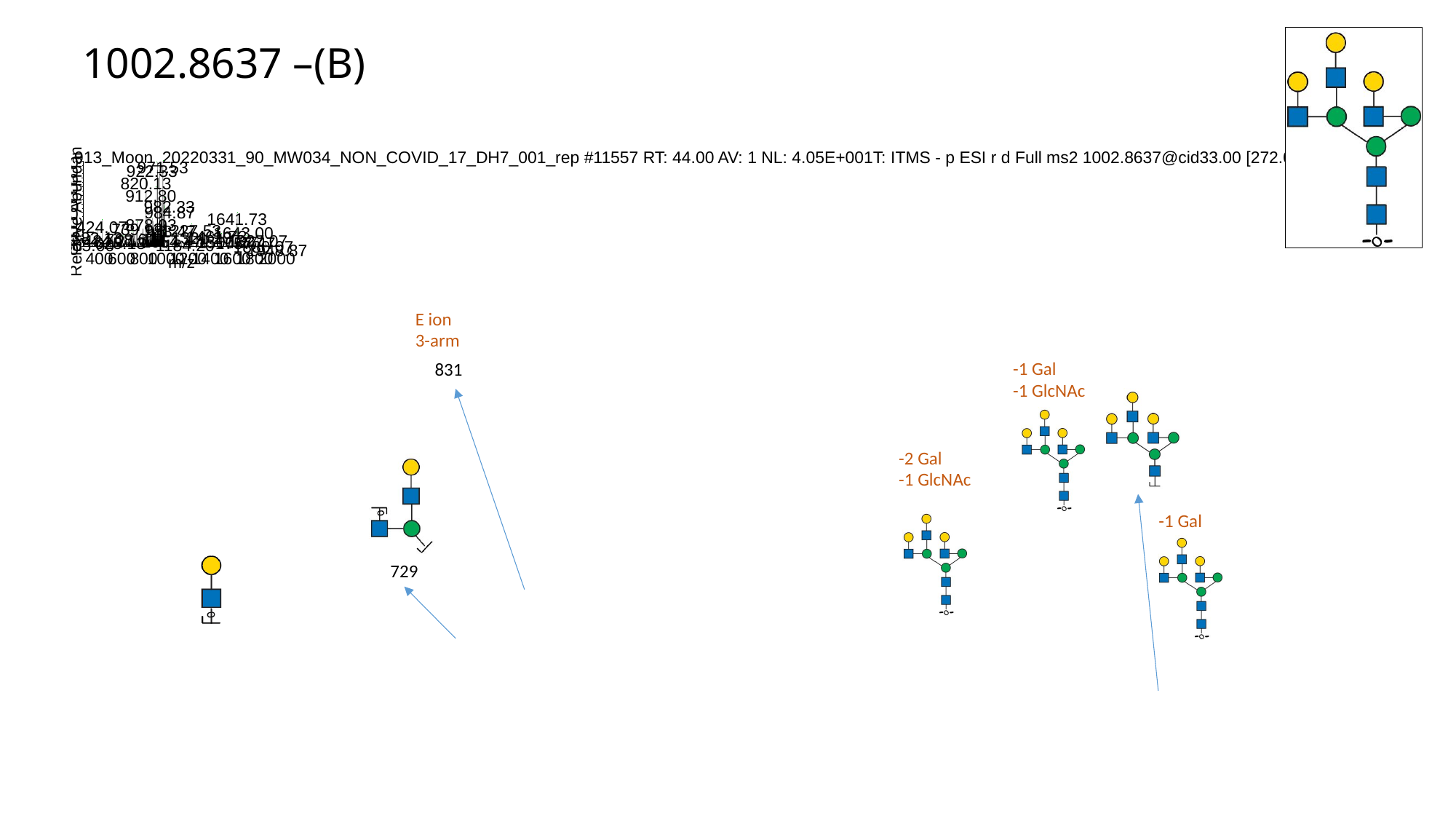

# 1002.8637 –(B)
E ion
3-arm
831
-1 Gal
-1 GlcNAc
-2 Gal
-1 GlcNAc
-1 Gal
729

## Slide 141
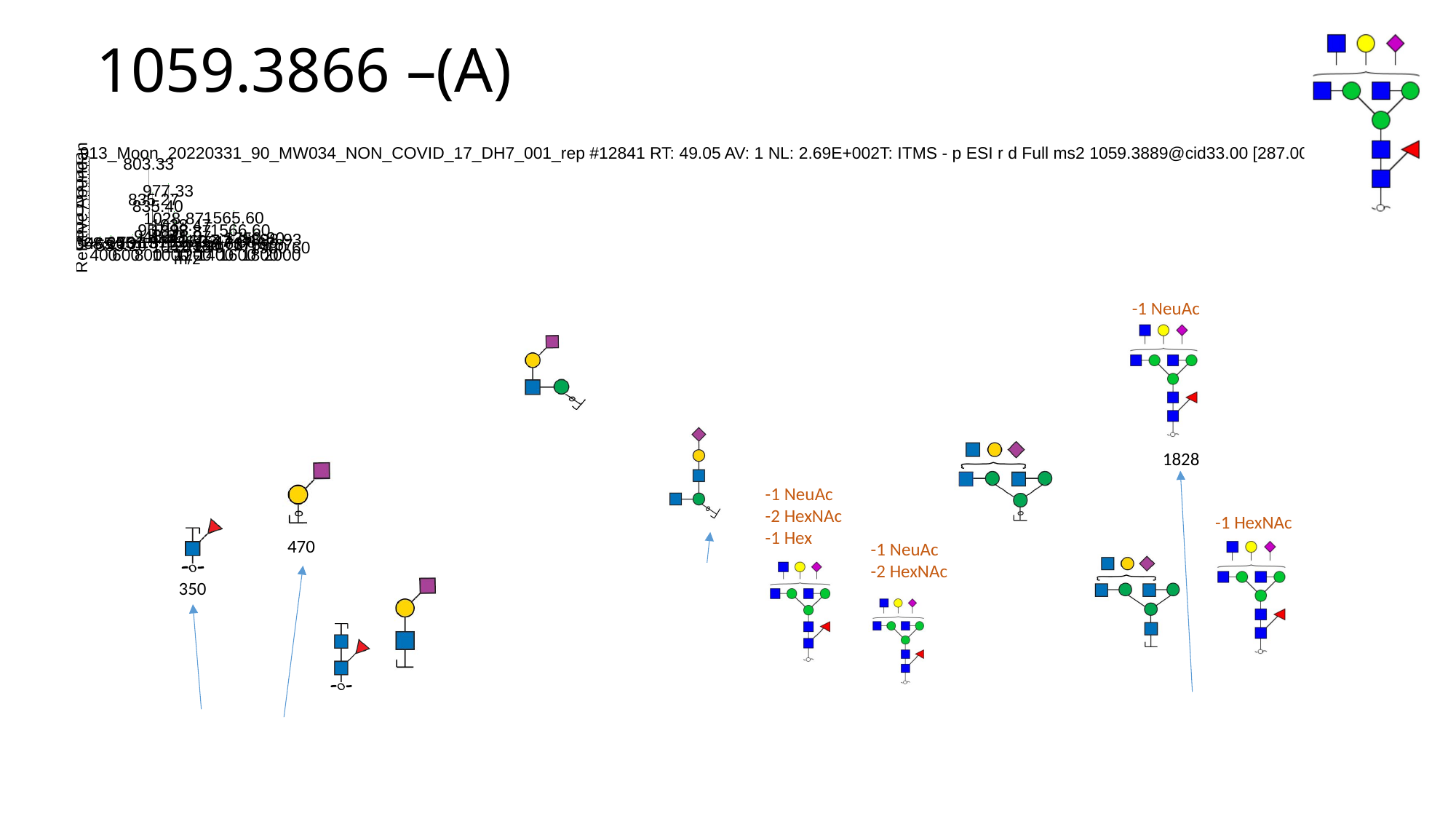

# 1059.3866 –(A)
-1 NeuAc
1828
-1 NeuAc
-2 HexNAc
-1 Hex
-1 HexNAc
470
-1 NeuAc
-2 HexNAc
350

## Slide 142
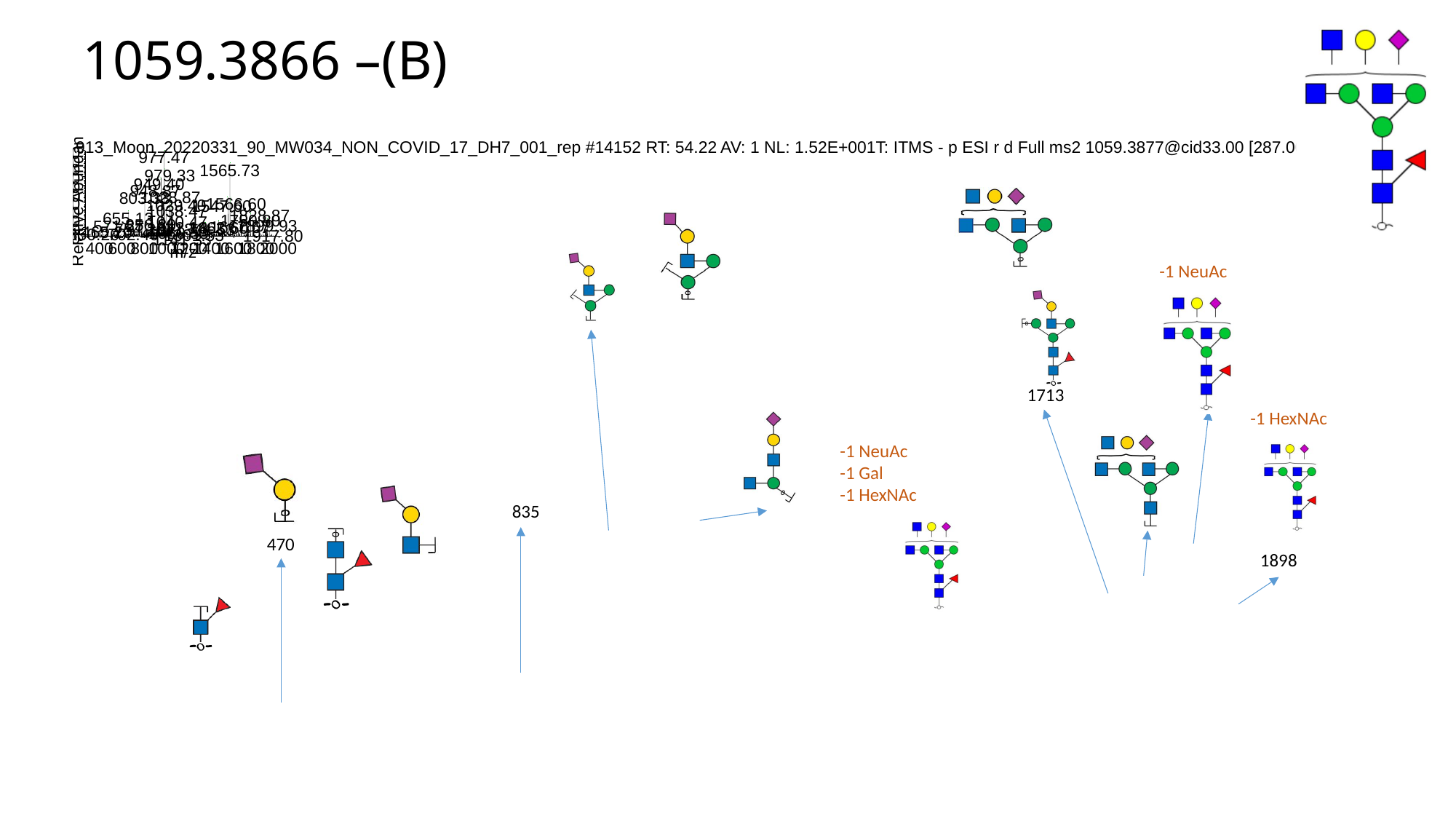

# 1059.3866 –(B)
-1 NeuAc
1713
-1 HexNAc
-1 NeuAc
-1 Gal
-1 HexNAc
835
470
1898

## Slide 143
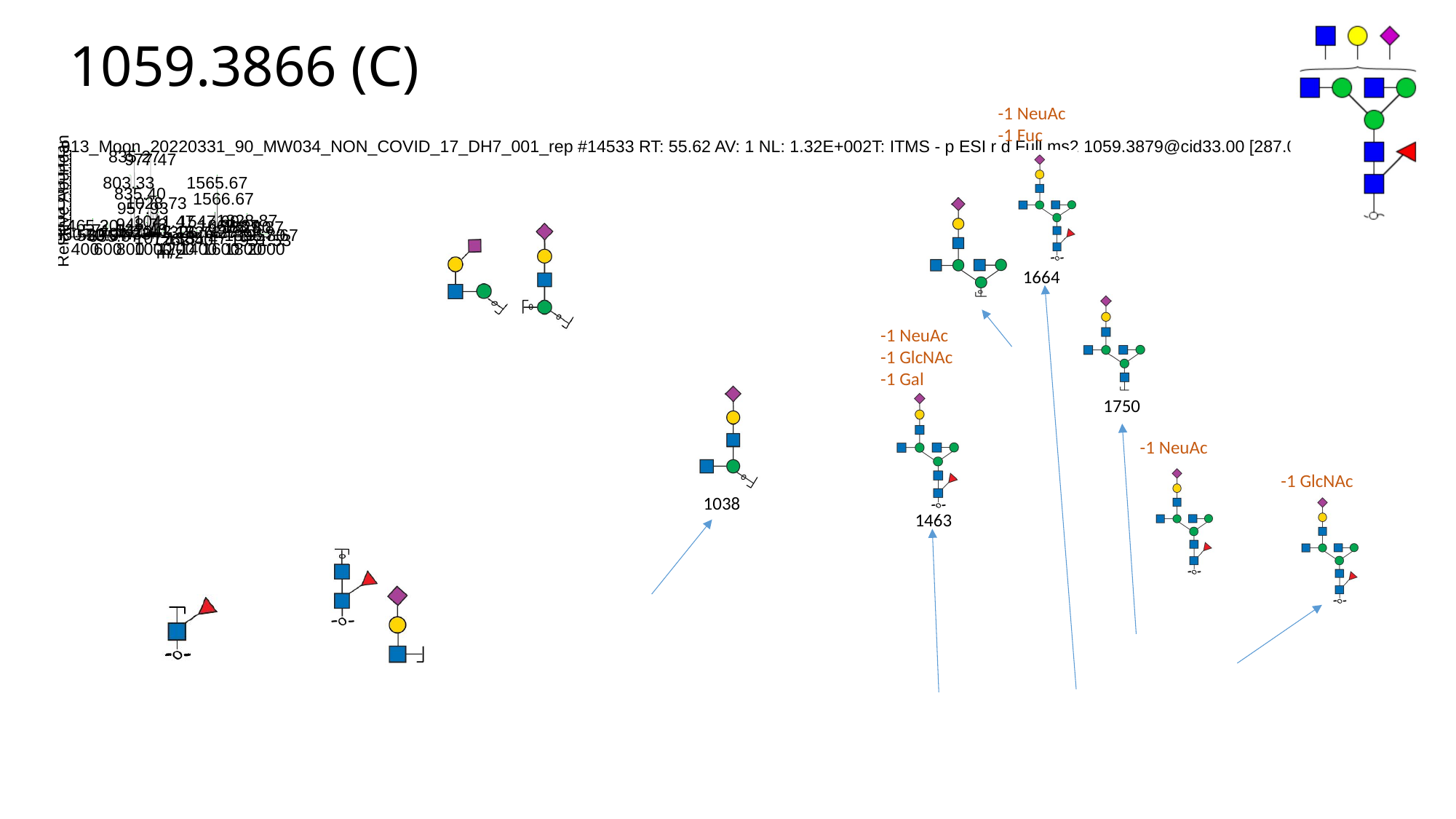

# 1059.3866 (C)
-1 NeuAc
-1 Fuc
1664
-1 NeuAc
-1 GlcNAc
-1 Gal
1750
-1 NeuAc
-1 GlcNAc
1038
1463

## Slide 144
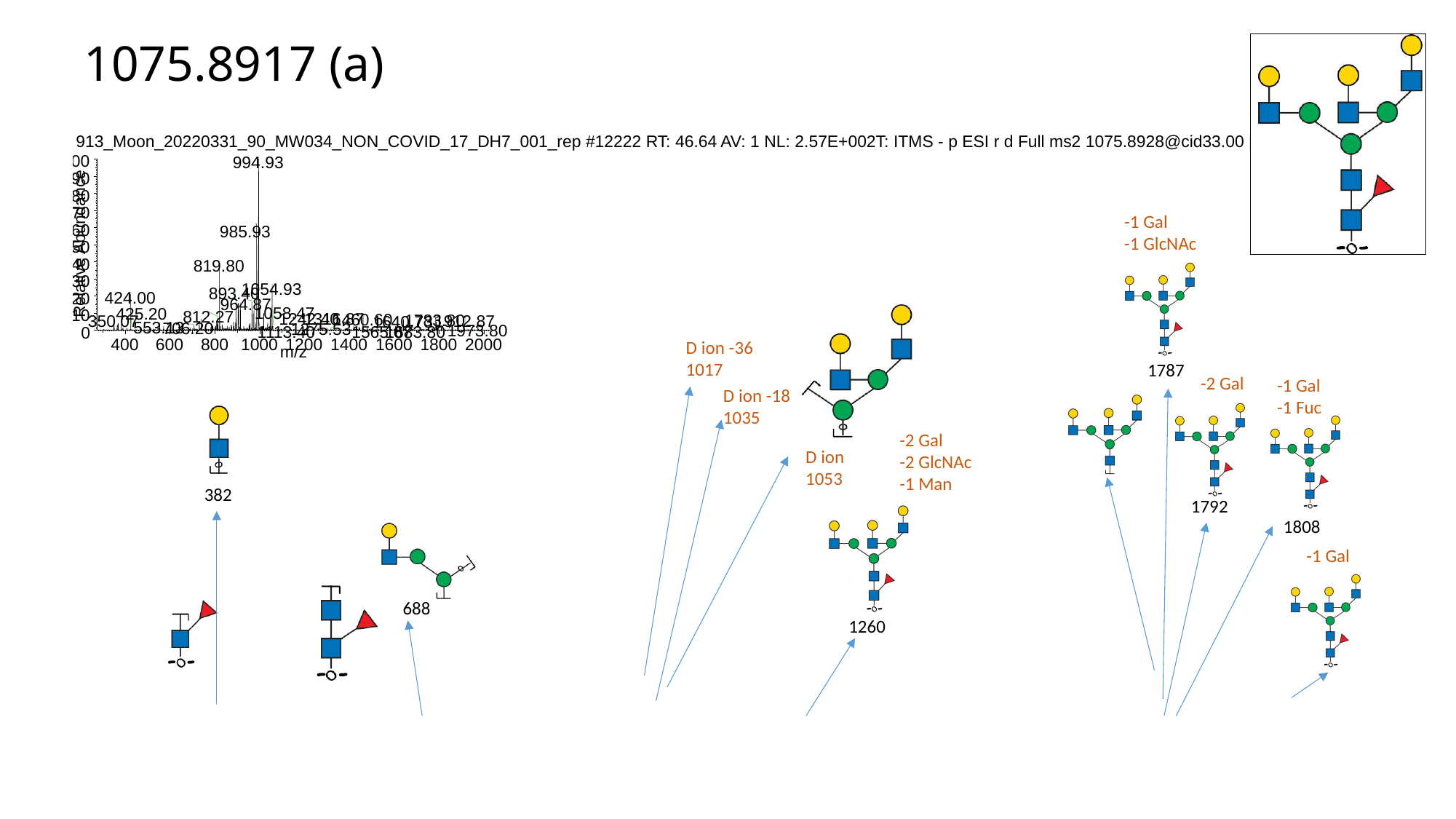

# 1075.8917 (a)
-1 Gal
-1 GlcNAc
D ion -36
1017
1787
-2 Gal
-1 Gal
-1 Fuc
D ion -18
1035
-2 Gal
-2 GlcNAc
-1 Man
D ion
1053
382
1792
1808
-1 Gal
688
1260

## Slide 145
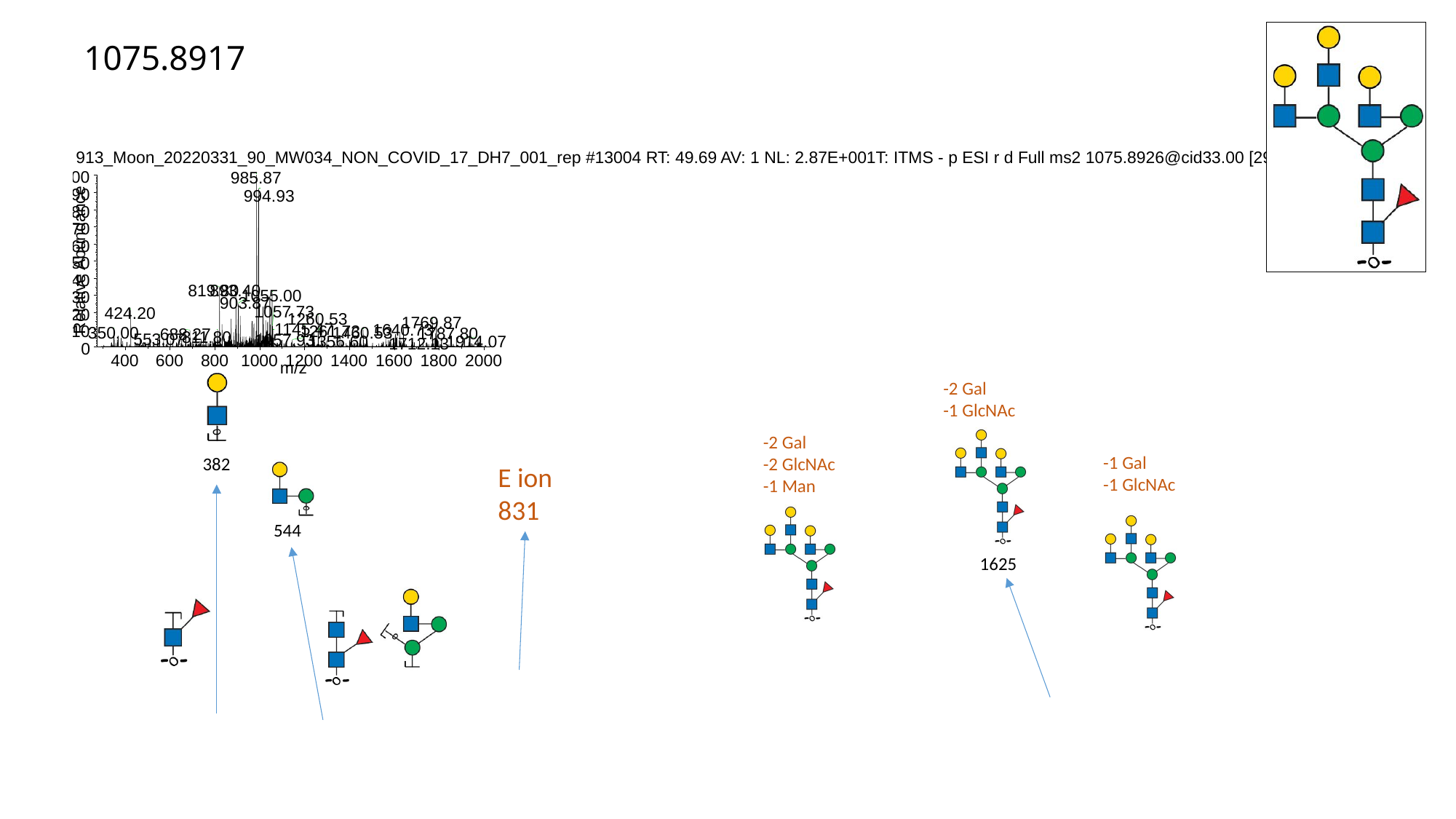

# 1075.8917
-2 Gal
-1 GlcNAc
-2 Gal
-2 GlcNAc
-1 Man
-1 Gal
-1 GlcNAc
382
E ion
831
544
1625

## Slide 146
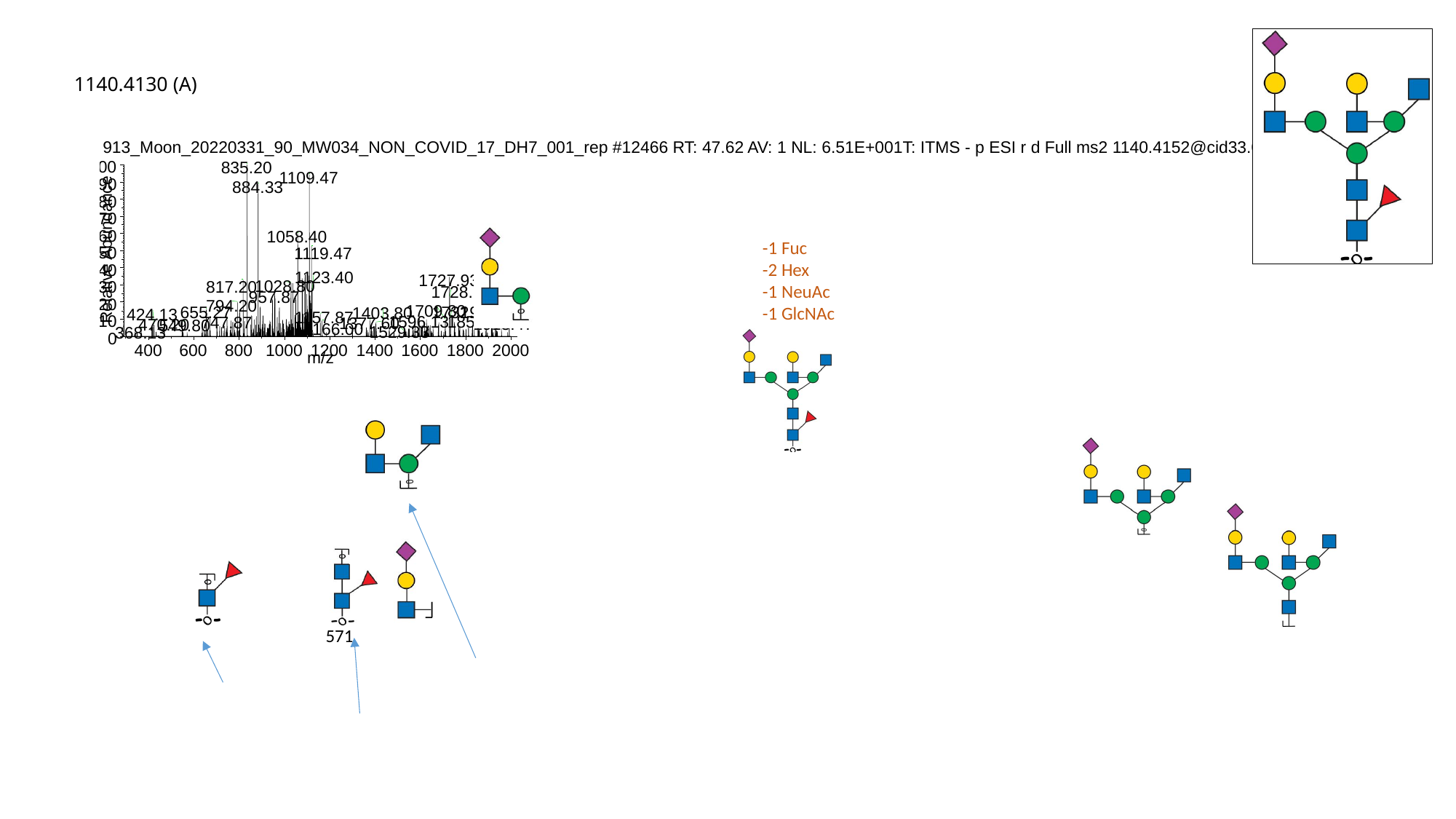

# 1140.4130 (A)
-1 Fuc
-2 Hex
-1 NeuAc
-1 GlcNAc
571

## Slide 147
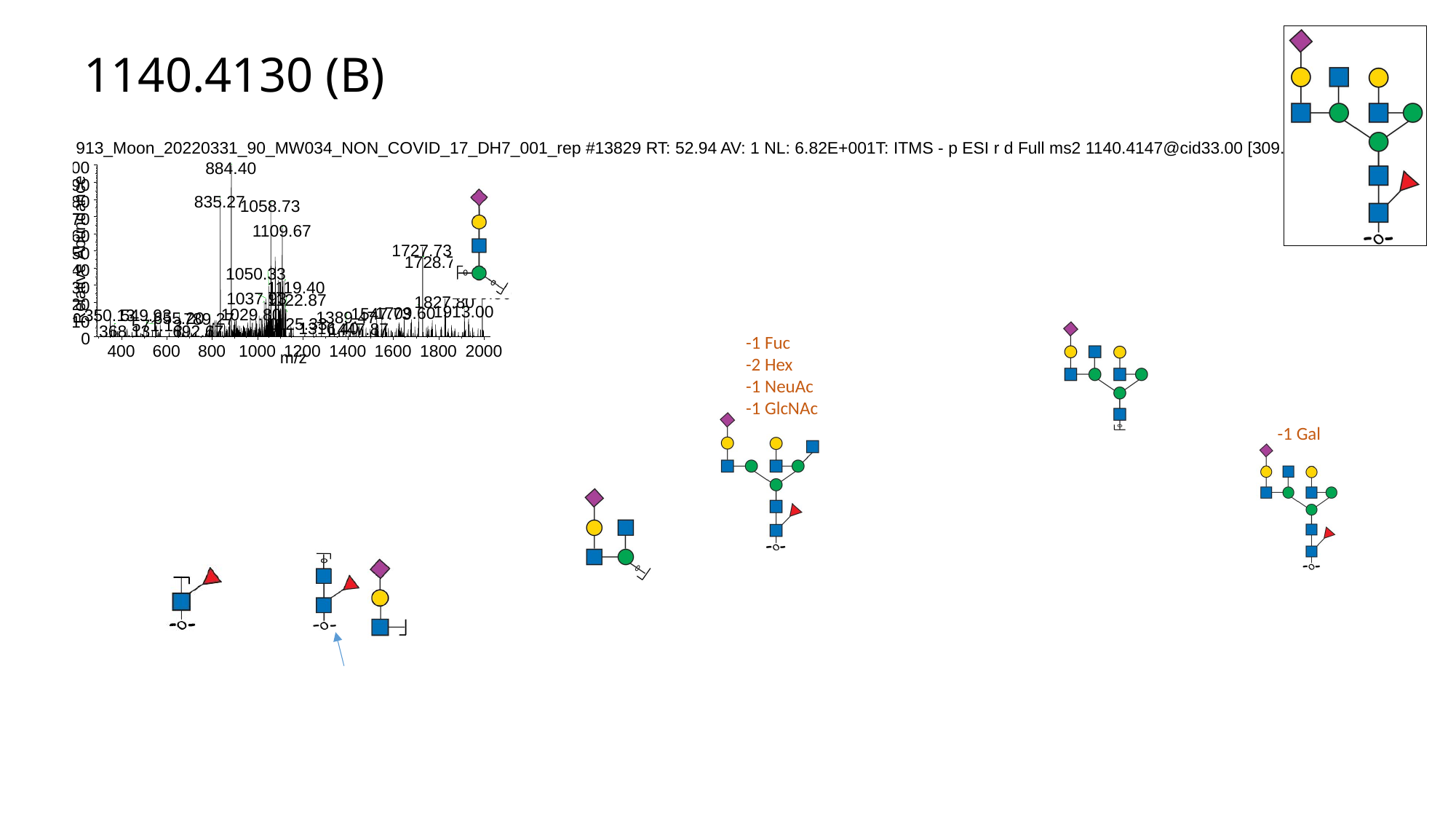

# 1140.4130 (B)
-1 Fuc
-2 Hex
-1 NeuAc
-1 GlcNAc
-1 Gal

## Slide 148
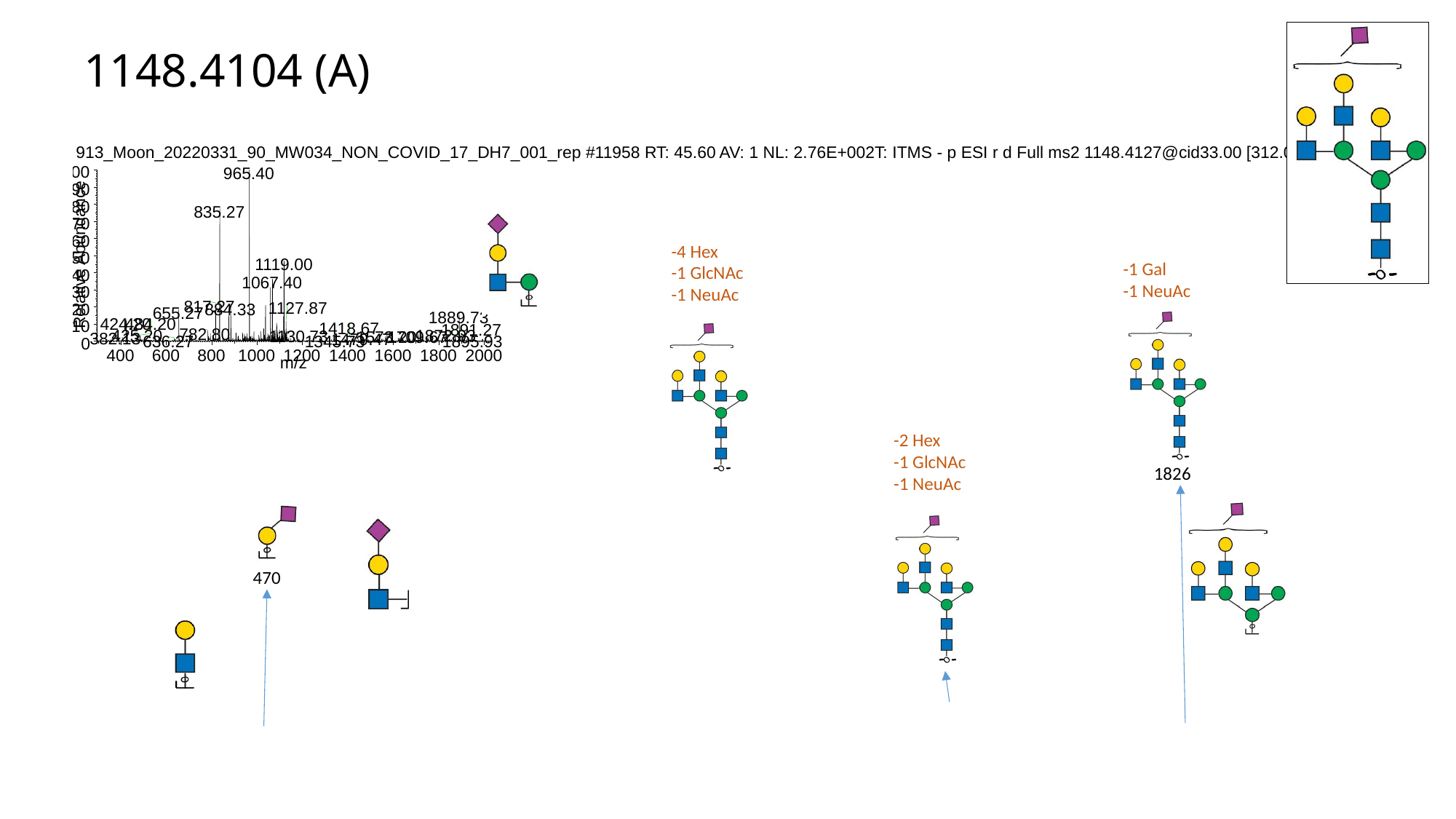

# 1148.4104 (A)
-4 Hex
-1 GlcNAc
-1 NeuAc
-1 Gal
-1 NeuAc
-2 Hex
-1 GlcNAc
-1 NeuAc
1826
470

## Slide 149
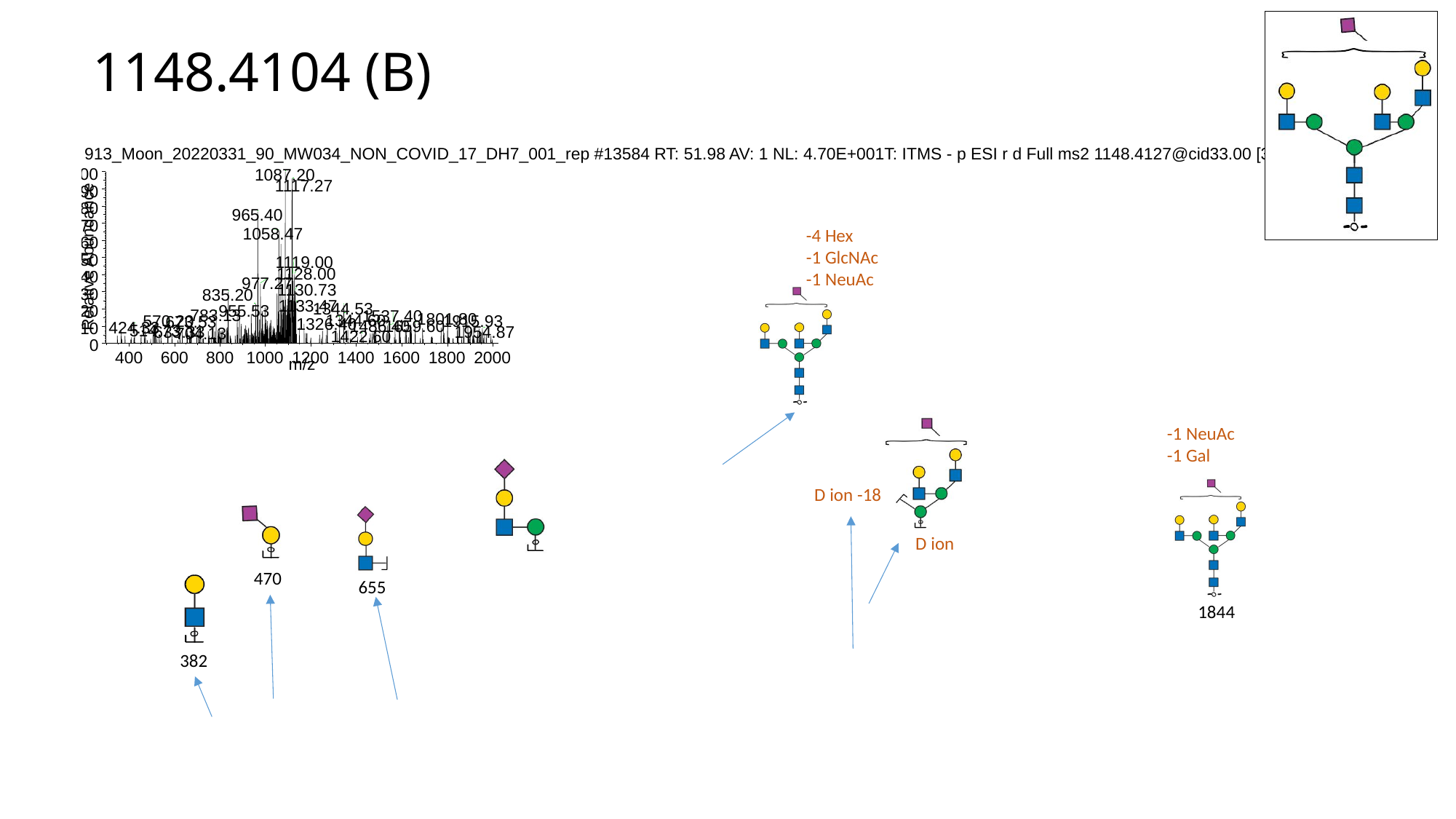

# 1148.4104 (B)
-4 Hex
-1 GlcNAc
-1 NeuAc
-1 NeuAc
-1 Gal
D ion -18
D ion
470
655
1844
382

## Slide 150
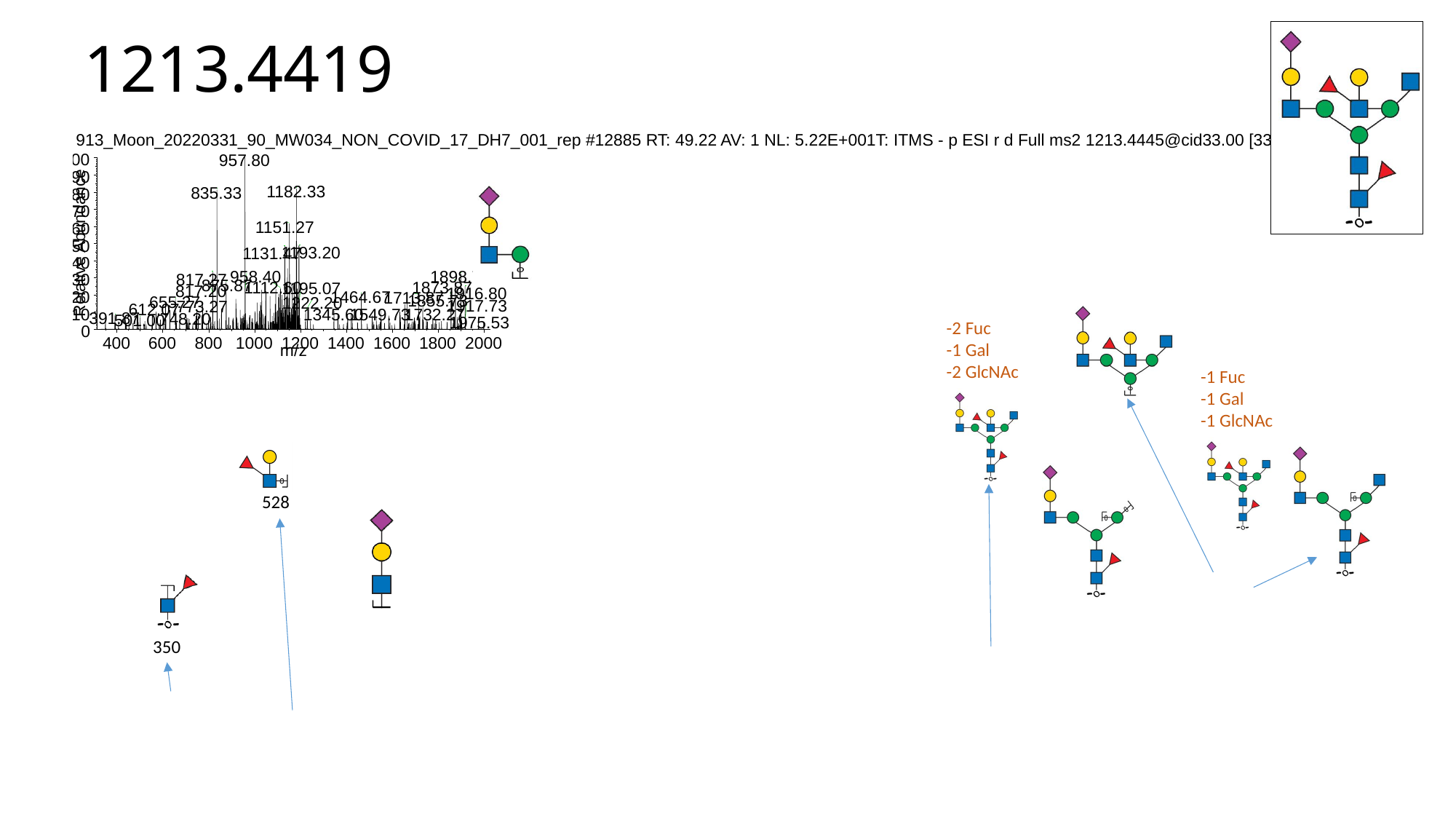

# 1213.4419
-2 Fuc
-1 Gal
-2 GlcNAc
-1 Fuc
-1 Gal
-1 GlcNAc
528
350

## Slide 151
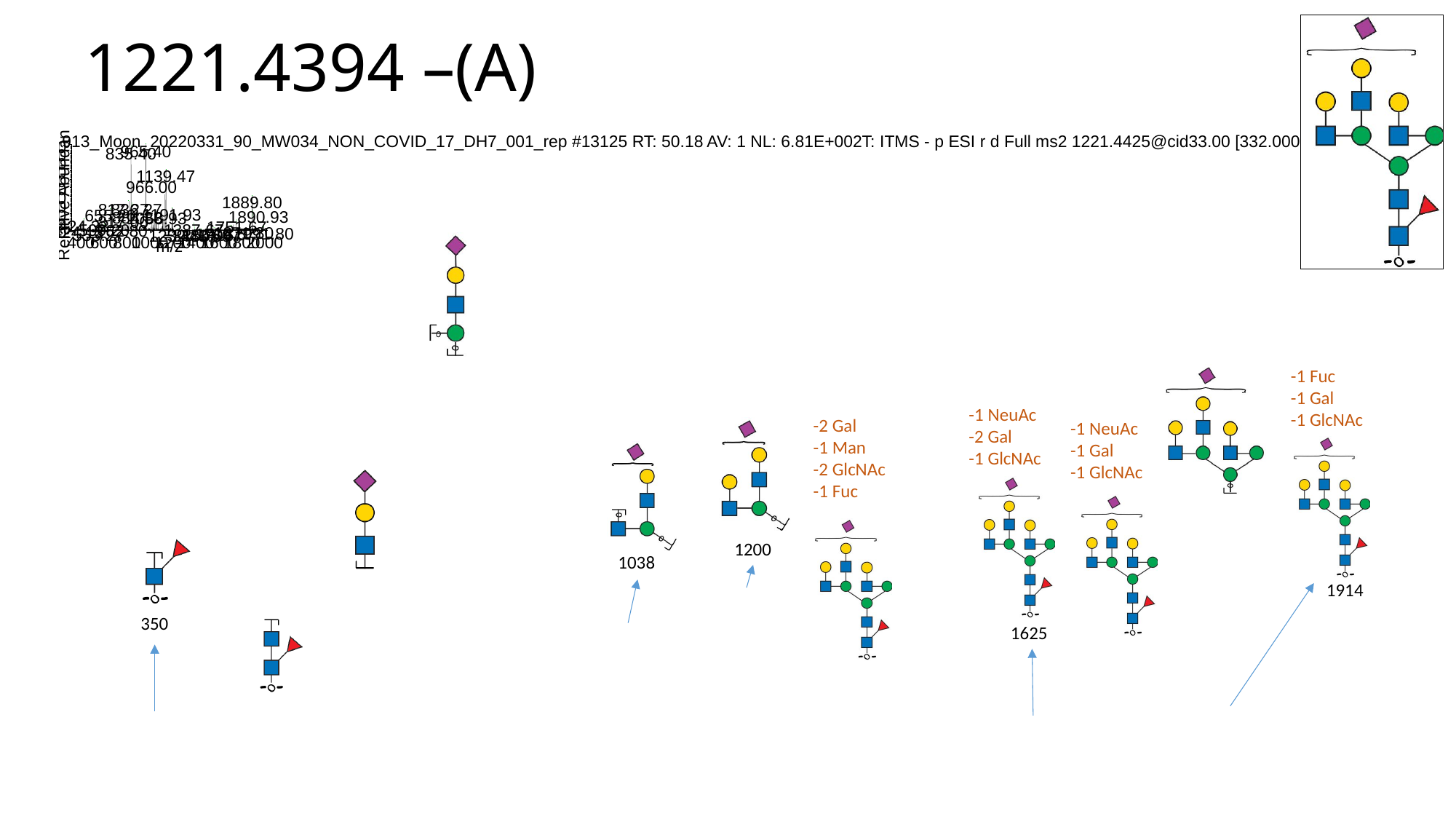

# 1221.4394 –(A)
-1 Fuc
-1 Gal
-1 GlcNAc
-1 NeuAc
-2 Gal
-1 GlcNAc
-2 Gal
-1 Man
-2 GlcNAc
-1 Fuc
-1 NeuAc
-1 Gal
-1 GlcNAc
1200
1038
1914
350
1625

## Slide 152
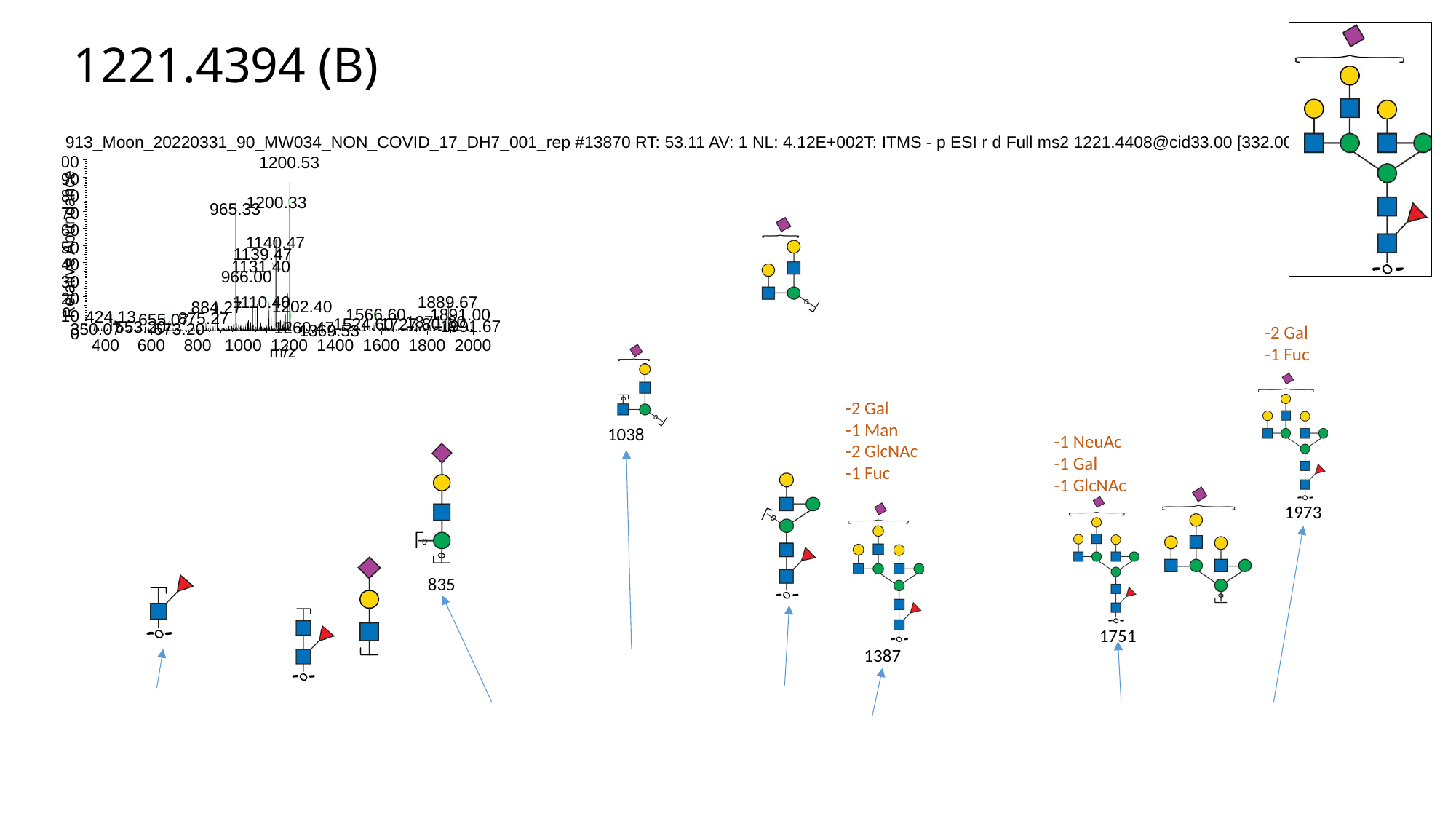

# 1221.4394 (B)
-2 Gal
-1 Fuc
-2 Gal
-1 Man
-2 GlcNAc
-1 Fuc
1038
-1 NeuAc
-1 Gal
-1 GlcNAc
1973
835
1751
1387

## Slide 153
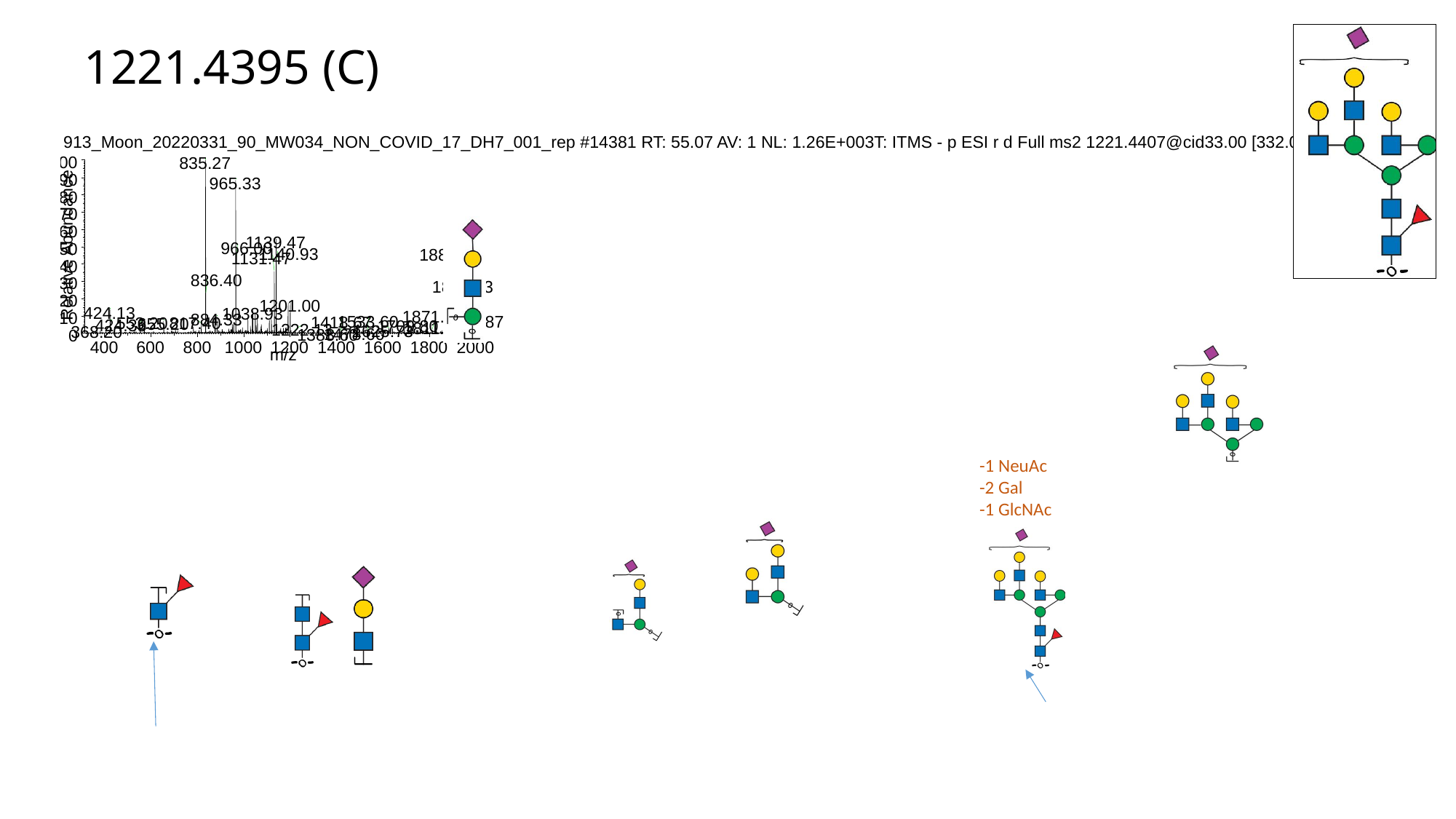

# 1221.4395 (C)
-1 NeuAc
-2 Gal
-1 GlcNAc

## Slide 154
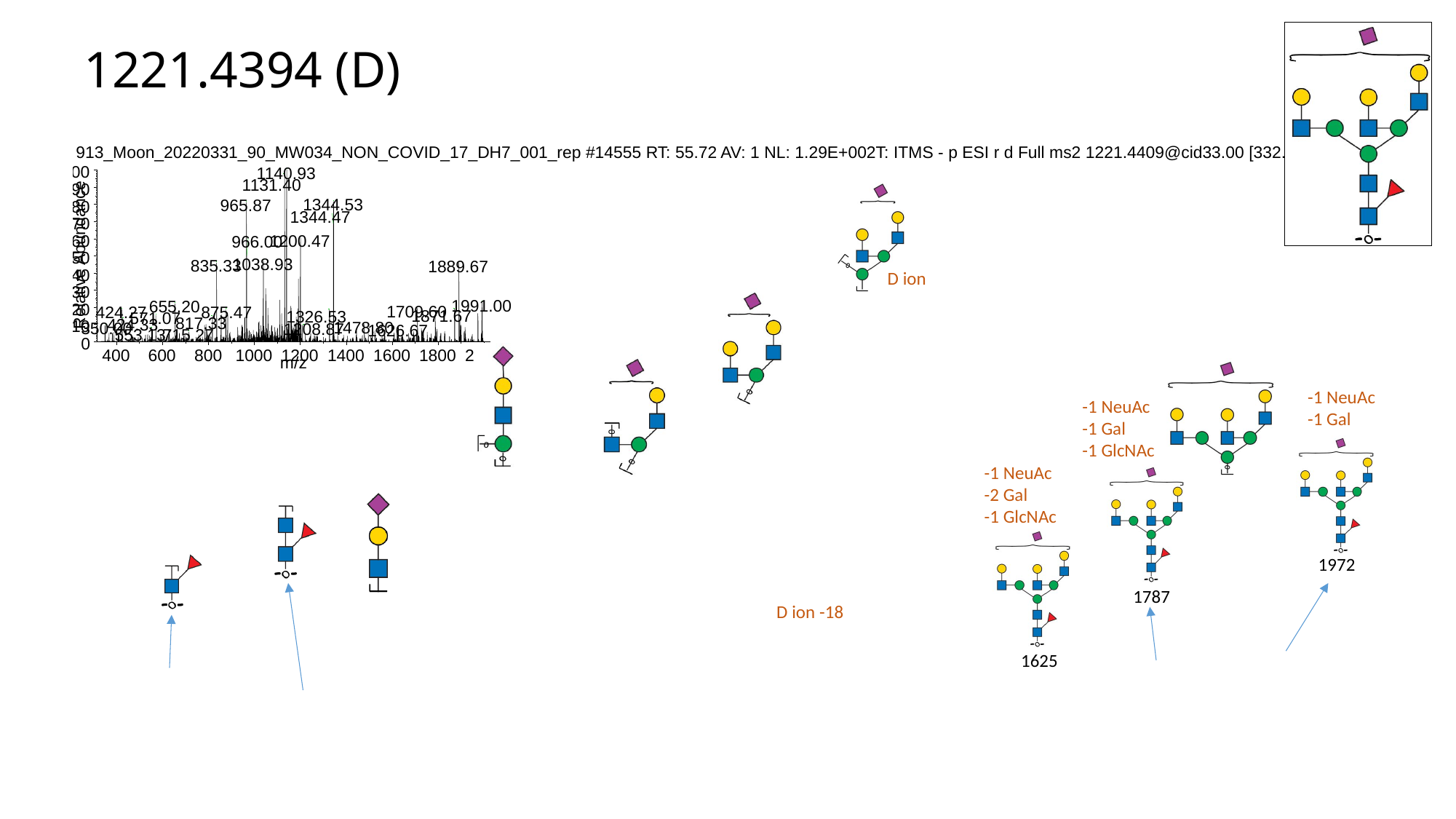

# 1221.4394 (D)
D ion
-1 NeuAc
-1 Gal
-1 NeuAc
-1 Gal
-1 GlcNAc
-1 NeuAc
-2 Gal
-1 GlcNAc
1972
1787
D ion -18
1625

## Slide 155
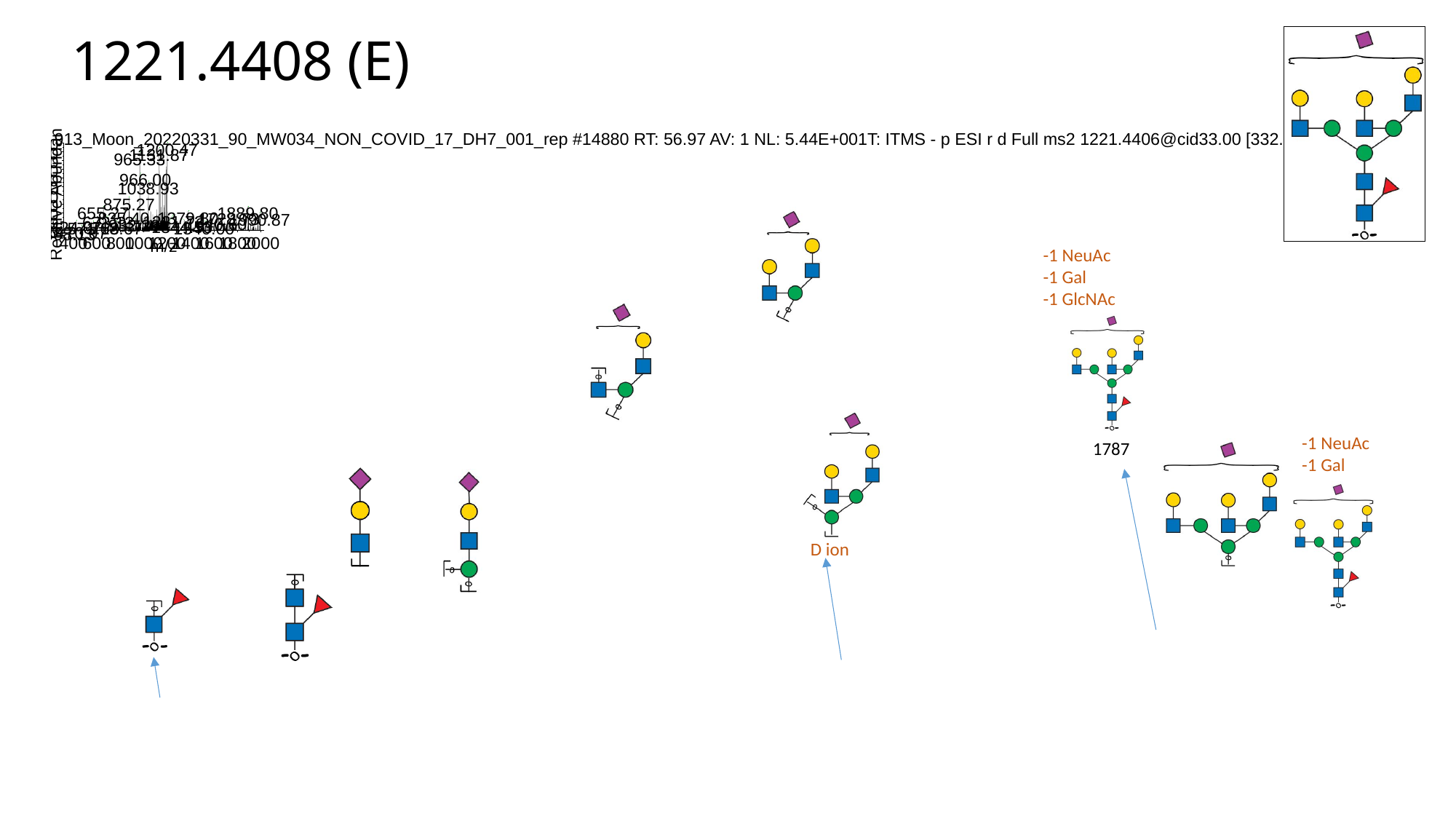

# 1221.4408 (E)
-1 NeuAc
-1 Gal
-1 GlcNAc
-1 NeuAc
-1 Gal
1787
D ion

## Slide 156
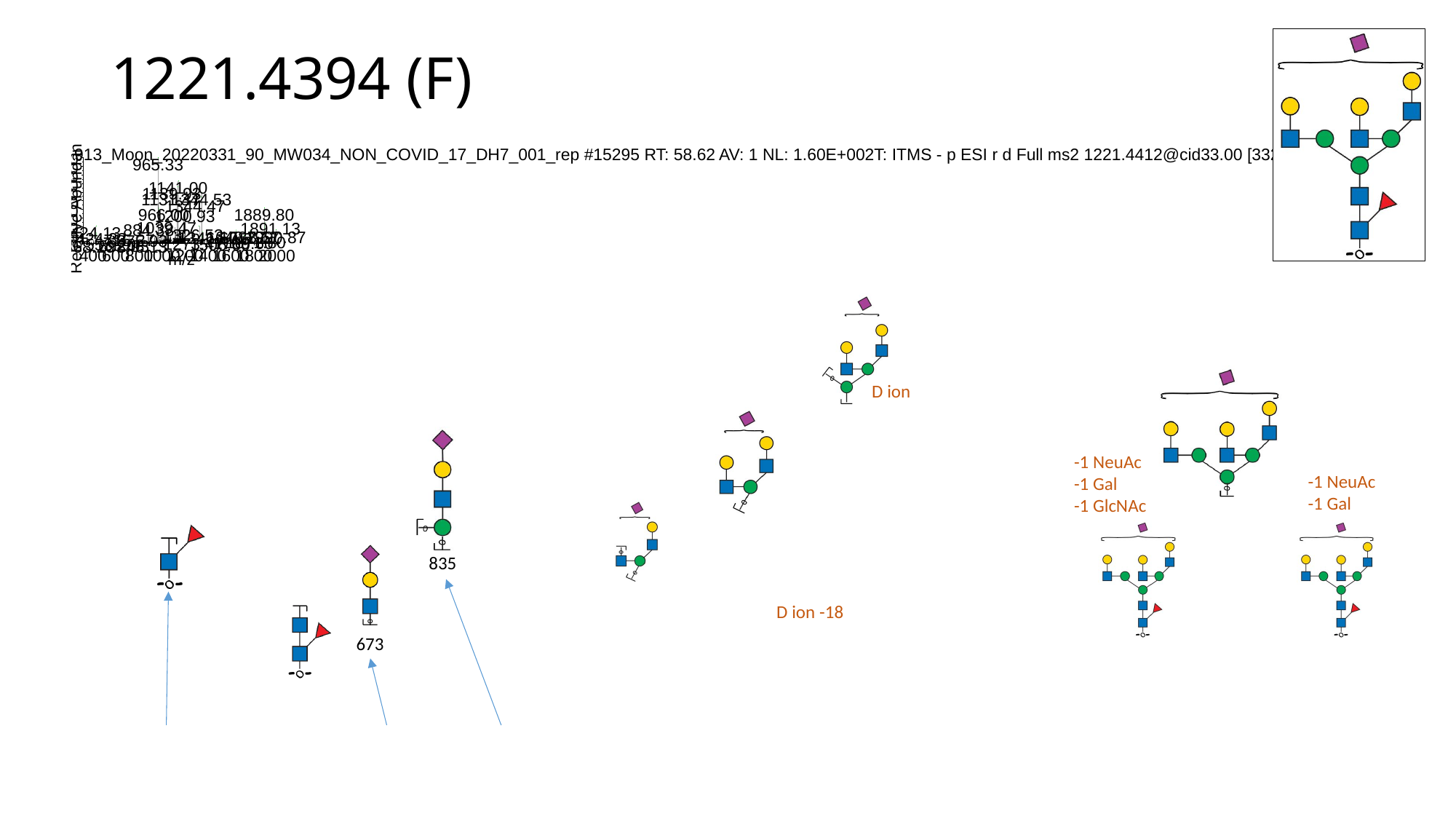

# 1221.4394 (F)
D ion
-1 NeuAc
-1 Gal
-1 GlcNAc
-1 NeuAc
-1 Gal
835
D ion -18
673

## Slide 157
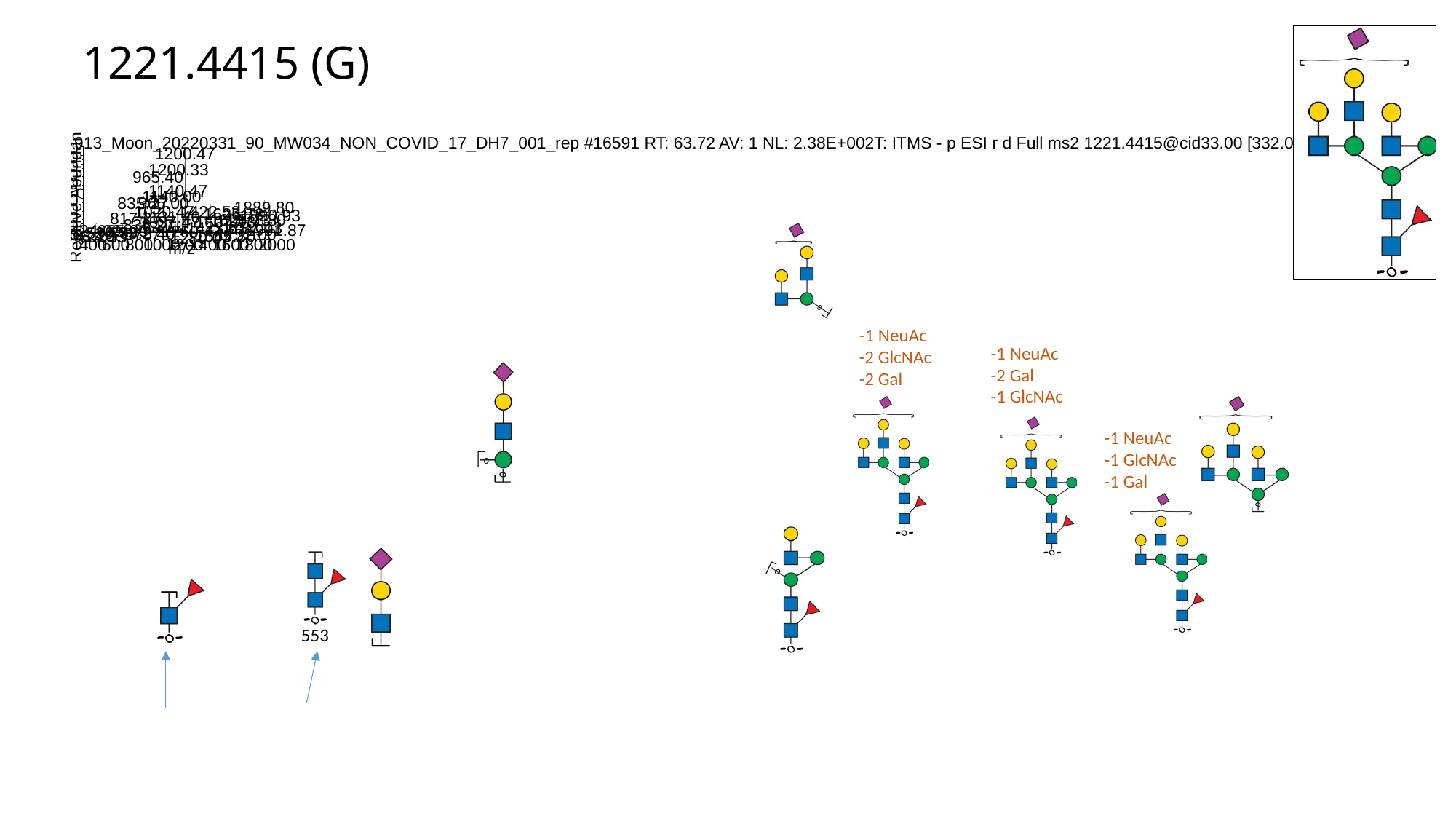

# 1221.4415 (G)
-1 NeuAc
-2 GlcNAc
-2 Gal
-1 NeuAc
-2 Gal
-1 GlcNAc
-1 NeuAc
-1 GlcNAc
-1 Gal
553

## Slide 158
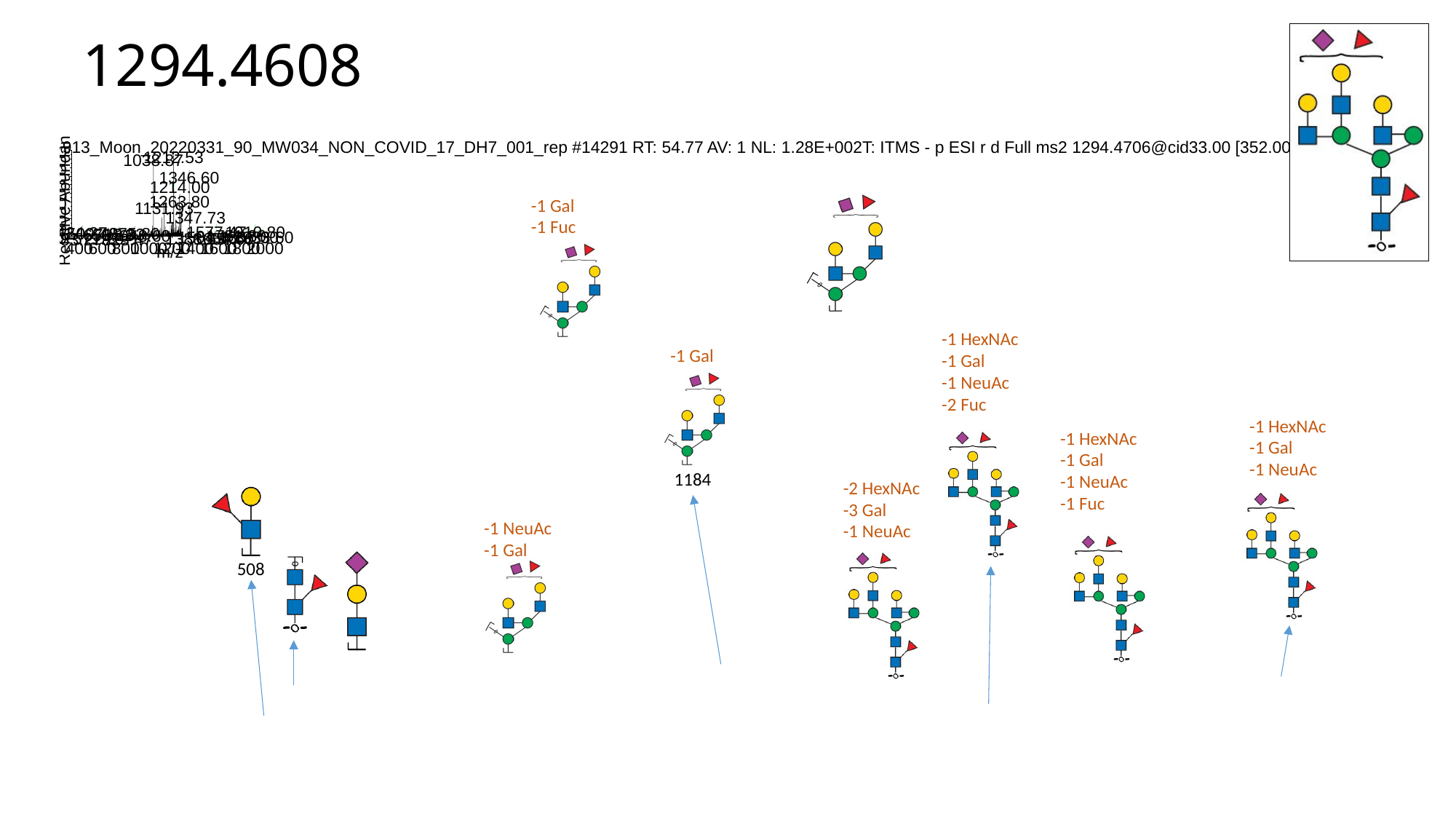

# 1294.4608
-1 Gal
-1 Fuc
-1 HexNAc
-1 Gal
-1 NeuAc
-2 Fuc
-1 Gal
-1 HexNAc
-1 Gal
-1 NeuAc
-1 HexNAc
-1 Gal
-1 NeuAc
-1 Fuc
1184
-2 HexNAc
-3 Gal
-1 NeuAc
-1 NeuAc
-1 Gal
508

## Slide 159
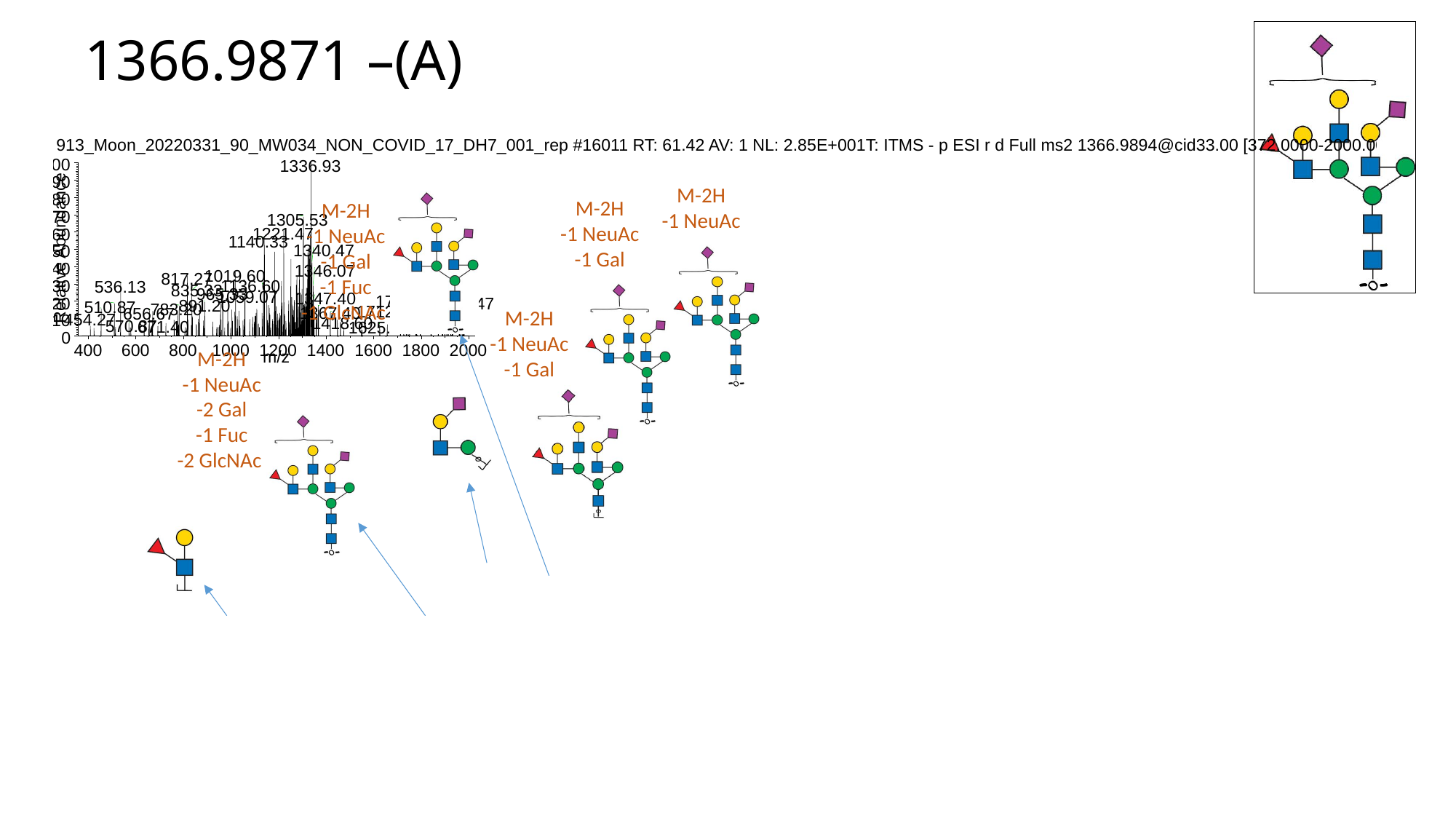

# 1366.9871 –(A)
M-2H
-1 NeuAc
M-2H
-1 NeuAc
-1 Gal
M-2H
-1 NeuAc
-1 Gal
-1 Fuc
-1 GlcNAc
M-2H
-1 NeuAc
-1 Gal
M-2H
-1 NeuAc
-2 Gal
-1 Fuc
-2 GlcNAc

## Slide 160
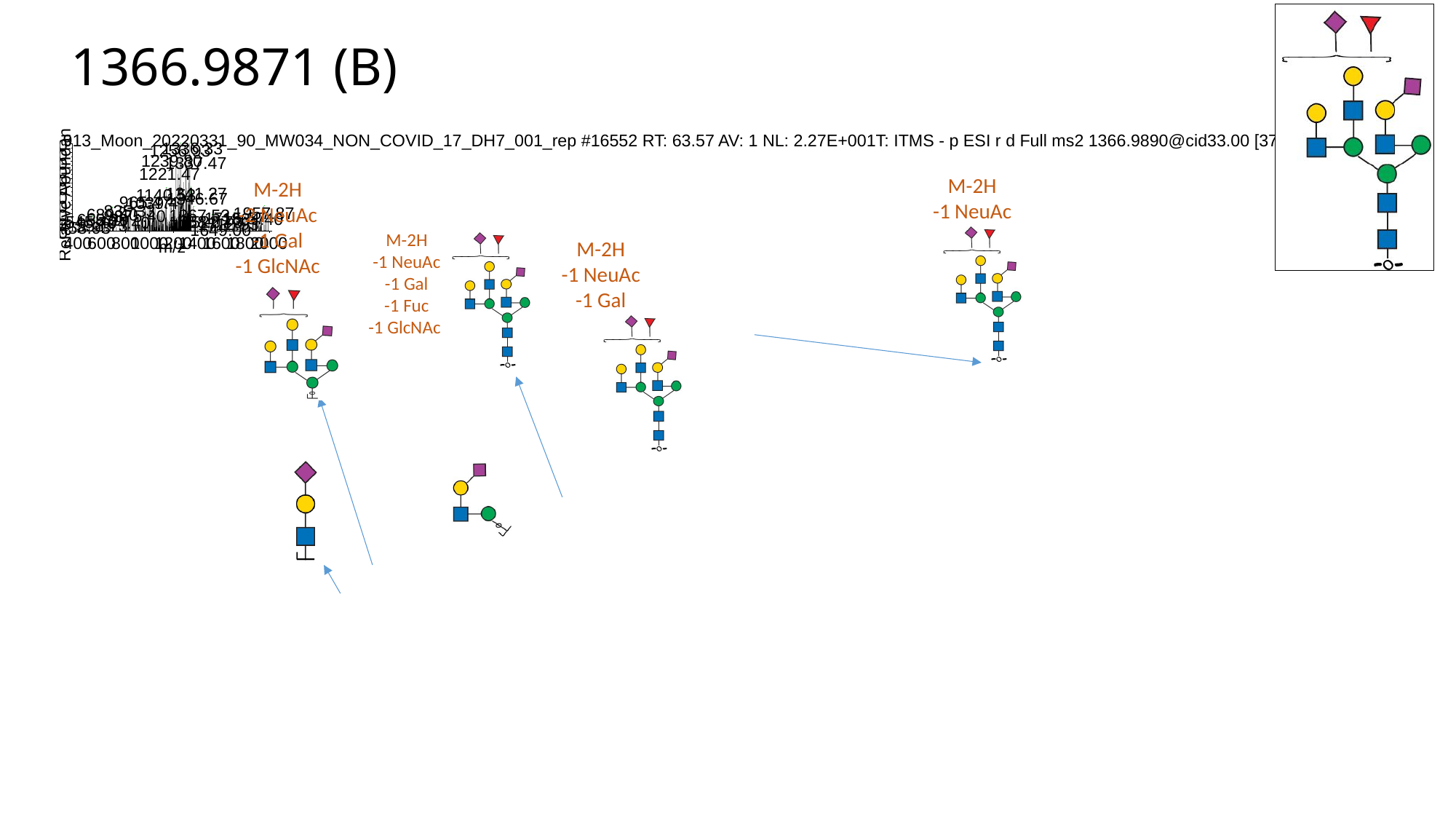

# 1366.9871 (B)
M-2H
-1 NeuAc
M-2H
-2 NeuAc
-1 Gal
-1 GlcNAc
M-2H
-1 NeuAc
-1 Gal
-1 Fuc
-1 GlcNAc
M-2H
-1 NeuAc
-1 Gal

## Slide 161
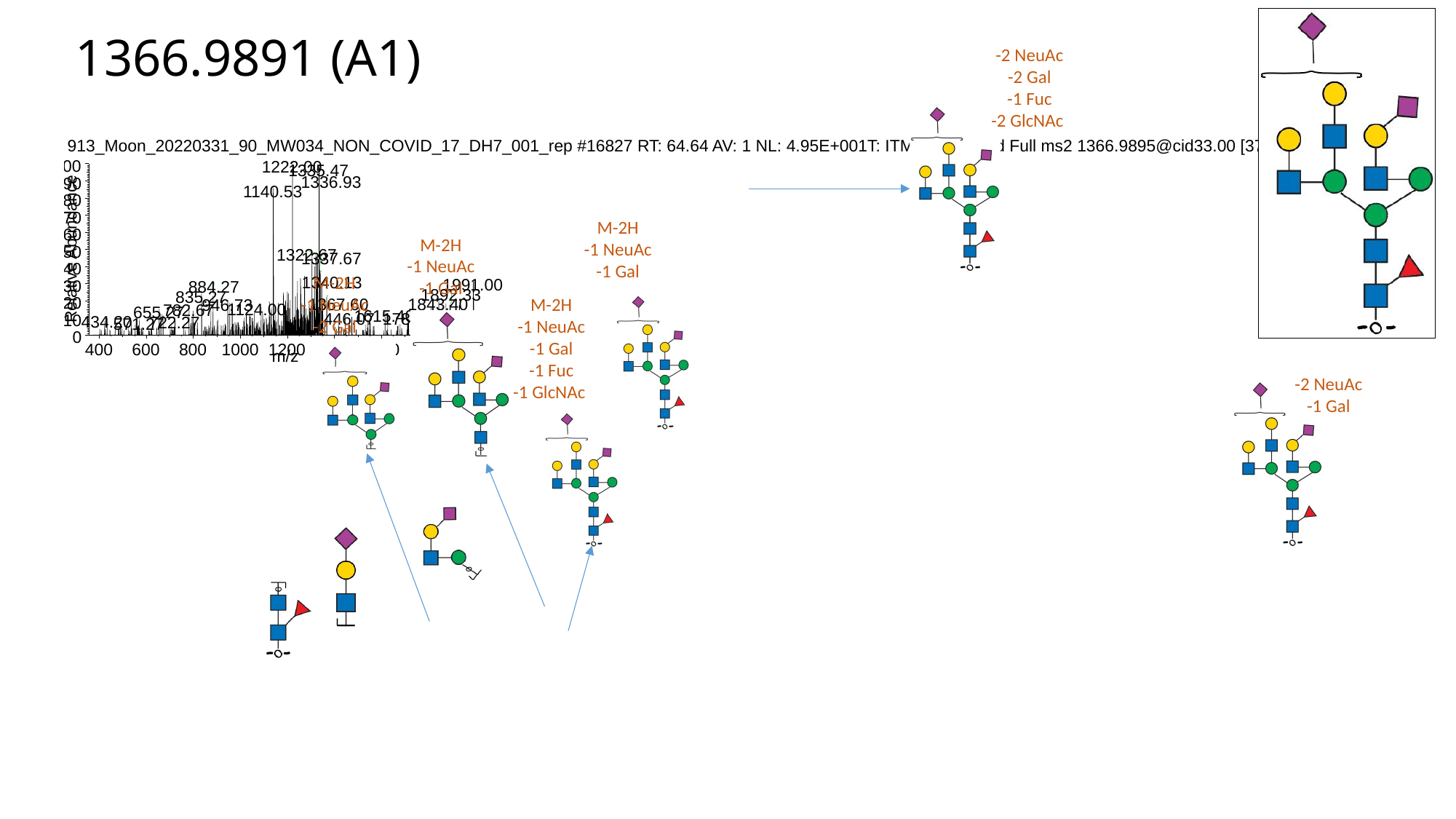

# 1366.9891 (A1)
-2 NeuAc
-2 Gal
-1 Fuc
-2 GlcNAc
M-2H
-1 NeuAc
-1 Gal
M-2H
-1 NeuAc
-1 Gal
M-2H
-1 NeuAc
-2 Gal
M-2H
-1 NeuAc
-1 Gal
-1 Fuc
-1 GlcNAc
-2 NeuAc
-1 Gal

## Slide 162
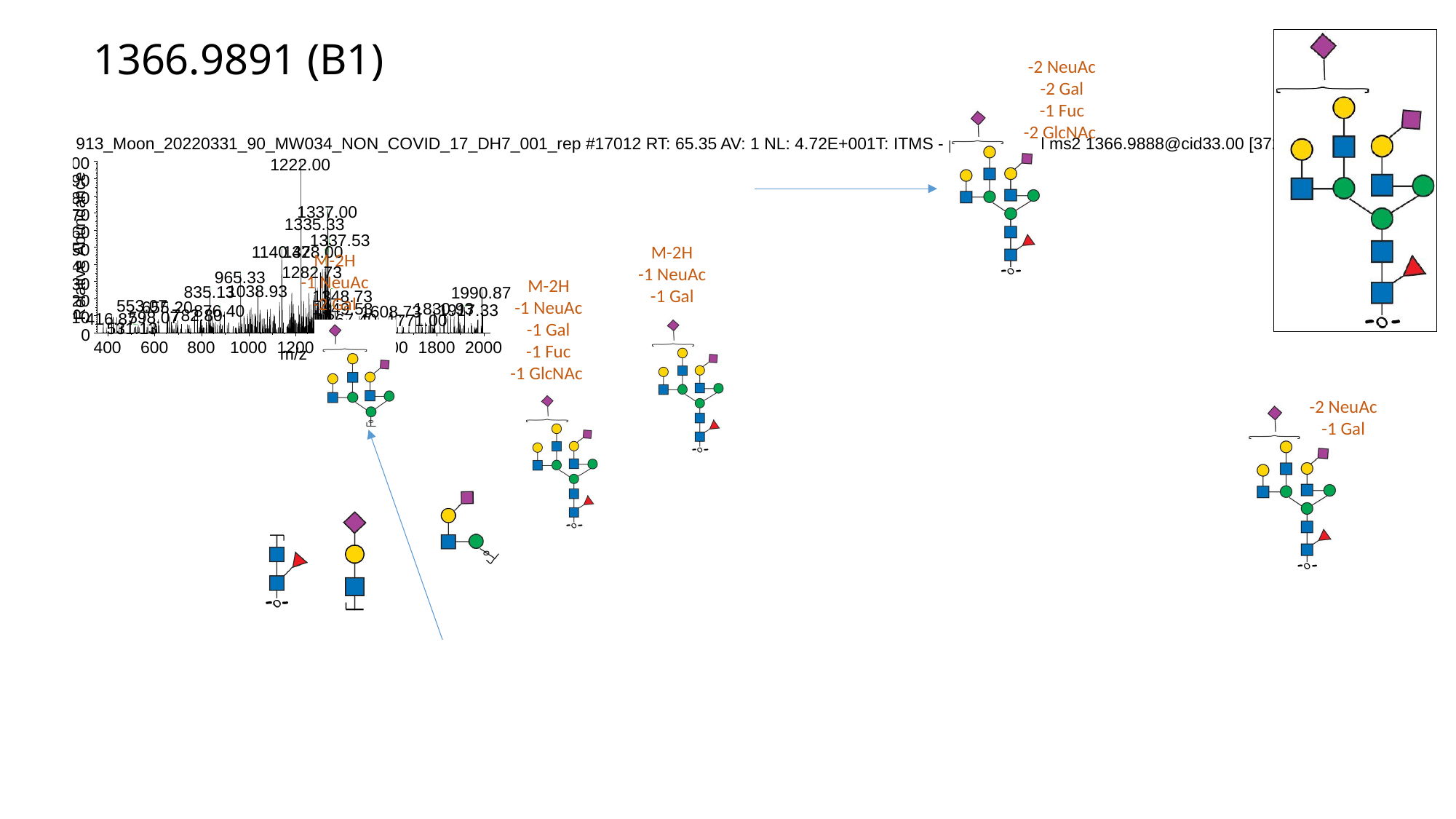

# 1366.9891 (B1)
-2 NeuAc
-2 Gal
-1 Fuc
-2 GlcNAc
M-2H
-1 NeuAc
-1 Gal
M-2H
-1 NeuAc
-2 Gal
M-2H
-1 NeuAc
-1 Gal
-1 Fuc
-1 GlcNAc
-2 NeuAc
-1 Gal

## Slide 163
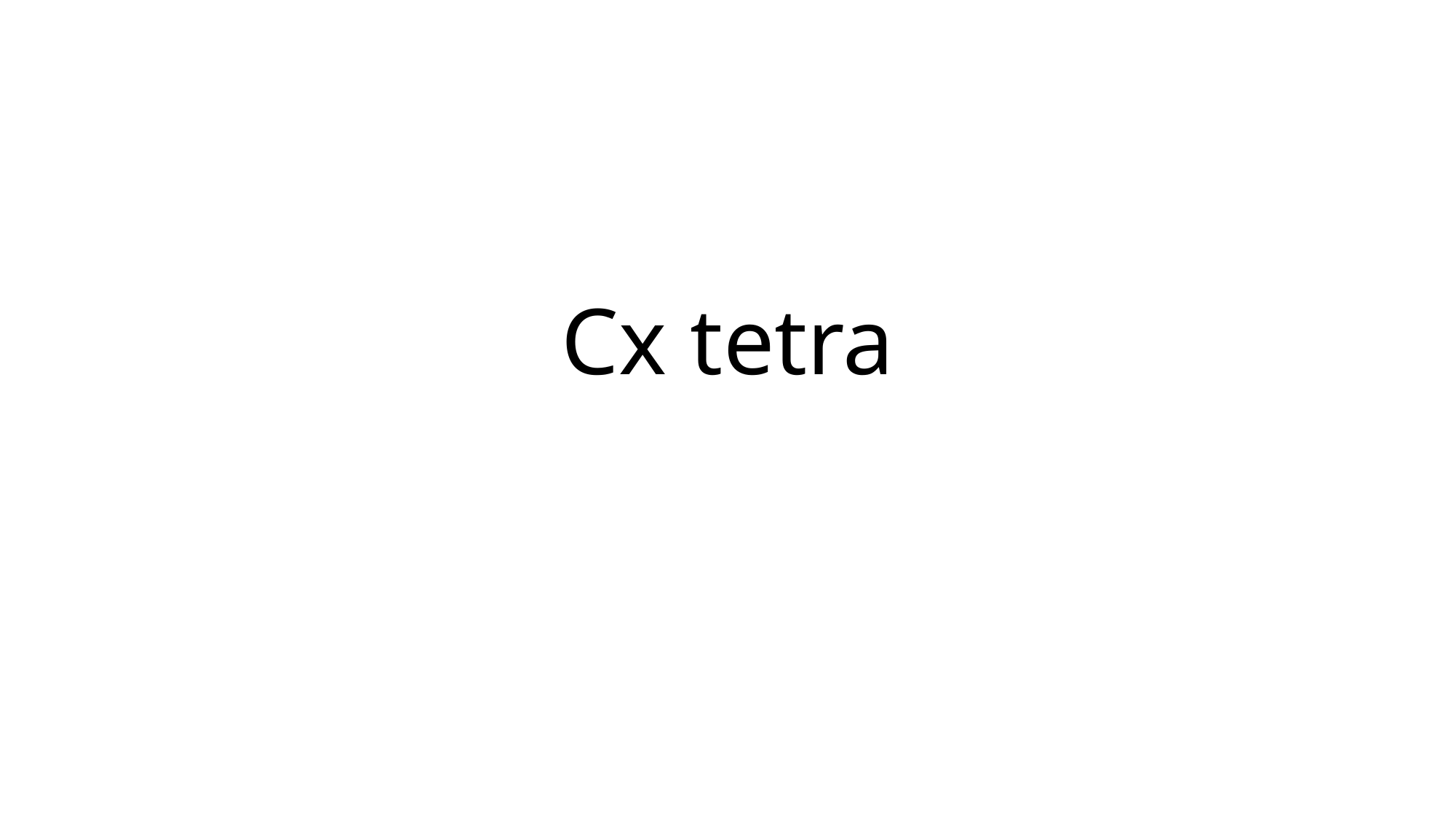

# Cx tetra

## Slide 164
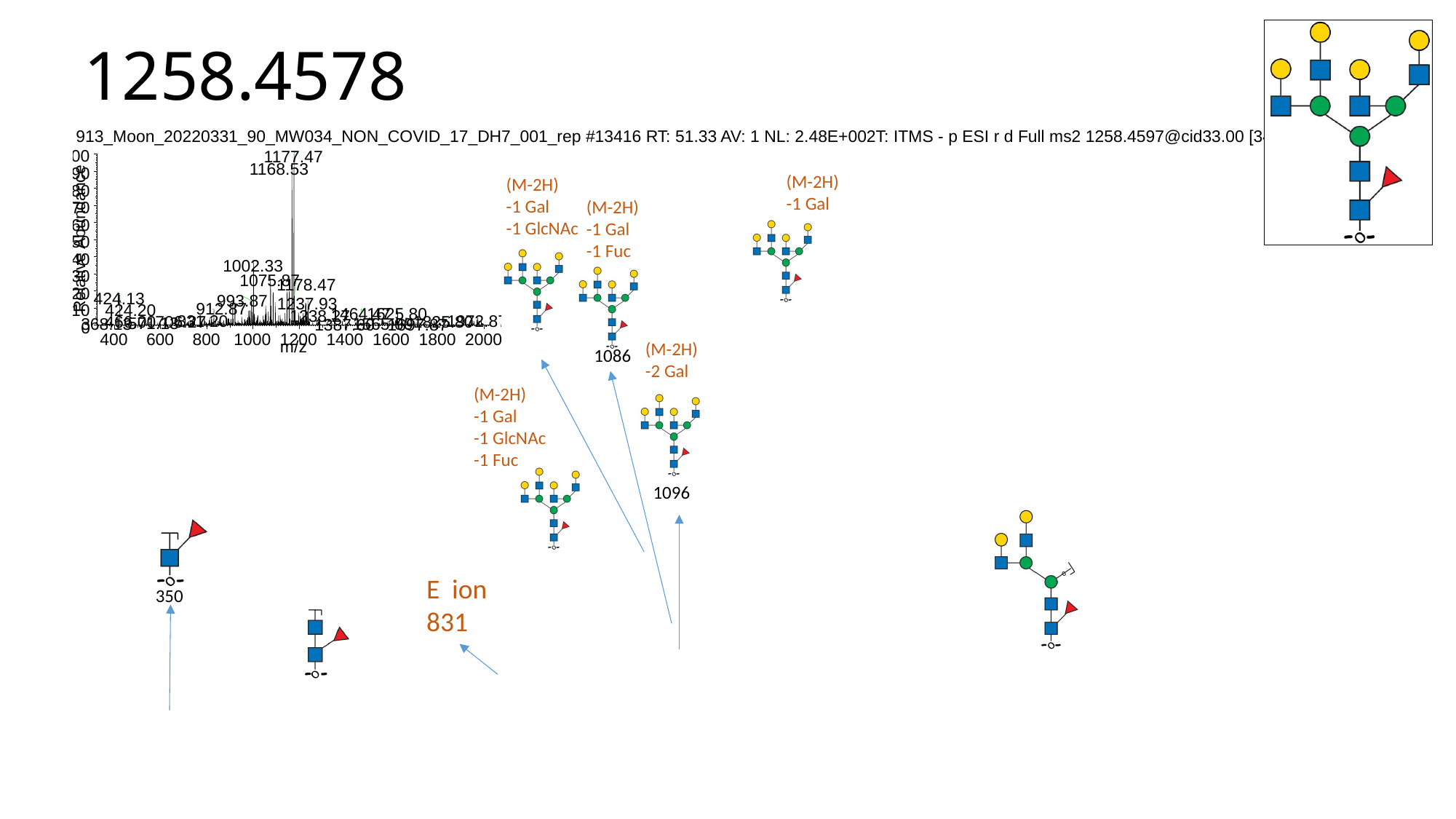

# 1258.4578
(M-2H)
-1 Gal
(M-2H)
-1 Gal
-1 GlcNAc
(M-2H)
-1 Gal
-1 Fuc
(M-2H)
-2 Gal
1086
(M-2H)
-1 Gal
-1 GlcNAc
-1 Fuc
1096
E ion
831
350

## Slide 165
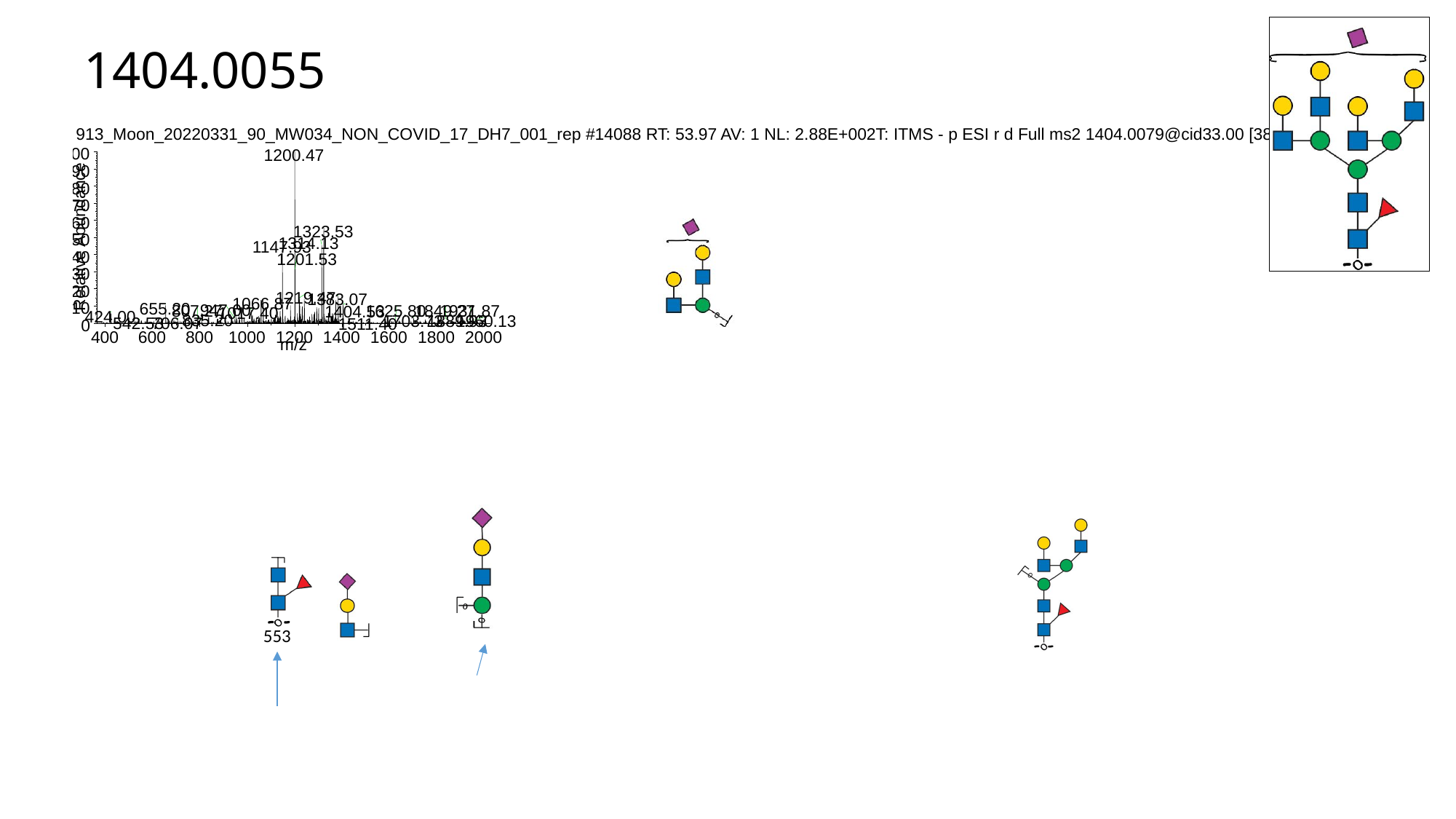

# 1404.0055
553

## Slide 166
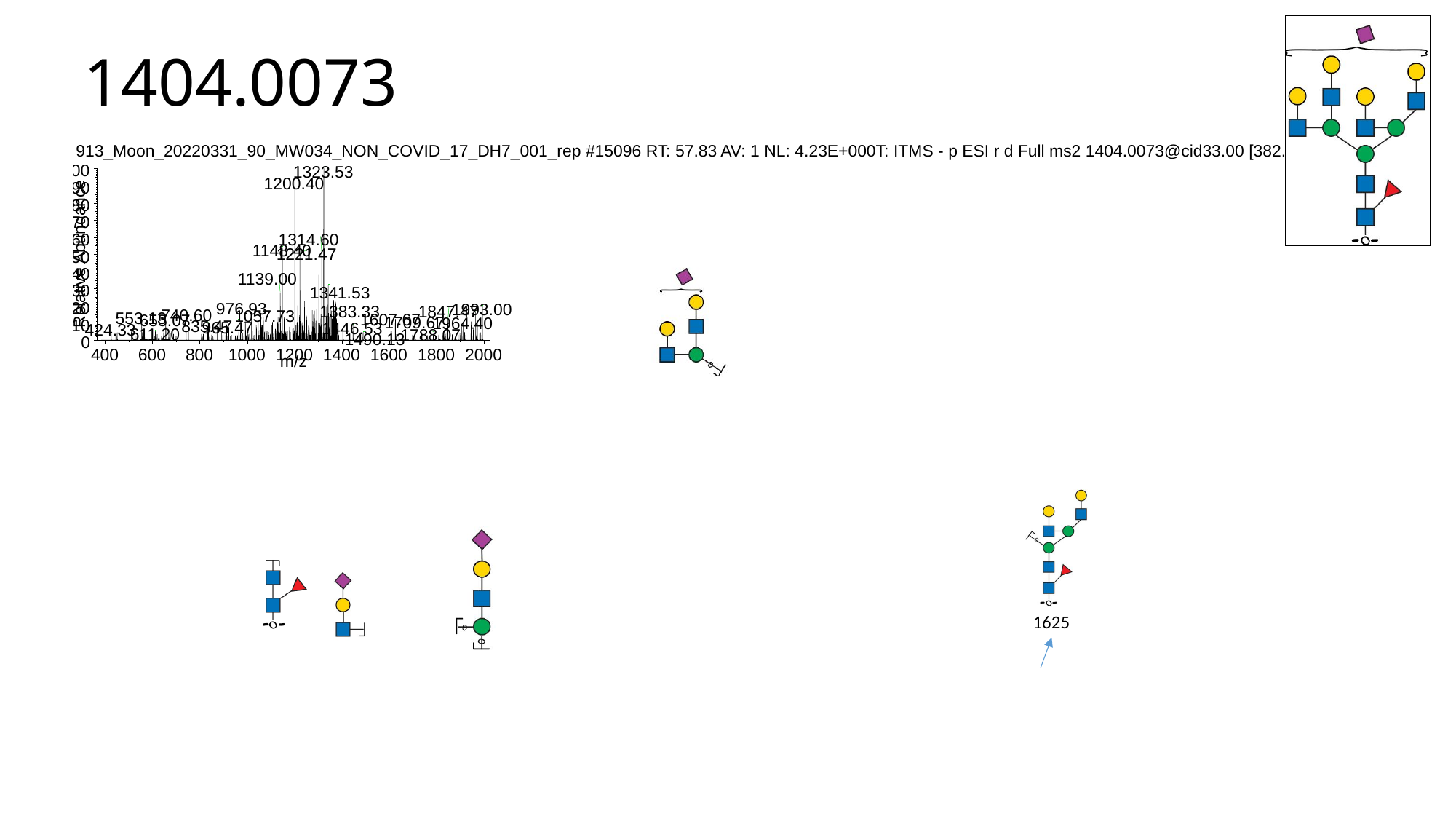

# 1404.0073
1625
