# Supplementary material for: Integrated Multiomics Reveals Alterations in Paucimannose and Complex Type N-Glycans in Cardiac Tissue of Patients with COVID-19
Source: Mol Cell Proteomics. 2025 Feb 22;24(4):100929. doi: 10.1016/j.mcpro.2025.100929 (PMC12131855; doi:10.1016/j.mcpro.2025.100929)
Supplement: Supplemental_Methods [file mmc2.docx]

**Integrated multiomics reveals alterations in paucimannose and complex type *N*-glycans in cardiac tissue of COVID-19 patients**

Sabarinath Peruvemba Subramanian^1#^, Melinda Wojtkiewicz^1^, Fang Yu^2^, Chase Castro^1^, Erin N. Schuette^1^, Jocelyn Rodriguez-Paar^1^, Jared Churko^3^, Pranav Renavikar^4^, Daniel Anderson^5^, Claudius Mahr^6^, and Rebekah L. Gundry^1#^

^#^Corresponding authors: Dr. Sabarinath Subramanian and Dr. Rebekah L. Gundry

^1^CardiOmics Program, Center for Heart and Vascular Research, and Department of Cellular and Integrative Physiology, University of Nebraska Medical Center, Omaha, NE, 68198, USA.

^2^Department of Biostatistics, University of Nebraska Medical Center, Omaha, NE, 68198, USA.

^3^Department of Cellular and Molecular Medicine, The University of Arizona, Tucson, AZ, USA

^4^Department of Pathology, Microbiology, and Immunology, University of Nebraska Medical Center, Omaha, NE, 68198, USA.

^5^Department of Internal Medicine, Division of Cardiovascular Medicine, University of Nebraska Medical Center, Omaha, NE, 68198, USA.

^6^Institute for Advanced Cardiac Care, Medical City Healthcare, Dallas, TX, 75243, USA

***Methods***

***Deparaffinized and hydration of FFPE sections***

Unstained FFPE tissue sections were deparaffinized and hydrated by immersing for 3 min sequentially in 100% xylene (twice), 50% v/v xylene: ethanol, 100% ethanol (twice), 95% v/v ethanol: deionized water, 75% v/v ethanol: deionized water, 50% v/v ethanol: deionized water and 100% water respectively. After rehydration, the slides were incubated for 20 min in boiling potassium citrate buffer (10 mM potassium citrate monohydrate tribasic, 0.05% Tween 20, pH adjusted to 6.0 with phosphoric acid) for antigen retrieval. The whole boiling unit was cooled in a water bath (with circulating water) until the temperature was brought down to room temperature. Following antigen retrieval, the slides were washed by immersion in deionized water for 3 min, water droplets were removed from the edges using Kimwipes, and a boundary was marked around the tissue section using a Pap pen (Vector Labs) to avoid lysis buffer from flowing out while scraping.

***High resolution mass spectrometry-based glycomics***

Reduced *N-*glycans were reconstituted in 40 µl MS grade water containing retention time standards and analyzed on porous graphitized column (PGC)-LC-MS using UltiMate3000 high-performance liquid chromatography (HPLC) system (Dionex, Sunnyvale, CA, USA) coupled to Orbitrap Eclipse Tribrid Mass spectrometer (Thermo Fisher Scientific, San Jose, CA, USA). PGC column was packed in-house into fritted 20 cm fused silica capillary (200 nm i.d., 363 µm o.d. from Polymicro Technologies, Phoenix, AZ). Briefly, fritted fused silica capillaries were prepared by inserting one end of the capillary with 200 µl 29% potassium silicate, 50 µl formamide, and glass microfiber filter (GE Healthcare) mixture and allowed to polymerize at 90°C overnight. PGC Hypercarb (Thermo Scientific) slurry (20 mg/mL in methanol and 3 µm particle size) was packed into fritted fused capillary up to 10.5 cm mark using a Pressure Injection Cell and a constant nitrogen pressure of 1200 psi. The packed column was washed extensively in methanol before use and compacted to 10 cm. LC method parameters include 10 mM ammonium bicarbonate aqueous solution (pH adjusted to 7.0; Solvent A) and 10 mM ammonium bicarbonate aqueous solution with 60% (v/v) acetonitrile (Solvent B) as mobile phase, 5 µl sample injection volume, 6 μl/min flow rate, 65°C oven temperature, and 10 µl post column make flow with 100% acetonitrile. The sample was directly injected into the column and eluted over 90 min gradient followed by a wash cycle and equilibration. LC gradient was 7% B (0-1 min), linear increase to 21% B in 42 min, 35% B (60 min), 80% B (69 min), and 99% B (70 min). The valves were switched to wash cycle with 99% methanol from loading pump at 70-78 min. The gradient was dropped to 1% B and held until 90 min. MS and MS/MS data was obtained in negative ion mode using Orbitrap Eclipse Tribrid Mass spectrometer coupled with M3 MnESI source (Newomics, USA) and 5 nozzle emitter of 20 µm ID. MS settings: Spray Voltage -3.0 kV, Sheath gas flow rate 20 (AU), Ion transfer tube temperature 275°C, MS1 scan range of m/z 500–2000, RF lens % 40; MS1 resolution 120000, MS1 normalized AGC target 200%, MS1 maximum IT 200 ms. MS2 scan rate: rapid, MS2 isolation window: 2 m/z, MS2 normalized AGC target 200%, MS2 maximum IT 200 ms, CID 33%, activation time 10 ms, mass tolerance of ±10 ppm.

***Structure annotation of glycan isomers***

The following criteria was followed for structure and isomer assignment: a) General order of elution of glycan class in PGC-LC-MS was paucimannose, oligo-mannose, bisecting, complex, hybrid, followed by complex bi-, tri-, and tetra-antennary structures; b) Linkage within isomers was assigned based on the elution pattern, glycans with 6-linked Man or Sia earlier than 3-linked counterparts. Likewise, LacdiNAc structures elute earlier than the isomeric complex tri- and terta antennary structures; c) Additionally, the presence of D and D-18-ions was used as diagnostic ions to assign glycans linked to the 6-arm and its absence confirmed the linkage on the 3-arm. Structural annotation with diagnostic ions is summarized in the Supplementary Table.1 and Supplementary data file 1. For Man_2_GlcNAc_2_, two isomers were observed at RRT of 5.5 (14.34 min) and 7.2 (18.41 min), 6-linked isomers showed presence of 0,2A_Man_ (m/z 281.07) and 0,4A_Man_ (m/z 221.07) respectively. These ions were absent in 3-linked structures. For Man_3_GlcNAc_2_ and Fuc_1_Man_3_GlcNAc_2,_ three isomers were observed at RRT 4.6, 5.7, 6.7 and Fuc_1_Man_3_GlcNAc_2_ at RRT 6.9, 8 and 9 respectively. Early eluting structures had di-man (m/z 341), tri-man (m/z 503 and 485) and 0,2 A_Man_ but did not contain D and D-18 ion (m/z 323 and 305). Therefore, early eluting Man3/Man3Fuc1 glycans was assigned as 1-6 mannose arm with 6- and 3-linked mannose. The latter eluting Man3/Man3Fuc1 containing D and D-18 ions suggesting presence of 1-3 and 1-6 arm mannose linked to 1-4 mannose linked to GlcNAc. These results corroborate with structures reported by (Liew et al., 2024). For LacdiNAc containing structures it has been observed that LacdiNAc structures elute earlier compared to the tri-antennary counterpart. Additionally, presence of diagnostic ions B-ion m/z 405 indicate LacDiNAc moiety and m/z 526 confirm LacNAc attached to the Mannose arm respectively. The core-fucosylation was confirmed by the presence of Z-ion m/z 350 and Y-ion 571 indicating the reducing end GlcNAc. Based on these criteria LacdiNAc structure containing glycans was assigned.

***Lectin staining and imaging***

Deparaffinization, antigen retrieval, and hydration of FFPE slides was carried out as described section for H&E imaging. Following deparaffinization and antigen retrieval, the slides were washed thrice by incubating for 3 min (each cycle) in Tris-buffered saline containing 0.05% Tween-20 (TBS-Tween). Wash buffer was removed using Kimwipes and a boundary was marked around the tissue section using a Pap pen (Vector Labs). The slides were washed in TBS-T for 3 min and blocked consecutively in 50 µl Streptavidin blocking solution (Vector Labs) 15 min at RT followed by 50 µl Biotin blocking solution (Vector Labs, USA) for 15 min at RT. The slides were washed in TBS-T to remove excess Biotin/Streptavidin contents and blocked with 100-200 µl 1X Carbo-Free blocking solution (Vector Labs, USA) in TBS-T for 30 min at RT. Blocking solution was removed and tissue sections were incubated overnight at 4°C in 200 µl biotin-conjugated lectin (Vector Labs, USA) AAL (5 µ/ml) and ECL, MAL, PNA, RCA, and VVA (20 µg/ml) in TBS-T containing 1X Carbo-Free™ blocking solution. For SNA staining, tissue sections were incubated in 200 µl Cy-5 conjugated SNA (20 µg/ml) for 2 h and washing steps were done as described below. Excess lectin was removed by washing thrice in PBS-T and bound lectin was tracked by incubated in 200 µl of Streptavidin-A568 (diluted 1:200 with TBS-T containing 1X Carbo-Free™ blocking solution) for 2 h at RT. After Streptavidin binding, tissue sections were incubated for 15 min in 200 µl Hoechst (ThermoFisher Scientific, USA) (10 µg/ml in TBS-T) to stain for nuclei. Slides were washed thrice in TBS-T, MilliQ water, dried, and mounted in ProLong™ Gold Antifade mountant (ThermoFisher Scientific). Lectin stained tissue samples were imaged on Mantra Snap 1.0 Build 1.0.7114.18437, 2019 Akoya Biosciences, INC.

**Figures**


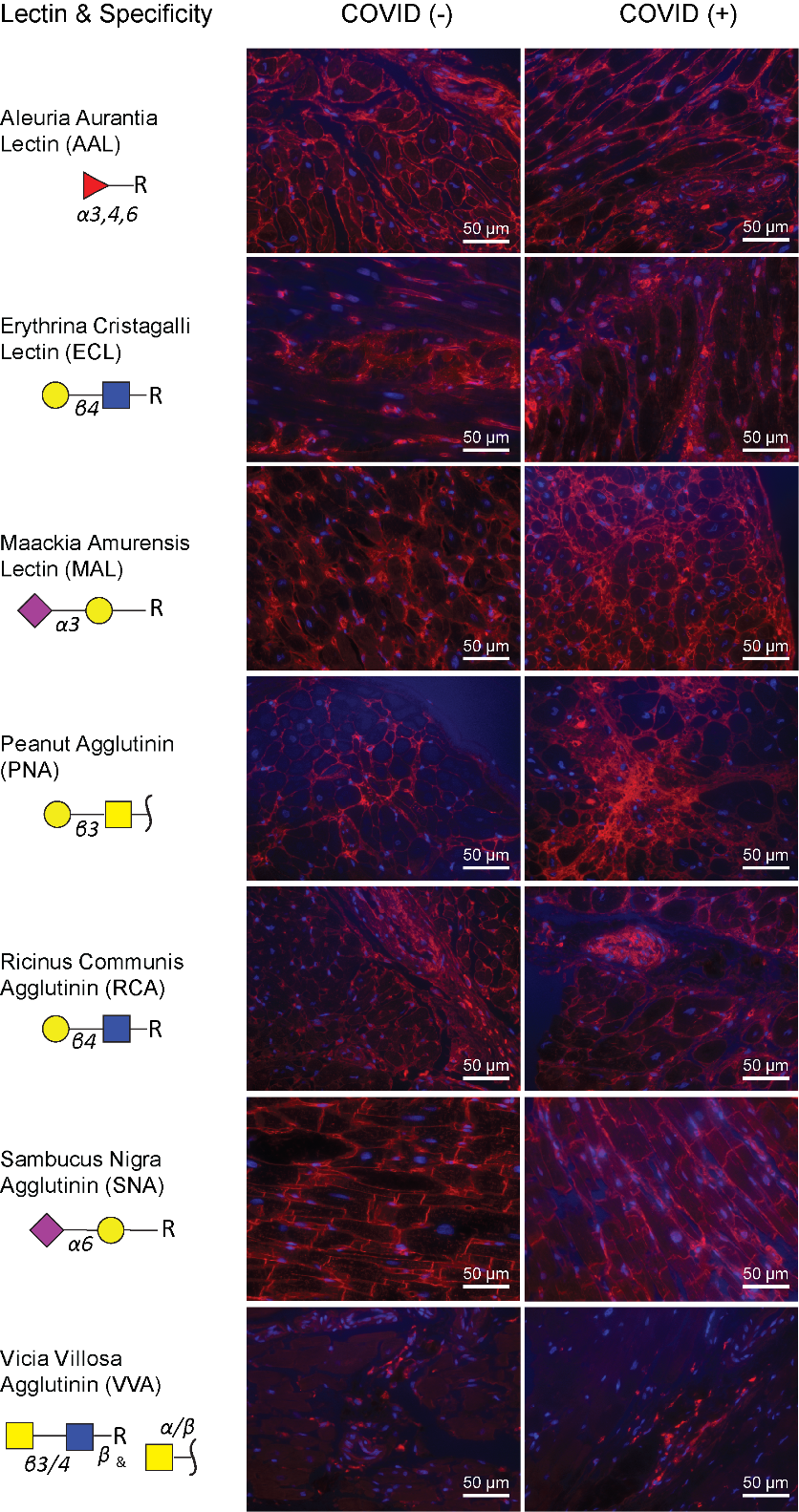


**Figure S1.** Immunofluorescent images of lectins in human cardiac tissue from COVID-19(+/-) patients. Images are representative of all patients (N=34).

Reference:

Liew, C.Y., Chen, J.-L., Lin, Y.-T., Luo, H.-S., Hung, A.-T., Magoling, B.J.A., Nguan, H.-S., Lai, C.P.-K., Ni, C.-K., 2024. Chromatograms and Mass Spectra of High-Mannose and Paucimannose N-Glycans for Rapid Isomeric Identifications. J. Proteome Res. 23, 939–955. https://doi.org/10.1021/acs.jproteome.3c00640
